# Supplementary material for: Thiocysteine lyases as polyketide synthase domains installing hydropersulfide into natural products and a hydropersulfide methyltransferase
Source: Nat Commun. 2021 Sep 28;12:5672. doi: 10.1038/s41467-021-25798-8 (PMC8479088; doi:10.1038/s41467-021-25798-8)
Supplement: Supplementary file 1 — Supplementary Information [file 41467_2021_25798_MOESM1_ESM.pdf]

Thiocysteine Lyases as Polyketide Synthase Domains Installing Hydropersulfide into Natural Products and a Hydropersulfide Methyltransferase

Authors: Song Meng,[1,a] Andrew D. Steele,[1,a] Wei Yan,[1,a] Guohui Pan,[1] Edward Kalkreuter,[1] Yu-Chen Liu,[1] Zhengren Xu,[1] Ben Shen[1,2,3,]*

Affiliation: [1]Department of Chemistry, [2]Department of Molecular Medicine, and [3]Natural Products Discovery Center at Scripps Research, The Scripps Research Institute, 130 Scripps Way, Jupiter, FL 33458

[a]These authors contributed equally
*Correspondence to: shenb@scripps.edu

Supplemantary Information

Table of Contents

Supplementary Methods S5-S7

1. Chemical synthesis of **9** and **13**. S5

2. Chemical synthesis of disulfide mimic substrate **17** and deacetylated mycothiol adducts **S15** and **S16**. S5

3. Chemical synthesis of peptides **19** and **21**. S5

4. Production of trisulfide compound **12**. S6

5. Large-scale enzymatic reaction to isolate **14** and **15**. S6

6. Structural elucidation of shunt products from *ΔgnmP* strain (SB21007). S7

Physicochemical properties of new compounds S8-S9

Supplementary Figures S10-S37

Supplementary Figure 1. Biosynthesis of sulfur-containing natural products. S12

Supplementary Figure 2. Previous studies suggesting *S-S* bond formation in a post-NRPS-PKS tailoring step in LNM biosynthesis. S13

Supplementary Figure 3. Hydropersulfides, as exemplified by LNM E (**8**) and GNM P (**7**), as common intermediates that undergo disproportionation to yield thiol congeners LNM E1 (**2**) and GNM B (**4**). S14

Supplementary Figure 4. DUF-SH didomains from LNM-type and non-LNM type BGCs catalyze similar chemistry. S15

Supplementary Figure 5. Bioinformatic analysis of DUF-SH-containing PKS proteins demonstrating the programmability of the DUF-SH didomain in the biosynthesis of diverse polyketide scaffolds. S17

Supplementary Figure 6. Phylogenetic analysis of GnmP showing the inability to predict GnmP as a *C*-, *O*-, *N*- or *S*-MT. S18

Supplementary Figure 7. Construction and confirmation of the Δ*gnmP* mutant strain SB21007. .... S19

Supplementary Figure 8. Establishment of GNM P (**7**) as the nascent product accumulated by the Δ*gnmP* mutant strain SB21007 and isolation and characterization of its degradation products. S20

Supplementary Figure 9. Elucidation of new metabolites **S1**, **S2**, **S3**, **S4**, and **S5** isolated from the *ΔgnmP* strain SB21007. .... S21

Supplementary Figure 10. Structural confirmation of the GNM P-mBB adduct **11**, L-cysteinyl-LNM E1 **13,** the methylated products of LNM E (**14**), its disproportionation trisulfide (**15**), and LNM E-mBB adduct **S9**. S22

Supplementary Figure 11. Overproduction in *E. coli* and purification of GnmP. S23

Supplementary Figure 12. Overproduction in *E. coli* and purification of the SH domains utilized in this study. S24

Supplementary Figure 13. Synthesis and structural characterization of **9**. S25

Supplementary Figure 14. Generation of the persulfide biosynthetic intermediate GNM P (**7**) using LnmJ-SH or WsmR-SH with substrate **9** and its trapping with mBB (**10**) or methylation with GnmP. S26

Supplementary Figure 15. Steady-state analysis of GnmT-SH, LnmJ-SH, and WsmR-SH with **9** as the substrate. S27

Supplementary Figure 16. Steady-state analysis of GnmP with the L-cysteinyl-GNM B adduct (**9**) as a surrogate substrate of GNM P (**7**) and GNM B (**4**) as an alternative substrate, demonstrating that GnmP prefers **7** over **4** as a substrate.. .... S28

Supplementary Figure 17. Structural confirmation of **S8** and **12**. S29

Supplementary Figure 18. Enzymatic reactions of GnmT-SH, LnmJ-SH, and WsmR-SH and GnmP, showing substrate promiscuity of both thiocysteine lyase and persulfide methyltransferase. S30

Supplementary Figure 19. Enzymatic reactions of GnmT-SH, LnmJ-SH, and WsmR-SH with **16**. S31

Supplementary Figure 20. Steady-state analysis of GnmT-SH, LnmJ-SH, and WsmR-SH, using the L-thiocysteine-polyketide adduct (**17**) as a substrate mimic in comparison with the L-cysteine-polyketide adduct (**16**), following Michaelis-Menten kinetics. S32

Supplementary Figure 21. A unified pathway for the biosynthesis of the LNM family of natural products. S33

Supplementary Figure 22. Overproduction in *E. coli* and purification of the CGL enzymes utilized in this study. S34

Supplementary Figure 23. CGL enzymes from LNM-, GNM-, and WSM-producing strains as a source of thiocysteine. S35

Supplementary Figure 24. Deacetylated mycothiol persulfide adducts are not viable substrates for LnmJ-SH, GnmT-SH, or WsmR-SH. S37

Supplementary Figure 25. Analysis of WsmR-SH-catalyzed peptide persulfide formation reactions. S38

HRMS and NMR Spectra S39-S100

Supplementary Tables S101-110

Supplementary Table 1. Strains used in this study........ S101

Supplementary Table 2. Plasmids and cosmids used in this study. S101

Supplementary Table 3. Primers used in this study. S102

Supplementary Table 4. ^{1}H NMR (600 MHz) and ^{13}C NMR (150 MHz) data of **9** in DMSO-d_6. .... S103

Supplementary Table 5. ^{1}H NMR (600 MHz) and ^{13}C NMR (150 MHz) data of **11** and **S9** in DMSO-d_6. . S104

Supplementary Table 6. ^{1}H NMR (600 MHz) and ^{13}C NMR (150 MHz) data of **13**, **14**, and **15** in DMSO-d_6. .... S105

Supplementary Table 7. ^{1}H NMR (600 MHz) and ^{13}C NMR (150 MHz) data of GNM P1 (**S1**) in methanol-d_4 and GNM P3 (**S3**) in DMSO-d_6. .... S106

Supplementary Table 8. ^{1}H NMR (600 MHz) and ^{13}C NMR (150 MHz) data of GNM P2 (**S2**), GNM P4 (**S4**), and GNM P5 (**S5**) in DMSO-d_6. .... S108

Supplementary Table 9. ^{1}H NMR (600 MHz) and ^{13}C NMR (150 MHz) data of **S8** in DMSO-d_6. .... S109

Supplementary Table 10. Characterized *C*-, *O*-, *N*-, and *S*-MTs from the literature used for the phylogenetic analysis. S110

Supplementary Table 11. Sequence comparison of representative cystathionine gamma lyase (CGL) proteins. S110

Supplementary References S111

Supplementary Methods

1. Chemical synthesis of 9 and 13.

To a stirring solution of GNM B (30 mg, 0.065 mmol) in H_2O (6 mL, pH 8.5) was added L-cysteine (40 mg, 0.330 mmol), and the reaction mixture was incubated at 30 °C for 3h. The desired product was purified by preparative HPLC, with the mobile phase consisting of solvent A (0.1% TFA in H_2O) and solvent B (0.1% TFA in CH_3CN) and eluted with a linear gradient of 5% B to 100% B for 30 min at a flow rate of 17 mL min^{-1}, yielding **9** as a yellow oil (32 mg, 85% yield).

To a stirring solution of LNM E1 (25 mg, 0.056 mmol) in H_2O (5 mL, pH 8.5) was added L-cysteine (34 mg, 0.281 mmol), and the reaction mixture was allowed to stir at room temperature overnight. The desired product was purified by HPLC using the same aforementioned condition yielding **13** as a yellow oil (23 mg, 72% yield).

2. Chemical synthesis of disulfide mimic substrate 17 and deacetylated mycothiol adducts S15 and S16.

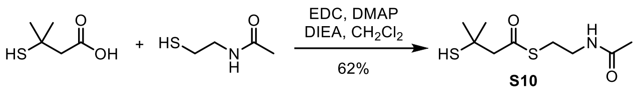

*S-(2-Acetamidoethyl) 3-mercapto-3-methylbutanethioate (**S10**).* 3-Mercapto-3-methylbutanoic acid (490 mg, 3.65 mmol), *N*-acetyl cysteamine (522 mg, 4.38 mmol), EDC (840 mg, 4.38 mmol), and DMAP (45 mg, 0.37 mmol) were dissolved in CH_2Cl_2 (10 mL) and stirred at room temperature for 1h, at which time Et_3N (0.77 mL, 4.38 mmol) was added. After stirring for another 3h, the reaction was poured into water and extracted 3x with CH_2Cl_2. The combined organic layers were washed with brine, dried over Na_2SO_4, filtered, and concentrated. The residue was purified by column chromatography (hexanes/EtOAc), yielding the title compound as a clear oil (530 mg, 62% yield).

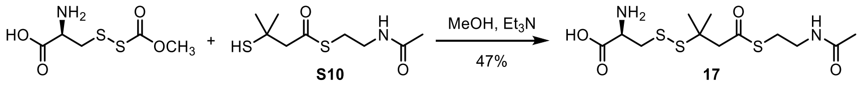

*S-((4-((2-Aacetamidoethyl)thio)-2-methyl-4-oxobutan-2-yl)thio)-L-cysteine (**17**).* To a solution of **S10** (30 mg, 0.127 mmol) in MeOH (2 mL) was added Et_3N (0.018 mL, 0.127 mmol), followed by *S*-(methoxycarbonylsulfenyl)cysteine (29 mg, 0.140 mmol). After 1h, the reaction was filtered and purified by preparative HPLC, with the mobile phase consisting of solvent A (H_2O) and solvent B (CH_3CN) and eluted using a solvent gradient from 5–95% B, yielding the title compound as a white residue (21 mg, 47% yield).

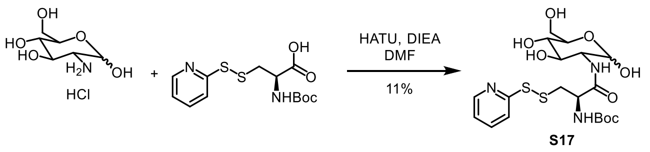

*tert-butyl ((R)-1-oxo-3-(pyridin-2-yldisulfaneyl)-1-(((3R,4R,5S,6R)-2,4,5-trihydroxy-6-(hydroxymethyl) tetrahydro-2H-pyran-3-yl)amino)propan-2-yl)carbamate (**S17**).* To a solution of D-glucosamine hydrochloride (51 mg, 0.237 mmol) and *N*-(tert-butoxycarbonyl)-*S*-(pyridin-2-ylthio)-L-cysteine (125 mg, 0.379 mmol)[1] dissolved in DMF (5 mL) at 0 °C was added HATU (151 mg, 0.397 mmol) and DIEA (0.11 mL, 0.635 mmol). The reaction was allowed to warm to room temperature and stirred overnight. The reaction was diluted with 8 mL of 0.1% formic acid in water and centrifuged at 10,000 *g* for 10 minutes. The supernatant was filtered through a 0.2 µm syringe filter and purified by preparative HPLC, with the mobile phase consisting of solvent A (0.1% TFA in water) and solvent B (0.1% TFA in CH_3CN) and eluted using a solvent gradient from 5–50 % B over 30

min at a flow rate of 17 mL min^{-1}, with the product eluting at 20.6 min. The solvent was removed by lyophilization to yield the title compound as a white solid (18 mg, 11% yield as a mixture of anomers).

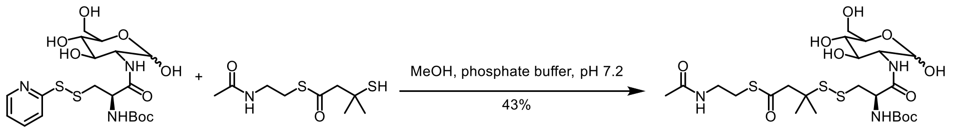

S17 S10 S18

*S-(2-acetamidoethyl) 3-(((R)-2-((tert-butoxycarbonyl)amino)-3-oxo-3-(((3R,4R,5S,6R)-2,4,5-trihydroxy-6-(hydroxymethyl)tetrahydro-2H-pyran-3-yl)amino)propyl)disulfaneyl)-3-methylbutanethioate (**S18**).* Disulfide **S17** (18 mg, 0.037 mmol) and thiol **S10** (17 mg, 0.073 mmol) were dissolved in MeOH (1.5 mL) and sodium phosphate buffer (500 mM, pH 7.2, 0.5 mL) was added followed by H_2O (0.5 mL). The reaction was stirred for 10 min at room temperature, at which time LC-MS analysis indicated consumption of the disulfide starting material. The reaction was diluted with MeOH to precipitate the buffer salts and the mixture was centrifuged at 10,000 *g* for 10 minutes. The supernatant was concentrated via rotary evaporation to ~2 mL volume and the mixture was purified by preparative HPLC, with the mobile phase consisting of solvent A (H_2O) and solvent B (CH_3CN) and eluted using a solvent gradient from 5–50 % B over 30 min at a flow rate of 17 mL min^{-1}, with the product eluting at 18 min. The solvent was removed by lyophilization to yield the title compound as a white solid (10 mg, 43% yield).

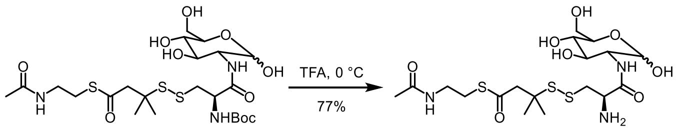

S18 S15

*S-(2-acetamidoethyl) 3-(((R)-2-amino-3-oxo-3-(((3R,4R,5S,6R)-2,4,5-trihydroxy-6-(hydroxymethyl)tetrahydro-2H-pyran-3-yl)amino)propyl)disulfaneyl)-3-methylbutanethioate (**S15**).* A vial containing **S18** (1.6 mg, 0.0026 mmol) was cooled on ice, and ice-cold TFA (0.5 mL) was added to the vial. The reaction was stirred on ice for 15 minutes, after which time the reaction was concentrated via rotary evaporation. Toluene was added and evaporated 3x to remove residual TFA, then the residue was dissolved in 2:1 MeOH:H_2O and purified by preparative HPLC, consisting of solvent A (0.1% TFA in H_2O) and solvent B (0.1% TFA in CH_3CN) and eluted using a solvent gradient from 5–50% B over 30 min at a flow rate of 17 mL min^{-1}, with the product eluting at 11 min. The solvent was removed by lyophilization, yielding the title compound as a white solid (1.0 mg, 77% yield, 2:1 mixture of anomers).

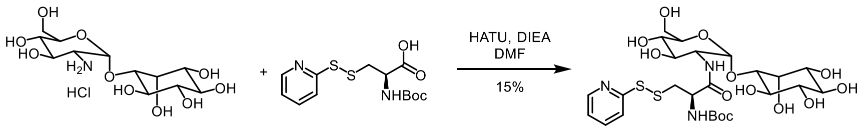

S19

*Tert-butyl ((R)-1-(((2R,3R,4R,5S,6R)-4,5-dihydroxy-6-(hydroxymethyl)-2-(((1S,2R,3R,4S,5S,6R)-2,3,4,5,6-pentahydroxycyclohexyl)oxy)tetrahydro-2H-pyran-3-yl)amino)-1-oxo-3-(pyridin-2-yldisulfaneyl)propan-2-yl)carbamate (**S19**).* To a solution of 1-*O*-(2-deoxy-2-amino-α-D-glucopyranosy)-D-*myo*-inositol hydrochloride (12 mg, 0.032 mmol)[2] and *N*-(tert-butoxycarbonyl)-*S*-(pyridin-2-ylthio)-L-cysteine (17 mg, 0.051 mmol)[1] dissolved in DMF (1.3 mL) at 0 °C was added HATU (20 mg, 0.054 mmol) and DIEA (15 µL, 0.086 mmol). The reaction was allowed to warm to room temperature and stirred overnight. The reaction was diluted with 6 mL of 0.1% formic acid in H_2O and centrifuged at 10,000 *g* for 10 min. The supernatant was filtered through a 0.2 µm syringe filter and purified by preparative HPLC, with the mobile phase consisting of solvent A (0.1% formic acid in H_2O) and solvent B (0.1% formic acid in CH_3CN) and eluted using a solvent gradient from 5–50 % B over 30

min at a flow rate of 17 mL min^{-1}, with the product eluting at 16 min. The solvent was removed by lyophilization to yield the title compound as a white solid (3.2 mg, 15% yield).

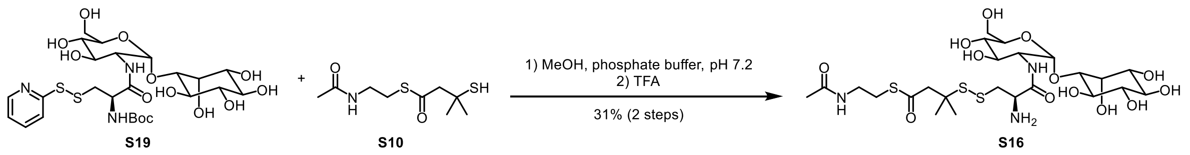

*S-(2-acetamidoethyl) 3-(((R)-2-amino-3-(((2R,3R,4R,5S,6R)-4,5-dihydroxy-6-(hydroxymethyl)-2-(((1S,2R,3R,4S,5S,6R)-2,3,4,5,6-pentahydroxycyclohexyl)oxy)tetrahydro-2H-pyran-3-yl)amino)-3-oxopropyl)disulfaneyl)-3-methylbutanethioate (**S16**).* Disulfide **S19** (3.2 mg, 0.0049 mmol) and thiol **S10** (4.1 mg, 0.017 mmol) were dissolved in MeOH (1.5 mL) and sodium phosphate buffer (500 mM, pH 7.2, 0.5 mL), and H_2O (0.5 mL) was added. The reaction was stirred for 10 min at room temperature, at which time LC-MS analysis indicated consumption of the disulfide starting material. The reaction was diluted with methanol to precipitate the buffer salts and the mixture was centrifuged at 10,000 *g* for 10 min. The supernatant was concentrated via rotary evaporation to ~2 mL volume and the mixture was purified by preparative HPLC, with the mobile phase consisting of solvent A (H_2O) and solvent B (CH_3CN) and eluted using a solvent gradient from 5–50 % B over 30 min at a flow rate of 17 mL min^{-1}, with the product eluting at 18 min. The solvent was removed by lyophilization to yield the *N*-Boc intermediate that co-eluted with an impurity that was removed in the next step. A vial containing the *N*-Boc intermediate (2.4 mg, 0.0031 mmol) was cooled on ice, and ice-cold TFA (0.5 mL) was added to the vial. The reaction was stirred on ice for 15 min, after which time the reaction was concentrated via rotary evaporation. Toluene was added and evaporated 3x to remove residual TFA. The residue was then dissolved in 2:1 MeOH:H_2O (1.5 mL), and purified by preparative HPLC, with the mobile phase consisting of solvent A (0.1% TFA in H_2O) and solvent B (0.1% TFA in CH_3CN) and eluted using a solvent gradient from 5–50 % B over 30 min at a flow rate of 17 mL min^{-1}, with the product eluting at 10 min. The solvent was removed by lyophilization to yield the TFA salt of title compound as a white solid (1.1 mg, 31% yield over 2 steps).

3. Chemical synthesis of peptides 19 and 21.

Swelling of the resin: 2-Chlorotrityl chloride resin (287 mg) was swelled by stirring in anhydrous CH_2Cl_2 (4 mL) in a flame-dried flask for 30 min.

First amino acid coupling/deprotection: The solvent was removed, and a freshly prepared mixture of Fmoc-(OtBu)-Thr-OH (365 mg, 0.918 mmol), and DIEA (0.41 mL, 2.295 mmol) in DMF (10 mL) was added to the resin. The reaction was stirred at room temperature overnight. The suspension was transferred to a fritted syringe, which was used as the reaction vessel for the rest of the synthesis. The solvent was drained from the syringe, and the resin was washed with CH_2Cl_2 3x, DMF 3x, MeOH 3x, CH_2Cl_2 3x, then DMF 3x (10 mL/wash). 20% piperidine in DMF (8 mL) was added to the resin in the syringe, which was capped and shaken on an orbital shaker for 10 minutes. The piperidine/DMF solution was drained from the syringe and set aside. Another 8 mL of 20% piperidine/DMF was added, and the syringe was shaken for 10 min. The solution was drained from the syringe and combined with the first wash. A Fmoc cleavage assay was performed on the combined washes, which indicated 50% loading.

Peptide elongation: A solution of Fmoc-(OtBu)-Thr-OH (365 mg, 0.918 mmol, 4.0 eq), HATU (349 mg, 0.918 mmol, 4.0 eq), and DIEA (0.321 mL, 1.836 mmol, 8.0 eq) in DMF (10 mL) was added to the resin, and the syringe was shaken for 4h. The solution was drained from the syringe, and the resin was washed with CH_2Cl_2 3x, DMF 3x, and CH_2Cl_2 3x (10 mL/wash). 20% piperidine/DMF (8 mL) was added to the syringe and shaken for 10 min. The solution was removed and another portion of 20% piperidine/DMF (8 mL) was added to the syringe and shaken for 10 min. The resin was washed with CH_2Cl_2 3x, DMF 3x, and CH_2Cl_2 3x (10 mL/wash). This

procedure was repeated until a fully elongated peptide was obtained. The full batch of resin-bound protected peptide was stored at 4 °C, and small portions were taken to the next step as needed.

Cleavage and deprotection: To the resin-bound protected peptide (54 mg) was added a solution of 95:2.5:2.5 (v:v:v) TFA:H_2O:TIPS. The reaction was stirred for 2h at room temperature. The reaction was filtered, and the solvent was removed by rotary evaporation. The residue was dissolved in a minimal amount of MeOH and added dropwise to a falcon tube containing cold Et_2O (20 mL). The tube was kept in a -20 °C freezer for 30 minutes, then centrifuged at 10,000 *g* for 10 min. The supernatant was decanted, and the precipitated peptide (11 mg) was used directly in the next step.

Air-mediated cysteine disulfide formation: The crude peptide from the previous step was dissolved in MeOH (1 mL) and an aqueous solution of 0.2 M NaOH containing 0.2 M cysteine (0.08 mL) was added. The reaction was stirred open to the air for 4h. The reaction was acidified to pH 2 with 5% TFA in water and filtered. The filtrate was purified by preparative HPLC, with the mobile phase consisting of solvent A (0.1% TFA in H_2O) and solvent B (0.1% TFA in CH_3CN) and eluted using a solvent gradient from 0–20 % B over 30 min at a flow rate of 17 mL min^{-1}, yielding compound **19** as a white solid. $[\alpha]_D^{25}$ -52.0 (*c* 0.26, MeOH); ^{1}H-NMR (methanol-d_4): δ 4.71 (dd, J = 4.7, 9.0 Hz, 1H), 4.44 (t, J = 5.8 Hz, 1H), 4.37 (d, J = 4.4 Hz, 1H), 4.33 (d, J = 3.0 Hz, 1H), 4.23-4.26 (m, 2H), 4.10 (m, 1H), 3.91 (dd, J = 6.8, 14.1 Hz, 1H), 3.77 (dd, J = 5.8, 10.9 Hz, 1H), 3.73 (dd, J = 6.1, 10.9 Hz, 1H), 3.36 (dd, J = 3.9, 14.9 Hz, 1H), 3.26 (dd, J = 4.7, 14.0 Hz, 1H), 3.09 (dd, J = 8.1, 14.9 Hz, 1H), 2.97 (dd, J = 8.9, 14.0 Hz, 1H), 1.44 (d, J = 6.8 Hz, 3H), 1.13 (d, J = 6.4 Hz, 3H), 1.10 (d, J = 6.5 Hz, 3H); ^{13}C-NMR (methanol-d_4): δ 172.1, 171.2, 171.0, 170.9, 169.8, 169.0, 115.6, 113.7, 67.0, 67.0, 61.6, 58.7, 57.9, 55.2, 52.6, 48.8, 38.8, 37.6, 19.1, 18.5, 16.2; HR-ESI-MS *m/z* 601.1957 $[M + H]^+$ (calcd for $C_{20}H_{37}N_6O_{11}S_2$, 601.1956).

Peptide **21** was synthesized using identical procedures to that of **19**, using Fmoc-Gly-OH in the first coupling step, followed by Fmoc-(OtBu)-Glu-OH, Fmoc-(Trt)-Cys-OH, Fmoc-(Trt)-Lys-OH, and Fmoc-(OtBu)-Tyr-OH in the subsequent coupling steps, in that order. $[\alpha]_D^{25}$ -10.0 (*c* 0.24, MeOH); ^{1}H-NMR (methanol-d_4): δ 7.14 (d, J = 8.4 Hz, 2H), 6.80 (d, J = 8.4 Hz, 2H), 4.71 (m, 1H), 4.49 (m, 1H), 4.36-4.43 (m, 2H), 4.12 (m, 1H), 4.00 (d, J = 17.2 Hz, 1H), 3.89 (d, J = 17.2 Hz, 1H), 3.50 (m, 1H), 3.36 (m, 1H), 3.22 (d, J = 8.1 Hz, 1H), 3.18 (d, J = 7.5 Hz, 1H), 3.07 (dd, J = 9.1, 14.3 Hz, 1H), 2.93-2.99 (m, 3H), 2.48 (t, J = 7.9 Hz), 2.16 (m, 1H), 1.67-2.03 (m, 5H), 1.49 (m, 2H); ^{13}C-NMR (methanol-d_4): δ 176.7, 173.9, 173.6, 172.7, 172.7, 169.9, 158.2, 131.7, 125.9, 116.9, 55.8, 54.7, 54.1, 53.9, 49.9, 49.6, 41.8, 40.5, 40.0, 37.8, 32.6, 30.9, 28.6, 28.1, 23.5; HR-ESI-MS *m/z* 718.2535 $[M + H]^+$ (calcd for $C_{28}H_{44}N_7O_{11}S_2$, 718.2535).

4. Production of trisulfide compound 12.

For production of **12**, each incubation was performed in 50 mM sodium phosphate, pH 8.0, containing 20 mM KCl, 0.2 mM PLP, 2 mM SAM, 1 mM **9**, 220 µM WsmR-SH and 10 µM GnmP in a total volume of 50 µL. After incubation at 28 °C for 1 min, 250 µL of methanol was added to quench the reaction. The reaction mixture was then centrifuged at 12,000 *g* for 10 min and the supernatant was injected and analyzed by LC-MS with Method I.

5. Large-scale enzymatic reaction to isolate 14 and 15.

For isolation of **14** and **15**, the large-scale enzymatic reaction was performed in 50 mM sodium phosphate, pH 8.0, containing 20 mM KCl, 0.2 mM PLP, 2 mM SAM, 5 mM **13**, 220 µM WsmR-SH and 30 µM GnmP in a total volume of 10 ml. After incubation at 28 °C for 2 hr, 30 ml of methanol was added to quench the reaction. The reaction mixture was then centrifuged at 12,000 *g* for 20 min and the supernatant was injected and analyzed by LC-MS with Method I. The reaction mixture was concentrated, dissolved in CH_3CN and purified by HPLC. The isolation was conducted using a 35 min solvent gradient from 20–100% CH_3CN in H_2O containing 0.1% formic acid at a flow rate of 3 mL min^{-1} to give **14** (3.3 mg) and **15** (0.3 mg).

6. Structural elucidation of shunt products from *ΔgnmP* strain (SB21007).

GNM P1-P5 (**S1**-**S5**) were isolated based on their characteristic UV absorption at 280~300 nm, indicating that they are all GNM B (**4**) congeners.

GNM P1 (**S1**) was isolated as a yellow oil and determined to have the molecular formula of $C_{41}H_{57}N_{3}O_{5}S_{3}$ by its $[M -H]^{-}$ ion peak at 941.2988 on HRESIMS. The 1D and 2D NMR spectra of **S1** revealed a GNM B moiety, which was confirmed by ^{1}H-^{1}H COSY and HMBC experiments. The presence of an N-acetylcysteine unit was elucidated by HMBC correlations from H-26 to C-25, C-27 and C-28, and those from H-29 to C28. Considering the molecular formula, a mycothiol (MSH)-conjugate structure was proposed by the observation of the N-acetylcysteine unit, which was seen as a "detoxification label" in MSH-related compounds. This assumption was further confirmed by comparison of the NMR data of **S1** with those reported in literature.[3,4]

GNM P2 (**S2**) was obtained as a yellow oil. The HRESIMS of **S2** produced the $[M-H]^{-}$ ion at *m/z* 505.1473, indicating the molecular formula was $C_{24}H_{30}N_{2}O_{6}S_{2}$, which contains three more oxygen atoms than **4**. The ^{1}H and ^{13}C NMR spectra of **S2** resembled those of **4**, with the exception that the resonance attributed to H-2 at δ_H 2.55 (1H, d, J = 13.1 Hz) and 2.21 (1H, d, J = 13.1 Hz) in **4** shifted to δ_H 2.69 (2H, s) in **S2**, and resonances attributed to C-2, C-3, and C-4 at δ_C 50.1, 46.6, and 41.6 in **4** shifted to δ_C 40.0, 59.3, and 31.2 in **S2**, respectively. Furthermore, **S2** and **4** had the same correlations in their COSY, HSQC, and HMBC spectra. These results suggested that the only difference between the two compounds was that the thiol group in **4** was oxidized in **S2**. Therefore, **S2** was established as a sulfonic acid analog of **4**.

GNM P3 (**S3**) was purified as a yellow oil and the molecular formula was determined to be $C_{29}H_{37}N_{3}O_{6}S_{3}$ as evidenced from $[M - H]^{-}$ ion at *m/z* 618.1772 in its HRESIMS. The 1D NMR spectra of **S3** showed two sets of signals, implying that it contained two rotamers, which was also observed in GNM A (**3**). Careful comparison of the NMR data of both rotamers to those of **3** and **4** indicated the N-acetylcysteine motif in **S3**, as confirmed by ^{1}H-^{1}H COSY correlations between H-26/ H-25 and H-26/NH-26, together with HMBC correlations from H-26 to C-25, C-27 and C-28, and those from H-29 to C28. Thus, **S3** was elucidated as an N-acetylcysteine adduct of GNM B, which could be rationalized as the degradation product of **S1**.

GNM P4 (**S4**) was isolated as a white powder and had a molecular formula of $C_{24}H_{28}N_{2}O_{4}S_{2}$ according to the $[M - H]^{-}$ ion at *m/z* 471.1421 in its HRESIMS. The ^{1}H and ^{13}C NMR spectra of **S4** resembled those of **4**, with the major difference located at from C2-C4. The resonance attributed to H-2 (δ_H 2.55 and 2.21) and H-4 (δ_H 1.94 and 1.69) in **3** were changed to H-2 (δ_H 2.87 and 2.59) and H-4 (δ_H 1.45 and 0.39) in **S4**, respectively. The resonances attributed to C-2, C-3, and C-4 at δ_C 50.1, 46.6, and 41.6 in GNM B was changed to δ_C 37.6, 62.0, and 26.8 in **S4**, respectively. Additionally, the NH signal in **4** is absent in ^{1}H NMR spectrum of **S4**. Taken together, we proposed that the thiol group was oxidized and then formed the S(O)-N bond to account for the molecular formula.

GNM P5 (**S5**), obtained as a white powder, was shown to have the same molecular formula as **S4** by the $[M - H]^{-}$ ion at *m/z* 471.1417 in HRESIMS. The ^{1}H-^{1}H COSY, HMBC and ROESY data of **S5** were almost identical to those of **S4**, indicating that they are structurally similar. The difference lies in the rotation of the thiazole ring, evidenced by the observation that the ROESY crosspeak between H-11 (δ_H 6.73) and H-15 (δ_H 7.50) in **S4** was replaced by a correlation between H-13 (δ_H 6.38) and H-15 (δ_H 7.45) in **S5**. Therefore, **S5** was determined as an isomer product of **S4**.

Physicochemical properties of new compounds.

9. Yellowish oil; $[\alpha]_D^{25}$ -60.0 (*c* 0.06, MeOH); UV (MeOH) λ_{max} (log ε) 245 (1.90), 289 (4.06); ^{1}H and ^{13}C NMR data see **Supplementary Table 4**; HR-ESI-MS *m/z* 576.1663 $[M - H]^{-}$ (calcd for $C_{27}H_{34}N_{3}O_{5}S_{3}$, 576.1666).

11. Yellowish oil; UV (MeOH) λ_{max} (log ε) 210 (0.50), 240 (4.06), 282 (3.91), 388 (1.20); ^{1}H and ^{13}C NMR data see **Supplementary Table 5**; HR-ESI-MS *m/z* 679.2085 [M - H]$^-$ (calcd for $C_{34}H_{39}N_4O_5S_3$, 679.2088).

13. Yellowish oil; $[\alpha]_D^{25}$ -65.6 (*c* 0.5, MeOH); UV (MeOH) λ_{max} (log ε) 242 (1.81), 330 (4.06) nm; ^{1}H and ^{13}C NMR data see **Supplementary Table 6**; HR-ESI-MS *m/z* 566.1461 [M - H]$^-$ (calcd for $C_{25}H_{32}N_3O_6S_3$, 566.1459).

14. White powder; $[\alpha]_D^{25}$ -18.5 (*c* 0.1, MeOH); UV (MeOH) λ_{max} (log ε) 329 (4.08) nm; ^{1}H and ^{13}C NMR data see **Supplementary Table 6**; HR-ESI-MS *m/z* 493.1297 [M - H]$^-$ (calcd for $C_{23}H_{29}N_2O_4S_3$, 493.1295).

15. White powder; UV (MeOH) λ_{max} (log ε) 329 (4.13) nm; ^{1}H and ^{13}C NMR data see **Supplementary Table 6**; HR-ESI-MS *m/z* 525.1017 [M - H]$^-$ (calcd for $C_{23}H_{29}N_2O_4S_4$, 525.1016).

17. White solid; $[\alpha]_D^{25}$ -160.0 (*c* 1.0, MeOH); ^{1}H-NMR (methanol-*d*$_4$): δ 3.77 (dd, *J* = 3.3, 9.6 Hz, 1H), 3.35 (dd, *J* = 3.3, 14.2 Hz, 1H), 3.25-3.29 (m, 2H), 2.95-2.99 (m, 3H), 2.89 (s, 2H), 1.87 (s, 3H), 1.42 (s, 3H), 1.41 (s, 3H); ^{13}C-NMR (methanol-*d*$_4$): δ 195.8, 172.1, 170.9, 53.7, 53.6, 49.4, 40.9, 38.6, 28.2, 26.6, 26.3, 21.1; HR-ESI-MS *m/z* 353.0665 [M - H]$^-$ (calcd for $C_{12}H_{21}N_2O_4S_3$, 353.0669).

19. White solid; $[\alpha]_D^{25}$ -10.0 (*c* 0.24, MeOH); ^{1}H-NMR (methanol-*d*$_4$): δ 7.14 (d, *J* = 8.4 Hz, 2H), 6.80 (d, *J* = 8.4 Hz, 2H), 4.71 (m, 1H), 4.49 (m, 1H), 4.36-4.43 (m, 2H), 4.12 (m, 1H), 4.00 (d, *J* = 17.2 Hz, 1H), 3.89 (d, *J* = 17.2 Hz, 1H), 3.50 (m, 1H), 3.36 (m, 1H), 3.22 (d, *J* = 8.1 Hz, 1H), 3.18 (d, *J* = 7.5 Hz, 1H), 3.07 (dd, *J* = 9.1, 14.3 Hz, 1H), 2.93-2.99 (m, 3H), 2.48 (t, *J* = 7.9 Hz), 2.16 (m, 1H), 1.67-2.03 (m, 5H), 1.49 (m, 2H), ; ^{13}C-NMR (methanol-*d*$_4$): δ 176.7, 173.9, 173.6, 172.7, 172.7, 169.9, 158.2, 131.7, 125.9, 116.9, 55.8, 54.7, 54.1, 53.9, 49.9, 49.6, 41.8, 40.5, 40.0, 37.8, 32.6, 30.9, 28.6, 28.1, 23.5; HR-ESI-MS *m/z* 718.2535 [M + H]$^+$ (calcd for $C_{28}H_{44}N_7O_{11}S_2$, 718.2535).

21. White solid; $[\alpha]_D^{25}$ -52.0 (*c* 0.26, MeOH); ^{1}H-NMR (methanol-*d*$_4$): δ 4.71 (dd, *J* = 4.7, 9.0 Hz, 1H), 4.44 (t, *J* = 5.8 Hz, 1H), 4.37 (d, *J* = 4.4 Hz, 1H), 4.33 (d, *J* = 3.0 Hz, 1H), 4.23-4.26 (m, 2H), 4.10 (m, 1H), 3.91 (dd, *J* = 6.8, 14.1 Hz, 1H), 3.77 (dd, *J* = 5.8, 10.9 Hz, 1H), 3.73 (dd, *J* = 6.1, 10.9 Hz, 1H), 3.36 (dd, *J* = 3.9, 14.9 Hz, 1H), 3.26 (dd, *J* = 4.7, 14.0 Hz, 1H), 3.09 (dd, *J* = 8.1, 14.9 Hz, 1H), 2.97 (dd, *J* = 8.9, 14.0 Hz, 1H), 1.44 (d, *J* = 6.8 Hz, 3H), 1.13 (d, *J* = 6.4 Hz, 3H), 1.10 (d, *J* = 6.5 Hz, 3H); ^{13}C-NMR (methanol-*d*$_4$): δ 172.1, 171.2, 171.0, 170.9, 169.8, 169.0, 67.0, 67.0, 61.6, 58.7, 57.9, 55.2, 52.6, 48.8, 38.8, 37.6, 19.1, 18.5, 16.2; HR-ESI-MS *m/z* 601.1957 [M + H]$^+$ (calcd for $C_{20}H_{37}N_6O_{11}S_2$, 601.1956).

GNM P1 (**S1**). Yellowish oil; $[\alpha]_D^{25}$ -33.0 (*c* 0.14, MeOH); UV (MeOH) λ_{max} (log ε) 245 (1.90), 289 (4.06); ^{1}H and ^{13}C NMR data see **Supplementary Table 7**; HR-ESI-MS *m/z* 941.2982 [M - H]$^-$ (calcd for $C_{41}H_{57}N_4O_{15}S_3$, 941.2988).

GNM P2 (**S2**). Yellowish oil; $[\alpha]_D^{25}$ 29.0 (c 0.1, MeOH); UV (MeOH) λmax (log ε) 285 (4.49) nm; ^{1}H and ^{13}C NMR data see **Supplementary Table 8**; HR-ESI-MS m/z 505.1476 [M - H]- (calcd for $C_{24}H_{30}N_2O_6S_2$, 505.1473).

GNM P3 (**S3**). Yellowish oil; $[\alpha]_D^{25}$ -58.0 (*c* 0.16, MeOH); UV (MeOH) λ_{max} (log ε) 245 (2.10), 315 (4.16); ^{1}H and ^{13}C NMR data see **Supplementary Table 7**; HR-ESI-MS *m/z* 618.1767 [M - H]$^-$ (calcd for $C_{29}H_{36}N_3O_6S_3$, 618.1772).

GNM P4 (**S4**). White powder; $[\alpha]_D^{25}$ -5.0 (*c* 0.04, MeOH); UV (MeOH) λ_{max} (log ε) 285 (4.09) nm; ^{1}H and ^{13}C NMR data see **Supplementary Table 8**; HR-ESI-MS *m/z* 471.1421 [M - H]$^-$ (calcd for $C_{24}H_{27}N_2O_4S_2$, 471.1418).

GNM P5 (**S5).** White powder; $[\alpha]_D^{25}$ -2.5 (*c* 0.05, MeOH); UV (MeOH) λ_{max} (log ε) 285 (4.21) nm, 303 (3.89) nm; ^{1}H and ^{13}C NMR data see **Supplementary Table 8**; HR-ESI-MS *m/z* 471.1417 [M - H]$^-$ (calcd for $C_{24}H_{27}N_2O_4S_2$, 471.1418).

S8. Yellowish oil; $[\alpha]_D^{25}$ 2.5 (*c* 0.04, MeOH); UV (MeOH) λ_{max} (log ε) 290 (4.21); ^{1}H and ^{13}C NMR data see **Supplementary Table 9**; HR-ESI-MS *m/z* 471.1781 [M - H]$^-$ (calcd for $C_{25}H_{31}N_2O_3S_2$, 471.1782).

S9. Yellowish oil; UV (MeOH) λ_{max} (log ε) 240 (4.06), 328 (4.12); ^{1}H and ^{13}C NMR data see **Supplementary Table 5**; HR-ESI-MS *m/z* 669.1882 [M - H]$^-$ (calcd for $C_{32}H_{37}N_4O_6S_3$, 669.1881).

S15; White solid; $[\alpha]_D^{25}$ -26.8 (*c* 0.3, MeOH); ^{1}H NMR (400 MHz, methanol-d_4, 2:1 mixture of anomers) δ 5.16 (d, J = 3.5 Hz, 0.66H), 4.66 (d, J = 8.3 Hz, 0.33H), 4.20 (dd, J = 9.9, 3.8 Hz, 0.66H), 4.08 (dd, J = 9.7, 4.1 Hz, 0.33H), 3.91-3.85 (m, 1H), 3.84-3.78 (m, 1.30H), 3.75-3.59 (m, 2.12H), 3.53-3.46 (m, 0.66H), 3.46-3.38 (m, 1H), 3.38-3.32 (m, 3H), 3.14-2.95 (m, 3H), 2.94 (s, 1H), 2.92 (s, 0.66H), 1.93 (s, 3H), 1.48 (s, 4H), 1.46 (s, 2H); ^{13}C NMR (100 MHz, methanol-d_4) δ 198.2, 197.7, 173.5, 169.0, 168.9, 96.6, 92.6, 78.2, 75.9, 73.2, 72.8, 72.5, 72.2, 62.7, 59.0, 56.1, 54.8, 54.6, 54.2, 53.8, 50.8, 50.6, 49.8, 42.1, 41.7, 39.9, 29.8, 29.7, 28.7, 28.2, 27.9, 27.7, 22.6; HR-ESI-MS *m/z* 516.1502 [M + H]$^+$ (calcd for $C_{18}H_{34}N_3O_8S_3$, 516.1503).

S16. White solid, TFA salt; $[\alpha]_D^{25}$ 3.7 (*c* 0.3, MeOH); ^{1}H NMR (600 MHz, D_2O) δ 5.10 (d, *J* = 3.6 Hz, 1H), 4.31 (dd, *J* = 7.8, 5.0 Hz, 1H), 4.19 (t, *J* = 2.7 Hz, 1H), 4.02-3.97 (m, 1H), 3.95 (ddd, *J* = 10.1, 4.5, 2.3 Hz, 1H), 3.84-3.75 (m, 3H), 3.71 (t, *J* = 9.7 Hz, 1H), 3.66-3.59 (m, 2H), 3.53-3.46 (m, 2H), 3.39-3.32 (m, 3H), 3.29 (t, *J* = 9.4 Hz, 1H), 3.21 (dd, *J* = 14.7, 8.0 Hz, 1H), 3.06-3.03 (m, 2H), 2.99-2.93 (m, 2H), 1.94 (s, 3H), 1.42 (s, 6H); ^{13}C NMR (150 MHz, D_2O) δ 200.4, 174.2, 168.3, 93.6, 74.9, 74.1, 72.1, 72.0, 71.0, 70.9, 70.7, 70.0, 68.0, 60.3, 53.7, 53.3, 52.7, 49.8, 40.7, 38.4, 28.5, 27.0, 26.9, 21.9; HR-ESI-MS *m/z* 678.2031 [M + H]$^+$ (calcd for $C_{24}H_{44}N_3O_{13}S_3$, 678.2031).

S17. White solid; $[\alpha]_D^{25}$ -1.9 (*c* 0.2, MeOH); ^{1}H NMR (400 MHz, methanol-d_4, 3:1 mixture of anomers, major anomer data shown) δ 8.75 (d, *J* = 4.2 Hz, 1H), 8.10-7.99 (m, 2H), 7.52-7.47 (m, 1H), 5.11 (d, *J* = 3.0 Hz, 1H), 4.60 (s, 2H), 4.44-4.38 (m, 1H), 3.88-3.77 (m, 3H), 3.73-3.64 (m, 2H), 3.39-3.33 (m, 1H), 3.30-3.22 (m, 2H), 3.15-3.07 (m, 1H) 1.71 (s, 9H); ^{13}C NMR (150 MHz, methanol-d_4) δ 173.1, 160.8, 157.8, 150.6, 139.1, 122.5, 121.7, 92.5, 81.0, 73.2, 72.9, 72.4, 62.8, 56.0, 55.3, 43.0, 28.7; HR-ESI-MS *m/z* 492.1468 [M + H]$^+$ (calcd for $C_{19}H_{30}N_3O_8S_2$, 492.1469).

S18. White solid; $[\alpha]_D^{25}$ -0.4 (*c* 0.50, MeOH); ^{1}H NMR (600 MHz, methanol-d_4) δ 5.11 (d, J = 3.0 Hz, 1H), 4.60 (s, 1H), 4.40 (dd, J = 9.1, 4.0 Hz, 1H), 3.84-3.78 (m, 2H), 3.73-3.66 (m, 2H), 3.40-3.32 (m, 3H), 3.24 (dd, J = 13.4, 4.0 Hz, 1H), 3.04-3.00 (m, 2H), 2.95 (dd, J = 13.2, 9.6 Hz, 1H), 2.91 (s, 2H), 1.93 (s, 3H), 1.47 (s, 9H), 1.43 (s, 6H); ^{13}C NMR (150 MHz, methanol-d_4) δ 197.6, 173.5, 157.9, 92.5, 81.0, 73.2, 72.9, 72.4, 62.8, 56.0, 55.6, 55.0, 50.3, 49.6, 49.0, 43.5, 40.0, 29.6, 28.7, 28.0, 27.9, 22.6; HR-ESI-MS *m/z* 616.2027 [M + H]$^+$ (calcd for $C_{23}H_{42}N_3O_{10}S_3$, 616.2032).

S19. White solid; $[\alpha]_D^{25}$ 0.1 (*c* 1.6, MeOH); ^{1}H NMR (600 MHz, methanol-d_4) δ 8.49 (d, *J* = 4.9 Hz, 1H), 7.82 – 7.77 (m, 2H), 7.24 (td, *J* = 5.3, 2.7 Hz, 1H), 4.95 (d, *J* = 3.3 Hz, 1H), 4.60 (br s, 1H), 4.34 (dd, *J* = 9.4, 4.4 Hz, 1H), 4.10-4.07 (m, 1H), 4.04-3.99 (m, 1H), 3.92 (dd, *J* = 10.6, 3.3 Hz, 1H), 3.82 (dd, *J* = 11.9, 2.1 Hz, 1H), 3.79-3.75 (m, 1H), 3.74 (t, *J* = 9.6 Hz, 1H), 3.68 (dd, *J* = 11.9, 5.4 Hz, 1H), 3.64 (t, *J* = 9.5 Hz, 1H), 3.52 (dd, *J* = 9.9, 2.6 Hz, 1H), 3.39 (t, *J* = 9.4 Hz, 1H), 3.28 (dd, *J* = 14.0, 4.6 Hz, 2H), 3.20 (t, *J* = 9.3 Hz, 1H), 3.08 (dd, *J* = 13.8, 9.6 Hz, 1H), 1.47 (s, 9H); ^{13}C NMR (150 MHz, methanol-d_4) δ 173.4, 160.8, 157.9, 150.6, 139.2, 122.6, 121.7, 95.6, 81.2, 77.4, 76.5, 74.1, 73.7, 73.2, 72.8, 72.8, 72.2, 69.9, 62.6, 55.6, 55.4, 42.8, 28.8; HR-ESI-MS *m/z* 654.1997 [M + H]$^+$ (calcd for $C_{25}H_{40}N_3O_{13}S_2$, 654.1997).

Supplementary Figures

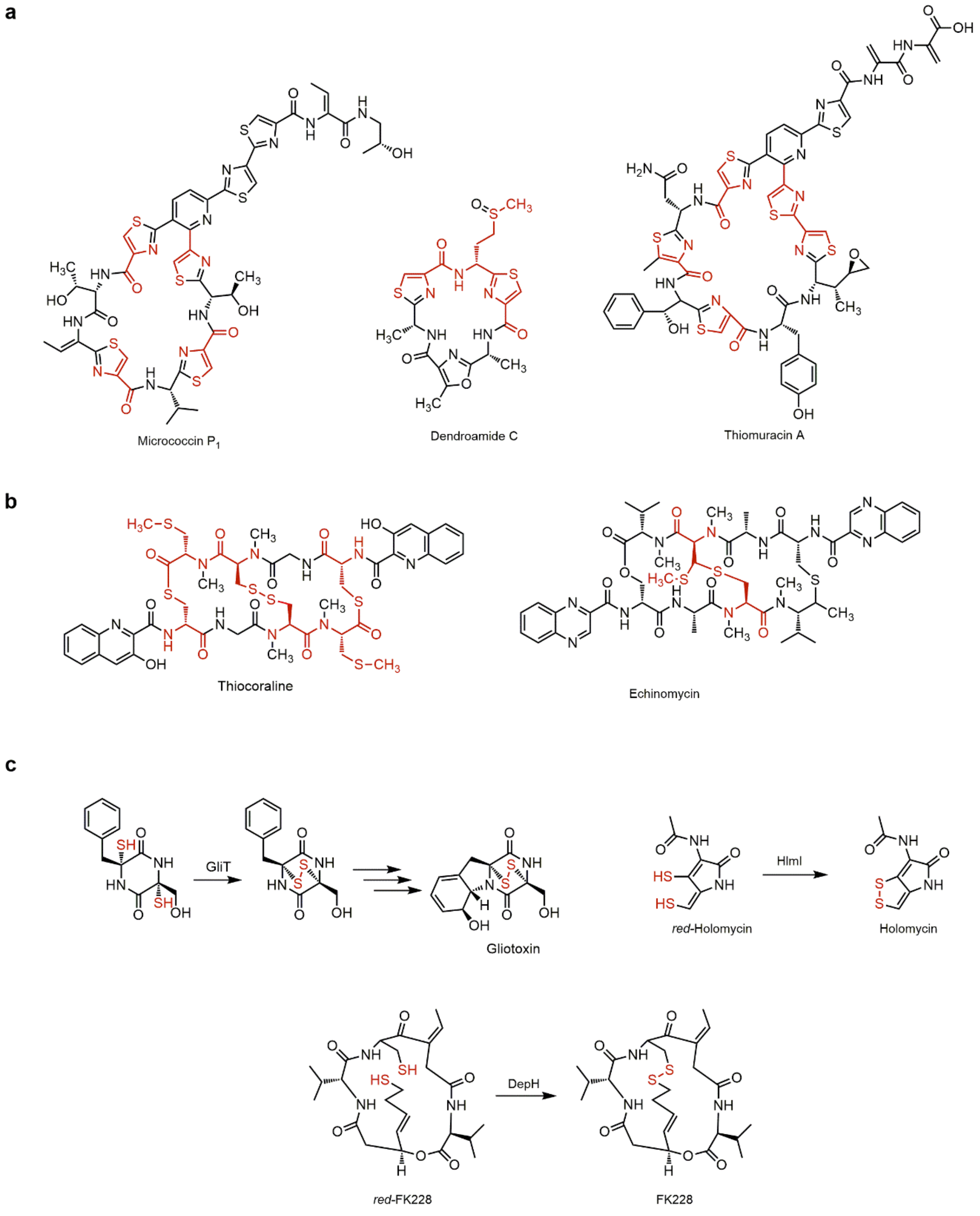

Supplementary Figure 1. Biosynthesis of sulfur-containing natural products. **a**, Peptide natural products containing Cys/Met of RiPP biosynthetic origin. **b**, Peptide natural products containing Cys/Met of NRPS biosynthetic origin. **c**, Formation of S-S bonds in natural products by oxidation of two sulfhydryl (-SH) groups.

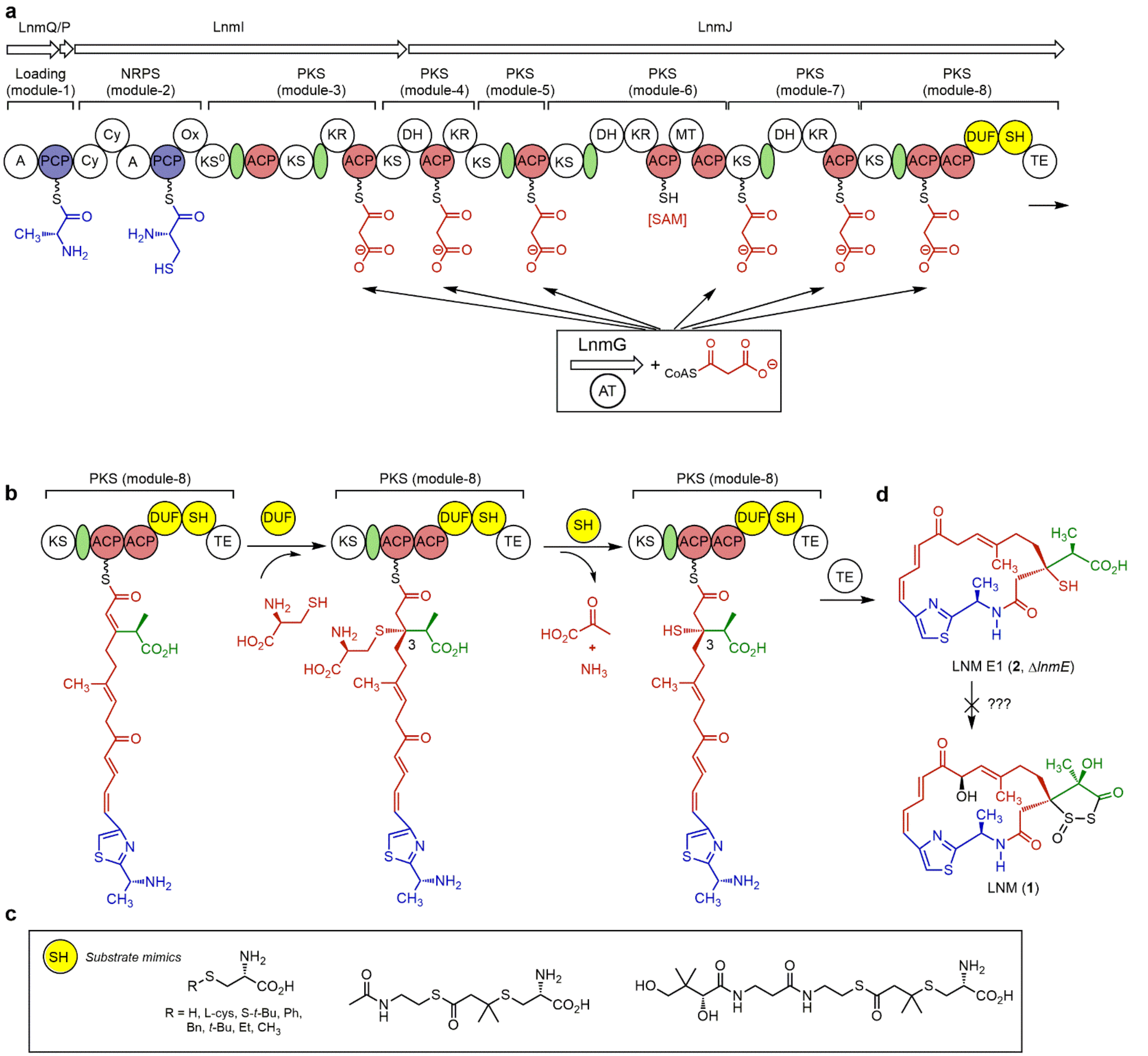

Supplementary Figure 2. Previous studies suggesting *S-S* bond formation in a post-NRPS-PKS tailoring step in LNM biosynthesis.[5,6] **a**, The LNM hybrid NRPS-PKS assembly line featuring a DUF-SH didomain, **b**, with the DUF domain to generate an L-cysteine-polyketide adduct and the SH domain to cleave the cysteine C-S bond of the adduct, installing a -SH group into a polyketide scaffold and **c**, demonstration of the LnmJ-SH domain as a cysteine lyase in vitro using varying substrate mimics. **d**, Isolation of LNM E1 (**2**) from both *S. atroolivaceus* S-140 wild-type and selected mutant strains but failure to establish LNM E1 as an intermediate for LNM biosynthesis despite extensive effort.

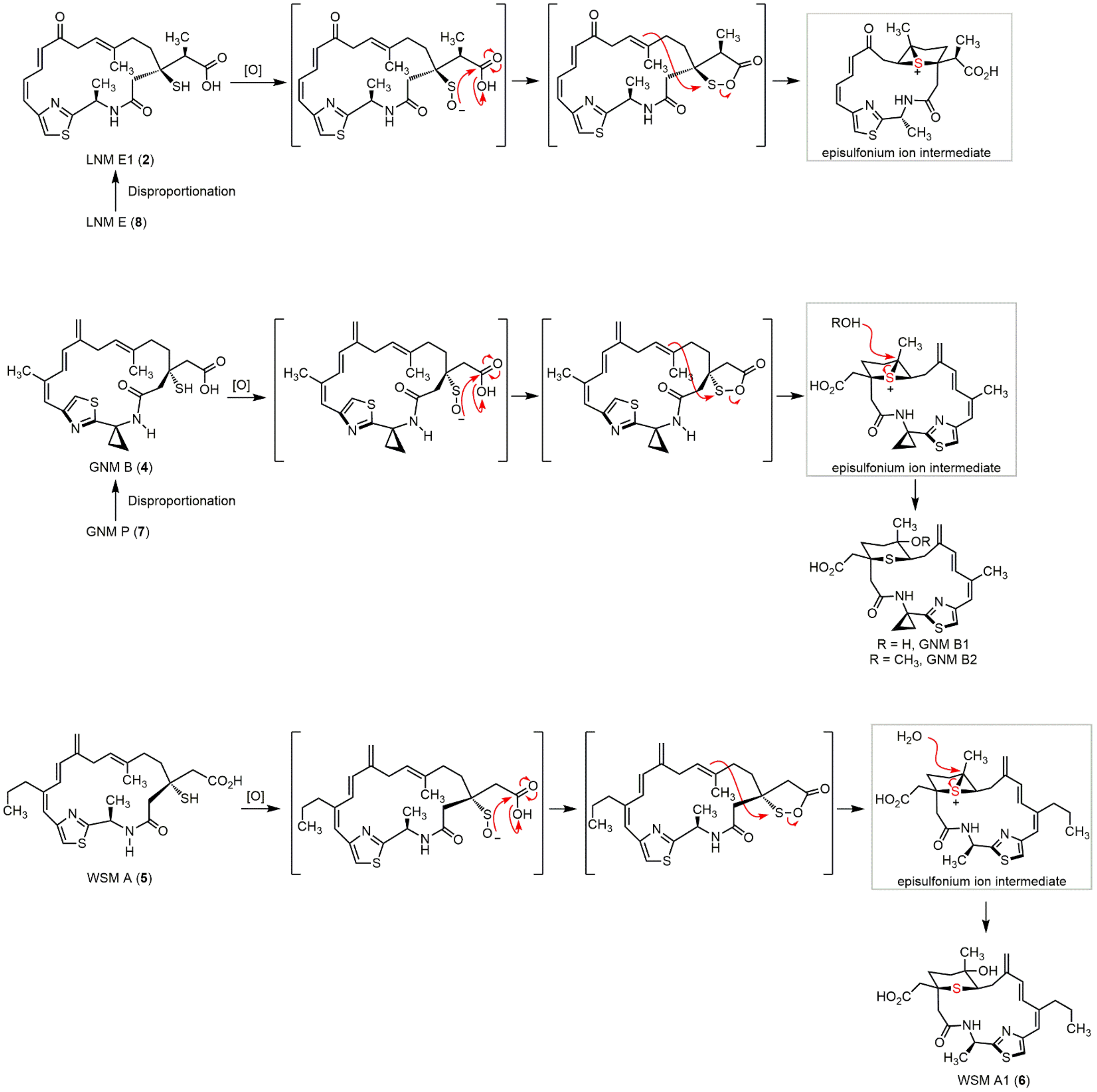

Supplementary Figure 3. Hydropersulfides, as exemplified by LNM E (**8**) and GNM P (**7**), as common intermediates that undergo disproportionation to yield thiol congeners LNM E1 (**2**) and GNM B (**4**). These thiols and WSM A (**5**) undergo oxidative activation and epi-sulfonium ion-mediated cyclization to form congeners isolated in previous studies.[7,8]

a

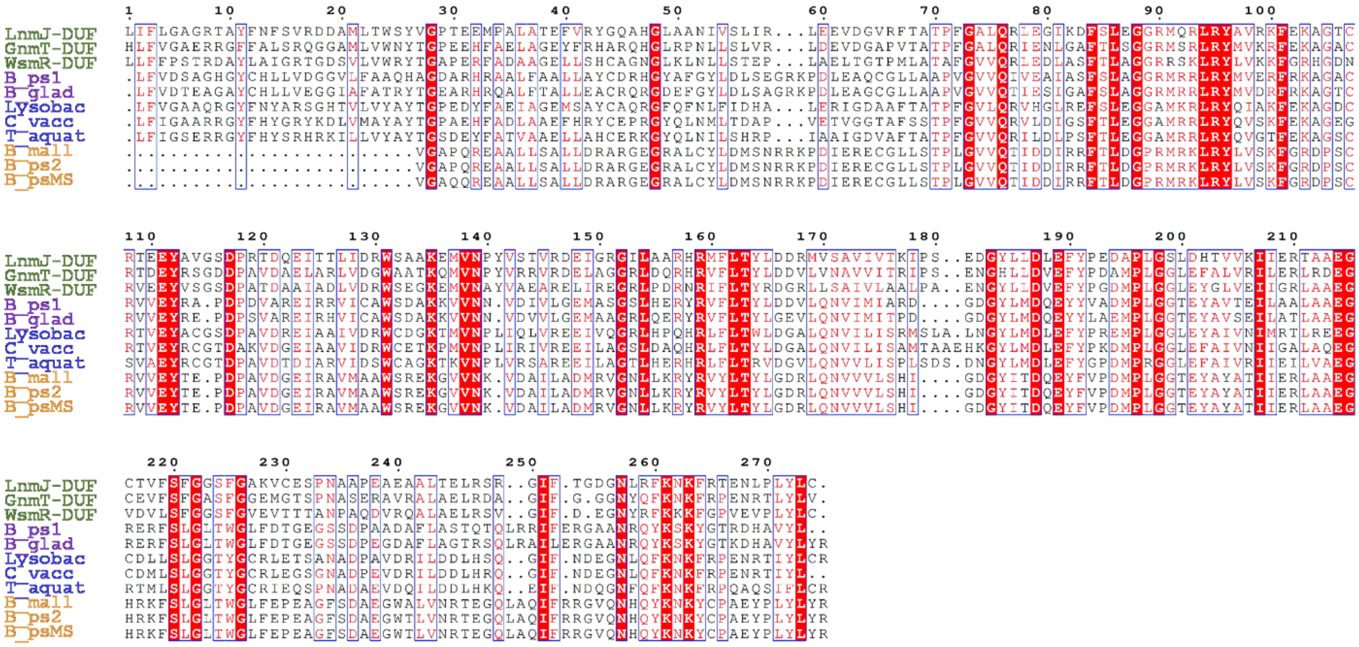
LnmJ-DUF
GnmT-DUF
WsmR-DUF
B_ps1
B_glad
Lysobac
C_vacc
T_aquat
B_mall
B_ps2
B_psMS

b

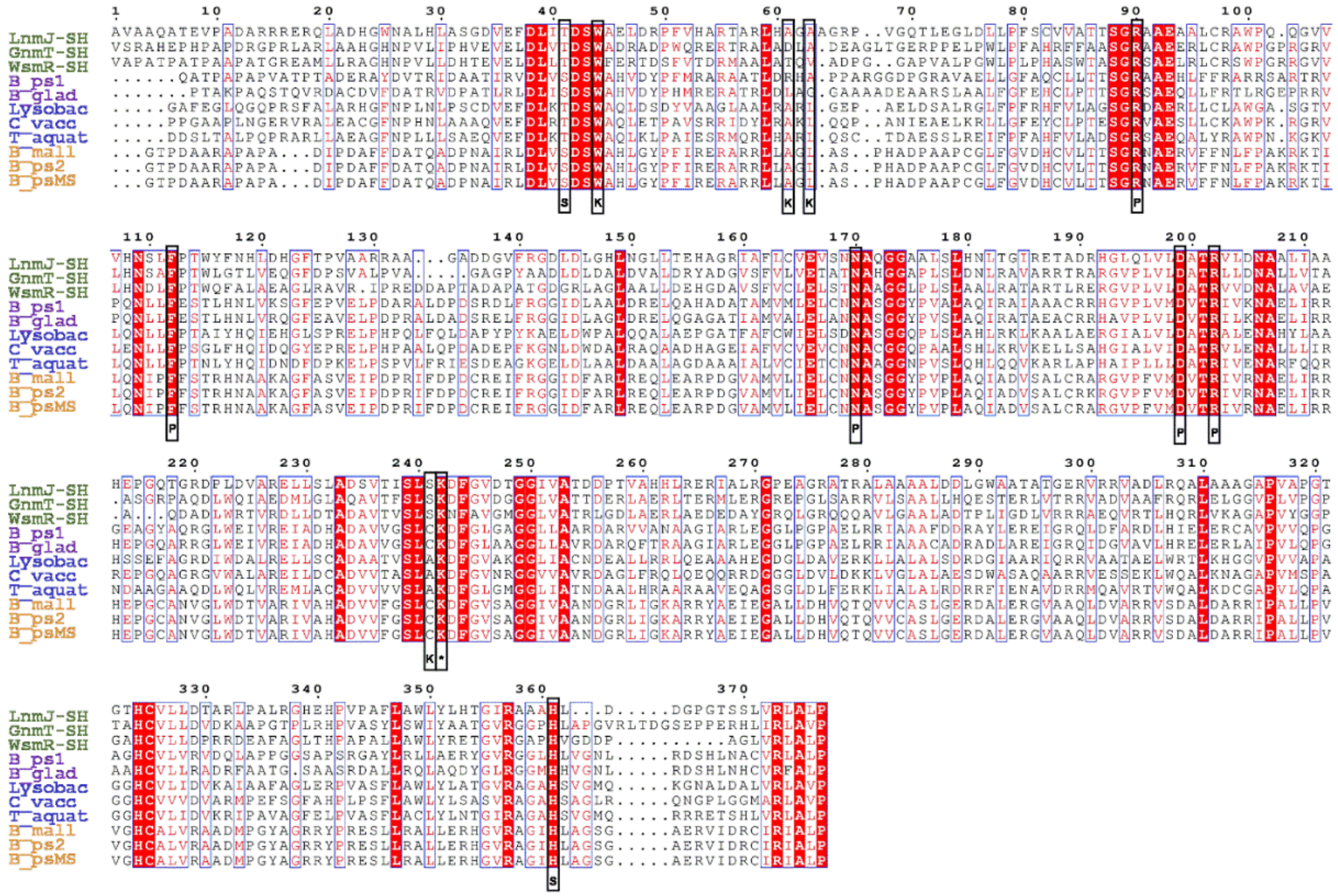

Supplementary Figure 4. DUF-SH didomains from LNM-type and non-LNM type BGCs catalyze similar chemistry.[6,8] Sequence alignment of **a**, DUF domains and **b**, SH domains with representative sequences from the SSN (Fig. 1C in the main text and Supplementary Fig. 5). The DUF domains range from 30 – 57% identity and 53 – 72% similarity with respect to LnmJ-DUF, while the SH domains range from 28 – 43% identity and 42 – 52% similarity with respect to LnmJ-SH. "S" denotes residues predicted to be involved in substrate binding, "K" indicates the predicted potassium-binding residues, "P" indicates the predicted PLP-binding residues, and * denotes the active site lysine residue. Selected proteins or domains for the alignments are (organisms, accession number): B_ps1 (*Burkholderia_pseudomallei*, ABA53217), B_glad (Burkholderia_gladioli, PEH41359), Lysobac (*Lysobacter* sp. ATCC 53042, AEH59109), C_vacc (*Chromobacterium vaccini*, AVG17432), T_aquat (*Tahibacter aquaticus*, TDR44821), B_mall (*Burkholderia_mallei*, RPA48615), B_ps2 (*Burkholderia pseudomallei*, ARK90304), B_psMS *(Burkholderia pseudomallei* MSHR346, EEP52025), Coral (*Corallococcus llansteffanensis*, RKH68632), B_SJZ (*Burkholderia* sp. SJZ115, TWD06333), Alter (*Alteromonadaceae bacterium*, PCK08398), B_glad2 (*Burkholderia gladioli*, PEH41361).

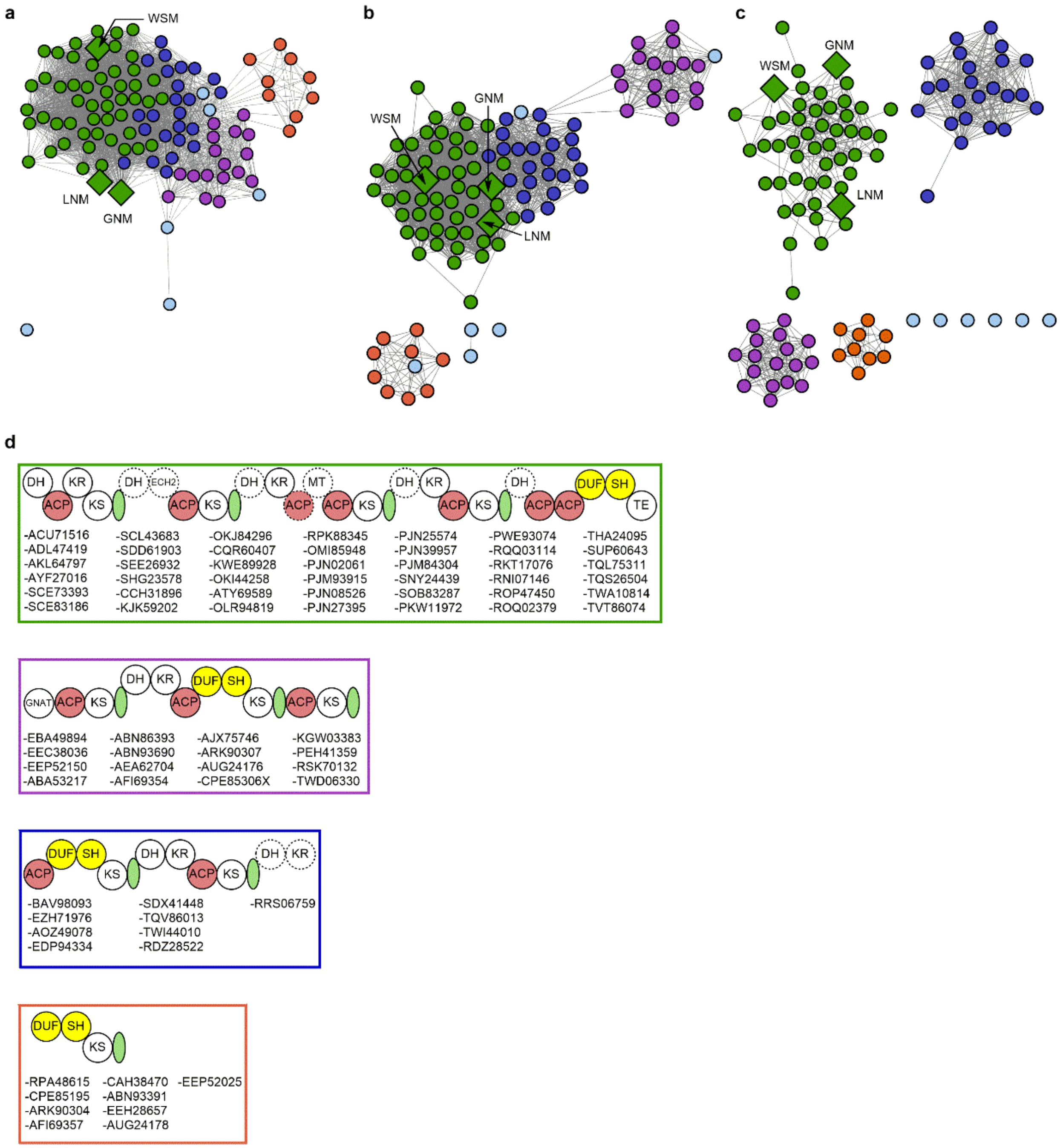

Supplementary Figure 5. Bioinformatic analysis of DUF-SH-containing PKS proteins demonstrating the programmability of the DUF-SH didomain in the biosynthesis of diverse polyketide scaffolds.[6,8] Sequence similarity network of DUF-SH-containing PKS proteins using LnmJ-DUF-SH as the query sequence, with an alignment score cutoff of **a**, 150, **b**, 210, and **c**, 410, respectively. The DUF-SH didomains from the LNM-type BGCs are color-coded in green, with the corresponding PKSs from the LNM, GNM, and WSM BGCs depicted as diamonds. **d**, Domain and modular architectures of representative PKSs from each of the four major clusters of the SSNs depicted in **a**, **b**, and **c**. NCBI accession numbers are given for each of the representative PKSs, and dotted circles denote domains that are not present in all members of the predicted PKSs within each of the four major clusters. ACP, acyl carrier protein; DH, dehydratase; DUF, domain of unknown function; ECH2, enoyl-CoA hydratase homologue 2; GNAT, GCN5-related *N*-acetyltransferase; KR, ketoreductase; KS, ketosynthase; MT, methyltransferase; SH, thiocysteine lyase; TE, thioesterase; and the green ovals denoting an acyltransferase docking domain.

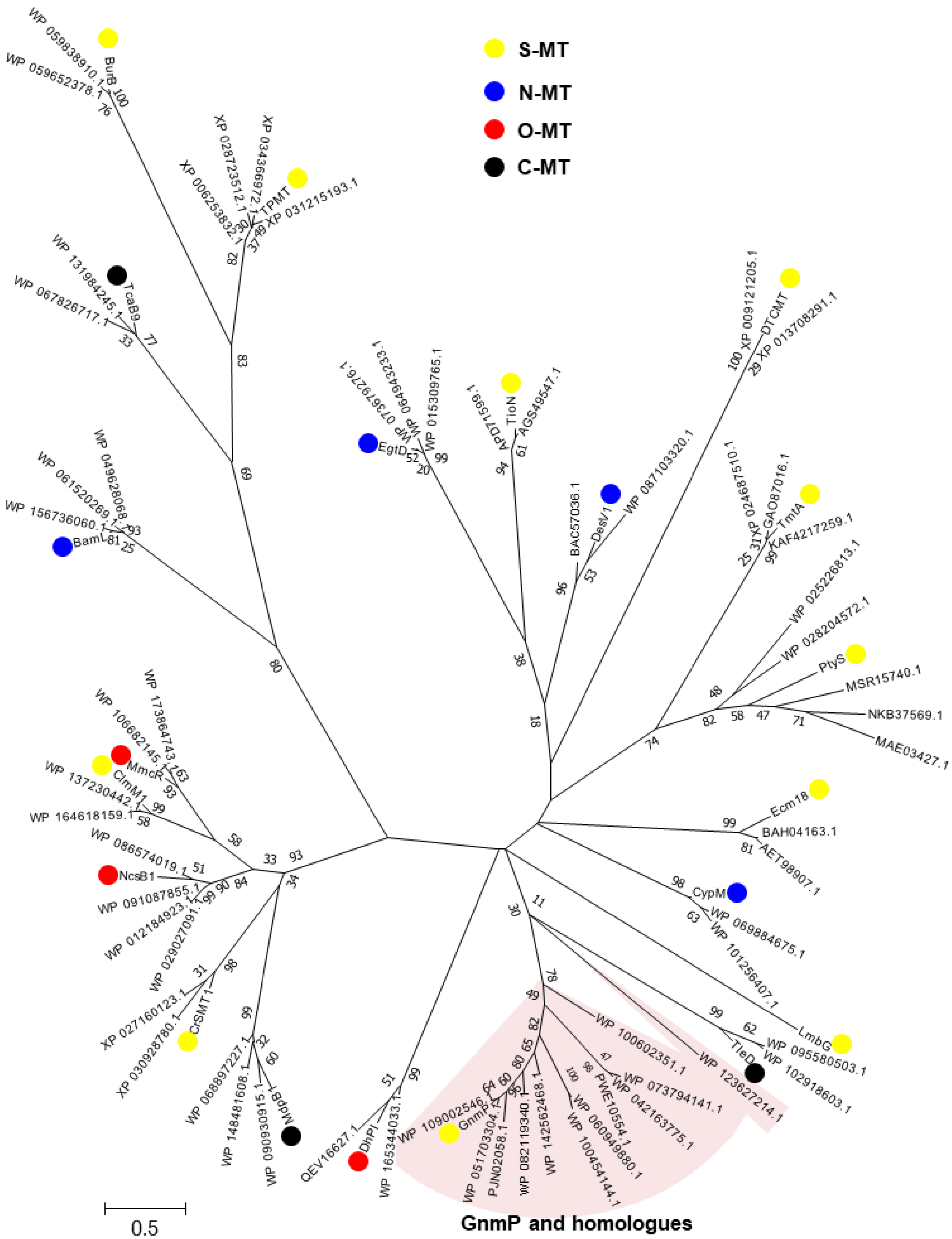

Supplementary Figure 6. Phylogenetic analysis of GnmP showing the inability to predict GnmP as a *C*-, *O*-, *N*- or *S*-MT. Phylogenetic tree of GnmP, its homologues from LNM-type BGCs, and functionally characterized *C*-, *O*-, *N*-, and *S*-MTs with their homologues from public database. Sequences were aligned with the ClustalW algorithm, and the phylogenetic tree was generated by MEGA7 using the maximum likelihood method with a bootstrap test of 500 replicates. See Supplementary Table 9 for accession numbers for the functionally characterized *C*-, *O*-, *N*-, and *S*-MTs used in the analysis.

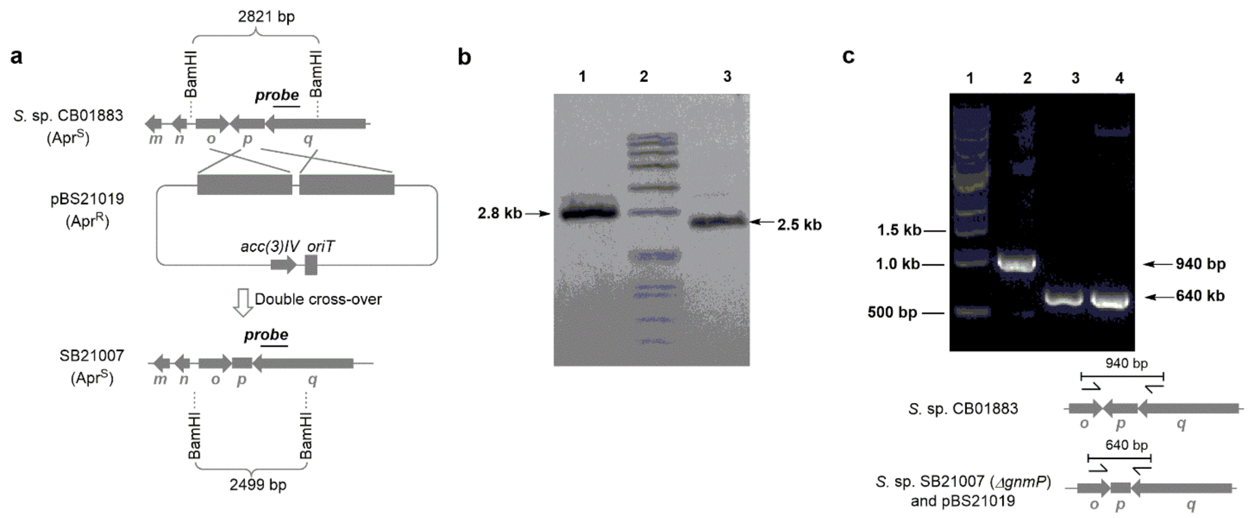

Supplementary Figure 7. Construction and confirmation of the Δ*gnmP* mutant strain *S.* sp. SB21007. **a**, Schematic representation for the deletion of *gnmP* in *S.* sp. CB01883 by homologous recombination. Apr^{S}, apramycin sentitive; Apr^{R}, apramycin resistance; *acc(3)IV*, apramycin resistance gene. **b**, Southern blot verification of CB01883 wild-type (2.8 kb) and SB21005 (2.5 kb). Genomic DNAs were digested with BamHI and then used for Southern analysis. Lane 1, CB01883 wild-type; lane 2, DNA marker VII, DIG-labeled (Roche); lane 3, SB21007. The Southern blot was performed once. **c**, PCR verification of SB21007. Lane 1, DNA marker; lane 2 PCR product (940 bp, CB01883 genomic DNA as the template); lane 3 PCR product (640 bp, SB21007 genomic DNA as the template); lane 4 PCR product (640 bp, pBS21019 as the template). The PCR reaction and gel electrophoresis experiment was performed once.

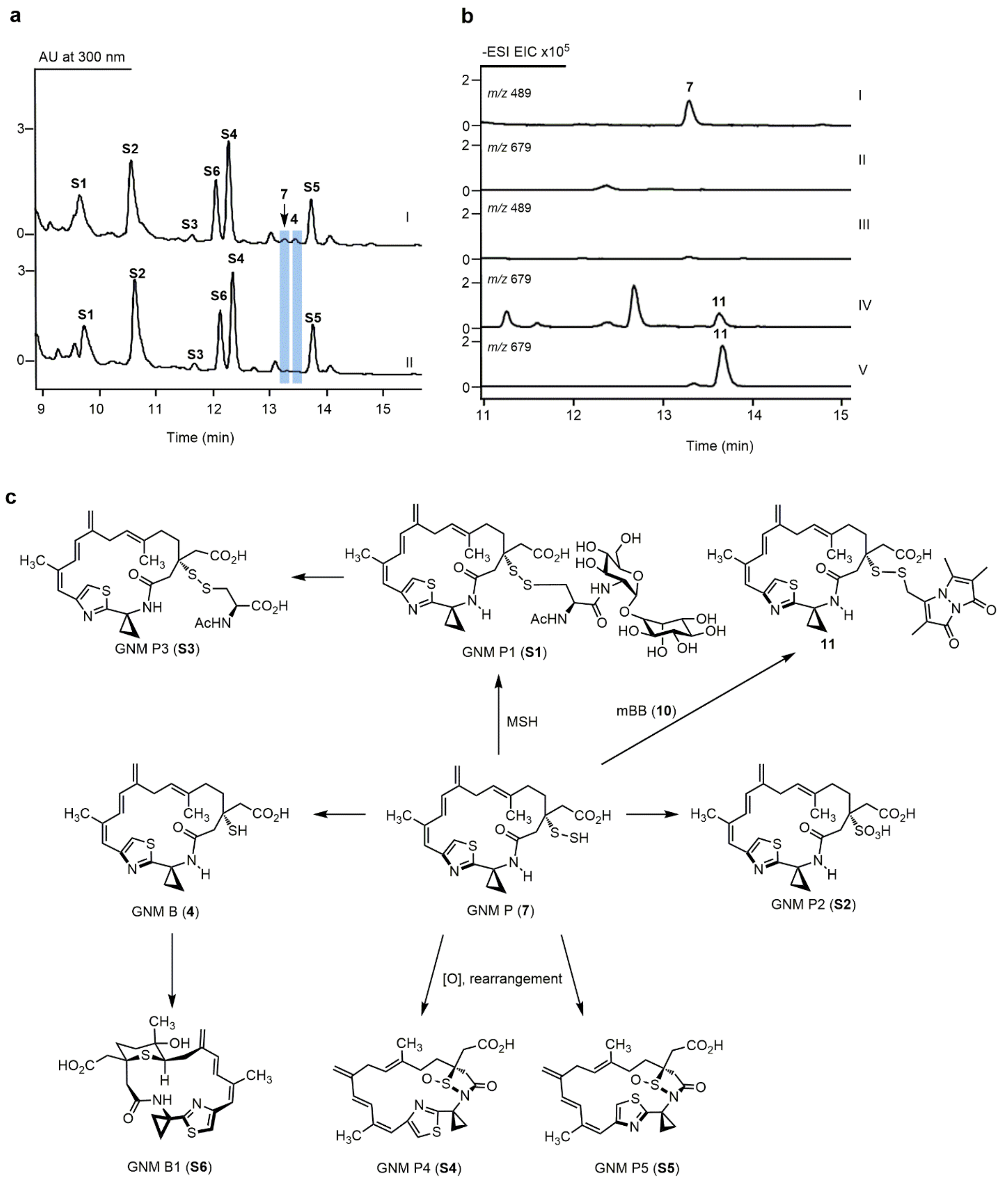

Supplementary Figure 8. Establishment of GNM P (**7**) as the nascent product accumulated by the Δ*gnmP* mutant strain SB21007 and isolation and characterization of its degradation products. **a**, HPLC analysis of metabolite profile of (I) *ΔgnmP* strain SB21007, (II) *ΔgnmP* strain SB21007 fermentation supplemented with mBB. **b**, EIC chromatograms (*m/z* 489 = **7** and *m/z* 679 = **10**) of the fermentation of *ΔgnmP* SB21007, the same fermentation supplemented with mBB, and standards. (I) *m/z* = 489 from *ΔgnmP*, (II) *m/z* = 679 from *ΔgnmP*, (III) *m/z* = 489 from *ΔgnmP* with mBB, (IV) *m/z* = 679 from *ΔgnmP* with mBB, (V) *m/z* = 679 from **10** standard. **c**, Characterization of the major metabolites, including GNM B (**4**), as degradation products of GNM P (**7**) due to its intrinsic instability based on high-resolution mass spectrometry (HRMS) and 1D and 2D NMR analysis (Supplementary Figs. 71 – 110 and Supplementary Tables 7 and 8).

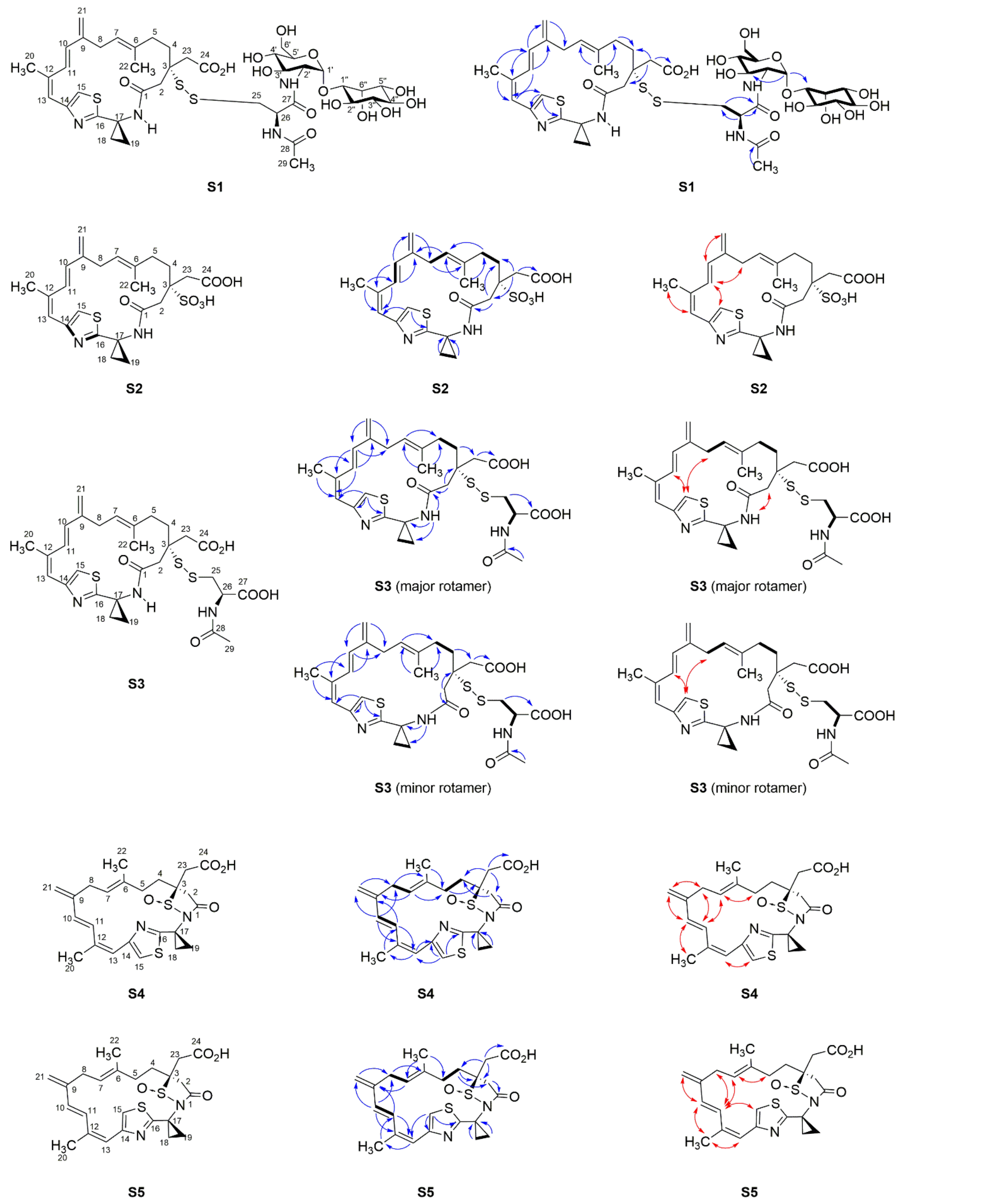

Supplementary Figure 9. Elucidation of new metabolites **S1**, **S2**, **S3**, **S4**, and **S5** isolated from the *ΔgnmP* strain SB21007. Key ^{1}H-^{1}H COSY (bold), HMBC (blue arrows), and ROESY correlations (red arrows) are shown. NMR and HRMS data are shown in Supplementary Figures 71 – 110 and Supplementary Tables 7 and 8.

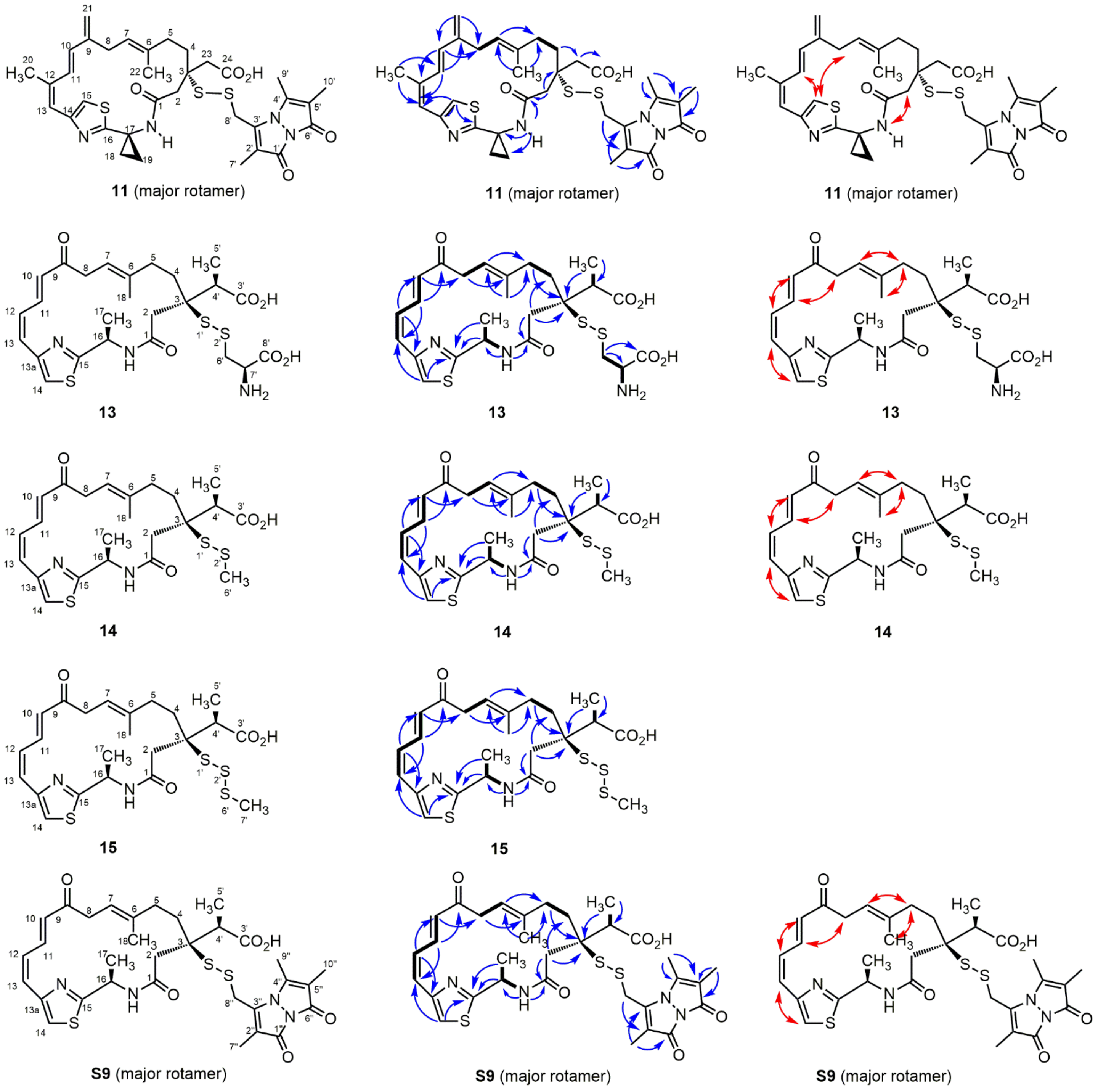

Supplementary Figure 10. Structural confirmation of the GNM P-mBB adduct **11**, L-cysteinyl-LNM E1 **13,** the methylated products of LNM E (**14**), its disproportionation trisulfide (**15**), and LNM E-mBB adduct **S9**. Key ^{1}H-^{1}H COSY (bold), HMBC (blue arrows), and ROESY correlations (red arrows) are shown. NMR and HRMS data are shown in Supplementary Figures 34 – 61 and 119 – 126 and Supplementary Tables 5 and 6.

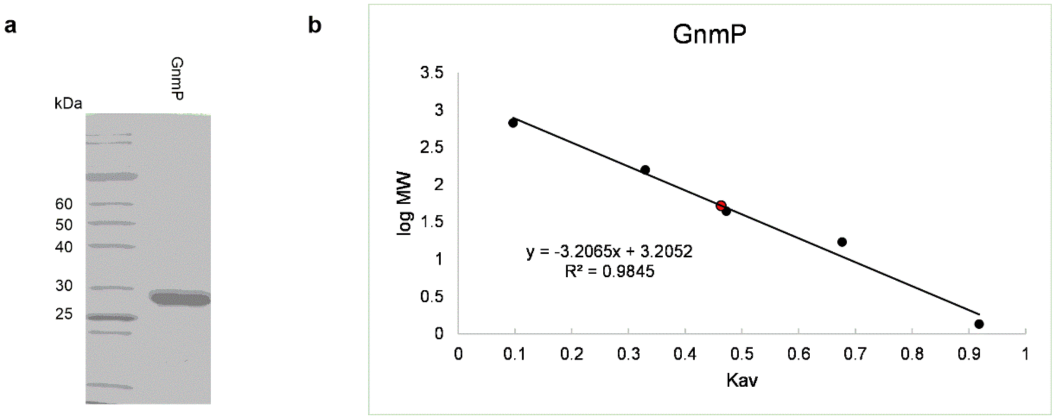

Supplementary Figure 11. Overproduction in *E. coli* and purification of GnmP. **a**, SDS-PAGE analysis of purified GnmP. **b**, Size-exclusion chromatography of the purified GnmP protein. GnmP eluted at retention volume of 79.3 mL, which was converted to K_{av} according to the equation $K_{av} = (V_e – V_o) / (V_t – V_o)$, where V_e, V_o, and V_t is the elution volume, column void volume, and total bed volume, respectively. The equation provided a K_{av} value of 0.46, which was plotted on the calibration curve (red circle) and provided a molecular weight (MW) of 52.3 kDa, with an expected MW of 29.7 kDa. Thus, GnmP is supported as a homodimer in solution. The data is representative of two independent enzyme preparations.

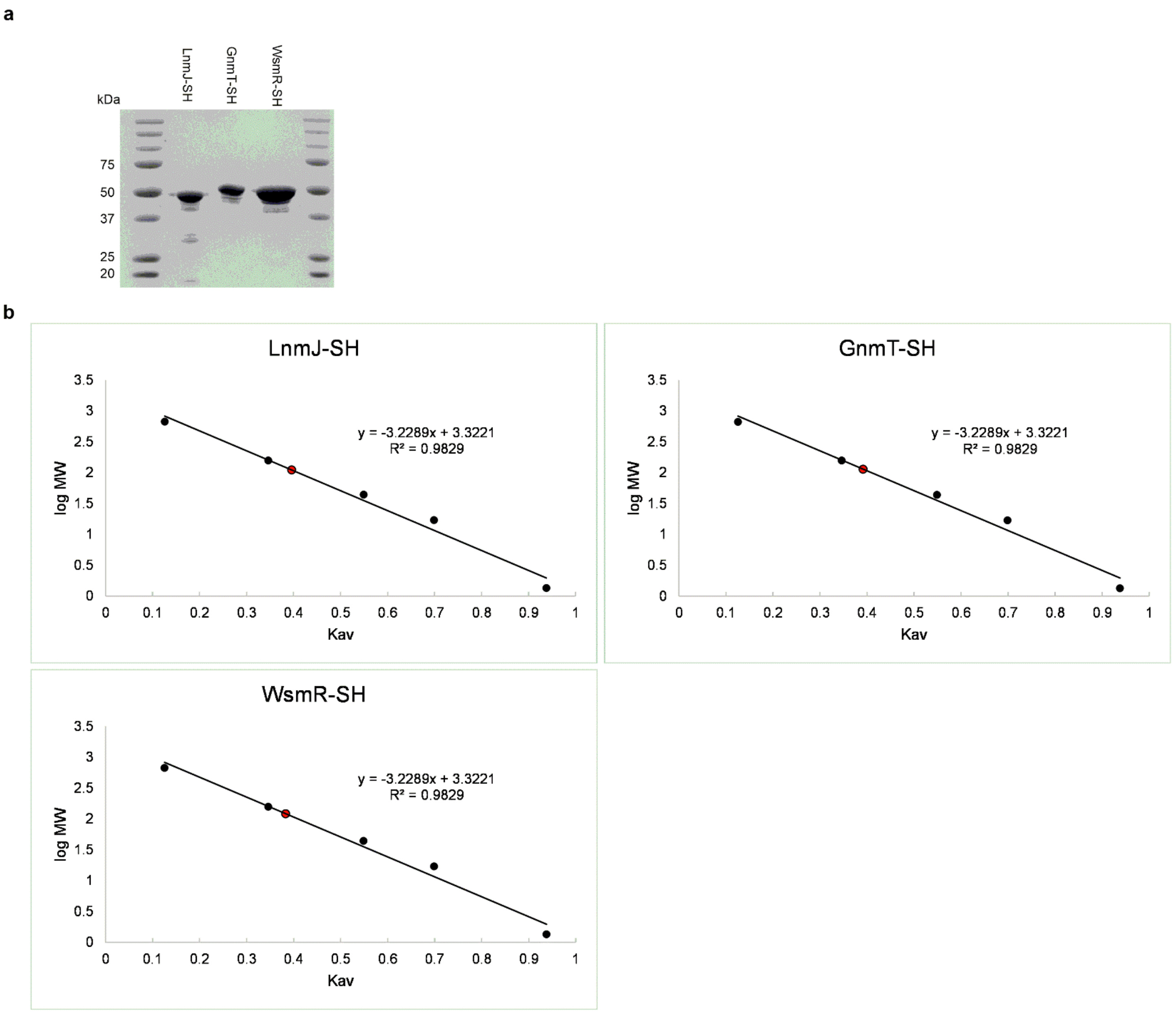

Supplementary Figure 12. Overproduction in *E. coli* and purification of the SH domains utilized in this study.[6] **a**, SDS-PAGE analysis of purified LnmJ-SH, GnmT-SH, and WsmR-SH. **b**, Size-exclusion chromatography of the purified SH domains. LnmJ-SH, GnmT-SH, and WsmR-SH eluted at retention volumes of 74.1, 73.8, and 73.2 mL, respectively, which were each converted to K_{av} according to the equation $K_{av} = (V_e - V_o) / (V_t - V_o)$, where V_e, V_o, and V_t is the elution volume, column void volume, and total bed volume, respectively. The equation provided K_{av} values of 0.40, 0.39, and 0.38 for LnmJ-SH, GnmT-SH, and WsmR-SH, respectively, which were individually plotted on the calibration curve (red circles) and provided MWs of 110, 114, and 121 kDa. The expected MW of LnmJ-SH, GnmT-SH, and WsmR-SH were 50, 52, and 49 kDa, respectively. Thus, the SH domains are supported as homodimers in solution. The data is representative of two independent enzyme preparations.

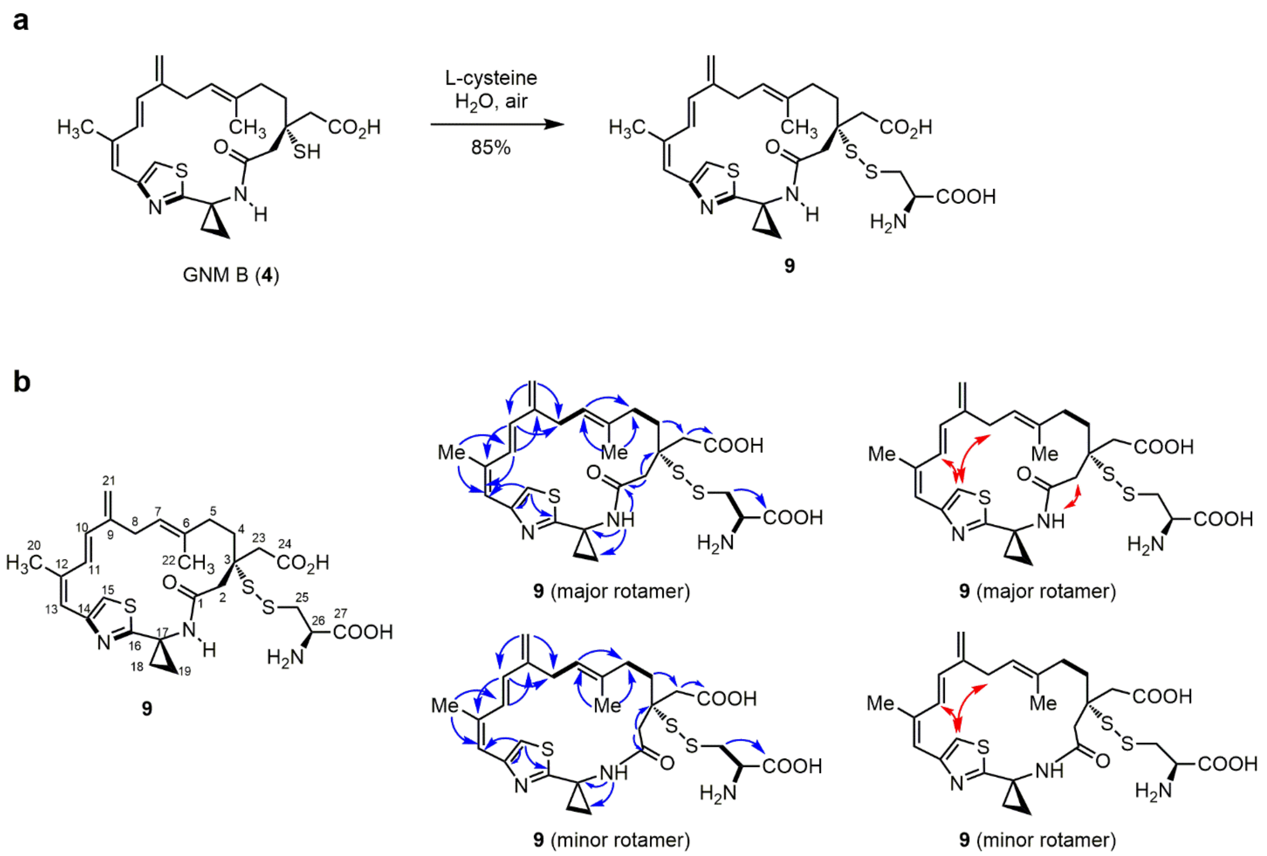

Supplementary Figure 13. Synthesis and structural characterization of **9**. **a**, Synthesis of **9** from GNM B (**4**). **b**, Structural confirmation of L-cysteinyl-GNM B adduct **9**. Key ^{1}H-^{1}H COSY (bold), HMBC (blue arrows) and ROESY (red arrows) correlations are shown. HRMS and NMR data are shown in Supplementary Figures 26 – 33 and Supplementary Table 4.

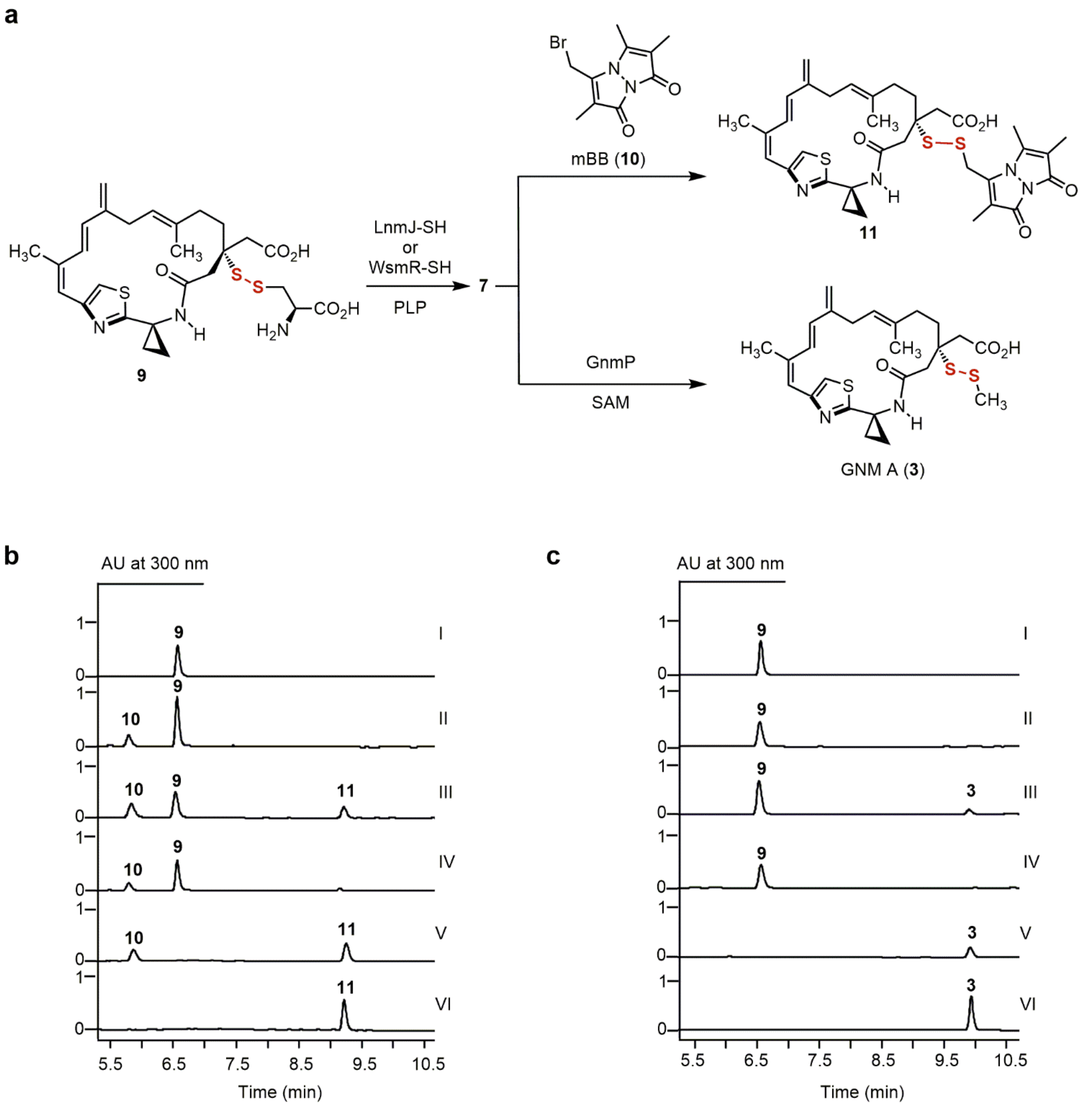

Supplementary Figure 14. Generation of the persulfide biosynthetic intermediate GNM P (**7**) using LnmJ-SH or WsmR-SH with substrate **9** and its trapping with mBB (**10**) or methylation with GnmP. **a**, Reaction scheme of the generation of **7** from **9** and trapping with mBB or methylation with GnmP to form the mBB-**7** adduct (**11**) or GNM A (**3**), respectively. **b**, HPLC analysis of the generation of **7** from **9** by LnmJ-SH or WsmR-SH and trapping with mBB (**10**) to form **11**. Each reaction was carried out in the presence of 0.2 mM PLP and 20 mM KCl. The structure of **11** was established by a combination of HRMS, 1D, and 2D NMR analysis (See Supplementary Figures 34 – 39 and Supplementary Table 5). (I) substrate **9**, (II) **9** + LnmJ-SH (boiled) + mBB, (III) **9** + LnmJ-SH + mBB, (IV) **9** + WsmR-SH (boiled) + mBB, (V) **9** + WsmR-SH + mBB, (VI) product **11**. **c**, HPLC analysis of the generation of **7** from **9** by LnmJ-SH or WsmR-SH and methylation by GnmP to form **3**. Each reaction was carried out in the presence of 0.2 mM PLP and 20 mM KCl. (I) substrate **9**, (II) **9** + LnmJ-SH (boiled) + GnmP (boiled) + SAM, (III) **9** + LnmJ-SH + GnmP + SAM, (IV) **9** + WsmR-SH (boiled) + GnmP (boiled) + SAM, (V) **9** + WsmR-SH + GnmP + SAM, (VI) Product **3** standard.

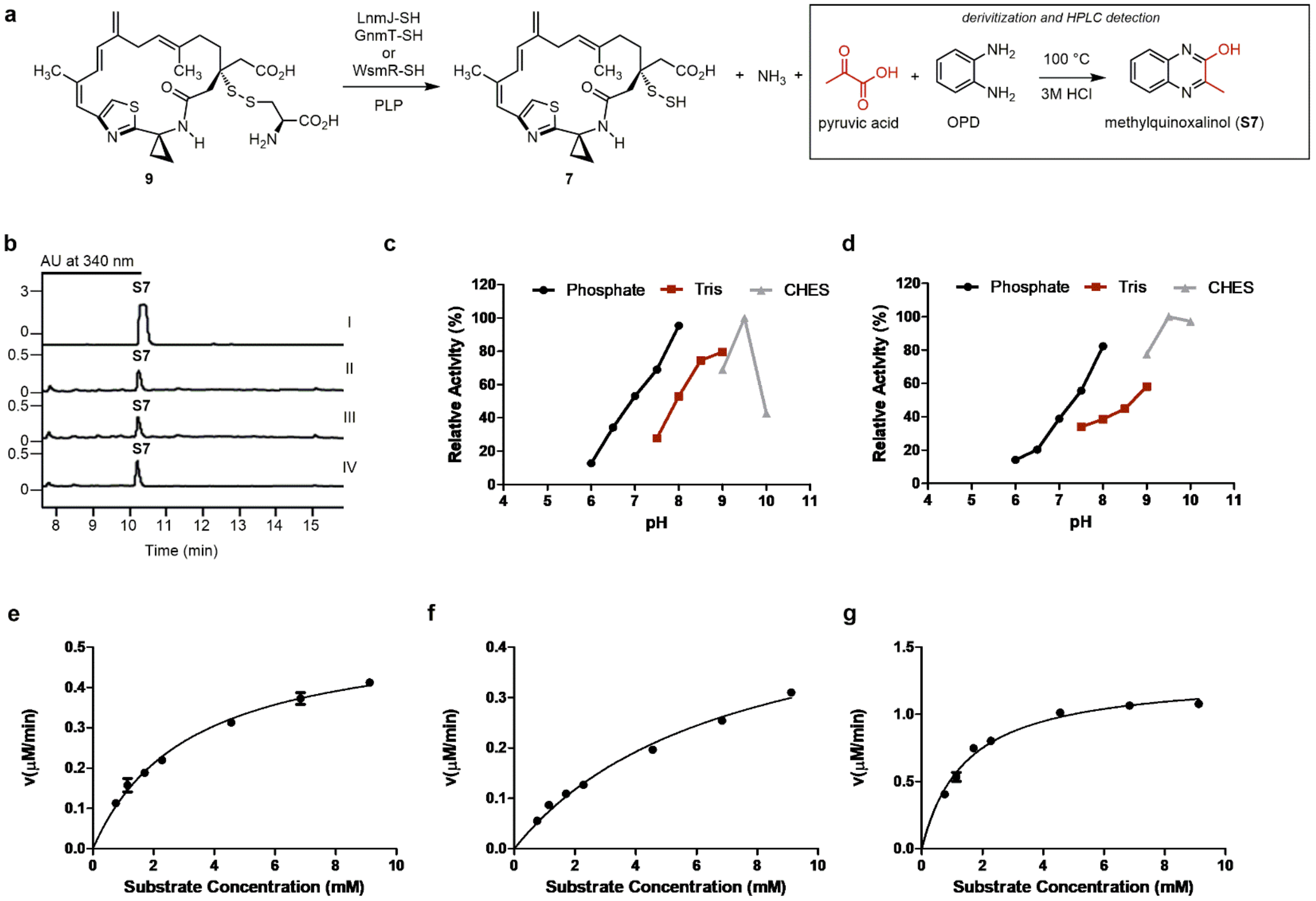

Supplementary Figure 15. Steady-state analysis of GnmT-SH, LnmJ-SH, and WsmR-SH with **9** as the substrate. **a**, SH-catalyzed β-elimination with concomitant pyruvate formation and trapping in situ with *o*-phenylenediamine (OPD) to form methylquinoxalinol (**S7**). **b**, HPLC analysis of pyruvate formation from GnmT-SH, LnmJ-SH, and WsmR-SH with **9**, upon derivatization with OPD. (I) methylquinoxalinol (**S7**) standard from the reaction of pyruvate with OPD, (II) **9** with GnmT-SH, (III) **9** with LnmJ-SH, (IV) **9** with WsmR-SH. pH optimization of **c**, GnmT-SH with **9** as a substrate, with n=2 independent experiments and **d**, WsmR-SH with **9** as a substrate, with n=2 independent experiments. Michaelis-Menten plots of **e**, GnmT-SH at 500 nM. **f**, LnmJ-SH at 500 nM, and **g**, WsmR-SH at 500 nM, with varying concentration of **9** (from 0.76 – 9.12 mM), affording GnmT-SH at K_m = 3.2 ± 0.3 mM and k_{cat} = 1.09 ± 0.04 min^{-1}, LnmJ-SH at K_m = 7.04 ± 0.84 mM and k_{cat} = 1.06 ± 0.07 min^{-1}, and WsmR-SH at K_m = 1.45 ± 0.12 mM and k_{cat} = 2.58 ± 0.07 min^{-1}, with each kinetic dataset from n=3 independent experiments. Data are presented as mean values ± S.E.M., with error bars representing the S.E.M. values.

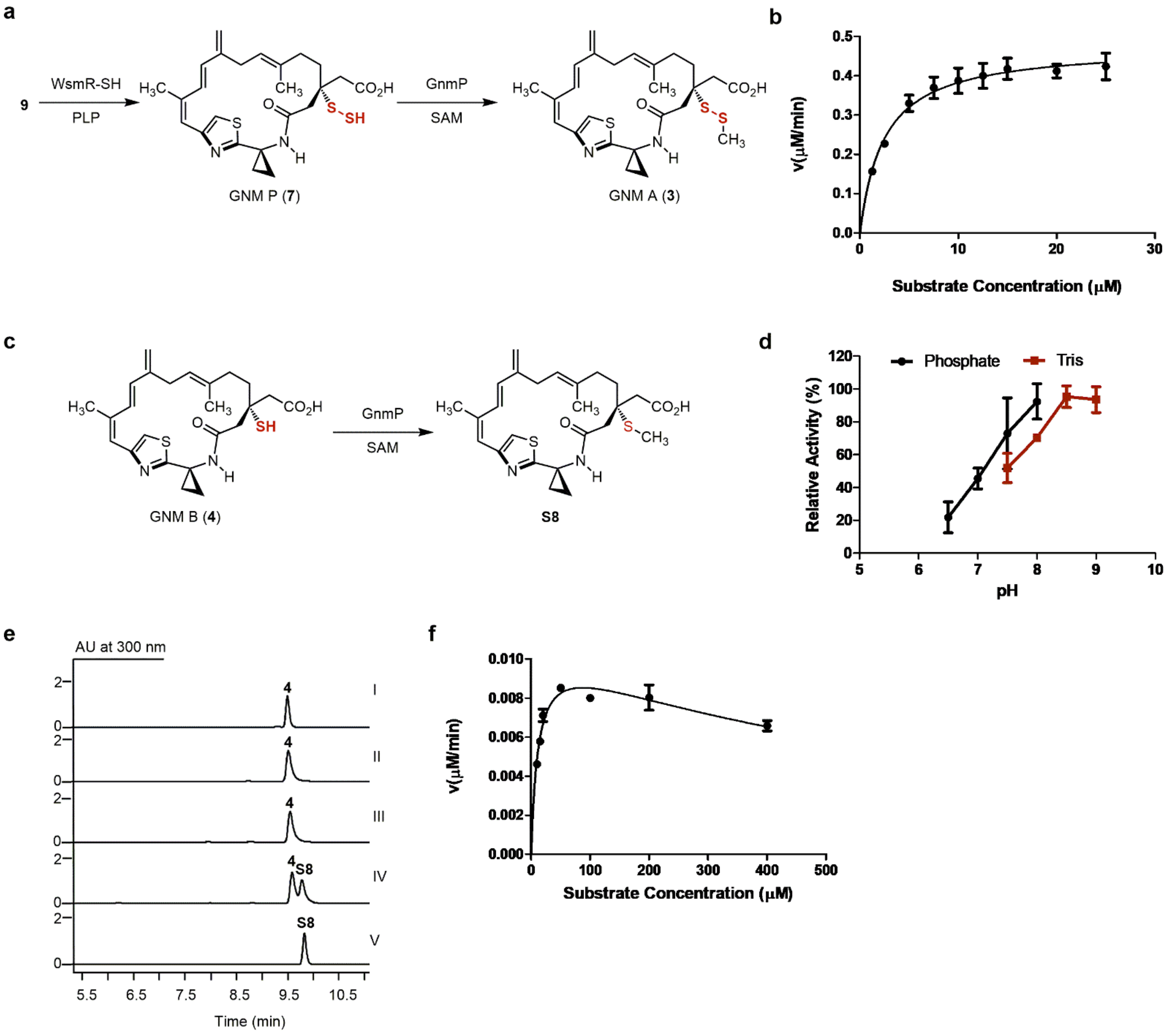

Supplementary Figure 16. Steady-state analysis of GnmP with the L-cysteinyl-GNM B adduct (**9**) as a surrogate substrate of GNM P (**7**) and GNM B (**4**) as an alternative substrate, demonstrating that GnmP prefers **7** over **4** as a substrate. **a**, Reaction of GnmP with **7**, generated from **9** and WsmR-SH in situ, and subsequent SAM-dependent methylation of **7** catalyzed by GnmP. **b**, Michaelis-Menten plot of a WsmR-SH and GnmP coupled assay, using an excess of WsmR-SH (220 µM) relative to GnmP (50 nM), at saturating SAM (2 mM) with varying concentration of **9** (from 1.25 – 25 µM), affording a K_m value of 2.45 ± 0.41 µM and a k_{cat} value of 9.53 ± 0.36 min^{-1}, with n=3 independent experiments. **c**, Reaction of **4** with GnmP to form GNM analog **S8**. **d**, pH optimization of the reaction of GnmP with GNM B (**4**) as a substrate, with n=3 independent experiments. **e**, HPLC chromatograms of GnmP with **4** as the substrate to form **S8**. (I) substrate **4**, (II) **4** with GnmP, (III) **4** with GnmP (boiled) and SAM, (IV) **4** with GnmP and SAM, (V) product **S8**. **f**, Michaelis-Menten plots of GnmP (2.5 µM), at saturating SAM (2 mM) with varying concentration of **4** (from 10 – 400 µM), the substrate inhibition equation was fit to the data, affording a K_m value of 12.1 ± 2.2 µM and a k_{cat} value of (4.36 ± 0.29) * 10^{-3} min^{-1}, with n=3 independent experiments. Data are presented as mean values ± S.E.M, with error bars representing the S.E.M values.

a

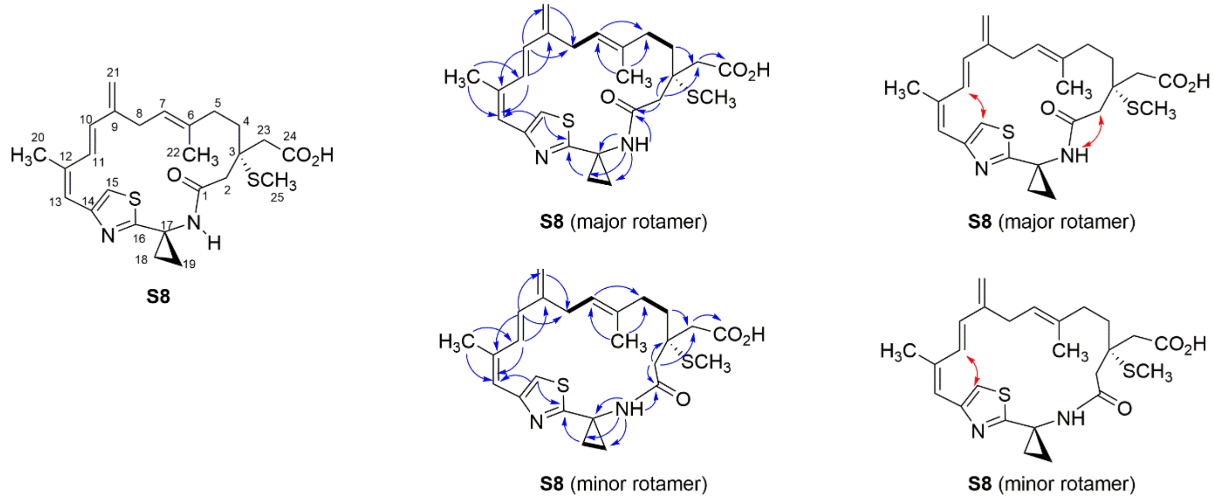

b

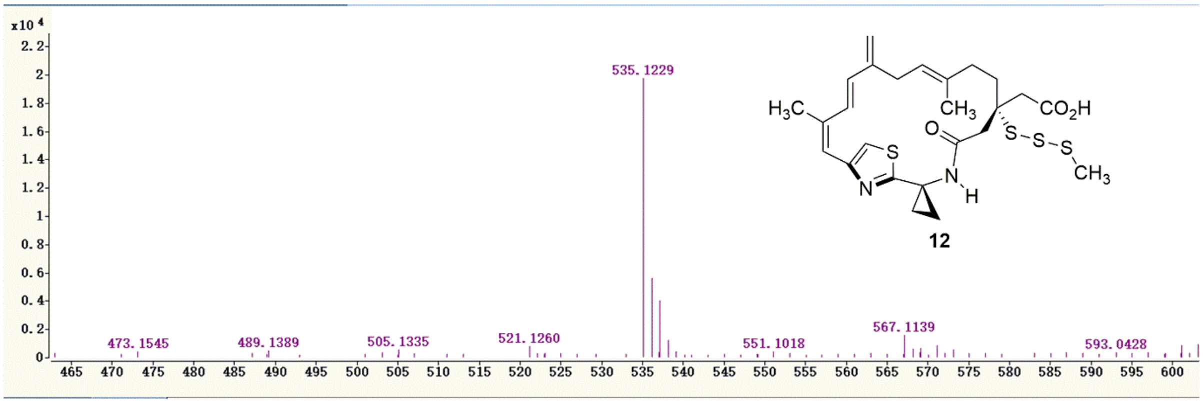

Supplementary Figure 17. Structural confirmation of **S8** and **12**. **a**, Structural confirmation of **S8**, key ^{1}H-^{1}H COSY (bold), HMBC (blue arrows) and ROESY (red arrows) correlations are shown. HRMS and NMR data are shown in Supplementary Figures 111 – 118 and Supplementary Table 9. **b**, HRMS (ESI) spectrum and structure of **12**.

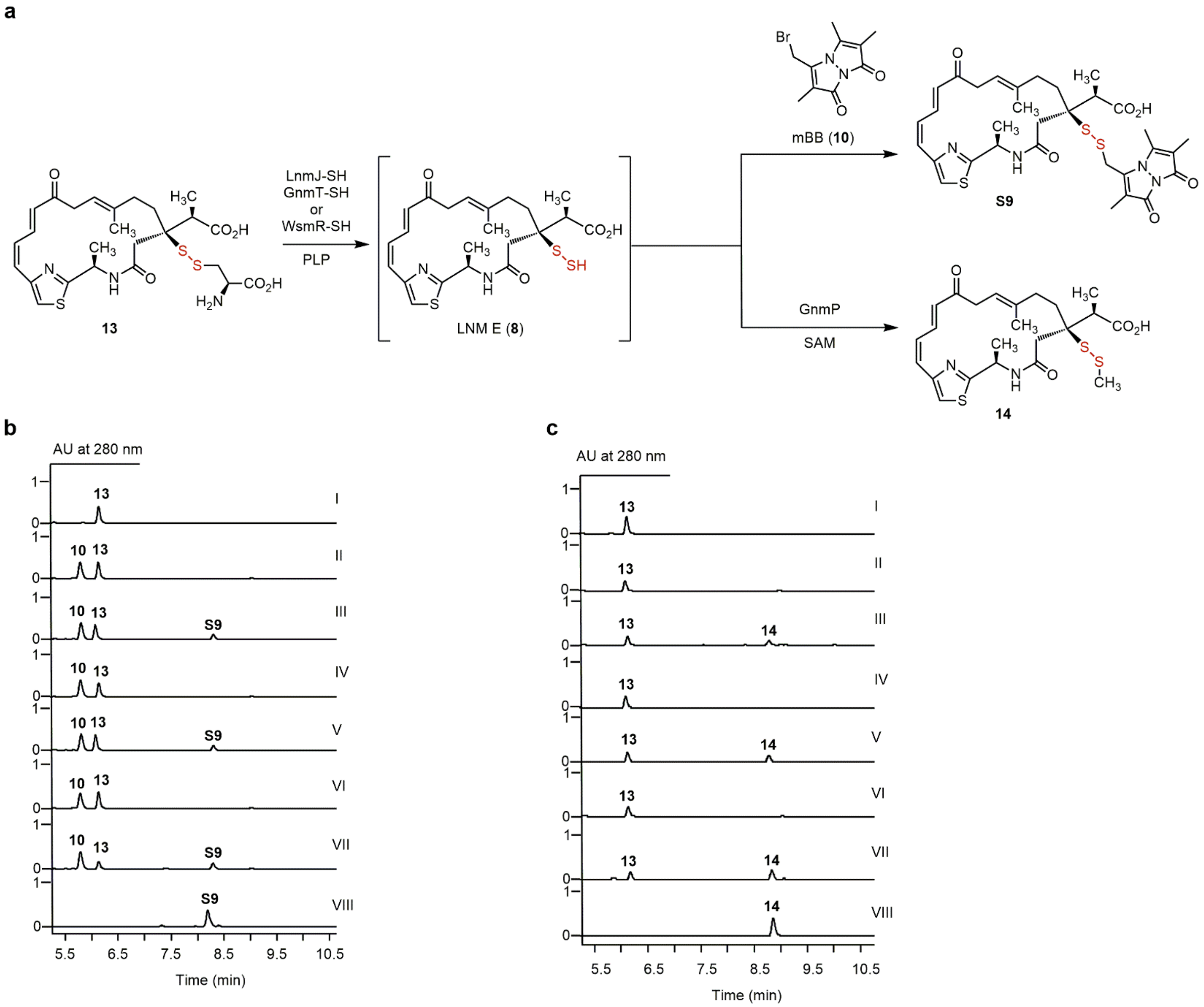

Supplementary Figure 18. Enzymatic reactions of GnmT-SH, LnmJ-SH, and WsmR-SH and GnmP, showing substrate promiscuity of both thiocysteine lyase and persulfide methyltransferase. **a**, Chemoenzymatic synthesis of LNM E (**8**) from the L-cysteinyl-LNM E1 adduct **13**, which was trapped with mBB to form **S9**, and SH-GnmP coupled assay of GnmP-catalyzed *S*-methylation of LNM E to **14** using **13** as a surrogate substrate. **b**, HPLC analysis of the generation of LNM E by GnmT-SH, LnmJ-SH, or WsmR-SH and trapping with mBB to form **S9**. The structure of **S9** was established by a combination of HRMS, 1D, and 2D NMR analysis (Supplementary Figures 119 – 126 and Supplementary Table 5). (I) substrate **13**, (II) **13** + GnmT-SH (boiled) + mBB, (III) **13** + GnmT-SH + mBB, (IV) **13** + LnmJ-SH (boiled) + mBB, (V) **13** + LnmJ-SH + mBB, (VI) **13** + WsmR-SH (boiled) + mBB, (VII) **13** + WsmR-SH + mBB, (VIII) product **S9**. **c**, Generation of LNM E by the GnmT-SH, LnmJ-SH, or WsmR-SH domain and its in situ methylation by GnmP. The structure of **14** was established by a combination of HRMS, 1D, and 2D NMR analysis (Supplementary Figures 48 – 55 and Supplementary Table 6). (I) substrate **13**, (II) **13** + GnmT-SH (boiled) + GnmP (boiled) + SAM, (III) **13** + GnmT-SH + GnmP + SAM, (IV) **13** + LnmJ-SH (boiled) + GnmP (boiled) + SAM, (V) **13** + LnmJ-SH + GnmP + SAM, (VI) **13** + WsmR-SH (boiled) + GnmP (boiled) + SAM, (VII) **13** + WsmR-SH + GnmP + SAM, (VIII) product **14**.

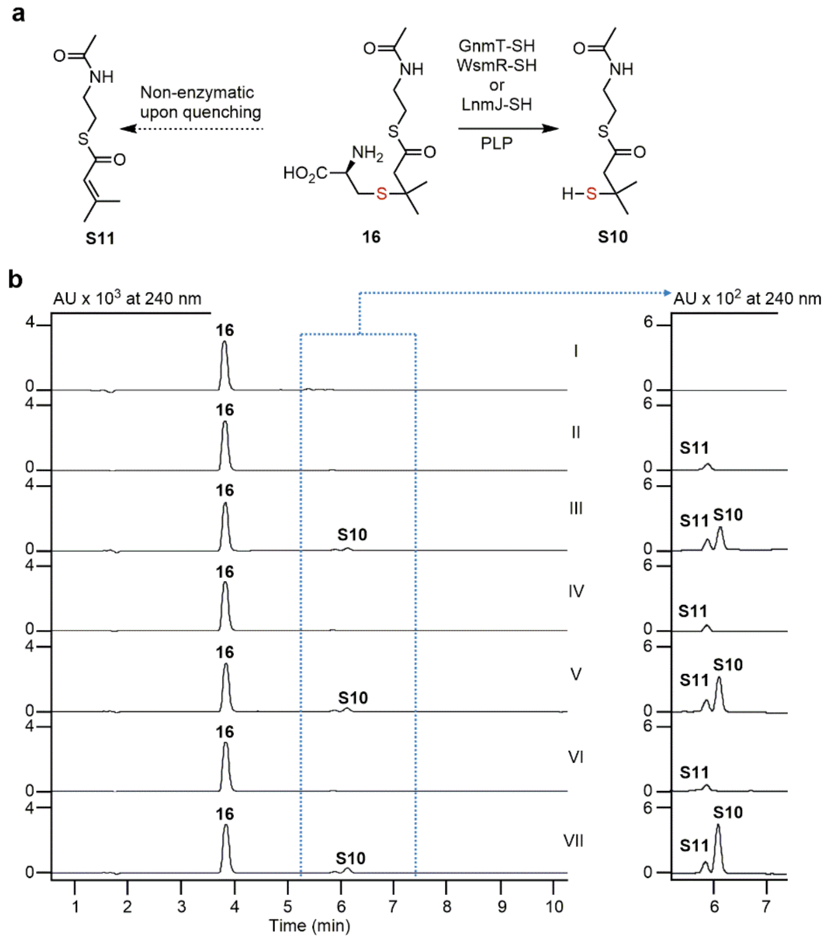

Supplementary Figure 19. Enzymatic reactions of GnmT-SH, LnmJ-SH, and WsmR-SH with **16**.[6] **a**, In vitro assay of GnmT-SH, LnmJ-SH, or WsmR-SH using **16** as a substrate afforded product **S10**. Upon quenching, a trace amount of **S11** can be observed, formed by a retro-Michael reaction. **b**, HPLC analysis of in vitro assays. (I) Substrate **16**, (II) **16** + GnmT-SH (boiled), (III) **16** + GnmT-SH, (IV) **16** + LnmJ-SH (boiled), (V) **16** + LnmJ-SH, (VI) **16** + WsmR-SH (boiled), (VII) **16** + WsmR-SH. Inset on the right shows the magnified (6.67x) chromatograms, where the trace amount of **S11** can be observed.

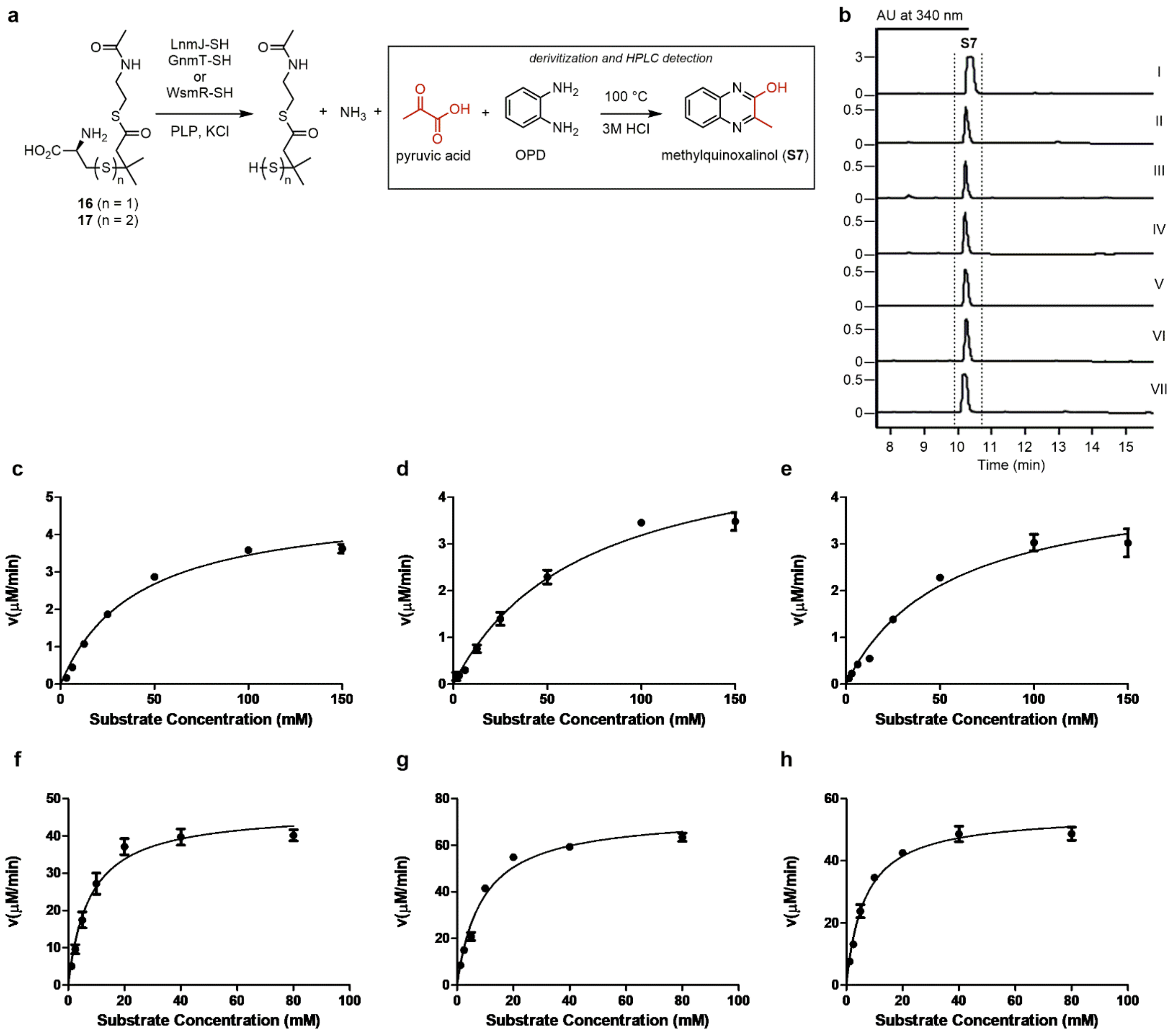

Supplementary Figure 20. Steady-state analysis of GnmT-SH, LnmJ-SH, and WsmR-SH, using the L-thiocysteine-polyketide adduct (**17**) as a substrate mimic in comparison with the L-cysteine-polyketide adduct (**16**), following Michaelis-Menten kinetics.[6] **a**, SH-catalyzed β-elimination with concomitant pyruvate formation and trapping in situ with *o*-phenylenediamine (OPD) to form methylquinoxalinol (**S7**). **b**, HPLC analysis of pyruvate formation from GnmT-SH, LnmJ-SH, and WsmR-SH with **16** and **17** as substrates, upon derivatization with OPD. (I) methylquinoxalinol (**S7**) standard from the reaction of pyruvate with OPD, (II) **16** with GnmT-SH, (III) **16** with LnmJ-SH, (IV) **16** with WsmR-SH, (V) **17** with GnmT-SH, (VI) **17** with LnmJ-SH, (VII) **17** with WsmR-SH. Michaelis-Menten plots of **c**, GnmT-SH at 5 μM, **d**, LnmJ-SH at 5 μM, and **e**, WsmR-SH at 5 μM, with varying concentration of **16** (from 1.56 – 150 mM), and **f**, GnmT-SH at 5 μM, **g**, LnmJ-SH at 5 μM, and **h**, WsmR-SH at 5 μM with varying concentration of **17** (from 1.25 – 80 mM) affording GnmT-SH at K_m = 7.7 ± 1.2 mM and k_{cat} = 9.4 ± 0.4 min^{-1} for **17** vs K_m = 41 ± 5 mM and k_{cat} = 0.97 ± 0.05 min^{-1} for **16**, LnmJ-SH at K_m = 9.0 ± 0.9 mM and k_{cat} = 14.6 ± 0.5 min^{-1} for **17** vs K_m = 69 ± 11 mM and k_{cat} = 1.07 ± 0.08 min^{-1} for **16**, and WsmR-SH at K_m = 6.6 ± 0.7 mM and k_{cat} = 11.0 ± 0.3 min^{-1} for **17** vs K_m = 54 ± 10 mM and k_{cat} = 0.88 ± 0.07 min^{-1} for **16**, respectively. All data were from n=3 independent experiments and are presented as mean values ± S.E.M, with error bars representing the S.E.M values.

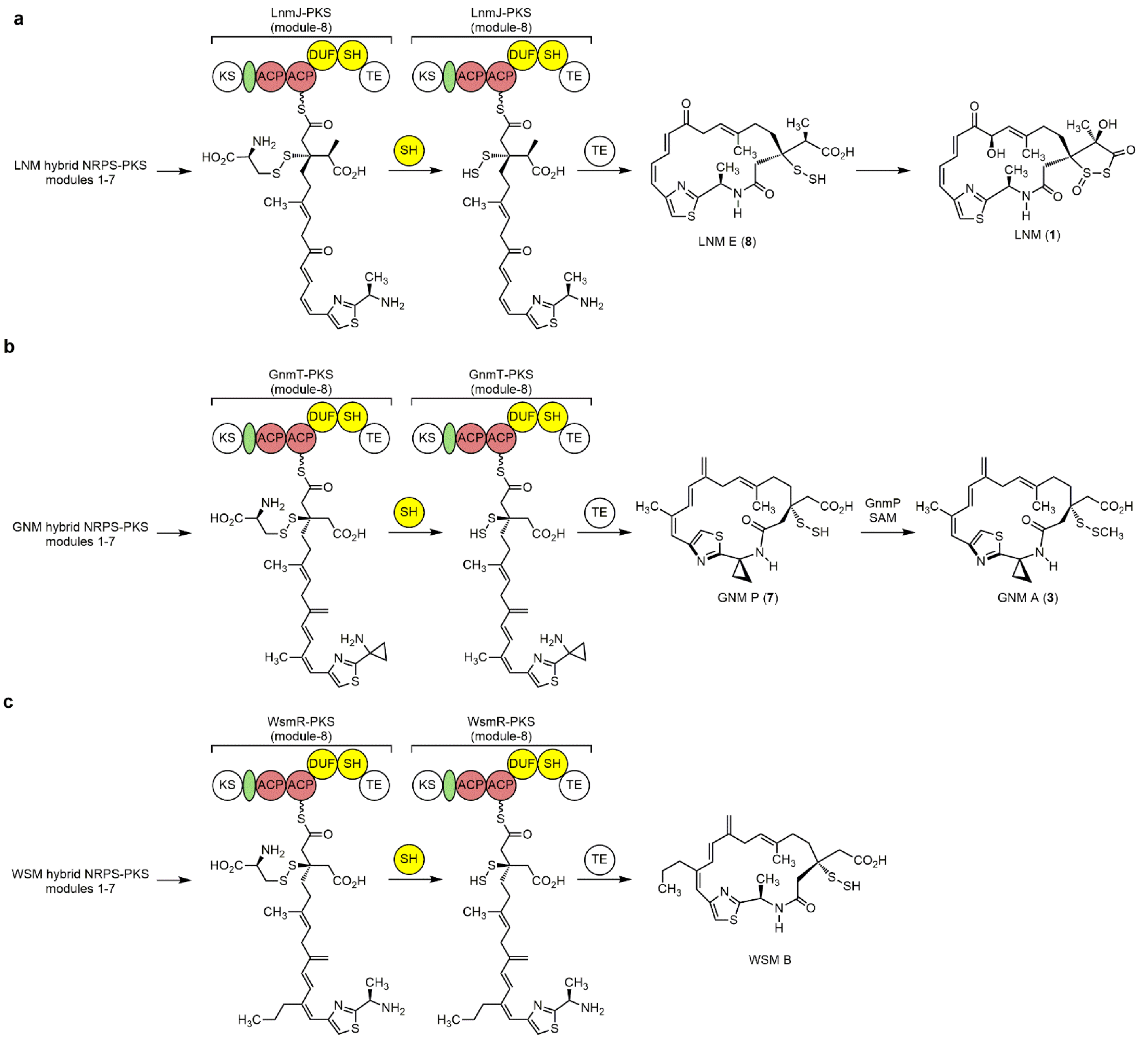

Supplementary Figure 21. A unified pathway for the biosynthesis of the LNM family of natural products. The pathways consist of a hybrid NRPS-PKS assembly line featuring DUF-SH didomains, as exemplified by the **a**, LnmJ-SH, **b**, GnmT-SH, and **c**, WsmR-SH domain, acting as a thiocysteine lyase that directly installs a -SSH group into varying polyketide scaffolds.[5,8]

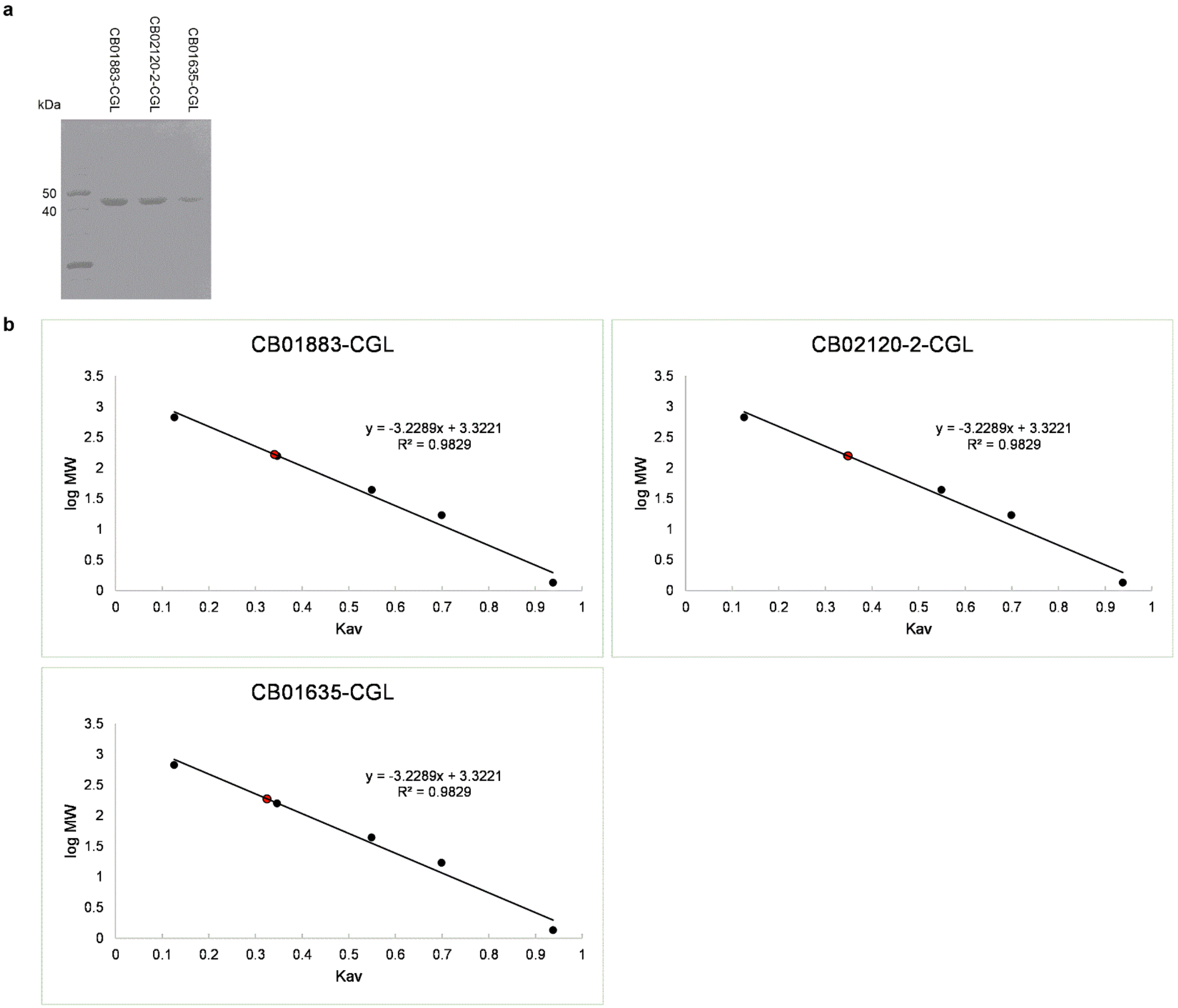

Supplementary Figure 22. Overproduction in *E. coli* and purification of the CGL enzymes utilized in this study. **a**, SDS-PAGE analysis of purified CB01883-CGL, CB02120-2-CGL, and CB01635-CGL enzymes. **b**, Size-exclusion chromatography of the purified CGL enzymes. CB01883-CGL, CB02120-2-CGL, and CB01635-CGL eluted at retention volumes of 69.9, 70.5, and 68.7 mL, respectively, which were each converted to K_{av} according to the equation $K_{av} = (V_e - V_o) / (V_t - V_o)$, where V_e, V_o, and V_t is the elution volume, column void volume, and total bed volume, respectively. The equation provided K_{av} values of 0.34, 0.35, and 0.32 for CB01883-CGL, CB02120-2-CGL, and CB01635-CGL, respectively, which were individually plotted on the calibration curve (red circles) and provided MWs of 167, 157, and 187 kDa. The expected MW of CB01883-CGL, CB02120-2-CGL, and CB01635-CGL were 44, 44, and 43 kDa, respectively. Thus, the CGL enzymes are supported as homotetramers in solution.

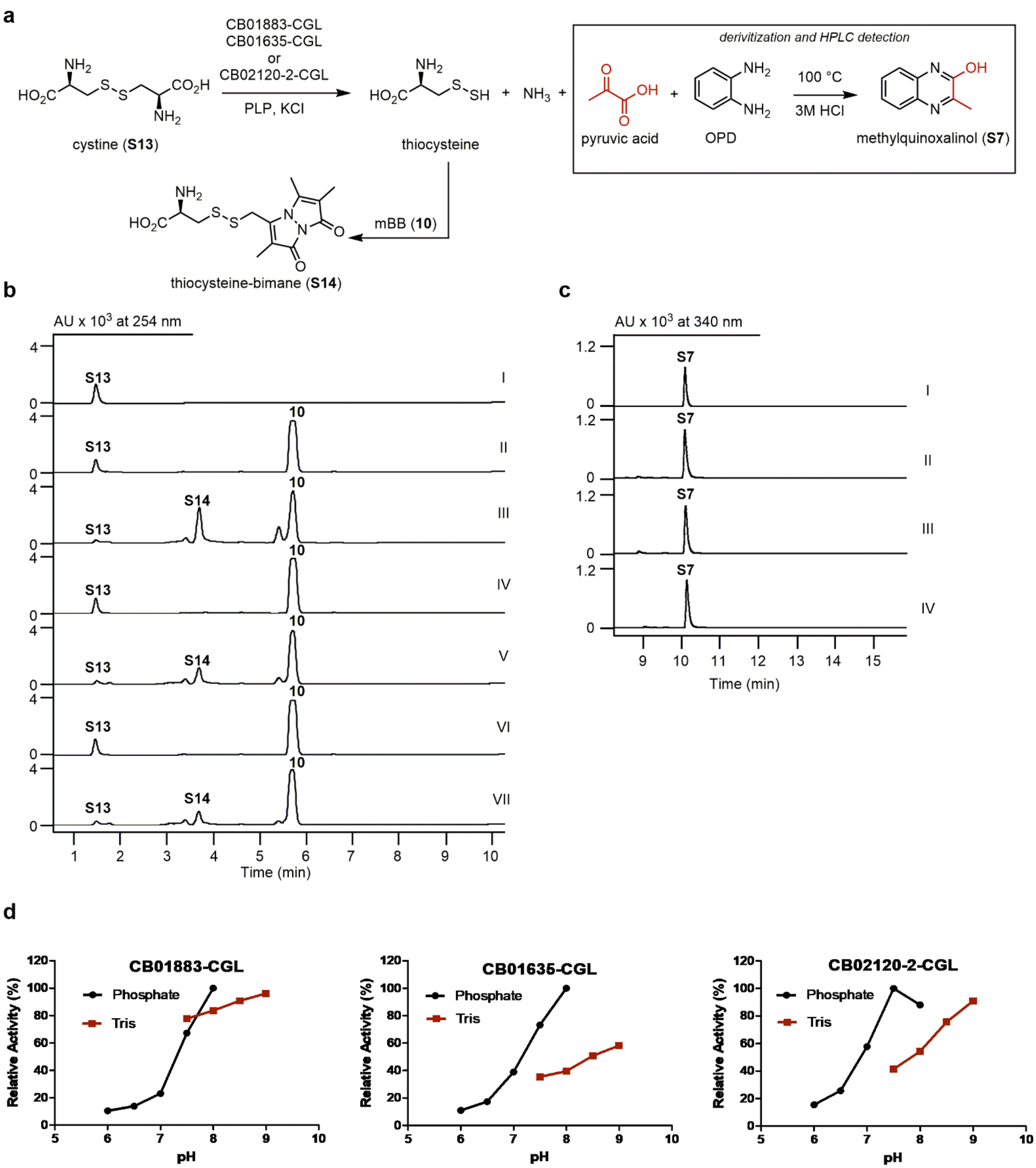
a
CB01883-CGL
CB01635-CGL
or
CB02120-2-CGL
PLP, KCl
cystine (S13)
thiocysteine
NH3
derivitization and HPLC detection
100 °C
3M HCl
pyruvic acid
OPD
methylquinoxalinol (S7)
mBB (10)
thiocysteine-bimane (S14)
b
AU x 10^3 at 254 nm
S13
S14
10
I
II
III
IV
V
VI
VII
Time (min)
c
AU x 10^3 at 340 nm
S7
Time (min)
d
CB01883-CGL
CB01635-CGL
CB02120-2-CGL
Phosphate
Tris
Relative Activity (%)
pH

e

enzyme	% conv.
CB01883-CGL	18.5
CB01635-CGL	15.3
CB02120-2-CGL	12.8

f

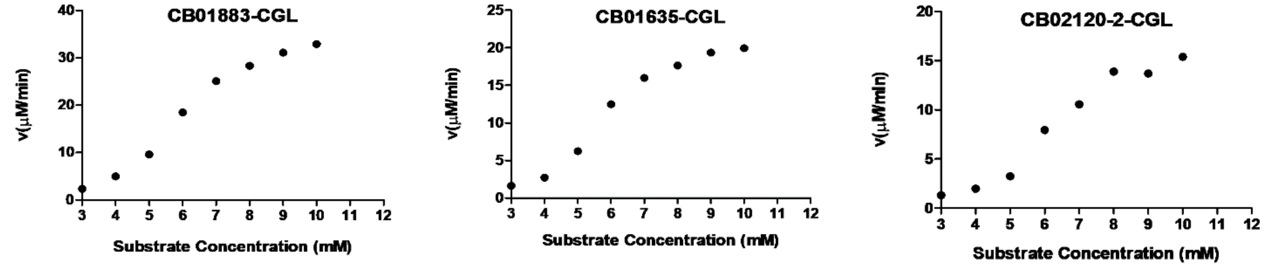

g

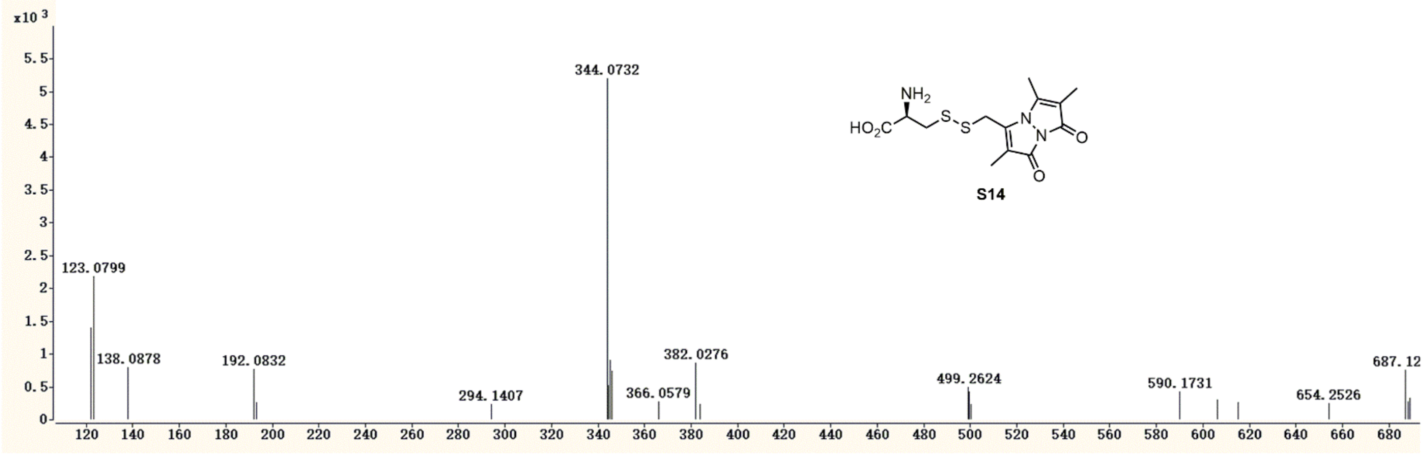

Supplementary Figure 23. CGL enzymes from LNM-, GNM-, and WSM-producing strains as a source of thiocysteine. **a**, In vitro formation of thiocysteine from L-cystine (**S13**) catalyzed by CGL enzymes,[9] with trapping by mBB (**10**), or trapping of the pyruvate product with OPD (**S7**). **b**, HPLC analysis of CGL-catalyzed formation of thiocysteine from **S13**, with trapping by mBB (**10**) to form **S14**. (I) Substrate **S13**, (II) CB01883-CGL (boiled) + **S13** + **10**, (III) CB01883-CGL + **S13** + **10**, (IV) CB01635-CGL (boiled) + **S13** + **10**, (V) CB01635-CGL + **S13** + **10**, (VI) CB02120-2-CGL (boiled) + **S13** + **10**, (VII) CB02120-2-CGL (boiled) + **S13** + **10**. **c**, HPLC analysis of pyruvate formation from CB01883-CGL, CB01635-CGL, and CB02120-2-CGL with **S13** as substrate, upon derivatization with OPD. (I) methylquinoxalinol (**S7**) standard from the reaction of pyruvate with OPD, (II) **S13** with CB01883-CGL, (III) **S13** with CB01635-CGL, (IV) **S13** with CB02120-2-CGL. **d**, pH optimization of CGL enzymes with substrate **S13**, with n=2 independent experiments. **e**, End-point assay of CGL-catalyzed thiocysteine formation after a 90-minute reaction with 5 mM **S13**, with n=2 independent experiments. **f**, Michaelis-Menten plots of CGL enzymes (10 µM) with **S13** (3 – 10 mM) as the substrate, with n=2 independent experiments. The poor solubility of **S13** prevented reliable determination of k_{cat} or K_m, and the value of k_{cat}/K_m was estimated from the slope of a linear fit between the points at 5, 6, and 7 mM substrate concentration. CB01883-CGL, CB02120-2-CGL and CB01635-CGL with substrate **S13** yielded k_{cat}/K_m values of 0.77 $min^{-1}mM^{-1}$, 0.37 $min^{-1}mM^{-1}$, and 0.49 $min^{-1}mM^{-1}$, respectively. **g**, HRMS (ESI) spectrum of **S14**. Data are presented as mean values.

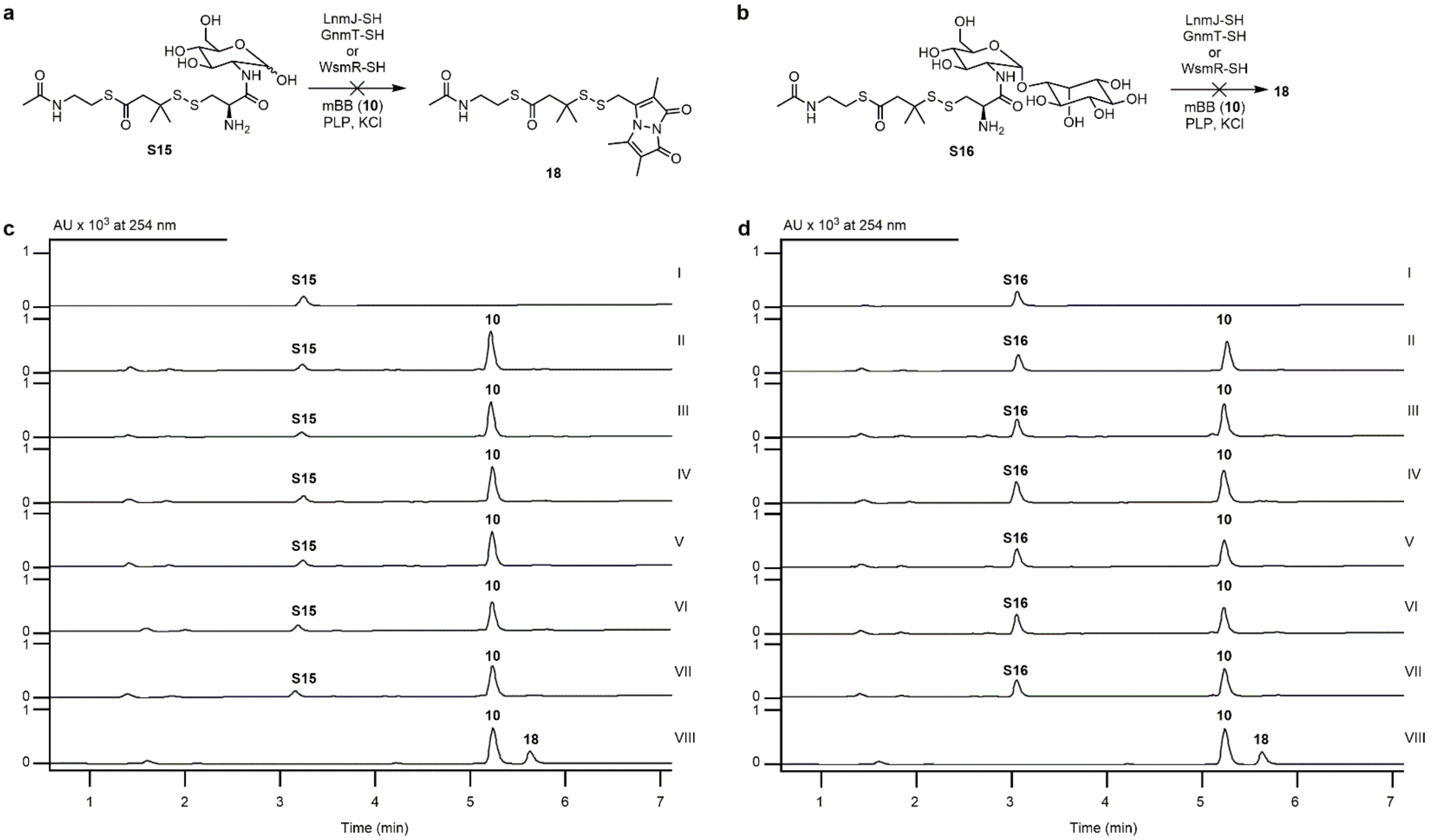

Supplementary Figure 24. Deacetylated mycothiol persulfide adducts are not viable substrates for LnmJ-SH, GnmT-SH, or WsmR-SH. **a**, Attempted in vitro formation of persulfide and its mBB-adduct **18** from mycothiol substrate mimics **S15** and **b**, **S16**. **c**, HPLC analysis of mBB-trapping experiments with substrate **S15**, with **17** serving as a positive control. (I) Substrate **S15**, (II) **S15** + GnmT-SH (boiled) + **10**, (III) **S15** + GnmT-SH + **10**, (IV) **S15** + LnmJ-SH (boiled) + **10**, (V) **S15** + LnmJ-SH + **10**, (VI) **S15** + WsmR-SH (boiled) + **10**, (VII) **S15** + WsmR-SH + **10**, (VIII) **17** + GnmT-SH + **10**. **d**, HPLC analysis of mBB-trapping experiments with substrate **S16**, with **17** serving as a positive control. (I) substrate **S16**, (II) **S16** + GnmT-SH (boiled) + **10**, (III) **S16** + GnmT-SH + **10**, (IV) **S16** + LnmJ-SH (boiled) + **10**, (V) **S16** + LnmJ-SH + **10**, (VI) **S16** + WsmR-SH (boiled) + **10**, (VII) **S16** + WsmR-SH + **10**, (VII) **17** + GnmT-SH + **10**.

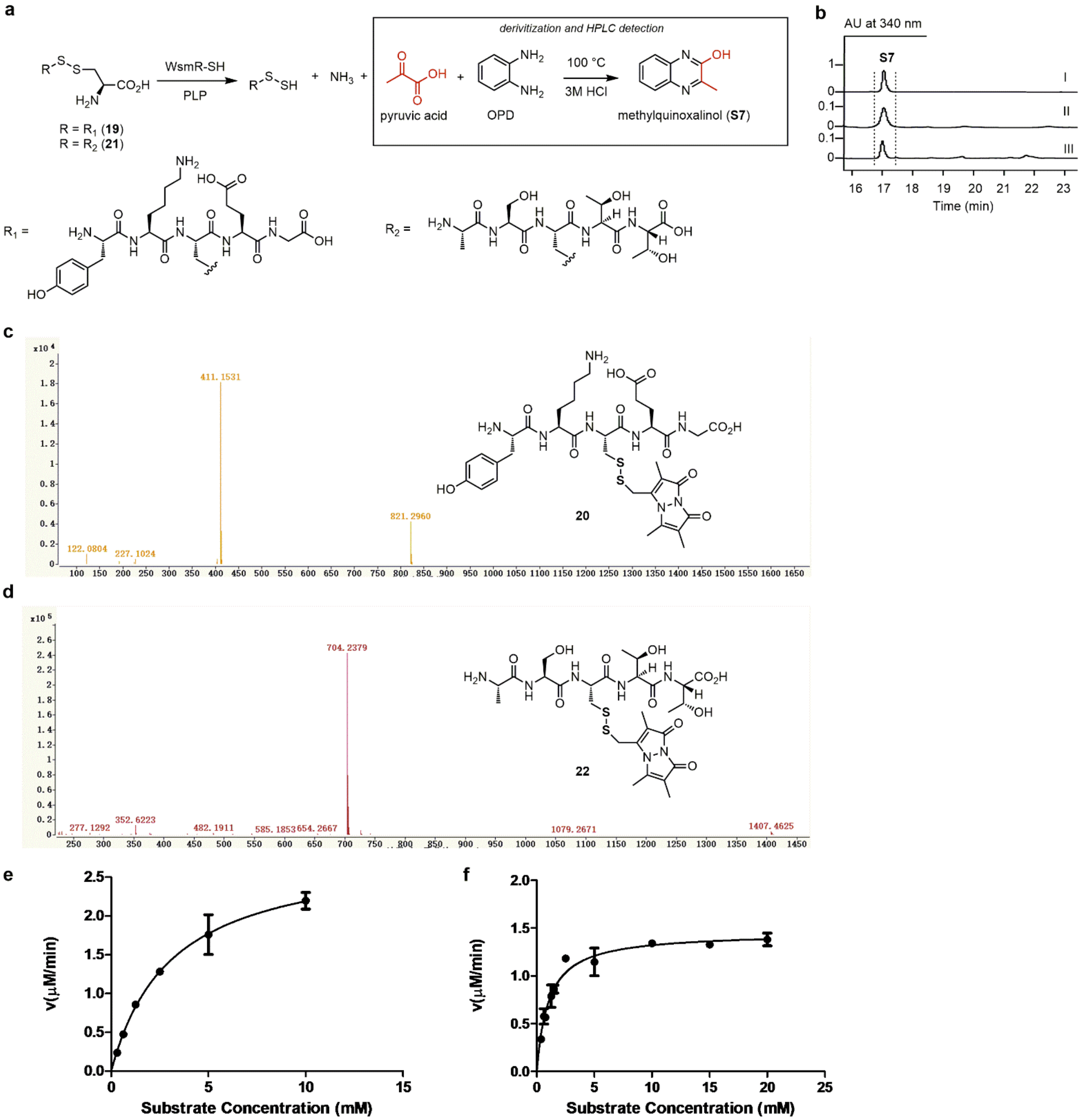

Supplementary Figure 25. Analysis of WsmR-SH-catalyzed peptide persulfide formation reactions. **a**, SH-catalyzed β-elimination with concomitant pyruvate formation and trapping in situ with *o*-phenylenediamine (OPD) to form methylquinoxalinol (**S7**), with substrates **19** and **21**. **b**, HPLC analysis of pyruvate formation from WsmR-SH with **19** and **21** as substrates, upon derivatization with OPD. (I) methylquinoxalinol (**S7**) standard from the reaction of pyruvate with OPD, (II) **19** with WsmR-SH, (III) **21** with WsmR-SH. **c**, HRMS (ESI) spectrum of mBB-adduct **20** and **d**, HRMS (ESI) spectrum of mBB-adduct **22** (see main text, Fig. 5). Michaelis-Menten plots of WsmR-SH at 30 μM with varying concentrations of **e**, substrate **19** (0.306 – 10 mM) and **f**, substrate **21** (0.375 – 20 mM), affording WsmR-SH at K_m = 3.1 ± 0.5 mM and k_{cat} = 0.096 ± 0.007 min^{-1} for **19** and K_m = 1.0 ± 0.1 mM and k_{cat} = 0.049 ± 0.002 min^{-1} for **21**. All data were from n=3 independent experiments and are presented as mean values ± S.E.M, with error bars representing the S.E.M values.

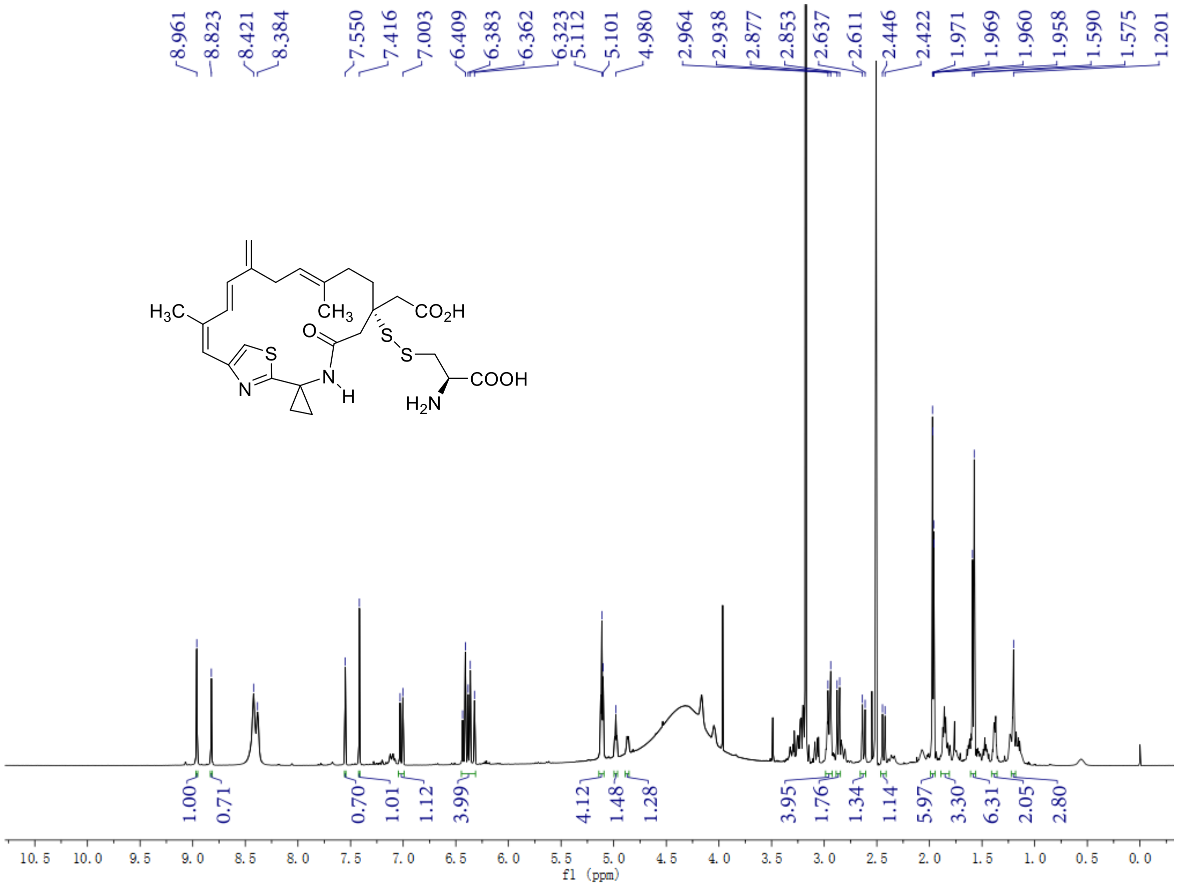

Supplementary Figure 26. ^{1}H NMR spectrum of **9** (600 MHz, DMSO-d_6).

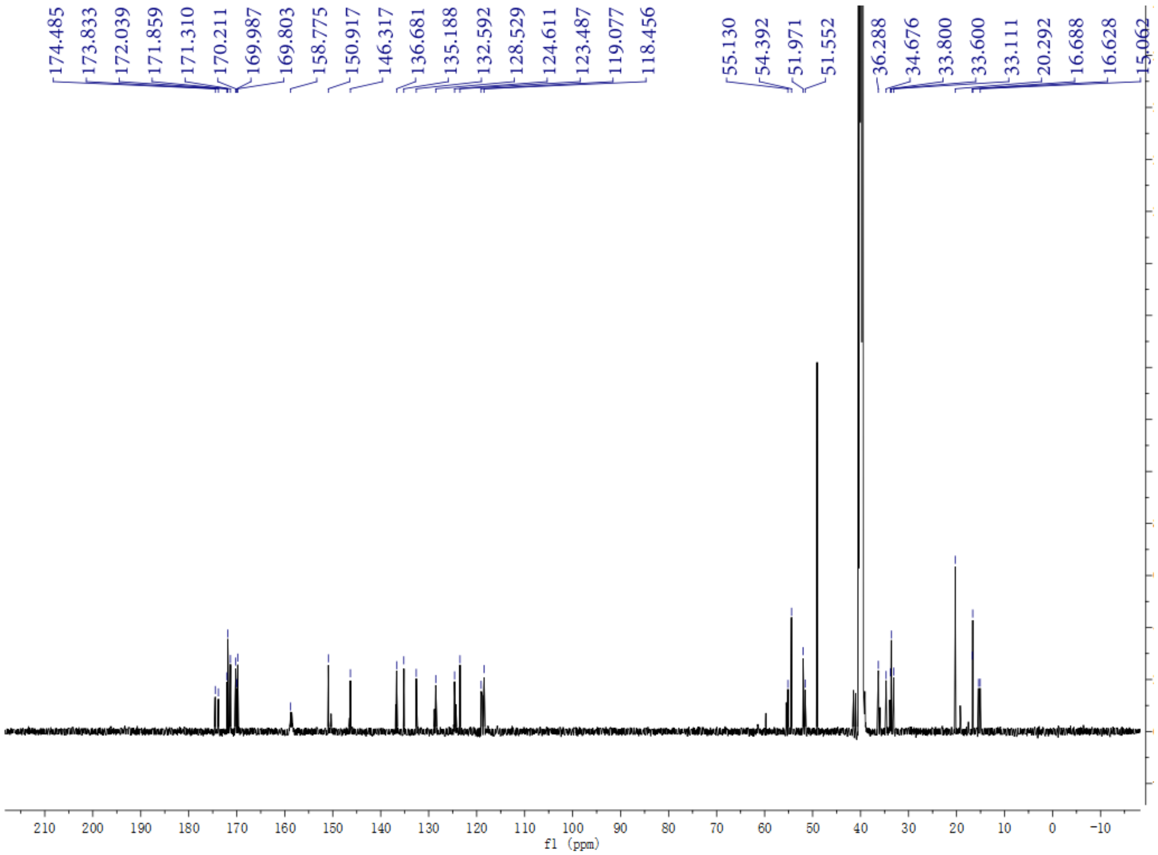

Supplementary Figure 27. ^{13}C NMR spectrum of **9** (150 MHz, DMSO-d_6).

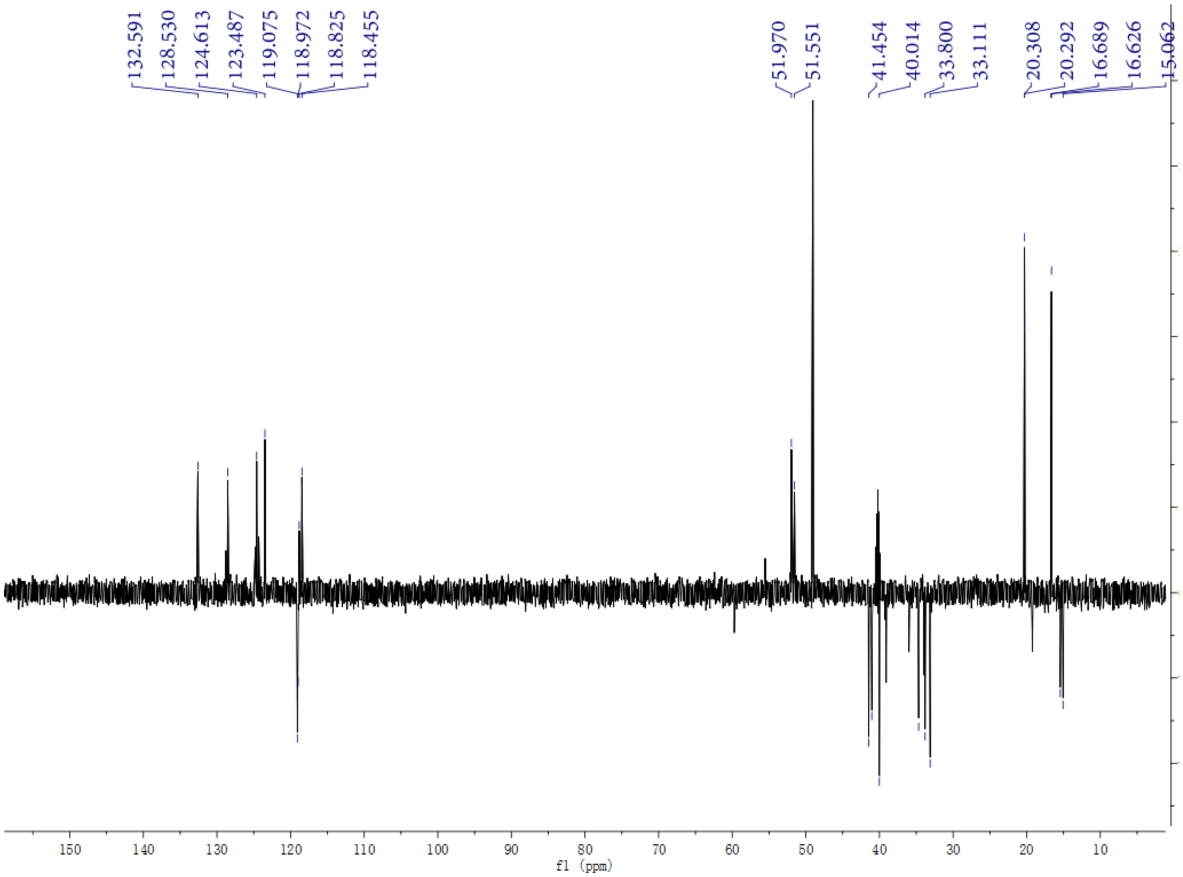

Supplementary Figure 28. DEPT-135 spectrum of **9** (DMSO-d_6).

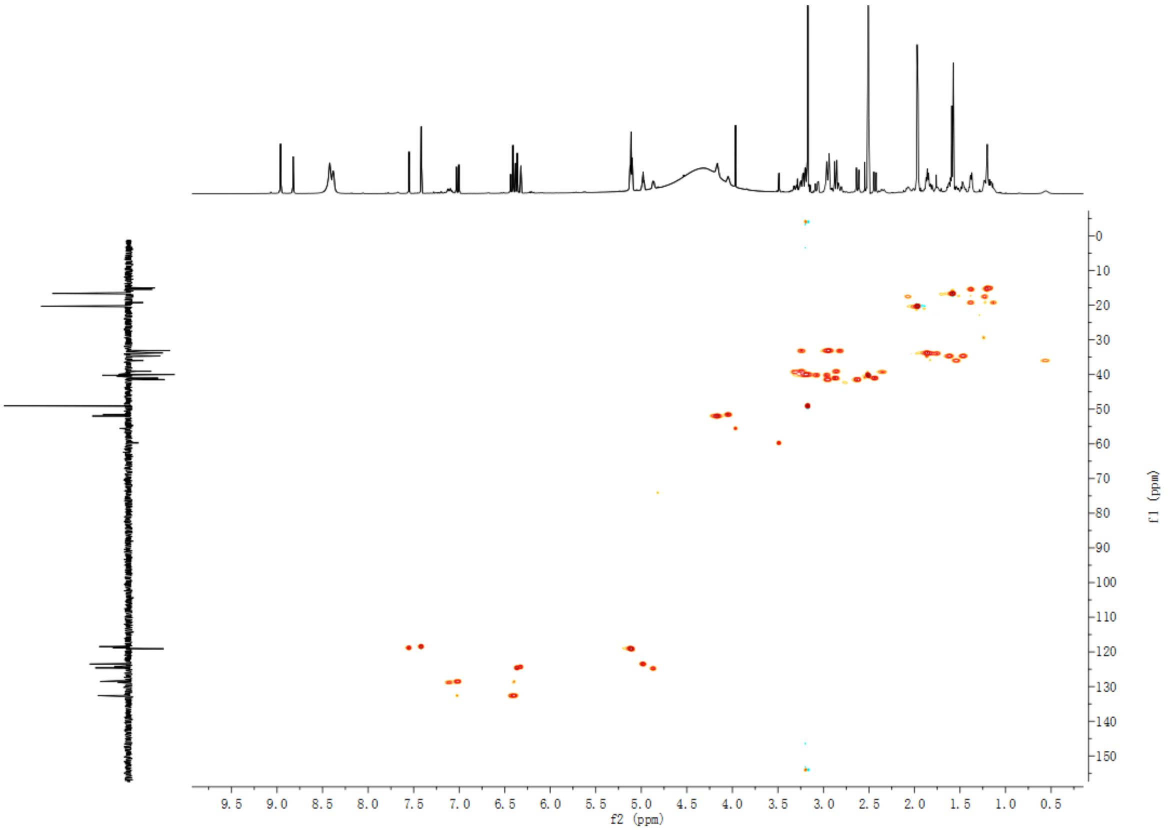

Supplementary Figure 29. HSQC spectrum of **9** (DMSO-d_6).

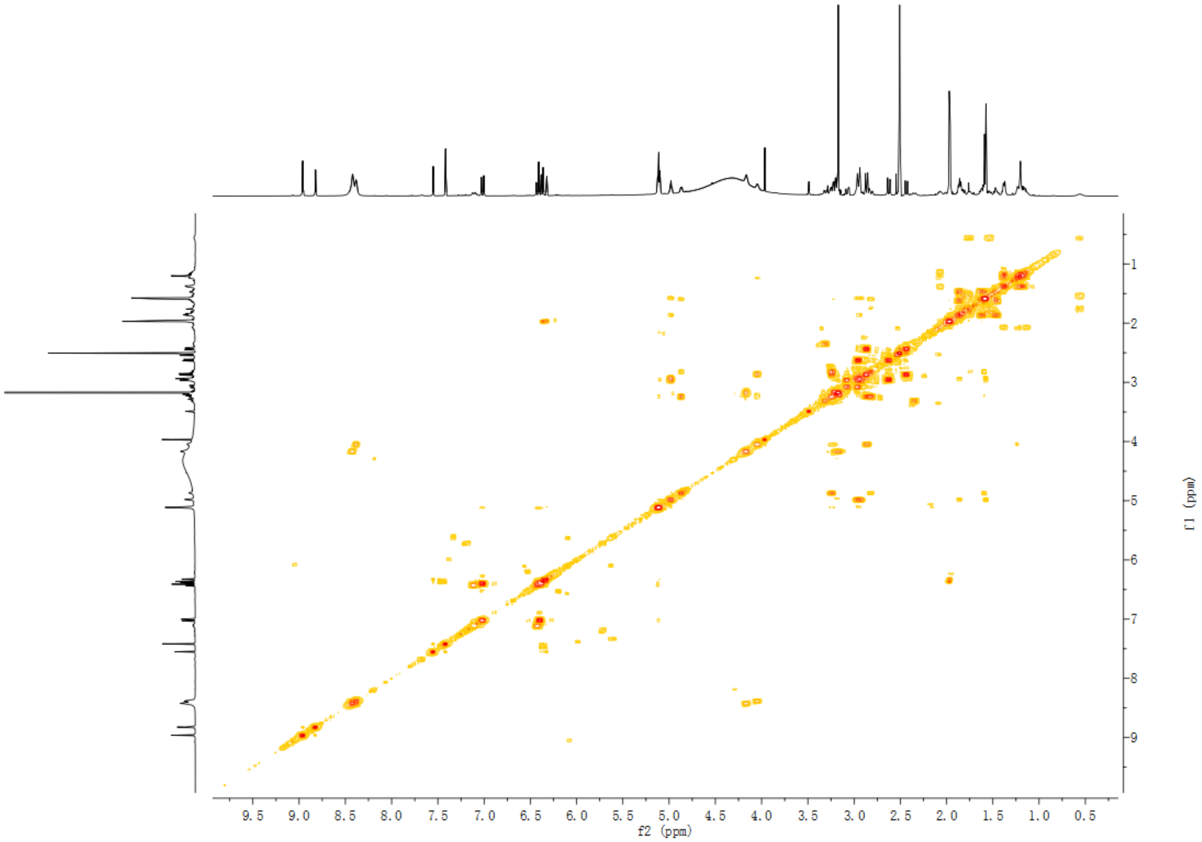

Supplementary Figure 30. ^{1}H-^{1}H COSY spectrum of **9** (DMSO-d_6).

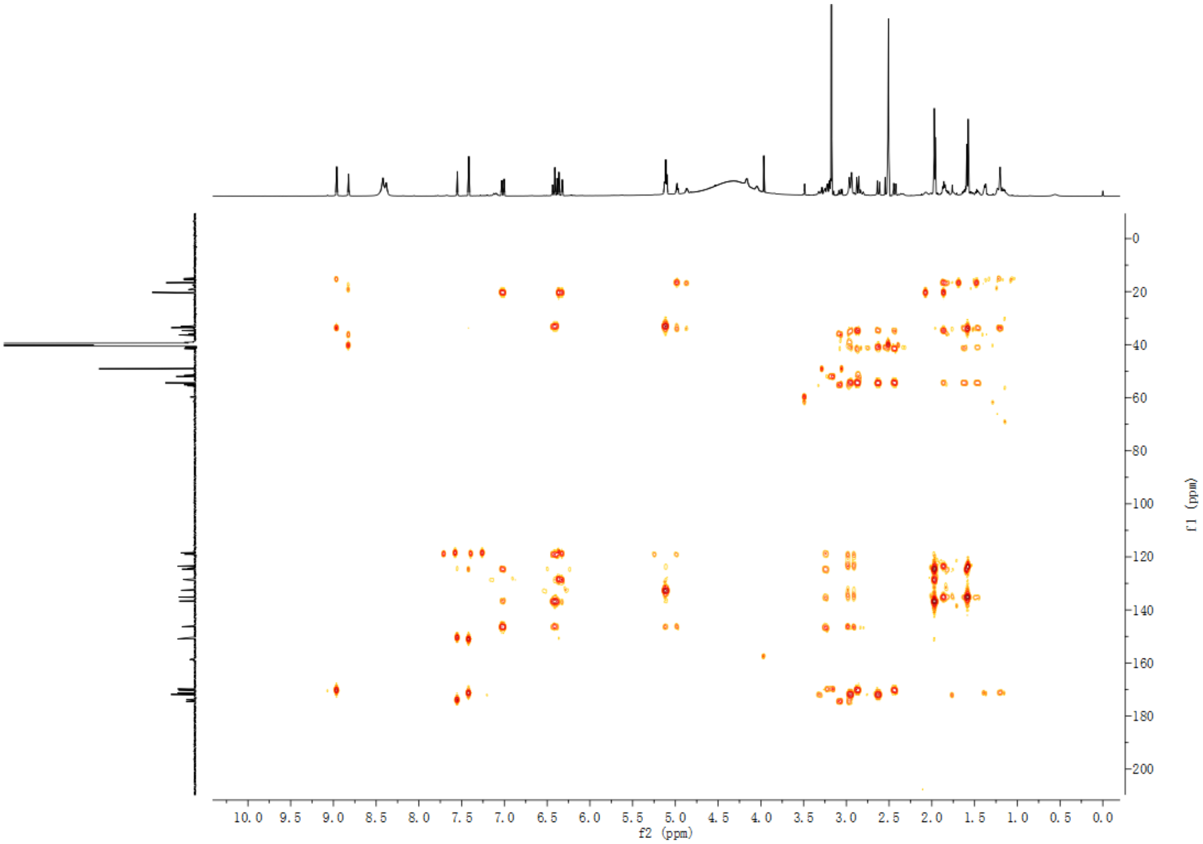

Supplementary Figure 31. HMBC spectrum of **9** (DMSO-d_6).

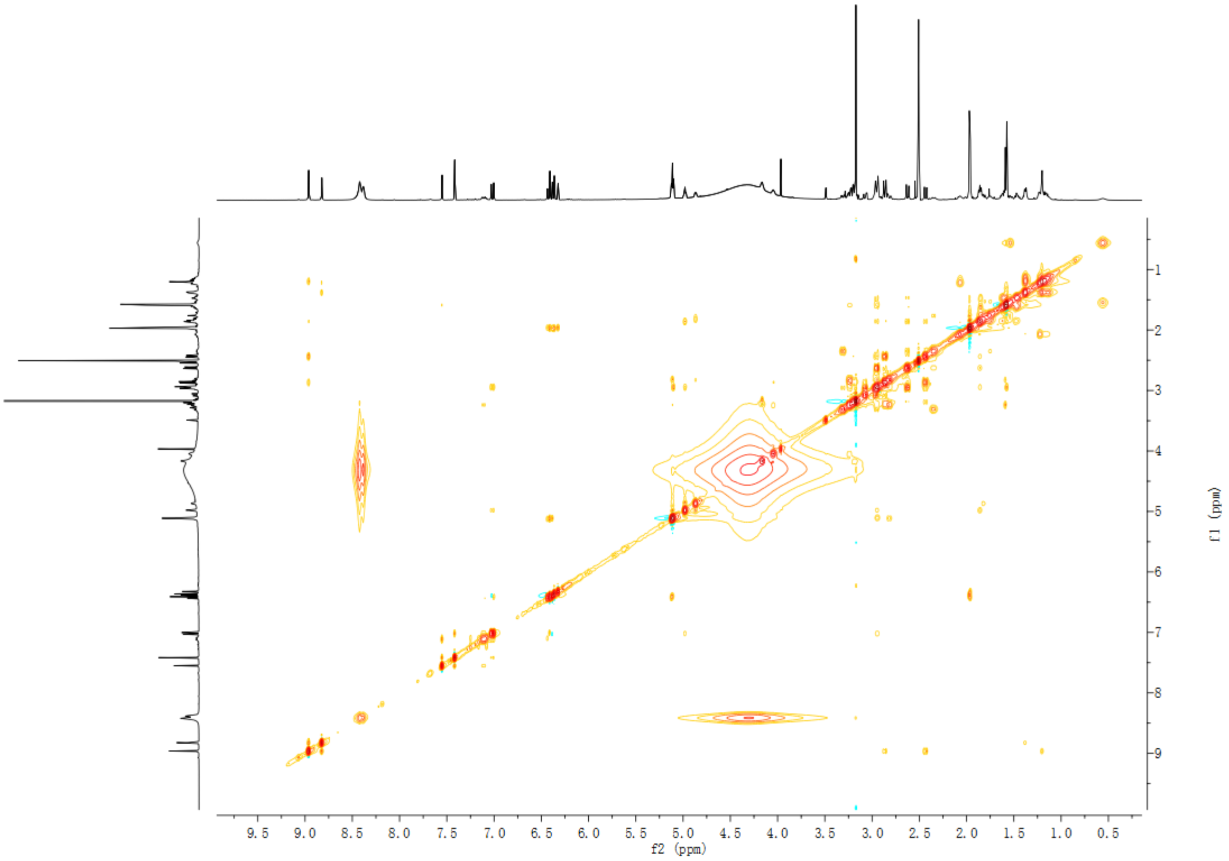

Supplementary Figure 32. ROESY spectrum of **9** (DMSO-d_6).

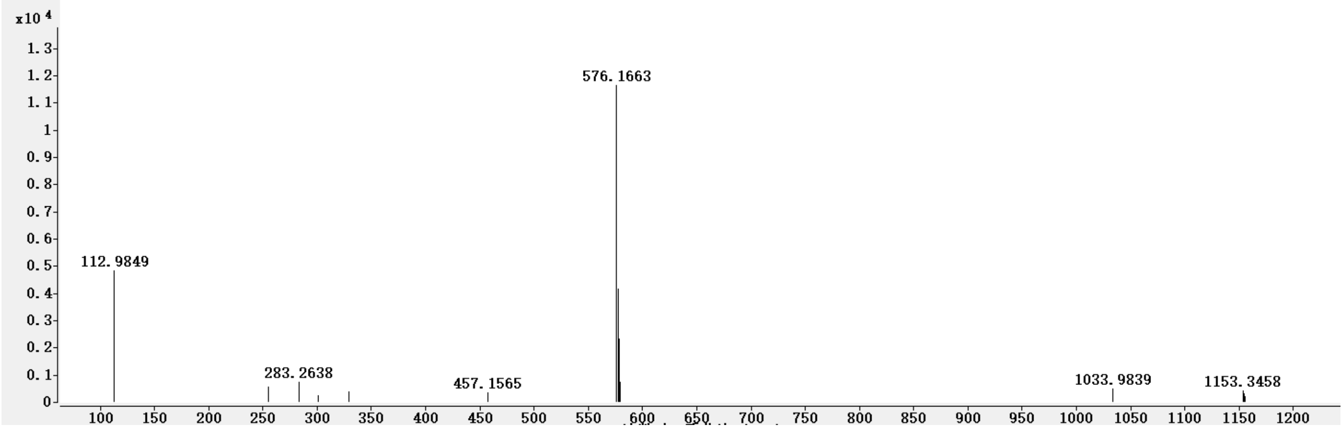

Supplementary Figure 33. HR-MS (ESI) spectrum of **9**.

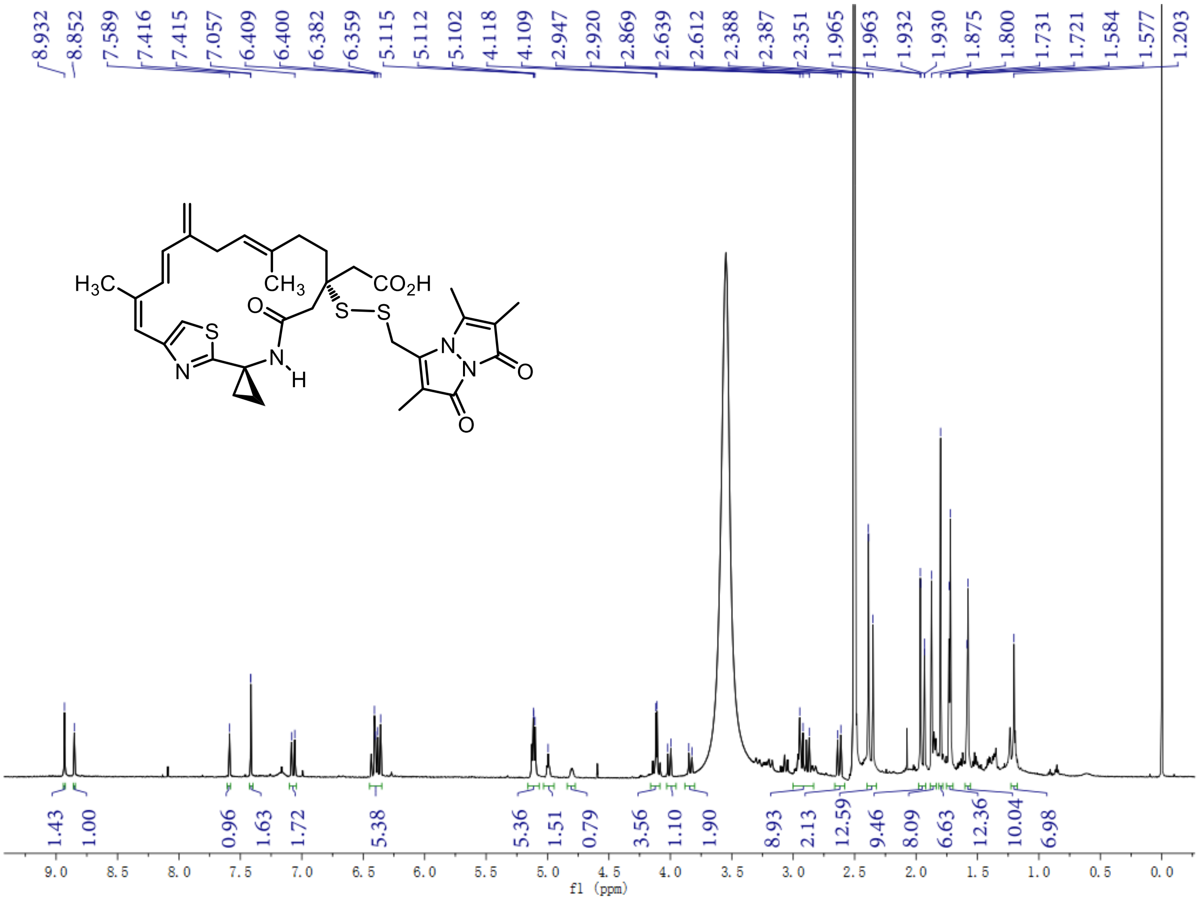

Supplementary Figure 34. ^{1}H NMR spectrum of **11** (600 MHz, DMSO-d_6).

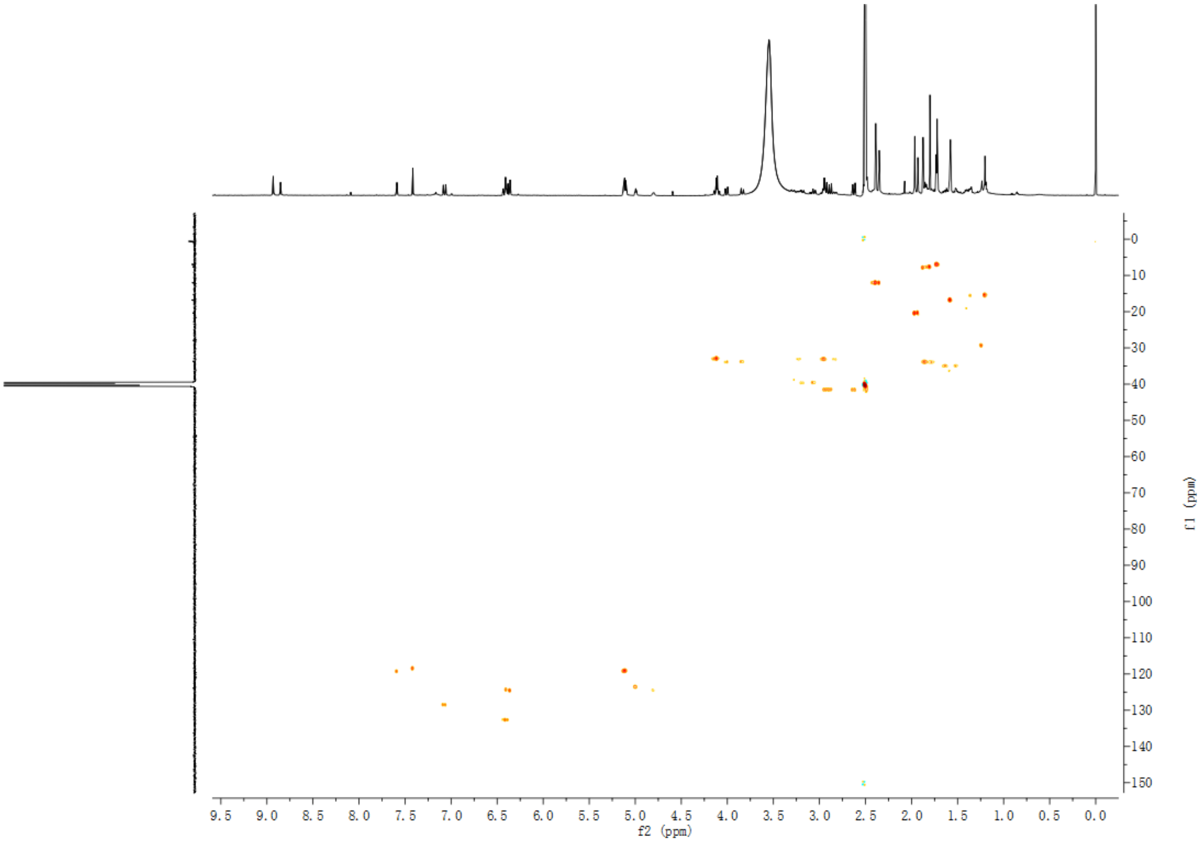

Supplementary Figure 35. HSQC spectrum of **11** (DMSO-d_6).

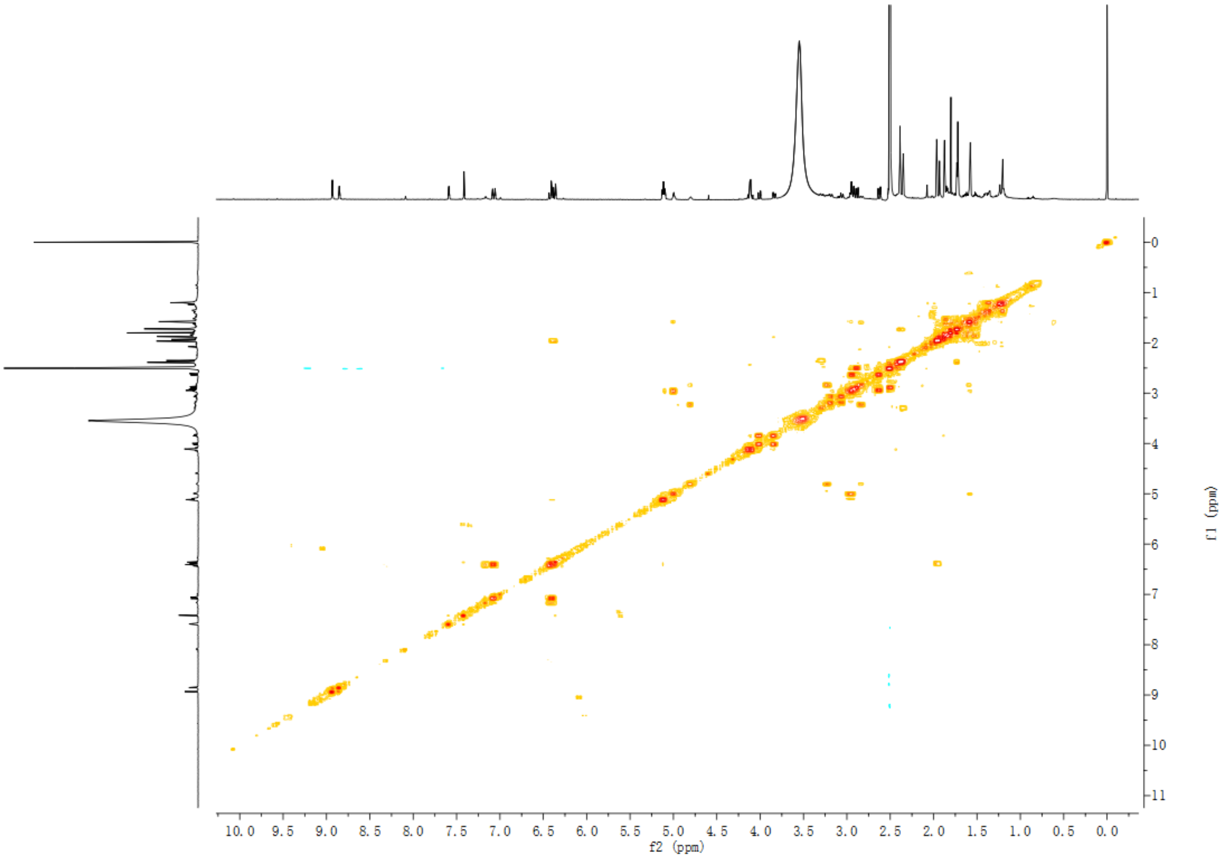

Supplementary Figure 36. ^{1}H-^{1}H COSY spectrum of **11** (DMSO-d_6).

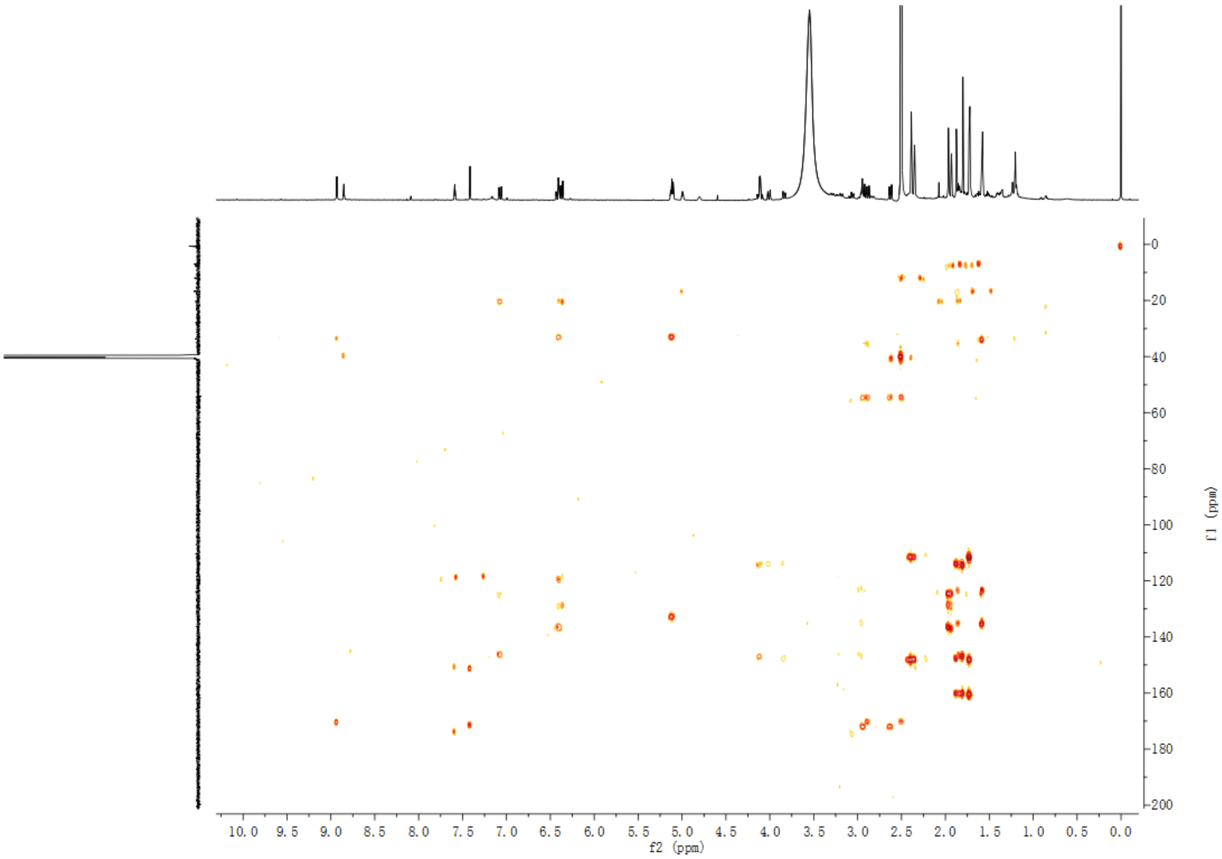

Supplementary Figure 37. HMBC spectrum of **11** (DMSO-d_6).

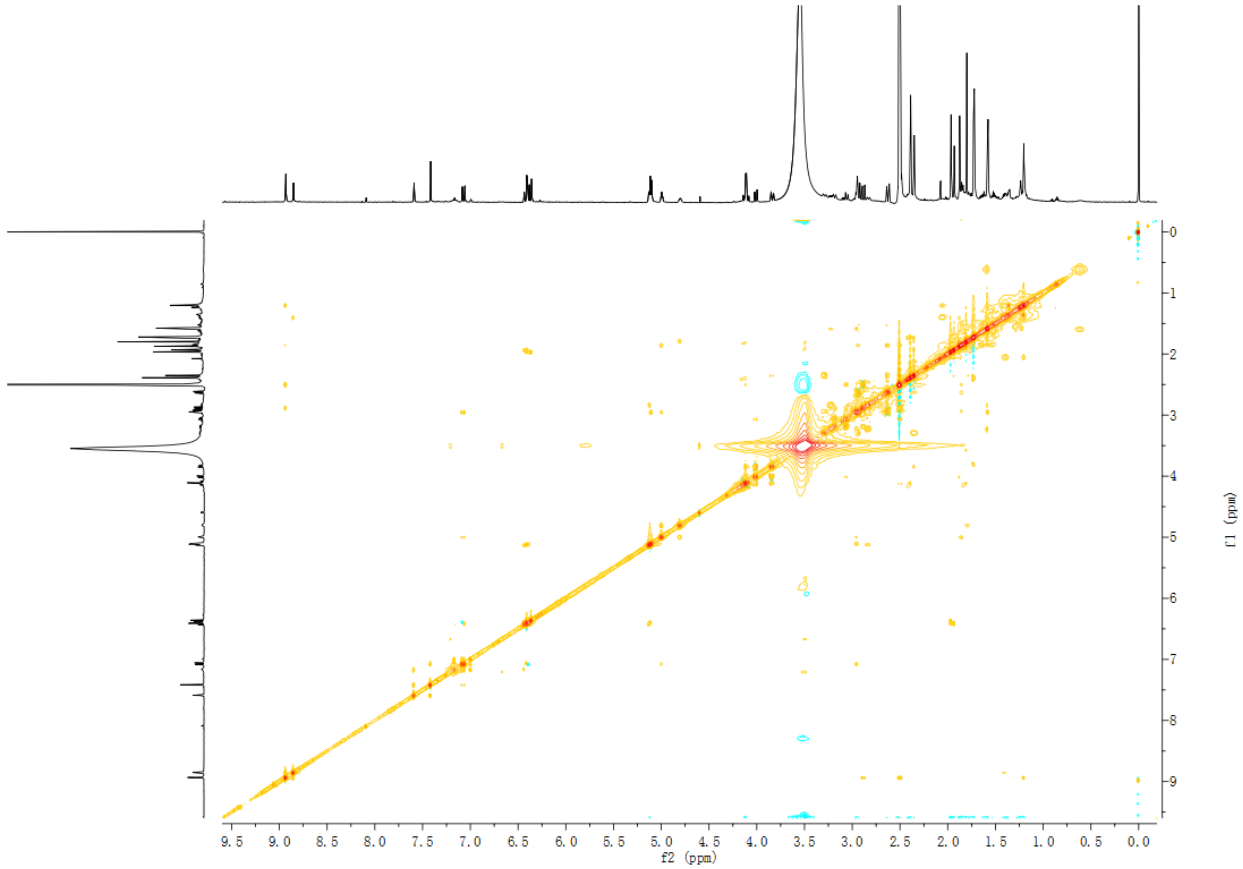

Supplementary Figure 38. ROESY spectrum of **11** (DMSO-d_6).

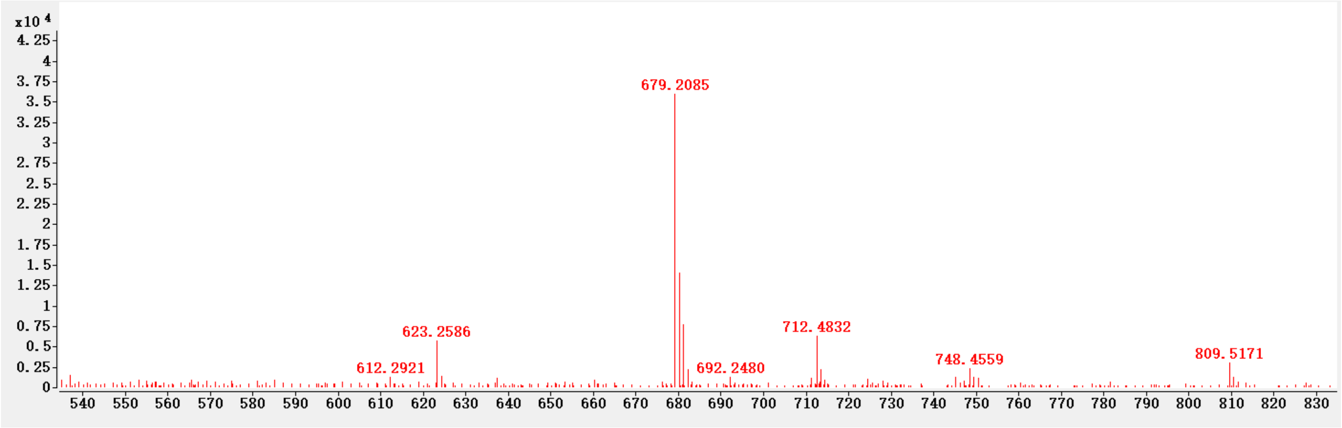

Supplementary Figure 39. HR-MS (ESI) spectrum of **11**.

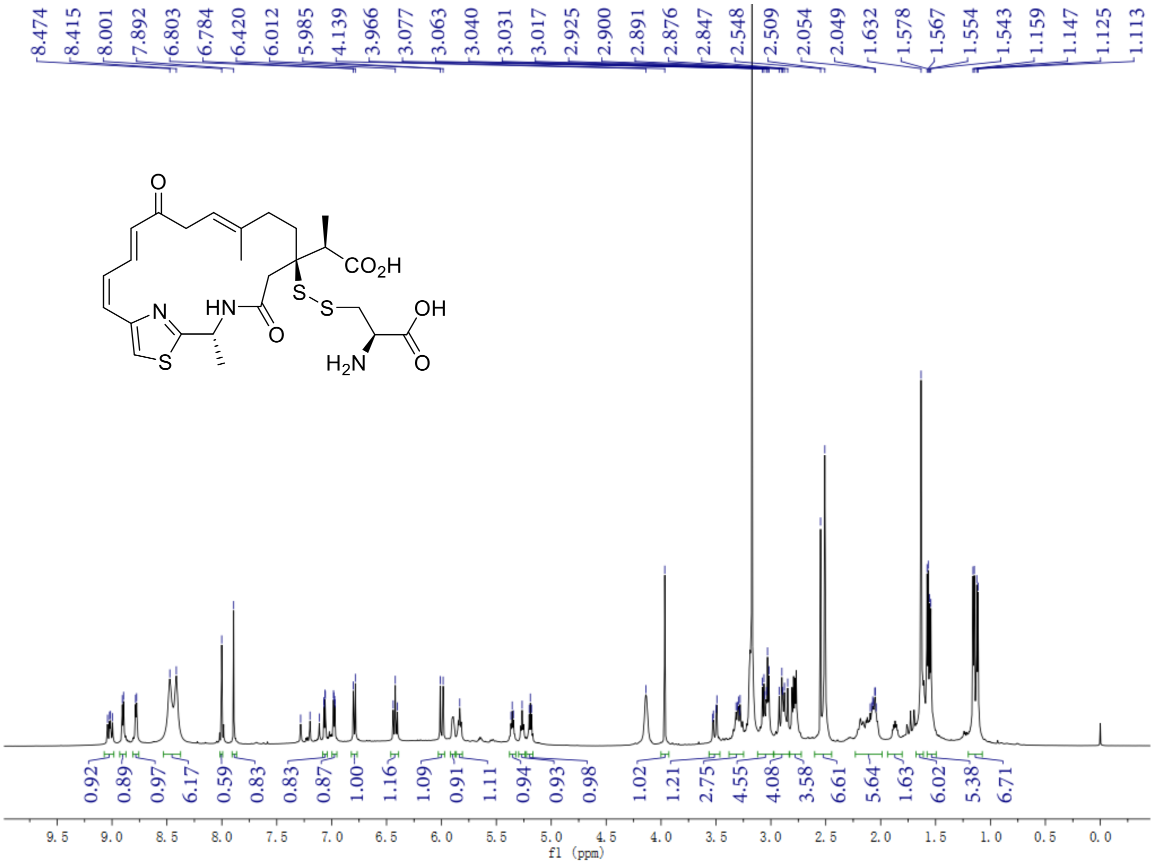

Supplementary Figure 40. ^{1}H NMR spectrum of **13** (600 MHz, DMSO-d_6).

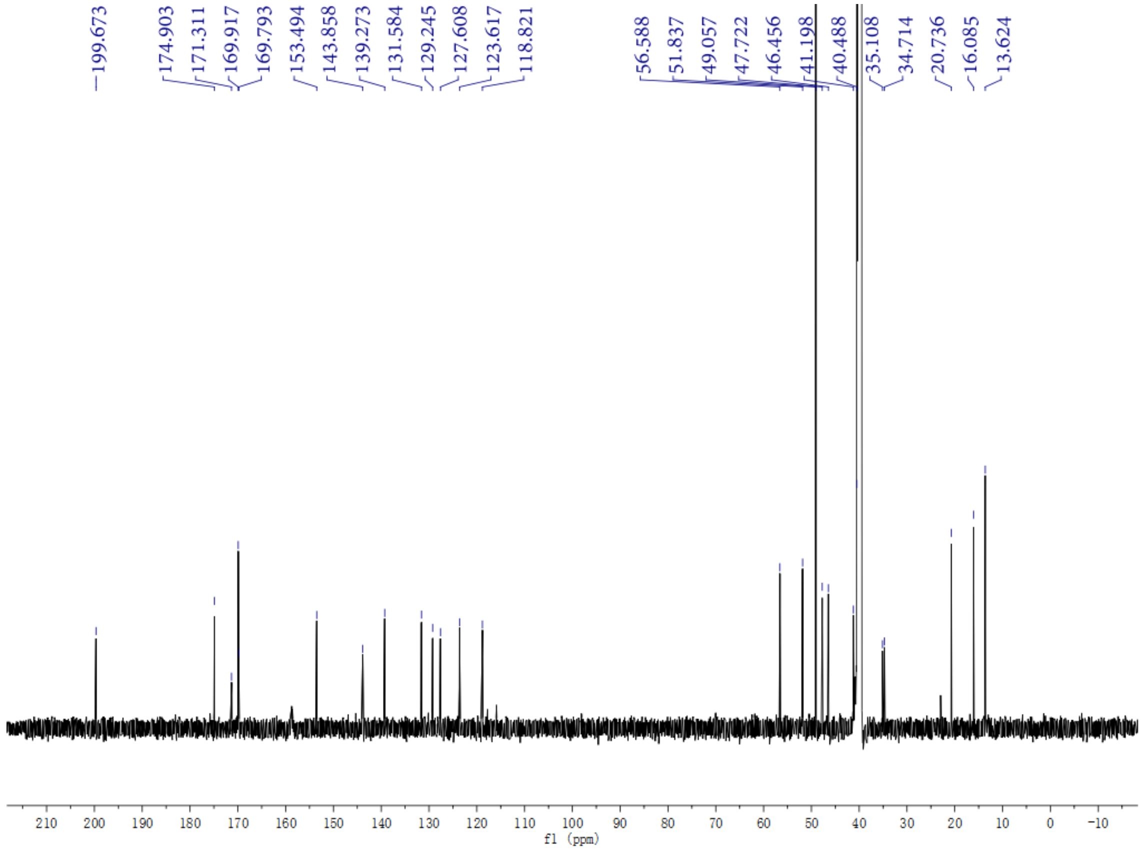

Supplementary Figure 41. ^{13}C NMR spectrum of **13** (150 MHz, DMSO-d_6).

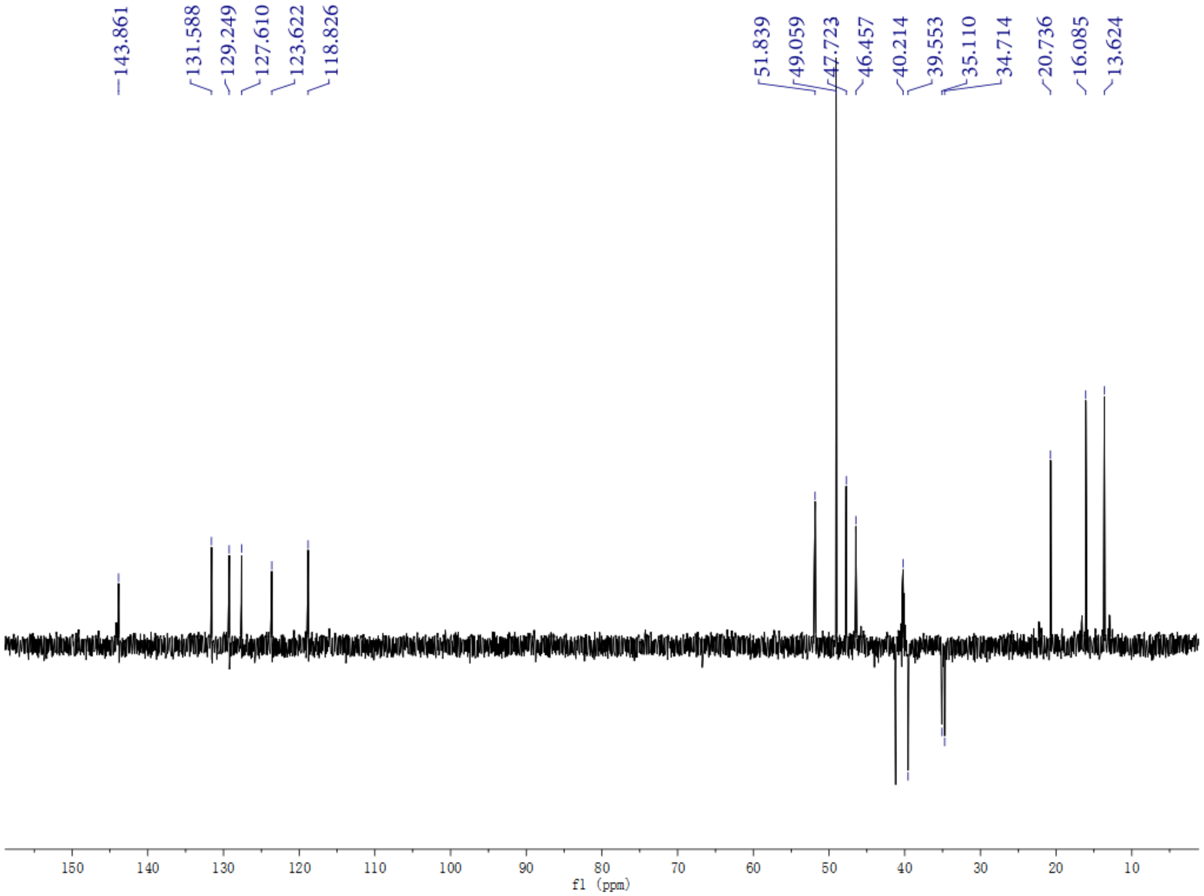

Supplementary Figure 42. DEPT-135 spectrum of **13** (DMSO-d_6).

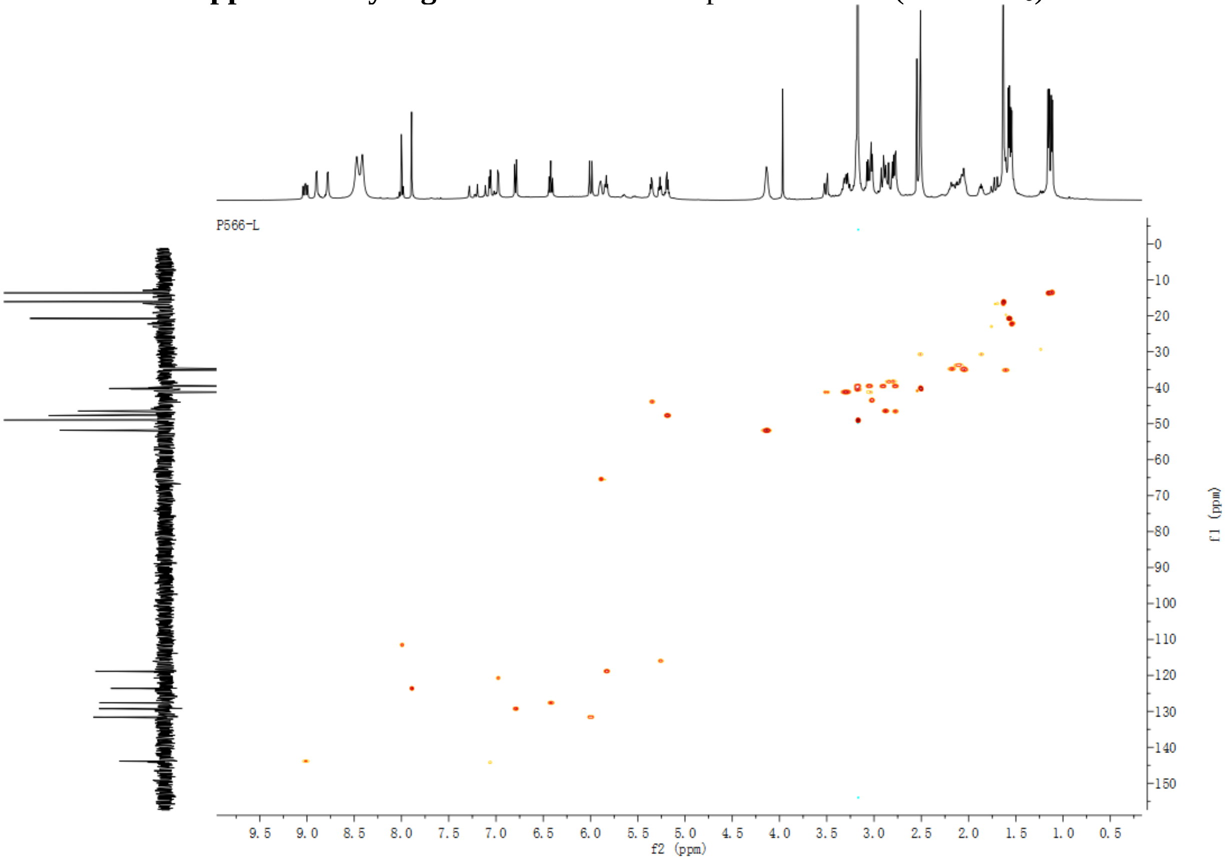

Supplementary Figure 43. HSQC spectrum of **13** (DMSO-d_6).

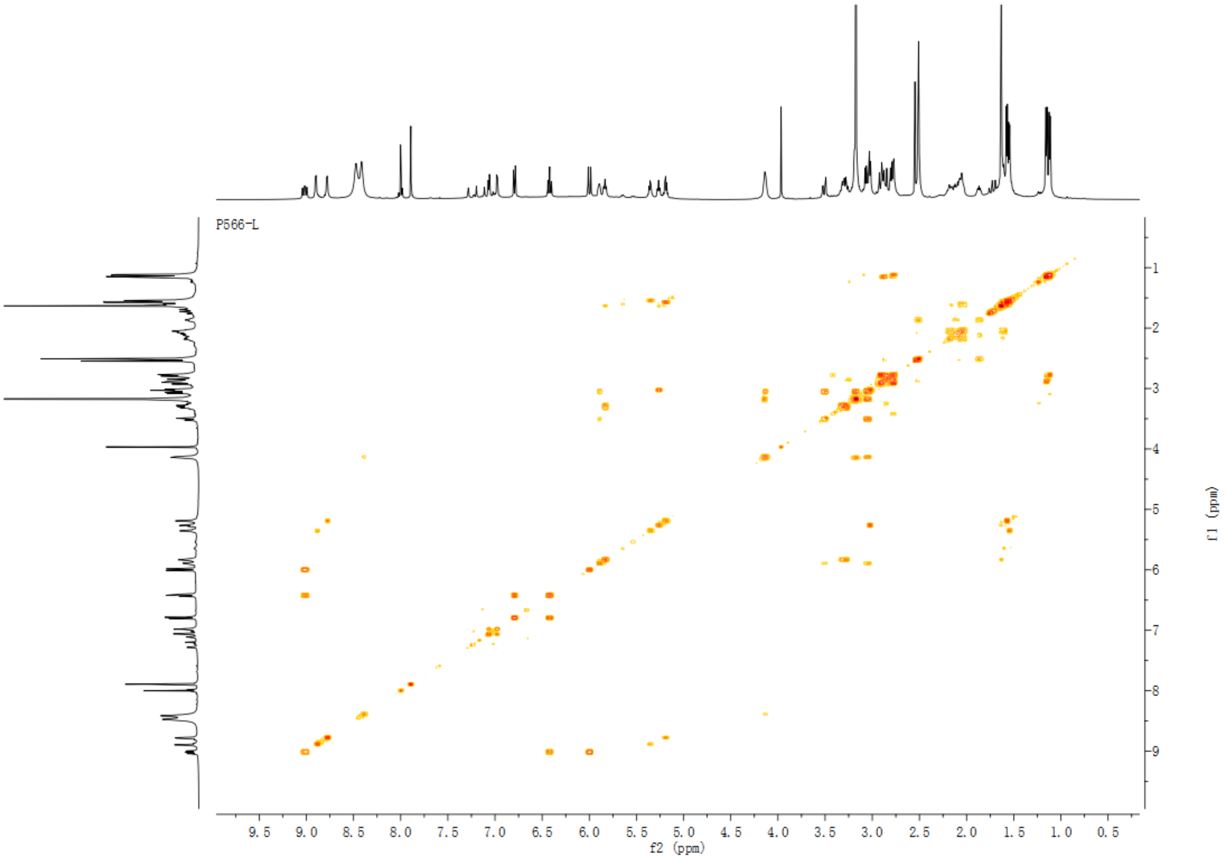

Supplementary Figure 44. ^{1}H-^{1}H COSY spectrum of **13** (DMSO-d_6).

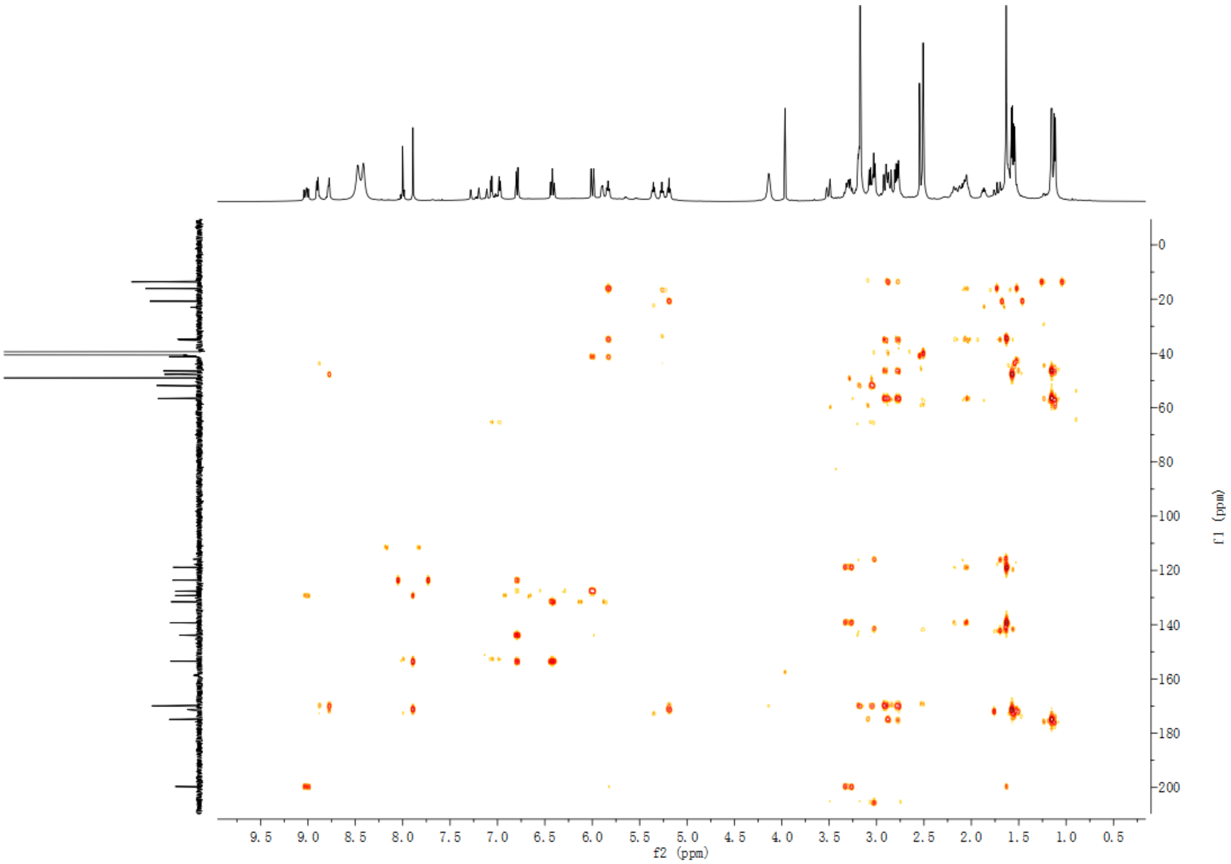

Supplementary Figure 45. HMBC spectrum of **13** (DMSO-d_6).

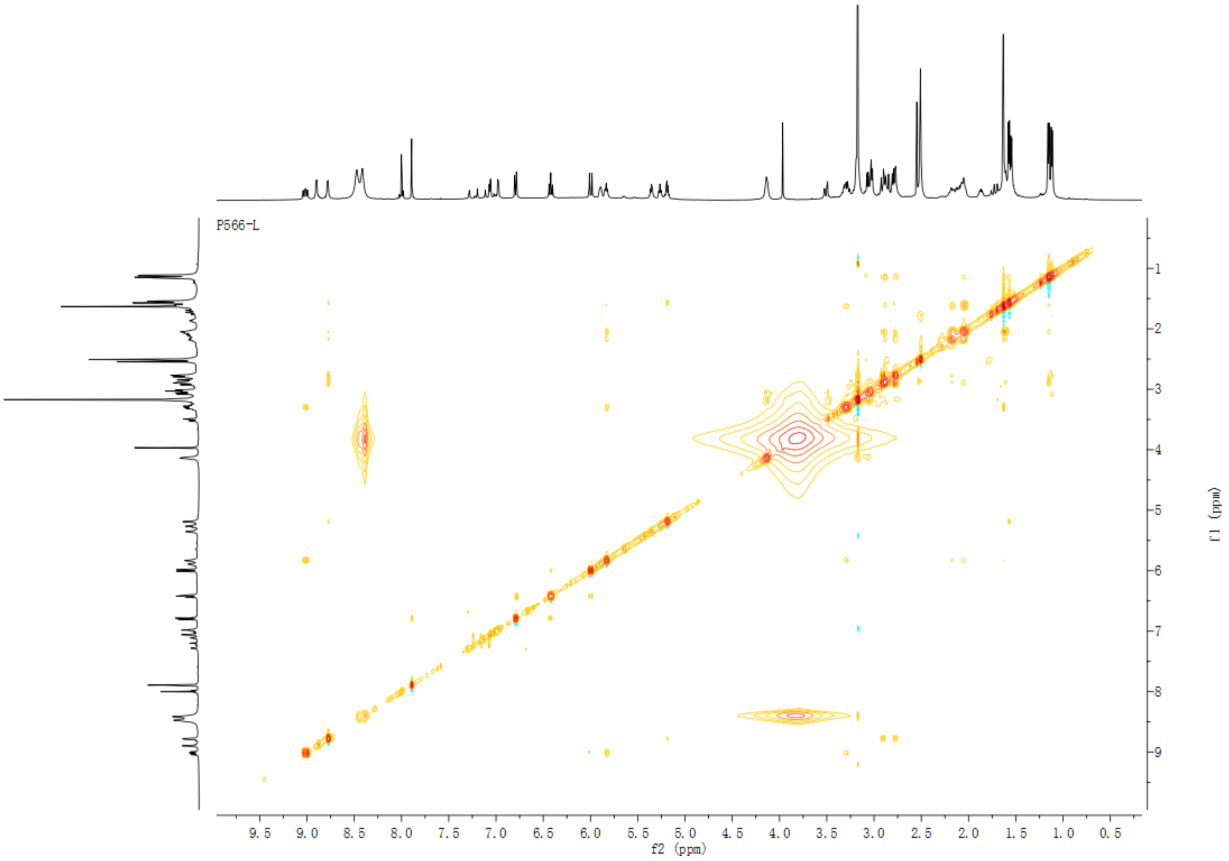

Supplementary Figure 46. ROESY spectrum of **13** (DMSO-d_6).

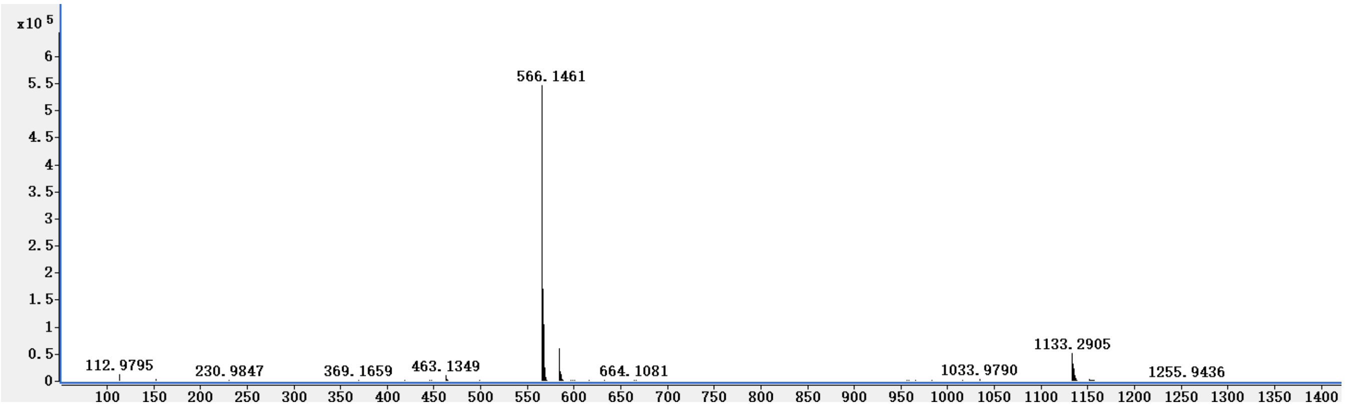

Supplementary Figure 47. HR-MS (ESI) spectrum of **13**.

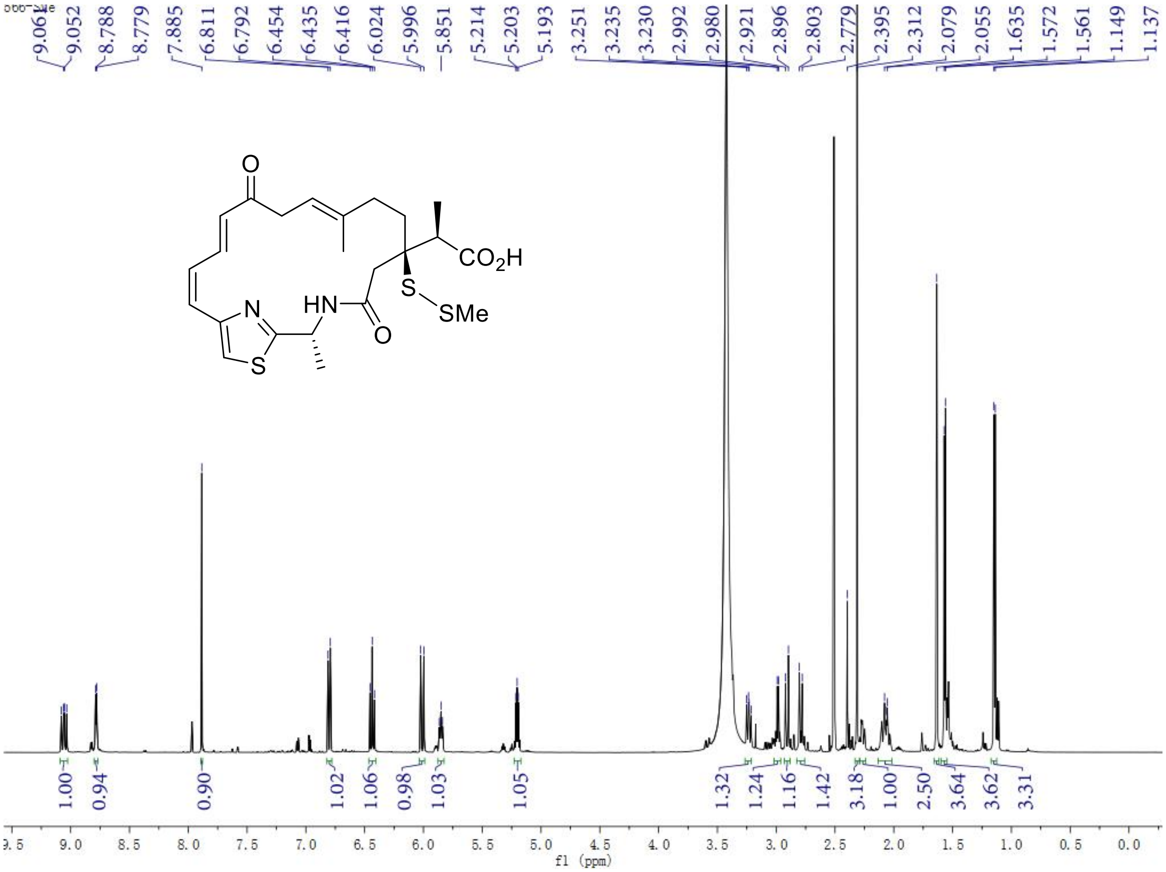

Supplementary Figure 48. ^{1}H NMR spectrum of **14** (600 MHz, DMSO-d_6).

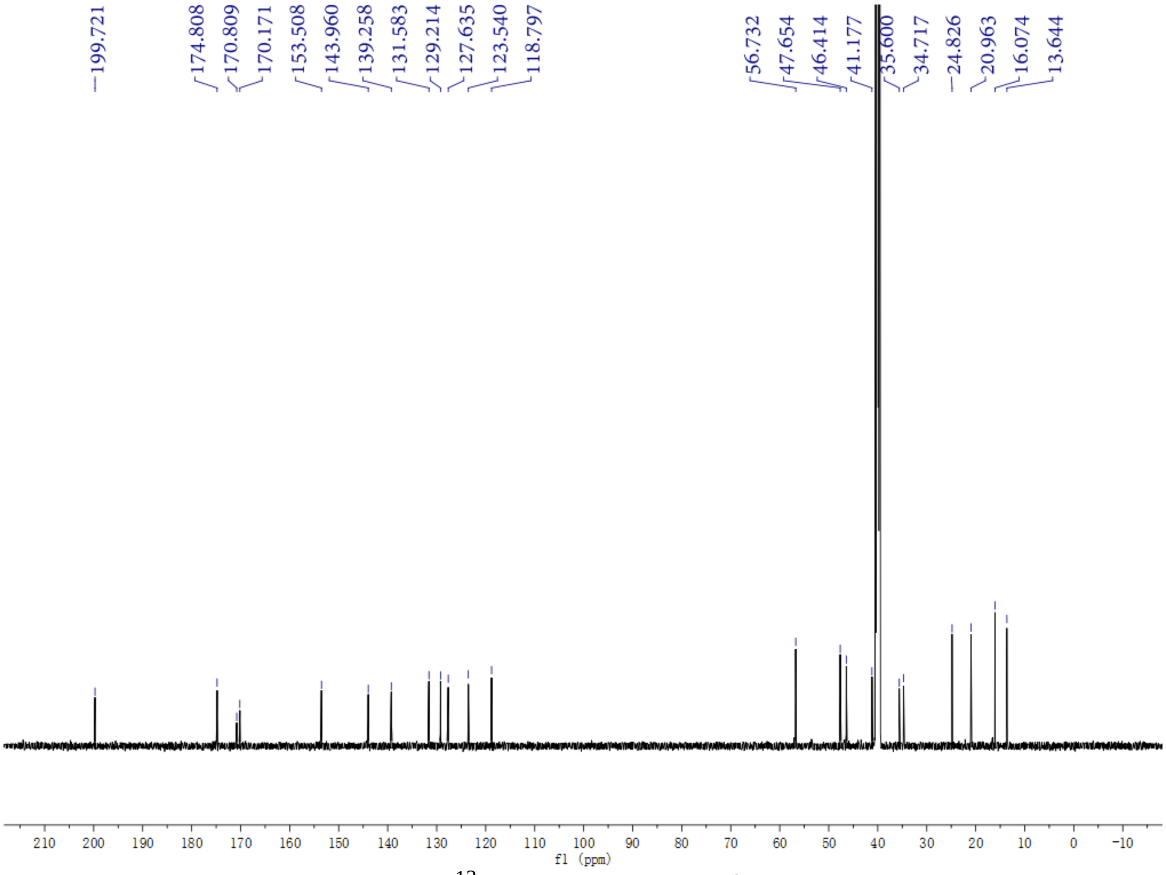

Supplementary Figure 49. ^{13}C NMR spectrum of **14** (150 MHz, DMSO-d_6).

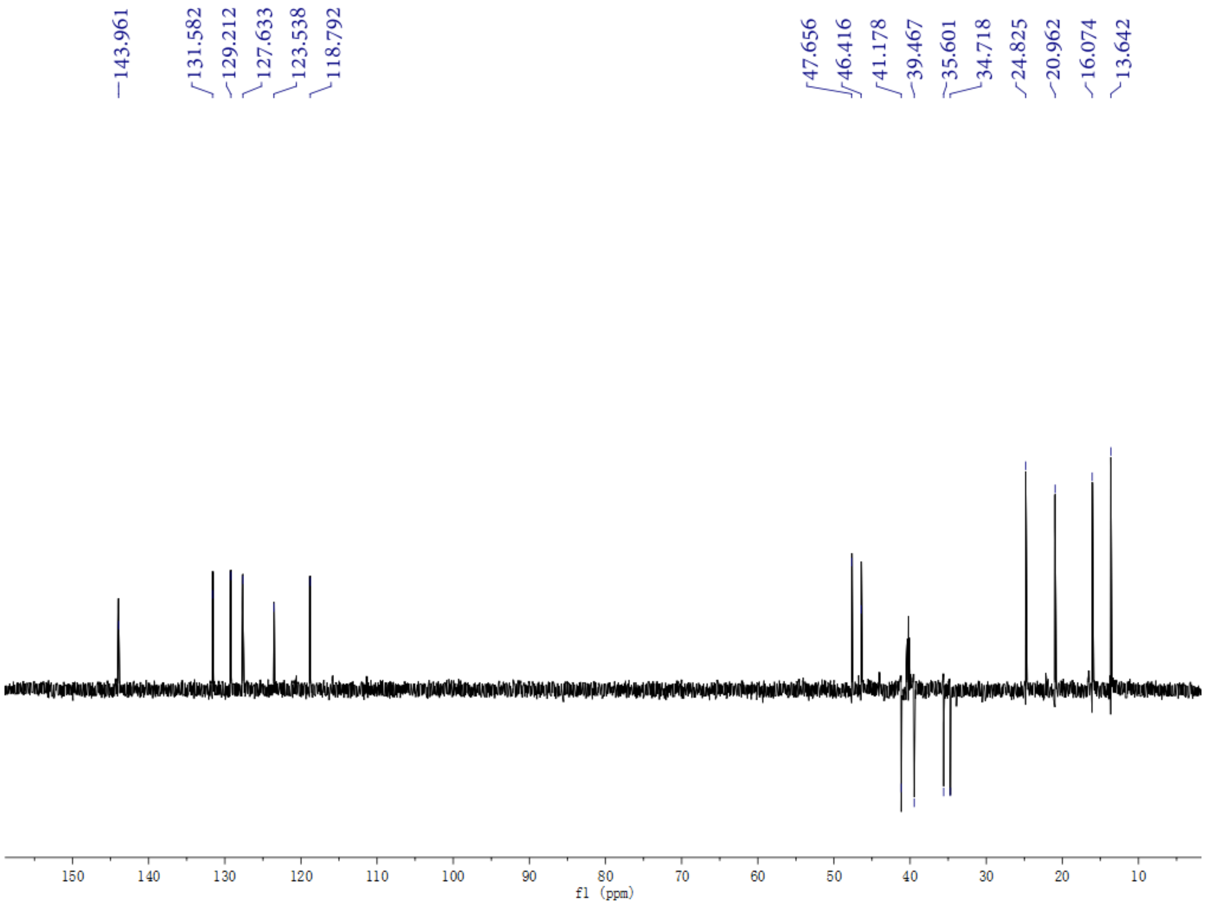

Supplementary Figure 50. DEPT-135 spectrum of **14** (DMSO-d_6).

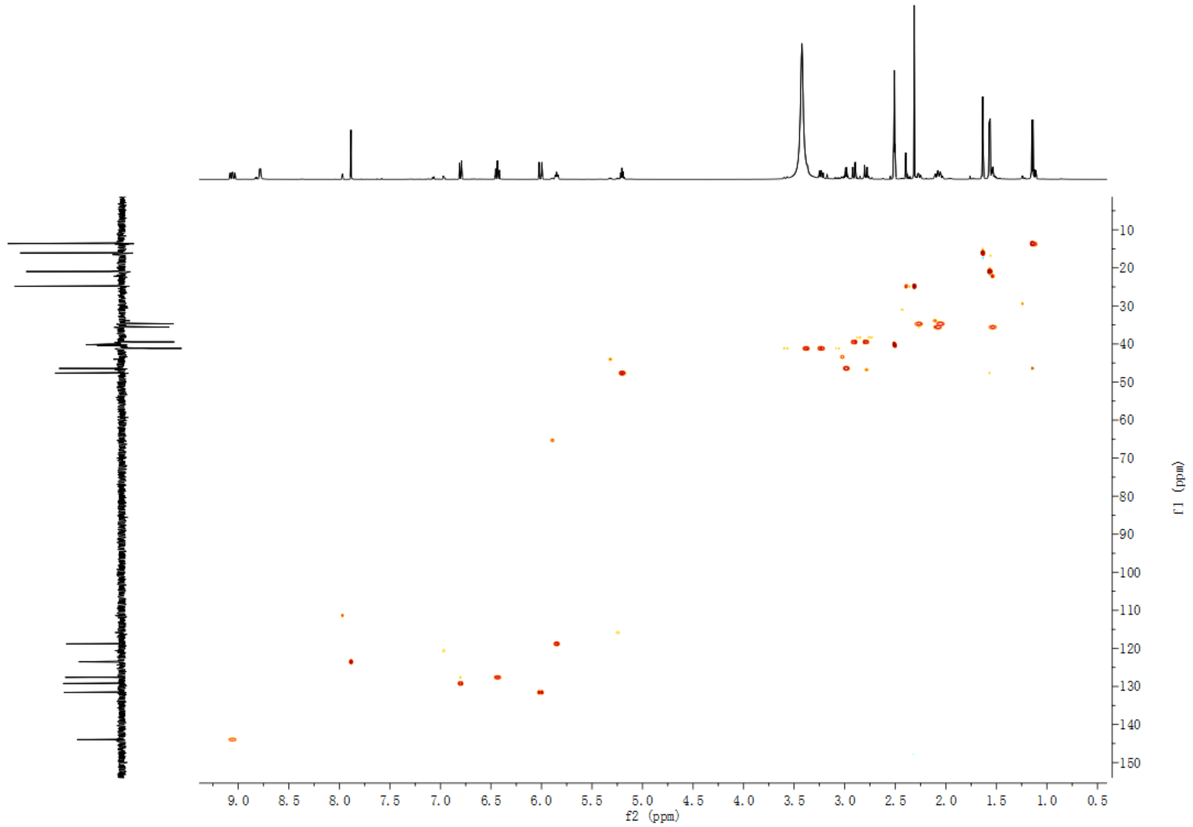

Supplementary Figure 51. HSQC spectrum of **14** (DMSO-d_6).

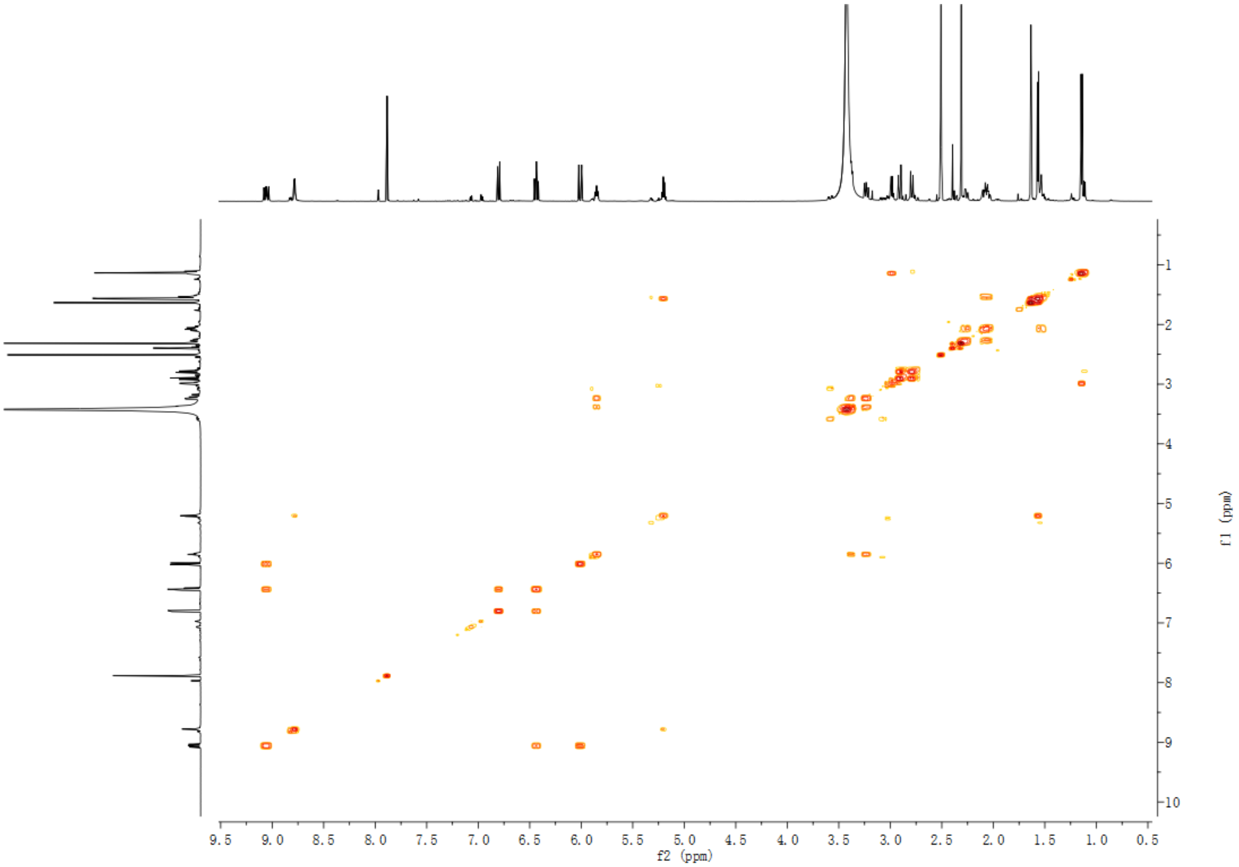

Supplementary Figure 52. ^{1}H-^{1}H COSY spectrum of **14** (DMSO-d_6).

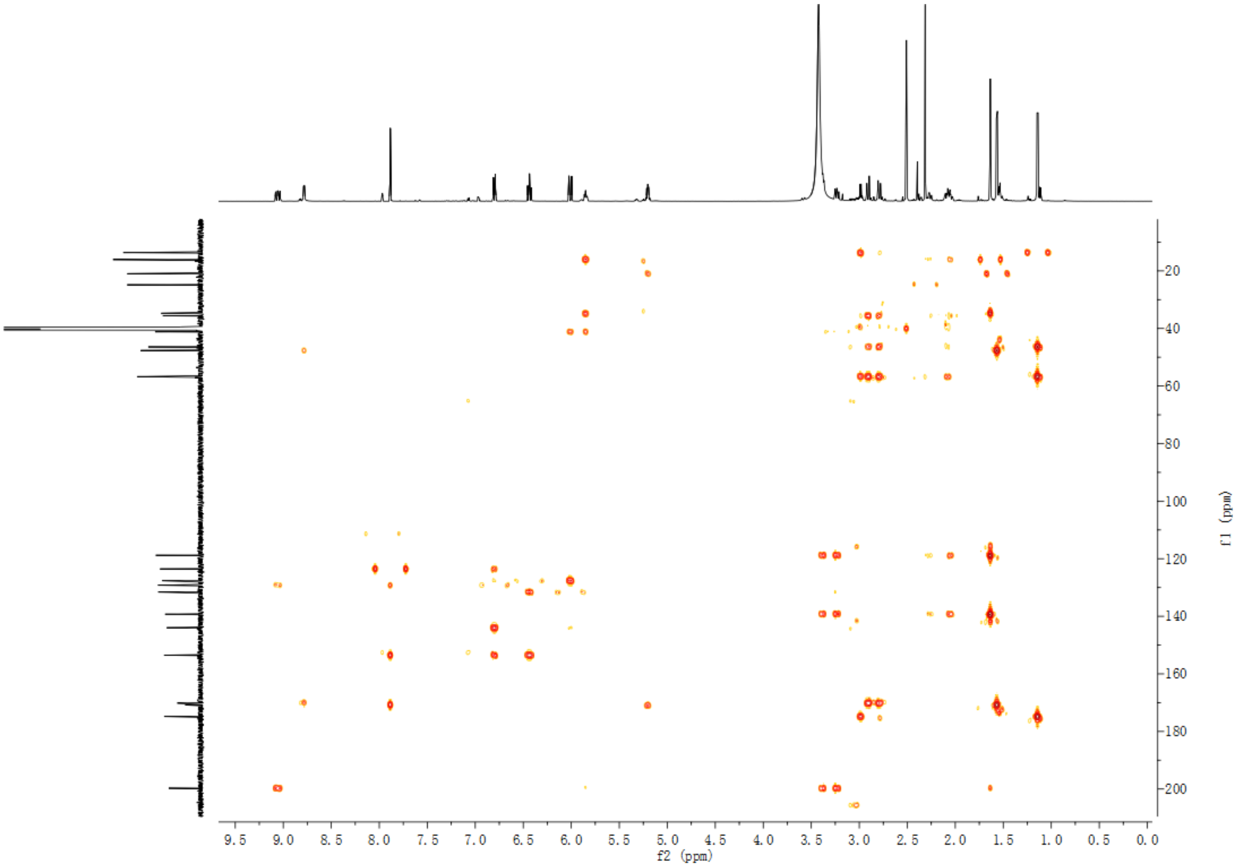

Supplementary Figure 53. HMBC spectrum of **14** (DMSO-d_6).

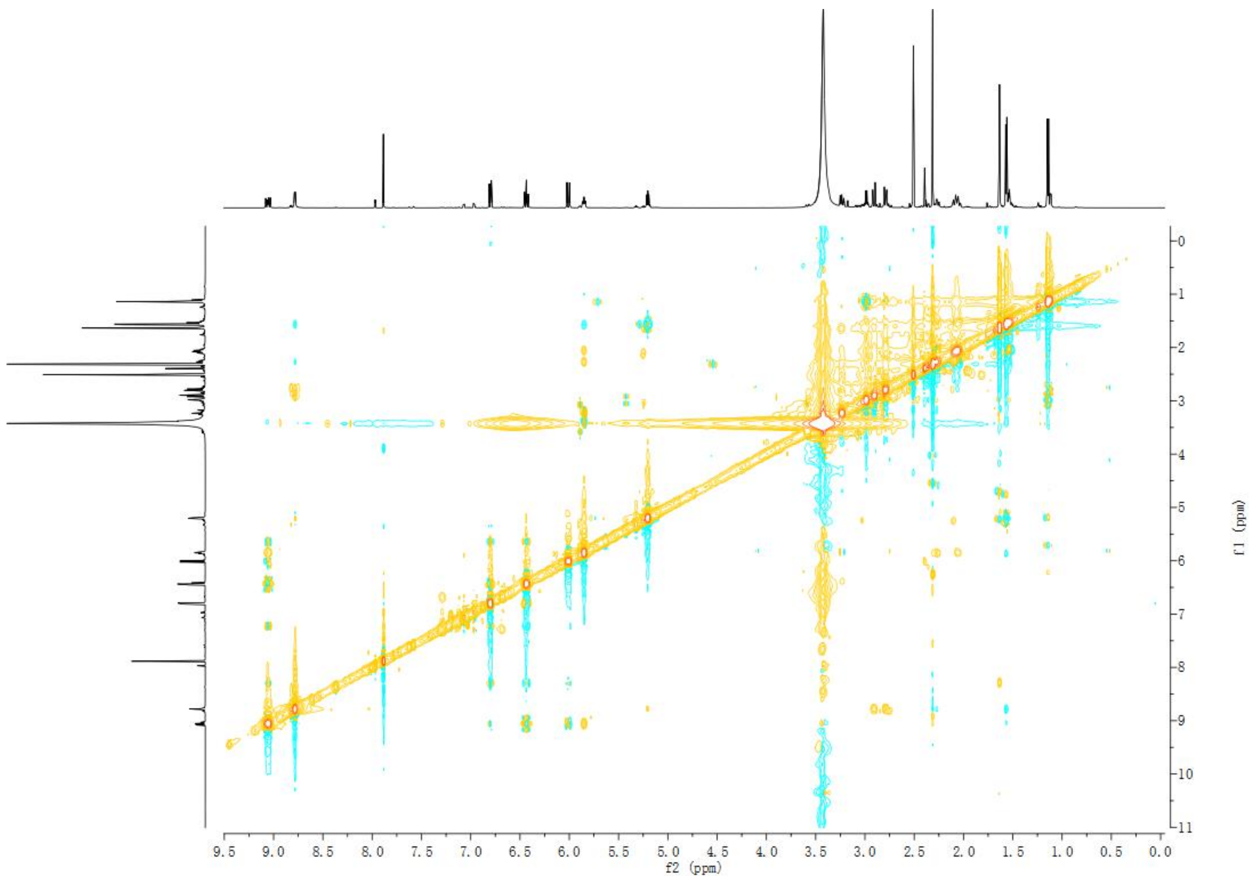

Supplementary Figure 54. ROESY spectrum of **14** (DMSO-d_6).

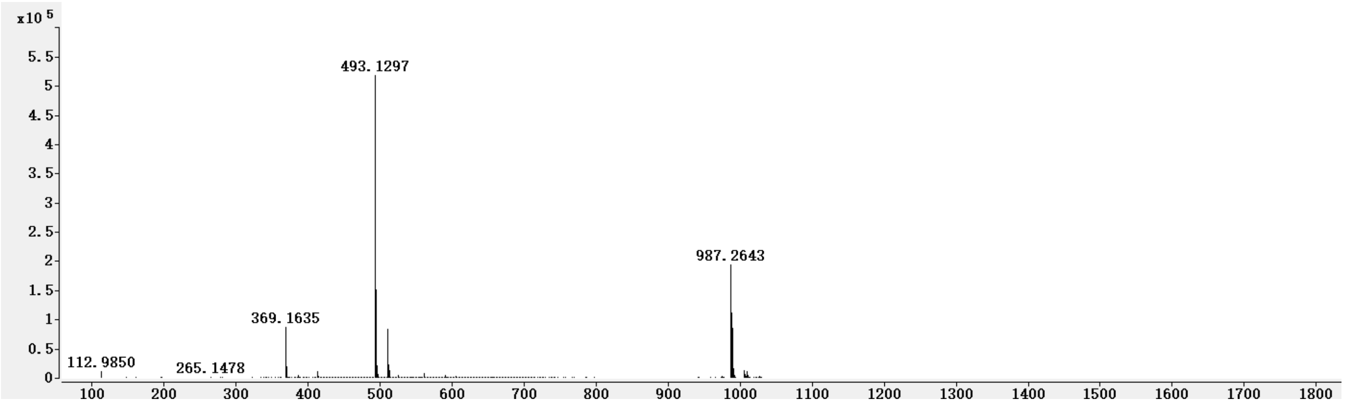

Supplementary Figure 55. HR-MS (ESI) spectrum of **14**.

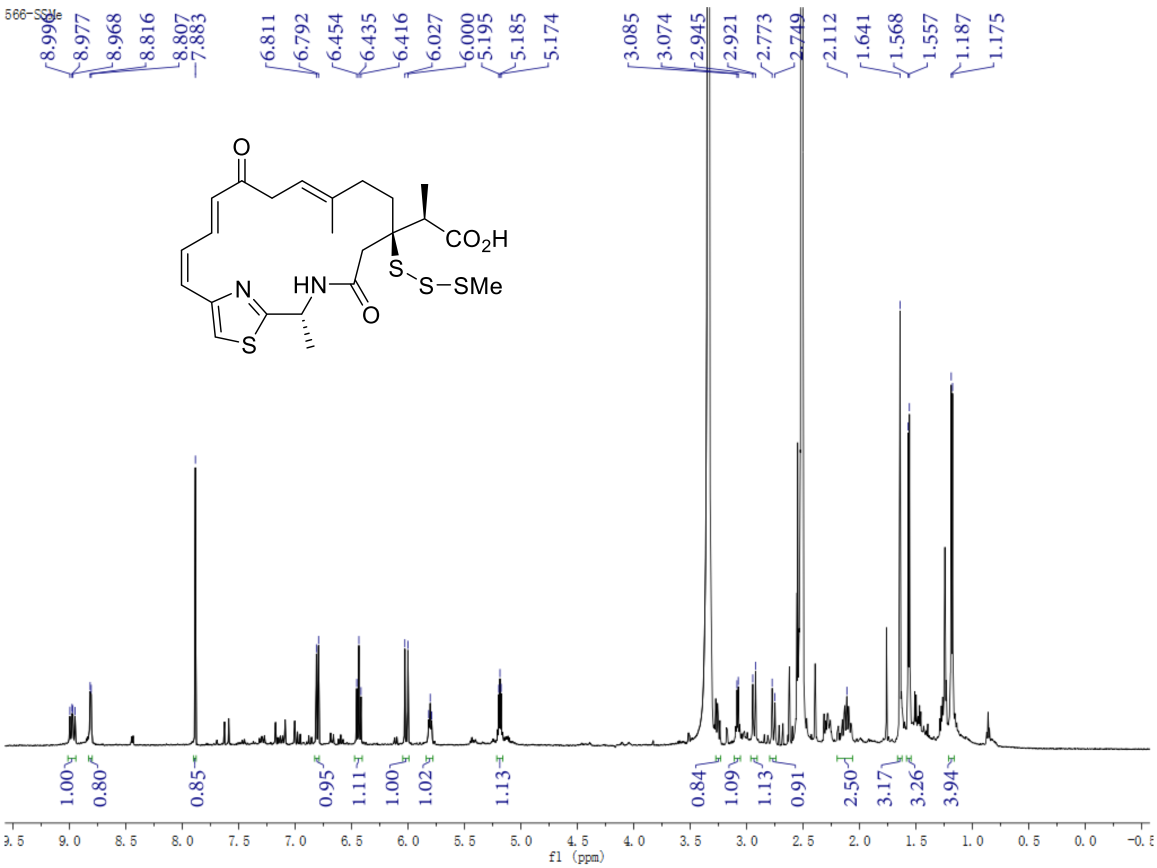

Supplementary Figure 56. ^{1}H NMR spectrum of **15** (600 MHz, DMSO-d_6).

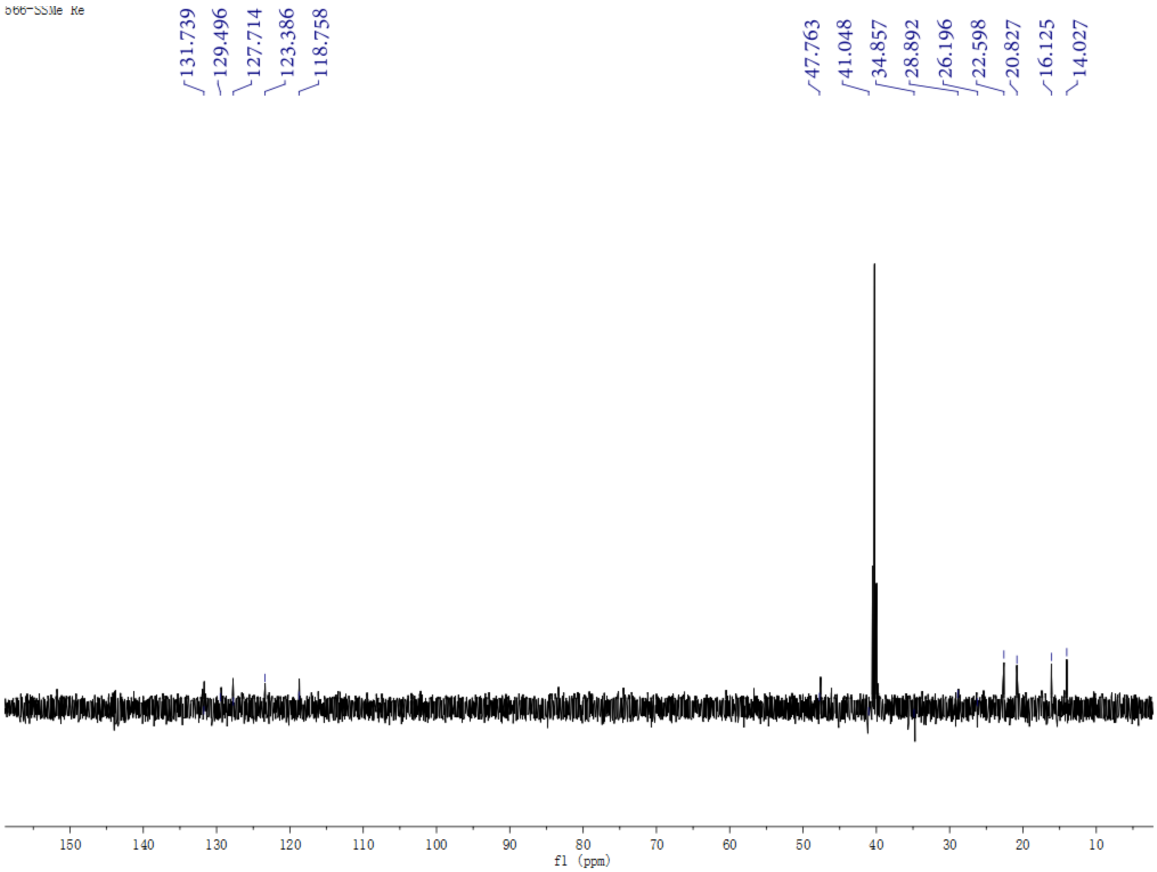

Supplementary Figure 57. DEPT-135 spectrum of **15** (DMSO-d_6).

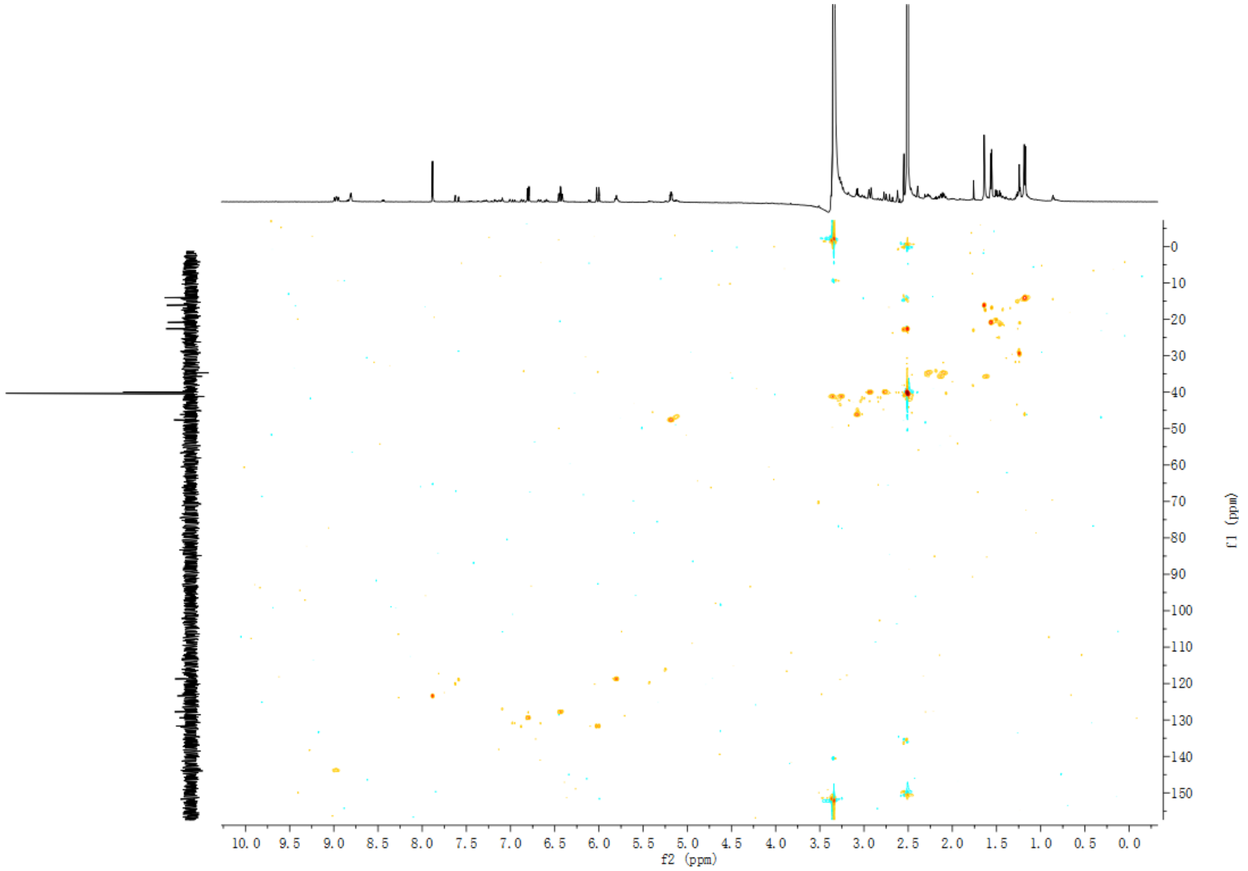

Supplementary Figure 58. HSQC spectrum of **15** (DMSO-d_6).

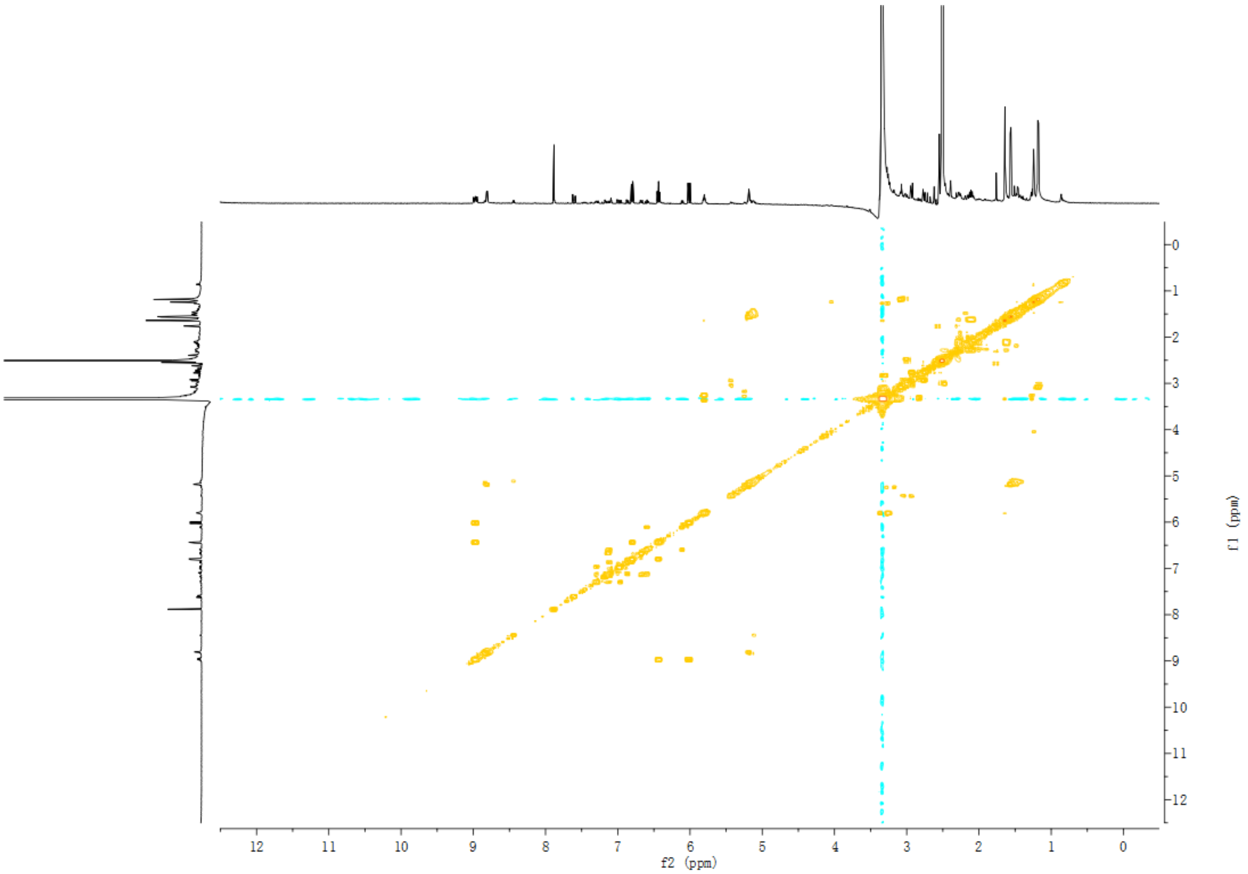

Supplementary Figure 59. ^1H-^1H COSY spectrum of **15** (DMSO-d_6).

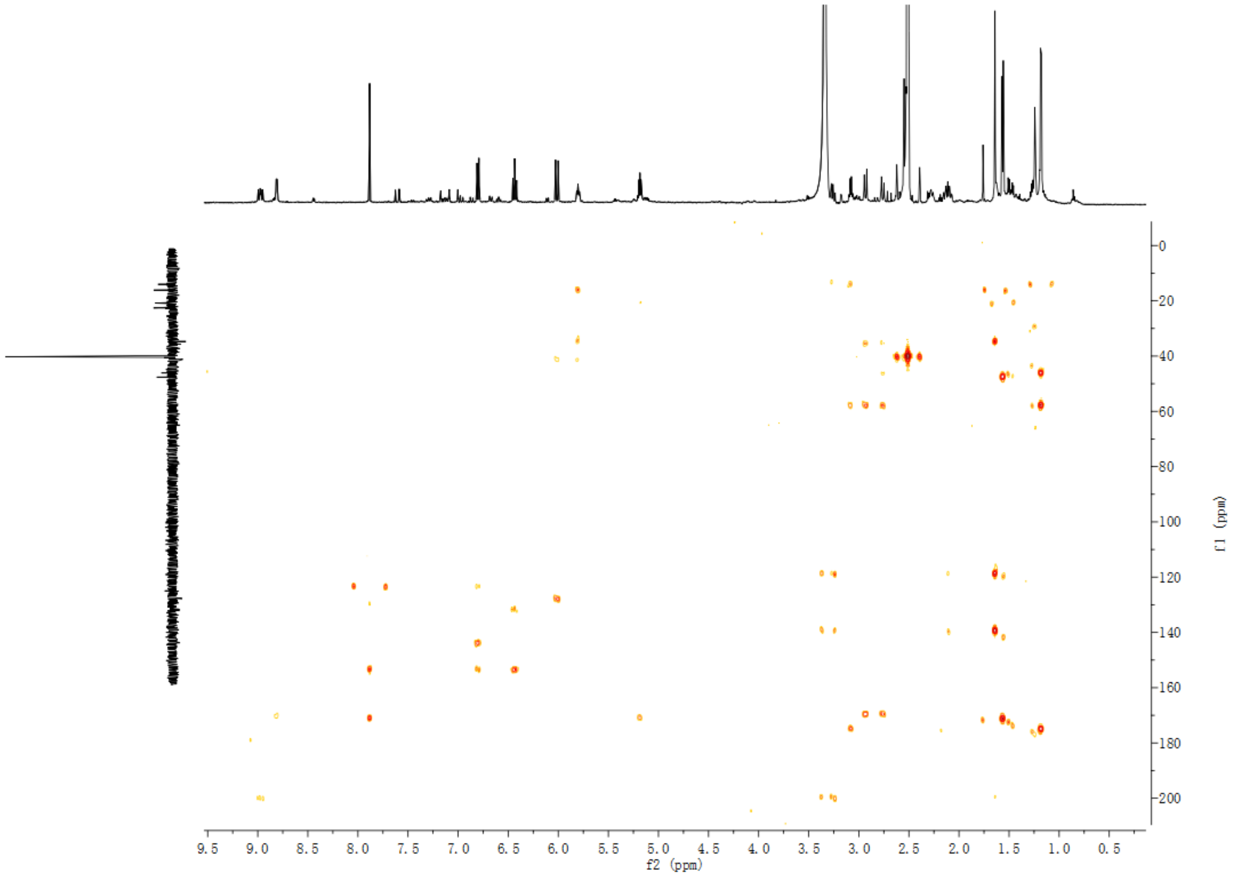

Supplementary Figure 60. HMBC spectrum of **15** (DMSO-d_6).

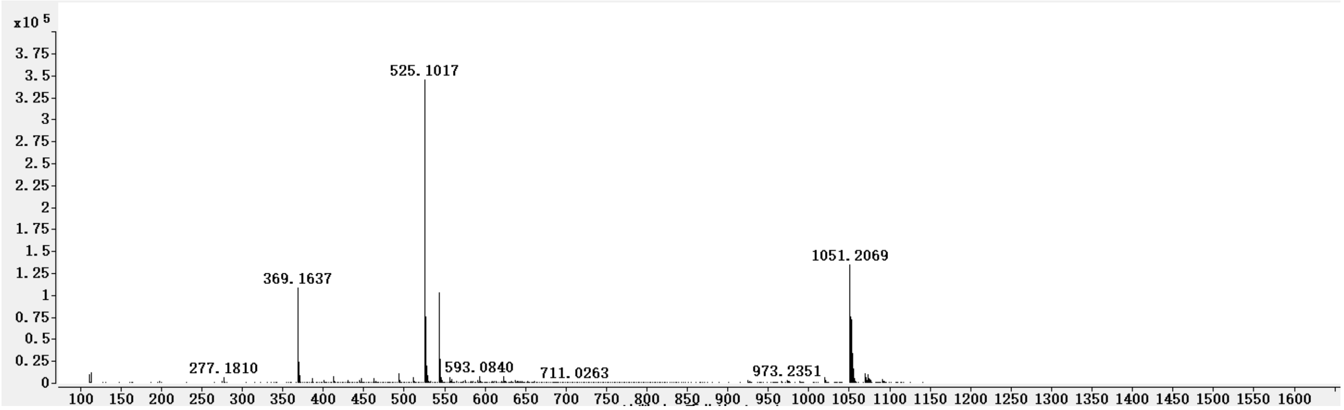

Supplementary Figure 61. HR-MS (ESI) spectrum of **15**.

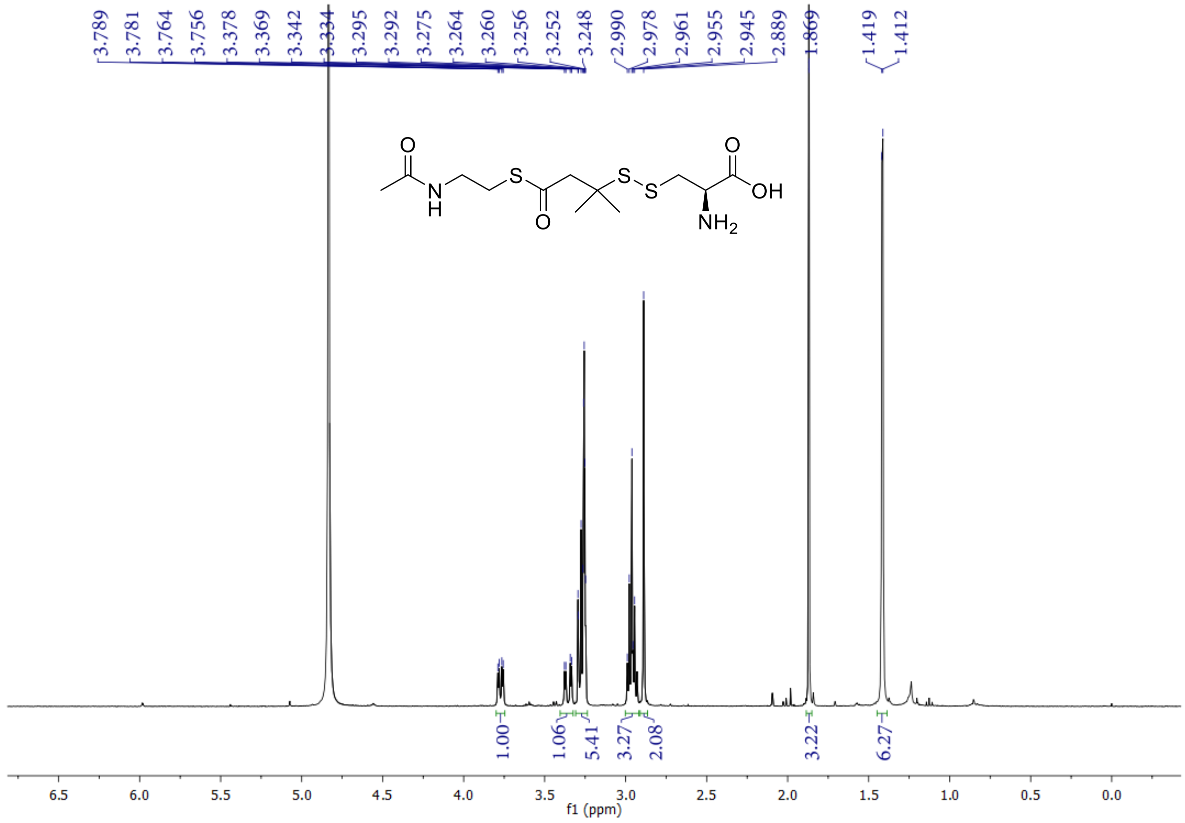

Supplementary Figure 62. ^{1}H NMR spectrum of **17** (600 MHz, methanol-d_4).

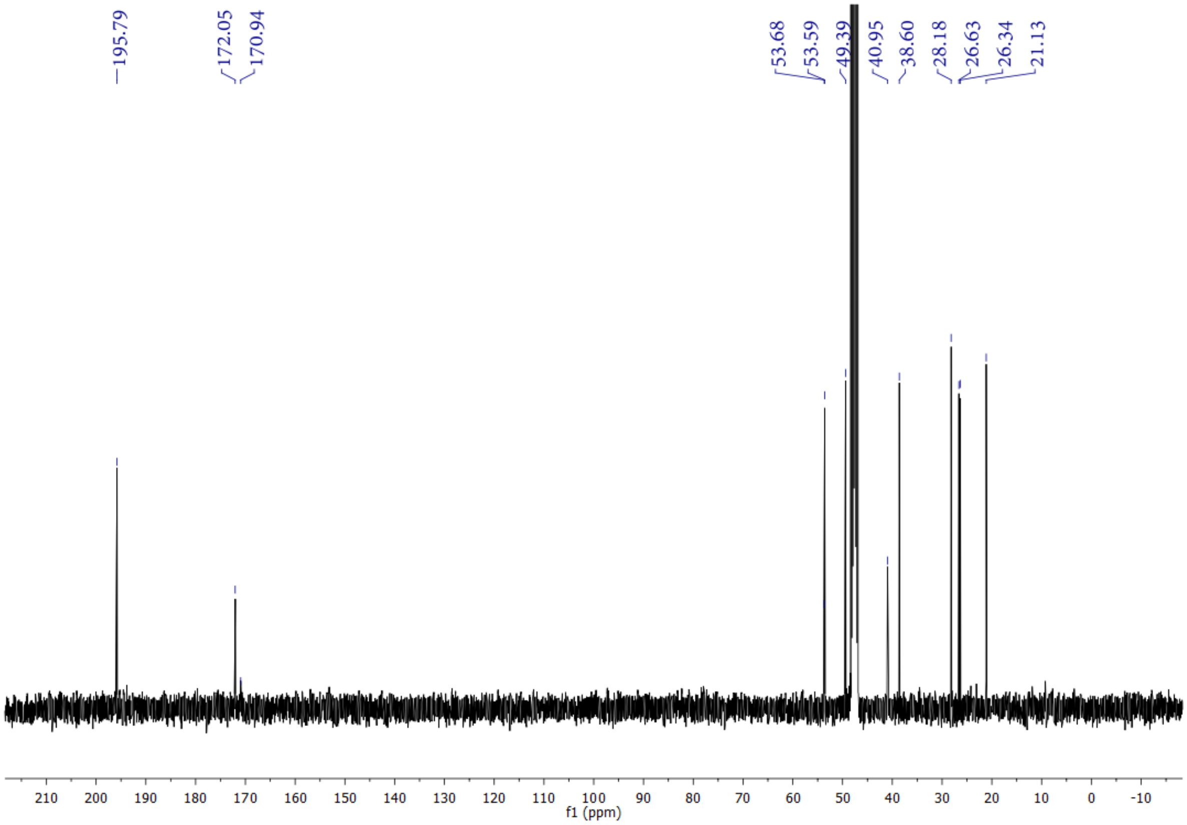

Supplementary Figure 63. ^{13}C NMR spectrum of **17** (150 MHz, methanol-d_4).

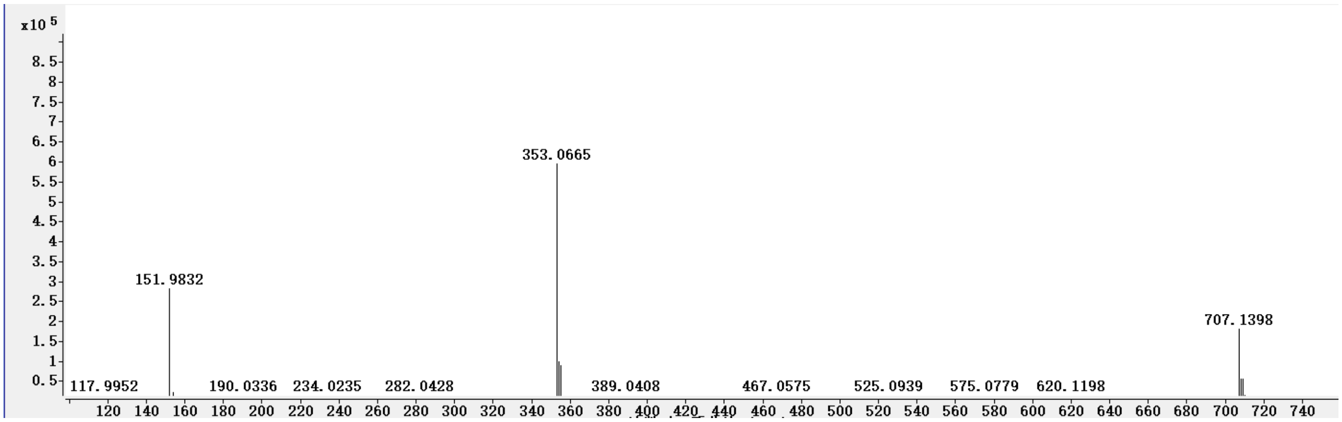

Supplementary Figure 64. HR-MS (ESI) spectrum of **17**.

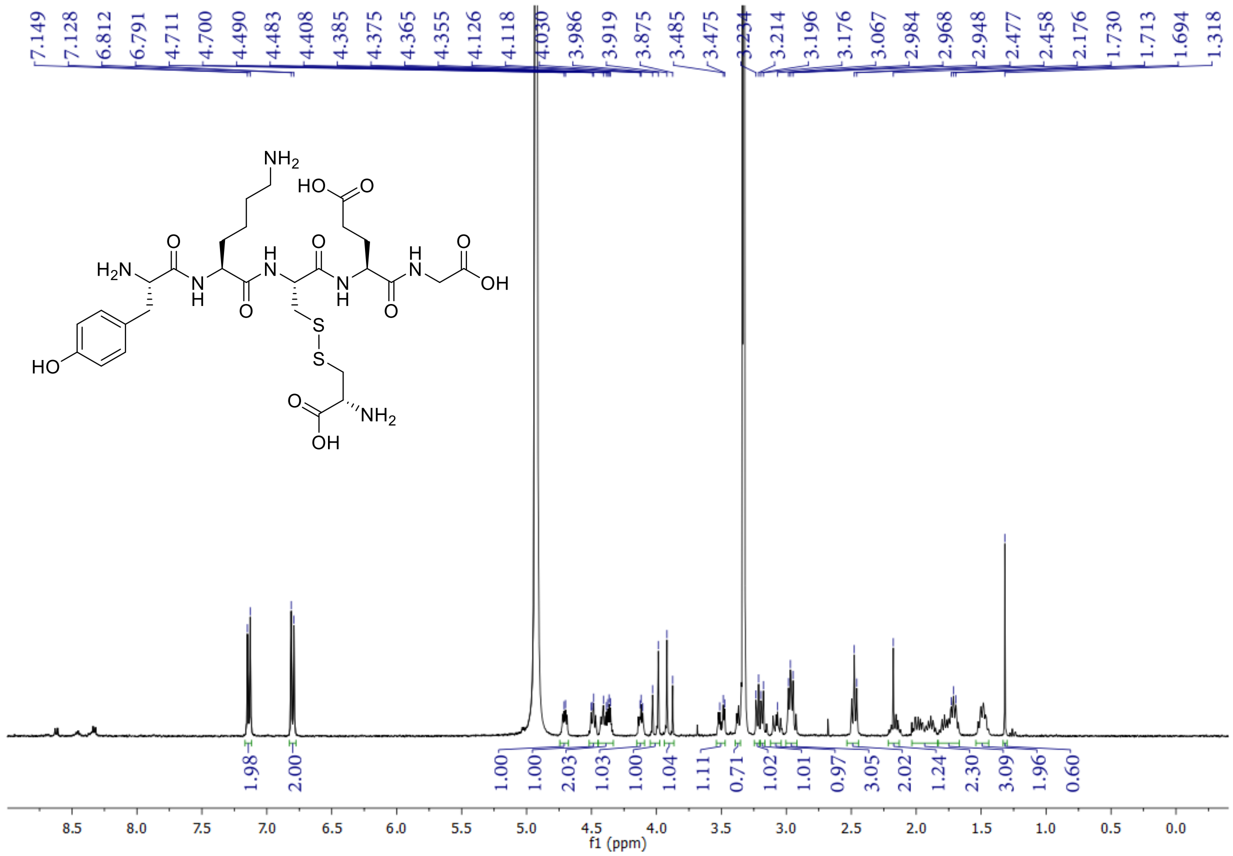

Supplementary Figure 65. ^{1}H NMR spectrum of **19** (600 MHz, methanol-d_4).

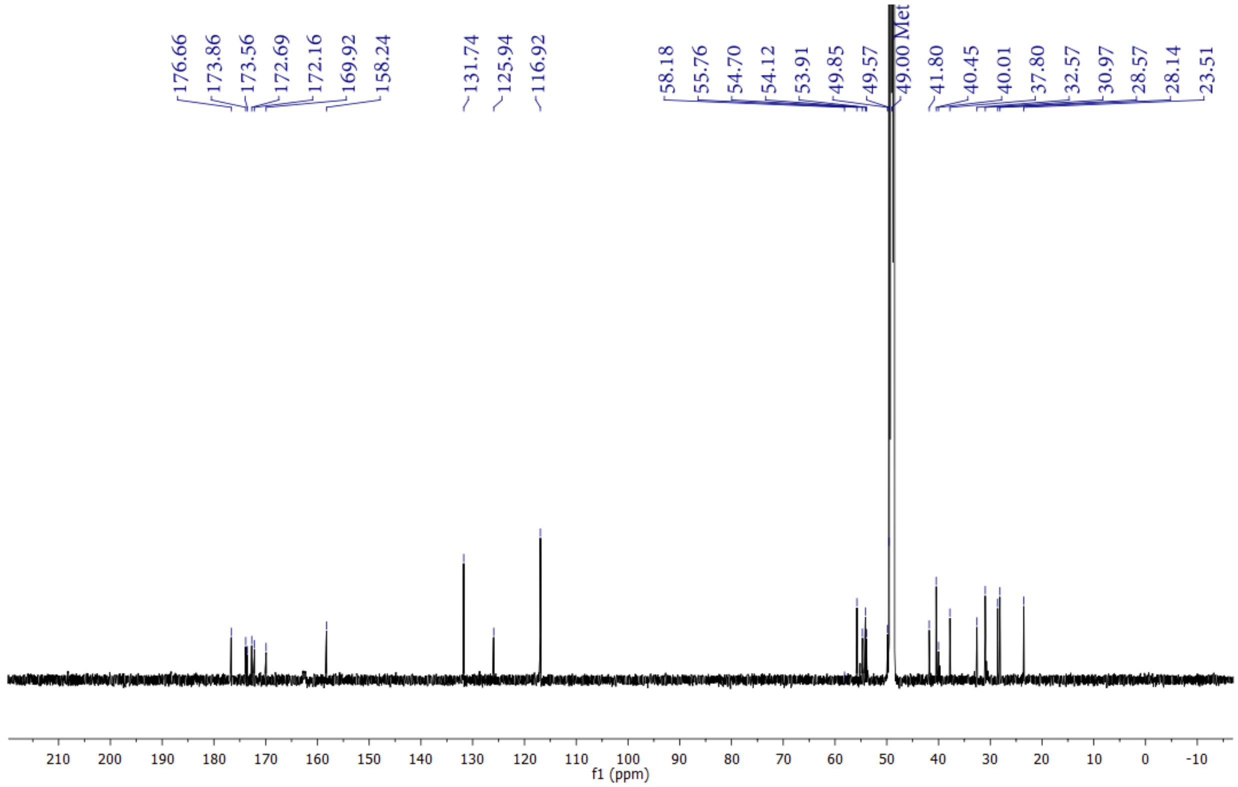

Supplementary Figure 66. ^{13}C NMR spectrum of **19** (150 MHz, methanol-d_4).

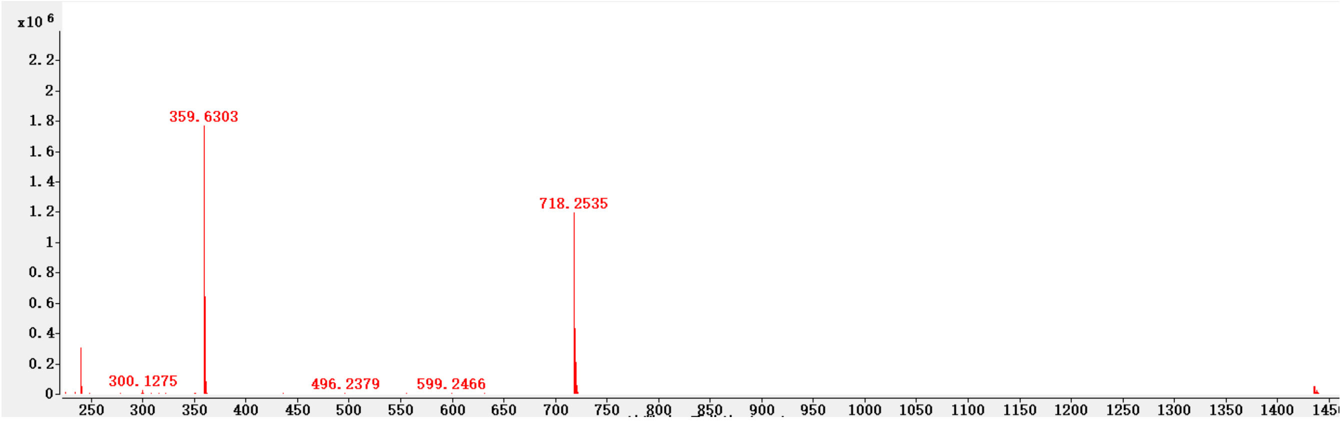

Supplementary Figure 67. HR-MS (ESI) spectrum of **19**.

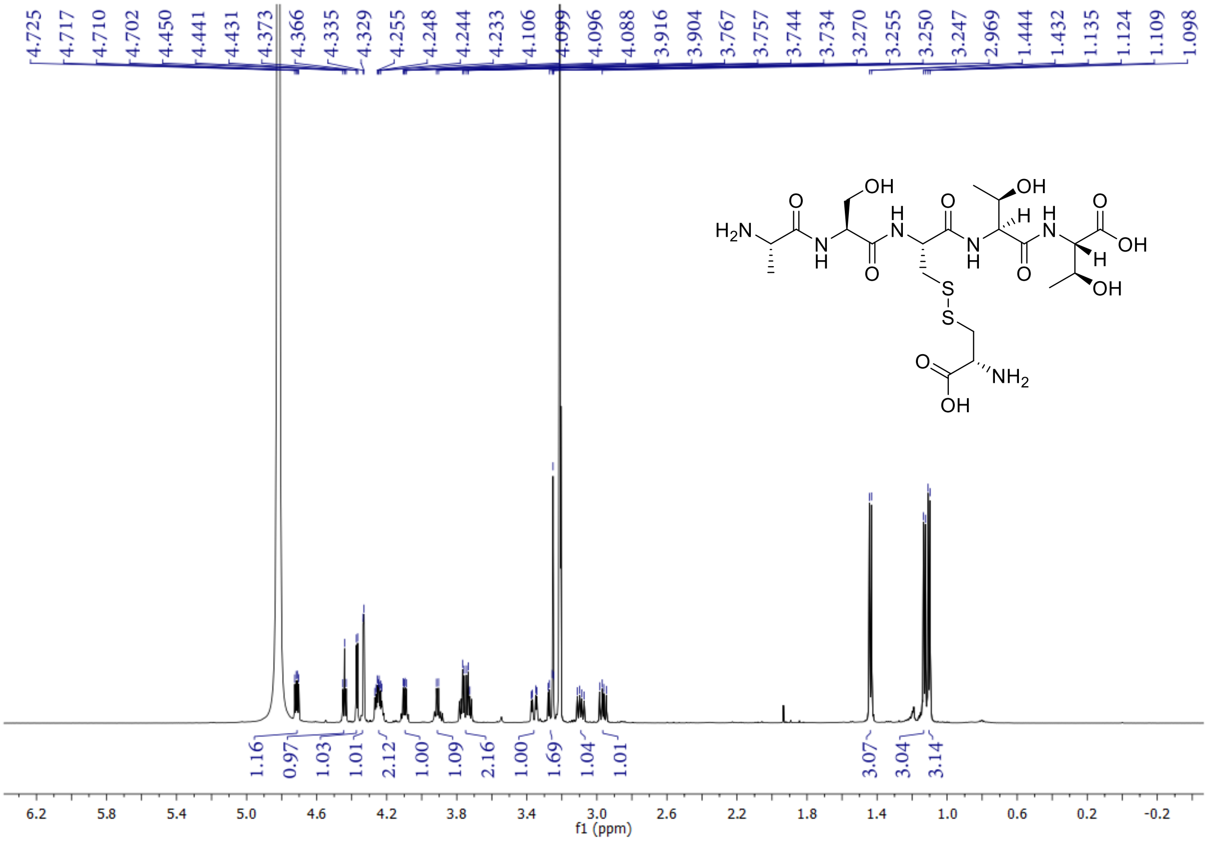

Supplementary Figure 68. ^{1}H NMR spectrum of **21** (600 MHz, methanol-d_4).

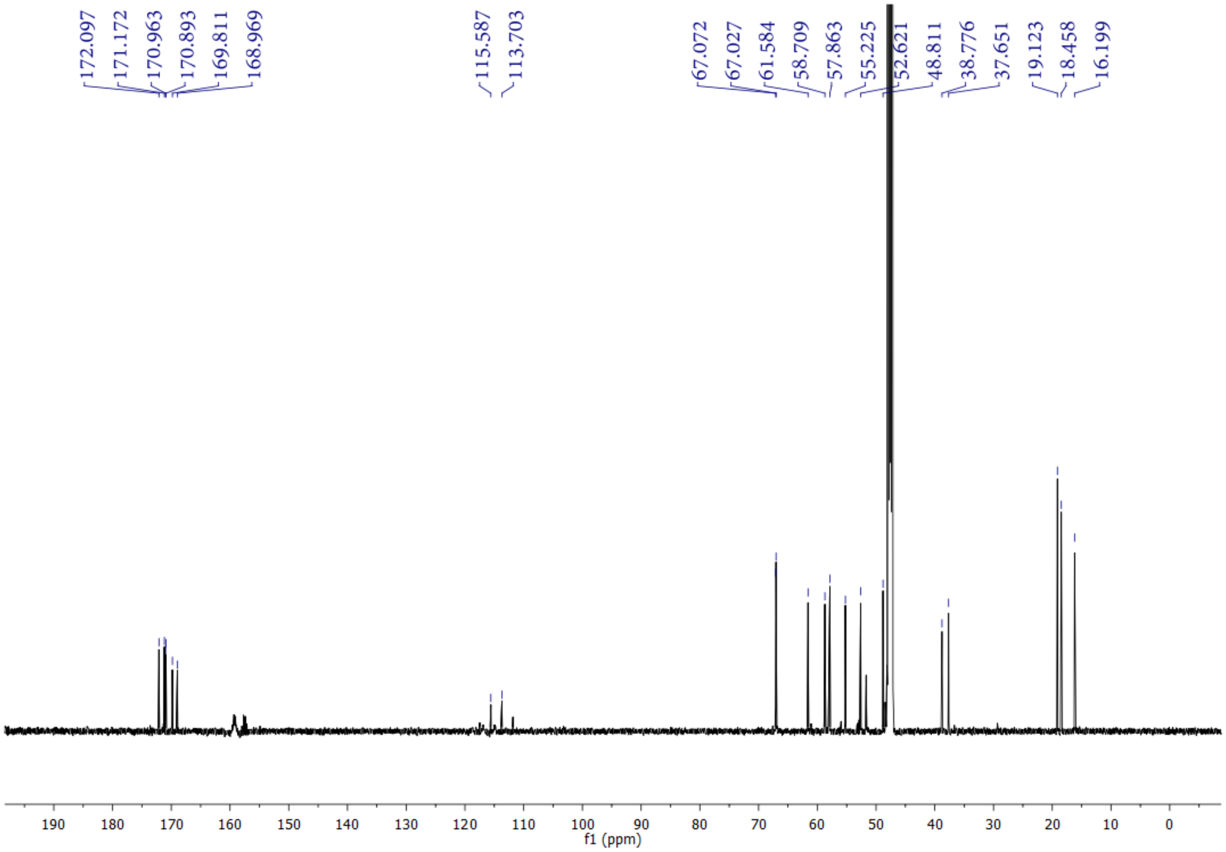

Supplementary Figure 69 ^{13}C NMR spectrum of **21** (150 MHz, methanol-d_4).

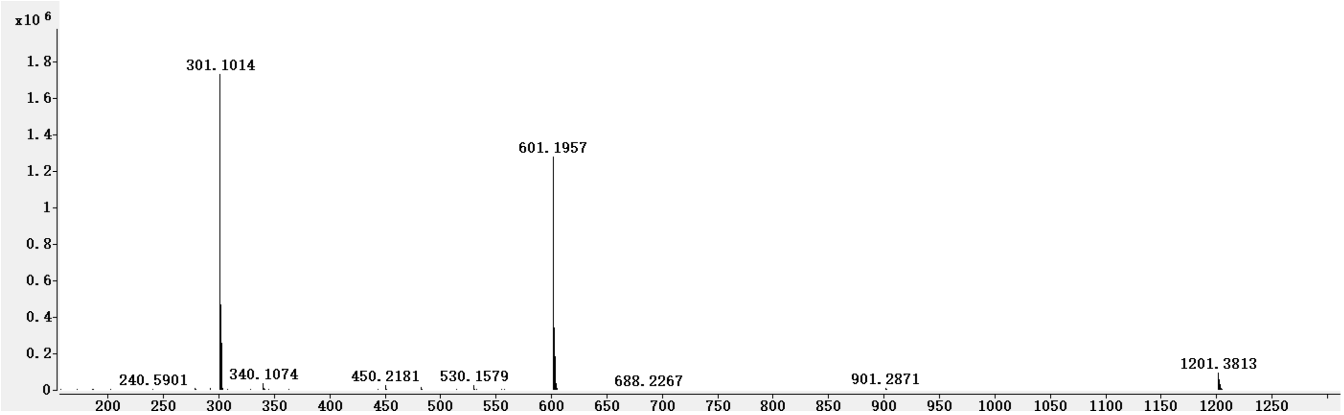

Supplementary Figure 70. HR-MS (ESI) spectrum of **21**.

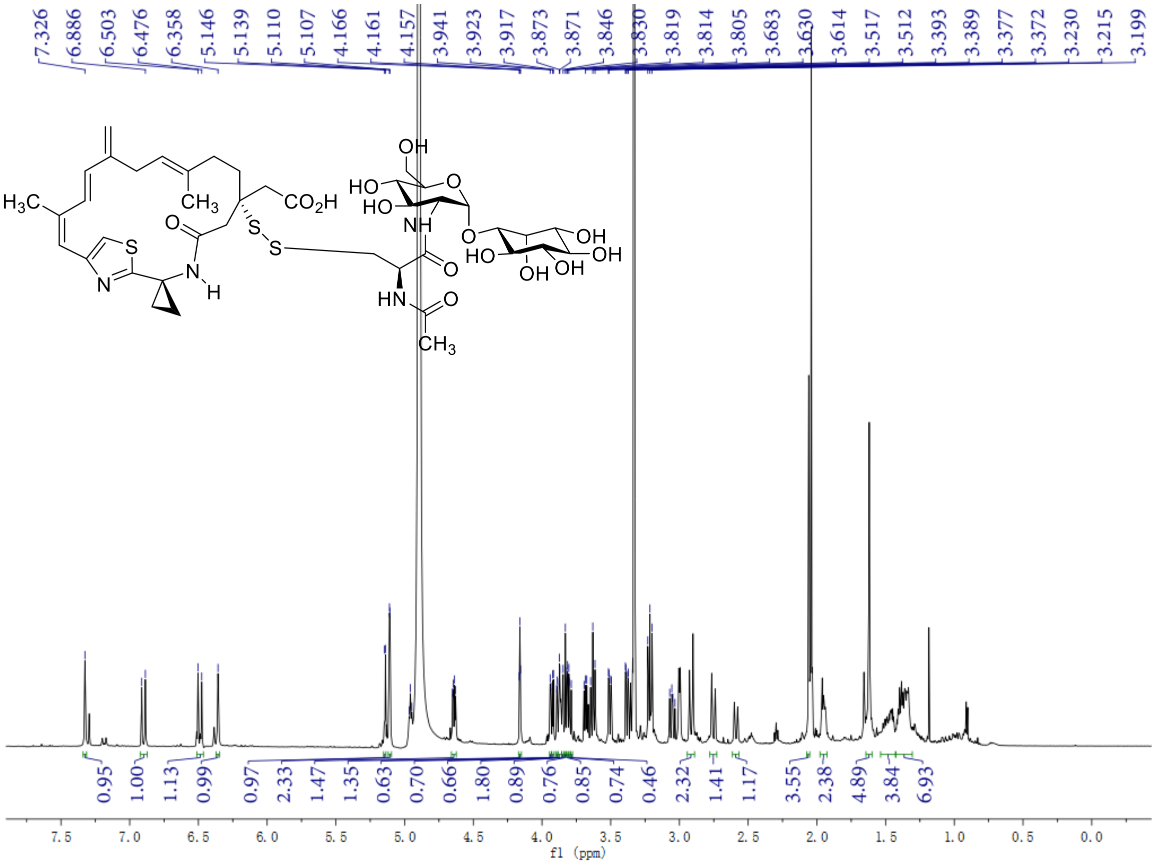

Supplementary Figure 71. ^{1}H NMR spectrum of **S1** (600 MHz, methanol-d_4).

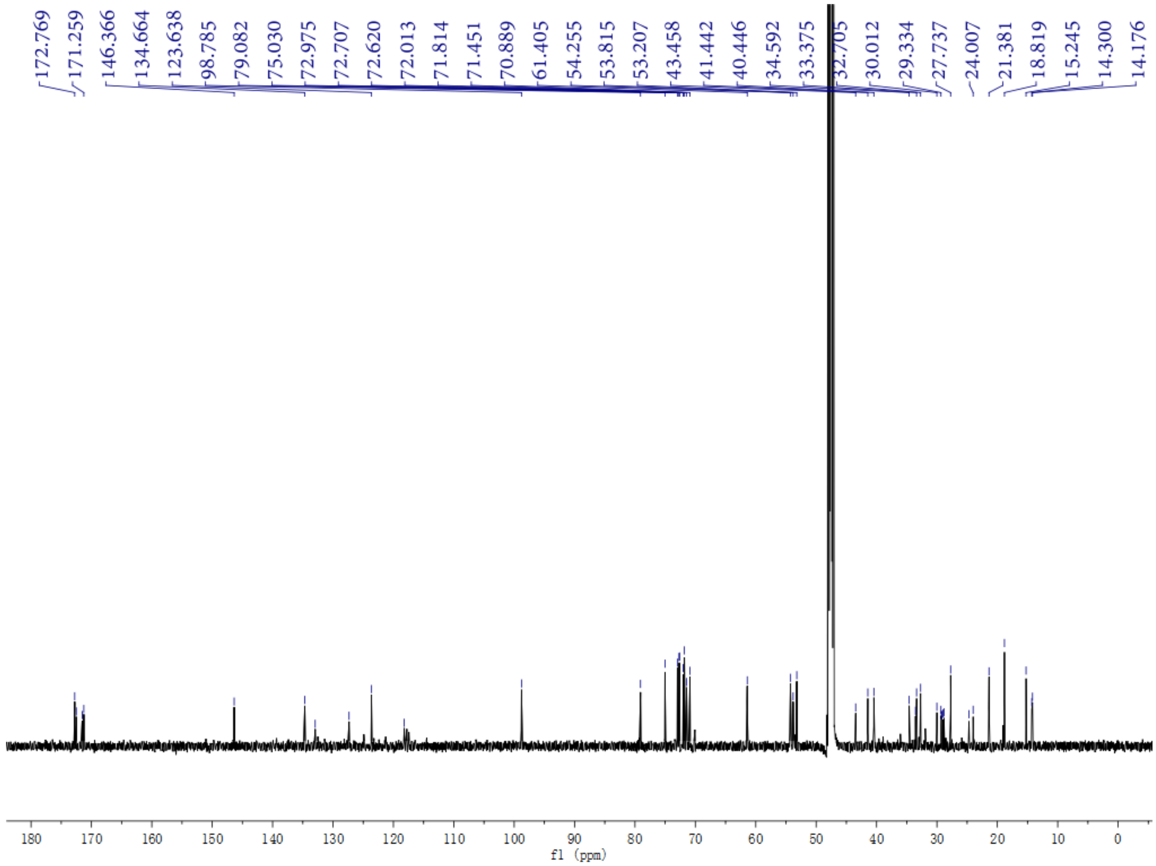

Supplementary Figure 72. ^{13}C NMR spectrum of **S1** (150 MHz, methanol-d_4).

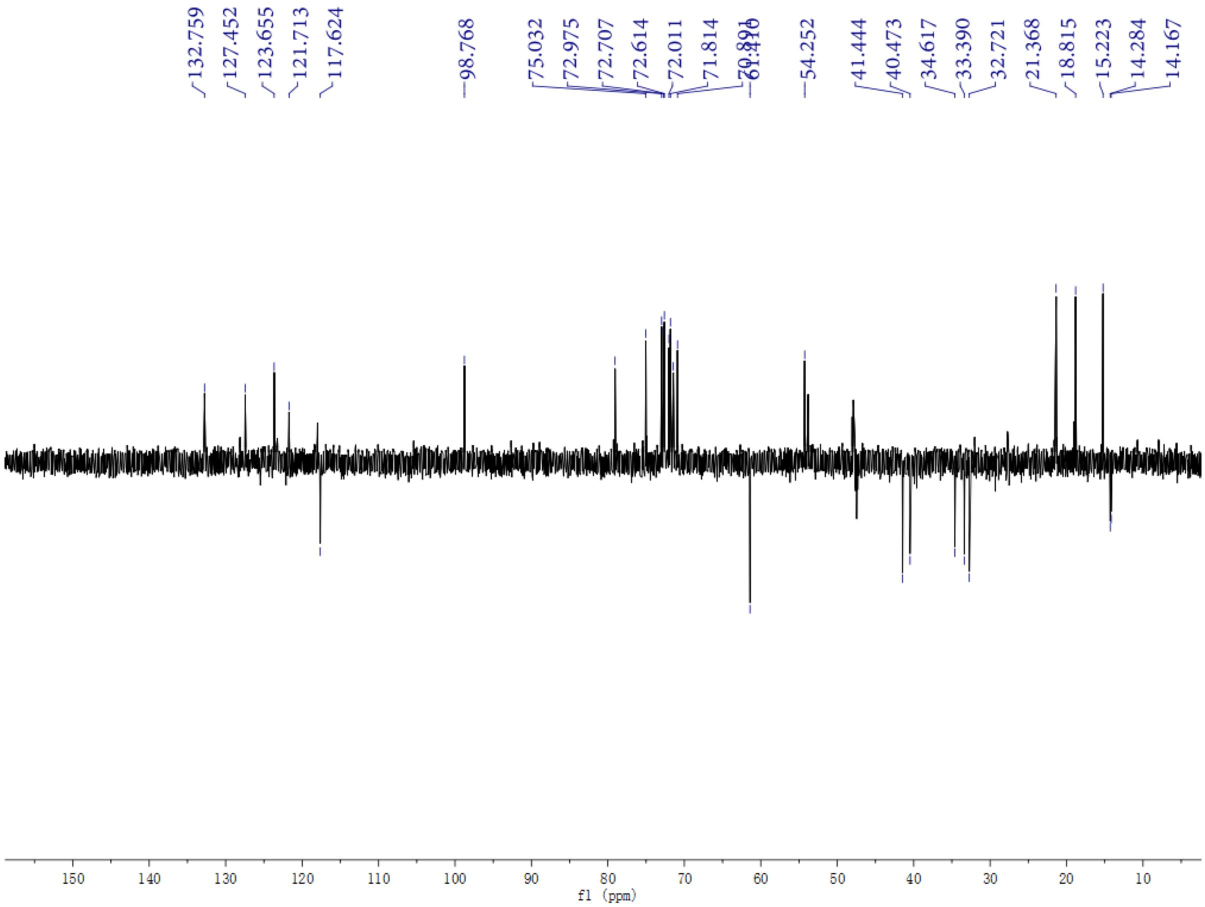

Supplementary Figure 73. DEPT-135 spectrum of **S1** (methanol-d_4).

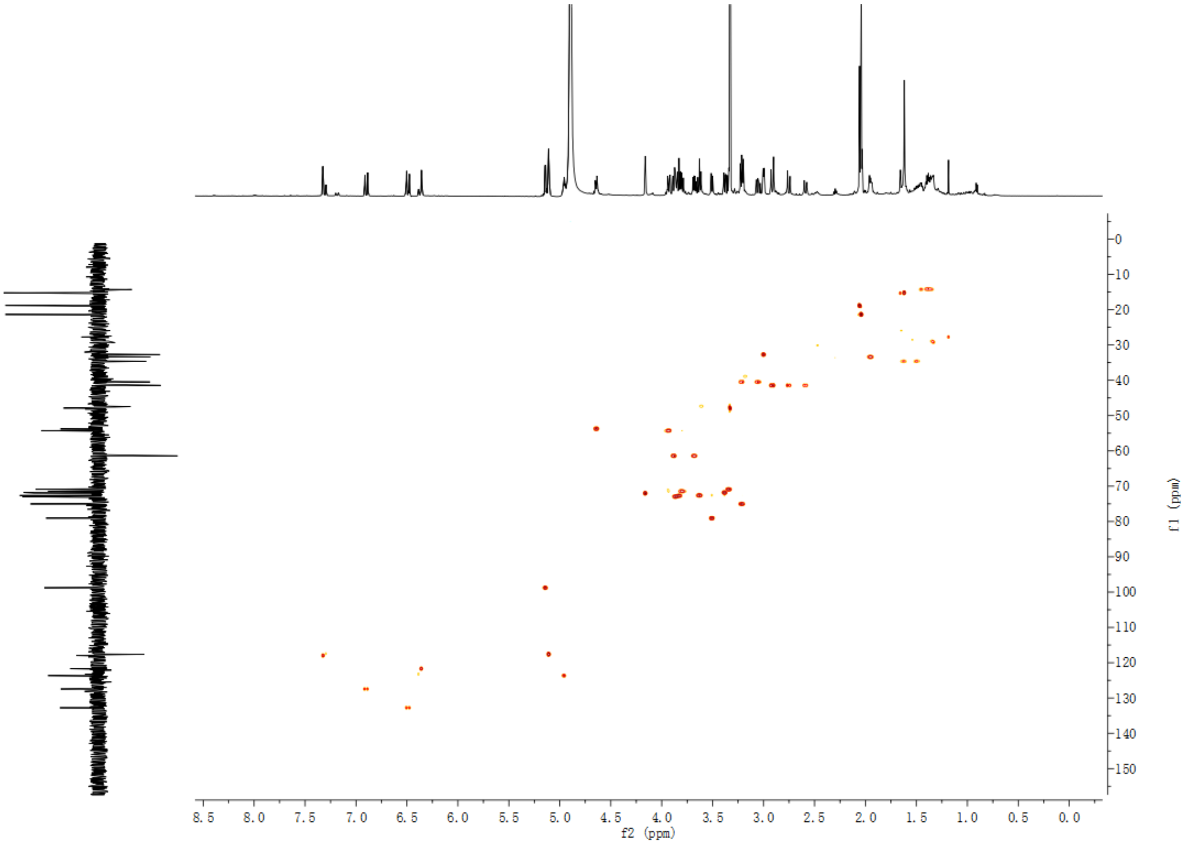

Supplementary Figure 74. HSQC spectrum of **S1** (methanol-d_4).

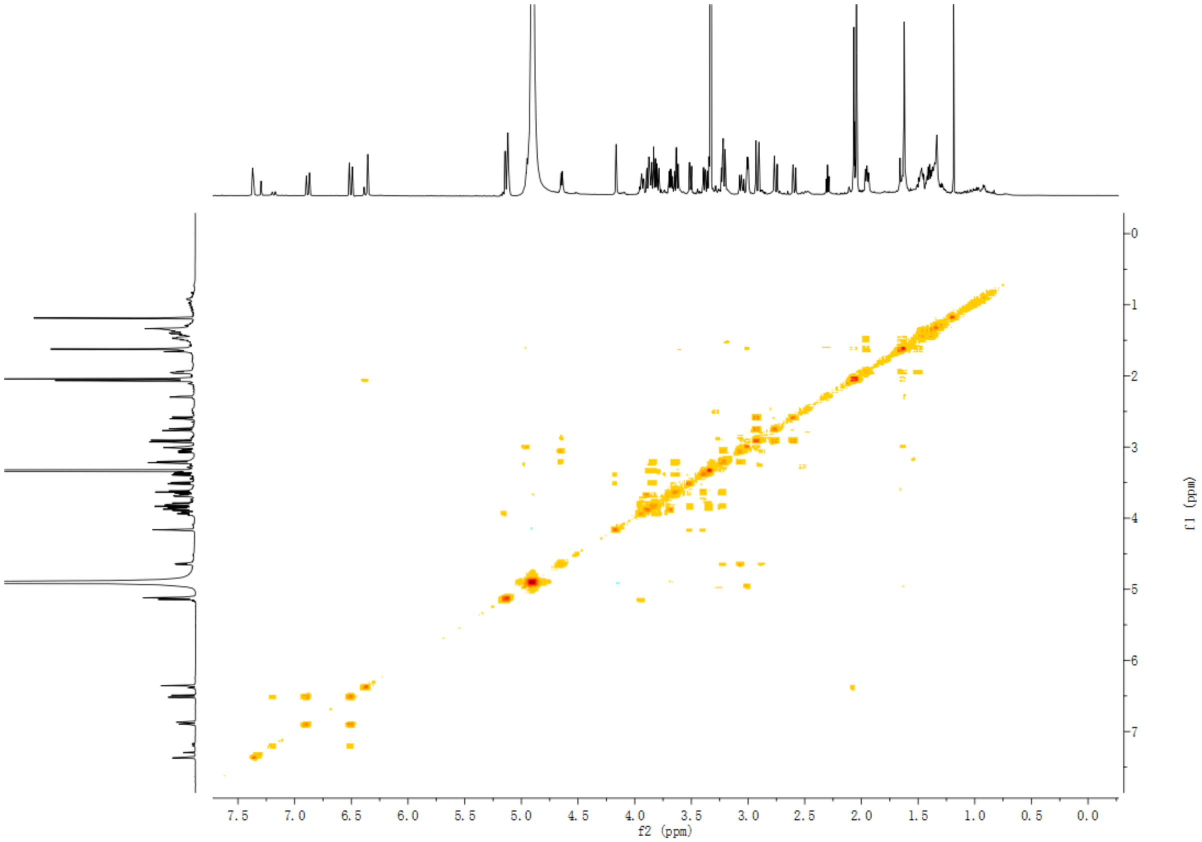

Supplementary Figure 75. ^{1}H-^{1}H COSY spectrum of **S1** (methanol-d_4).

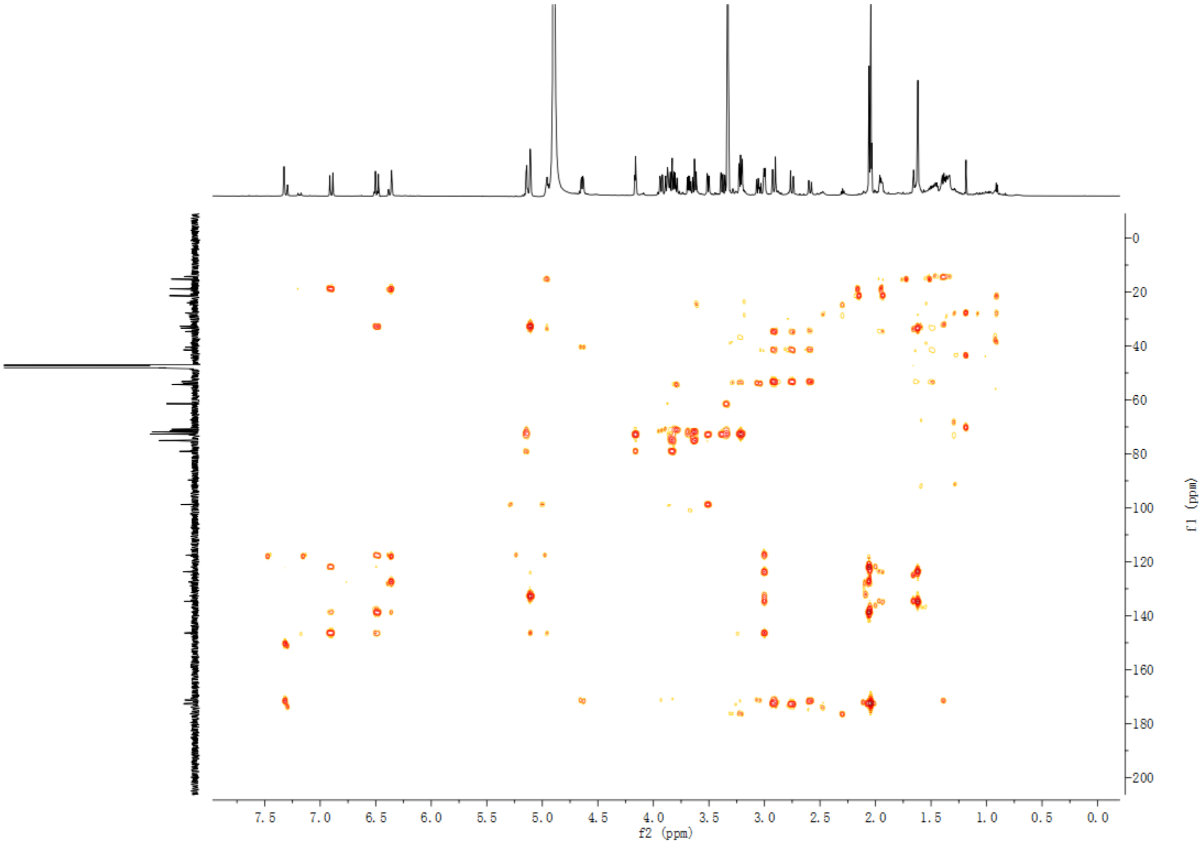

Supplementary Figure 76. HMBC spectrum of **S1** (methanol-d_4).

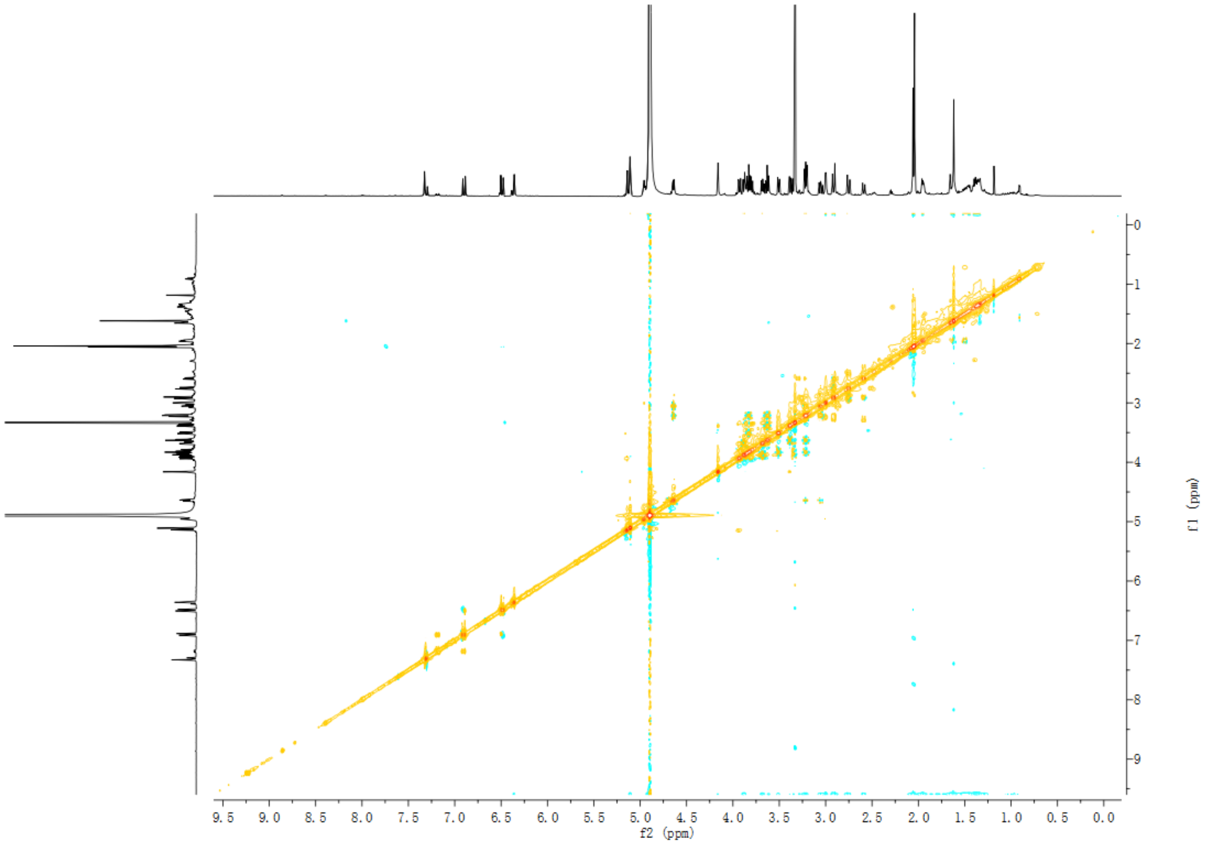

Supplementary Figure 77. ROESY spectrum of **S1** (methanol-d_4).

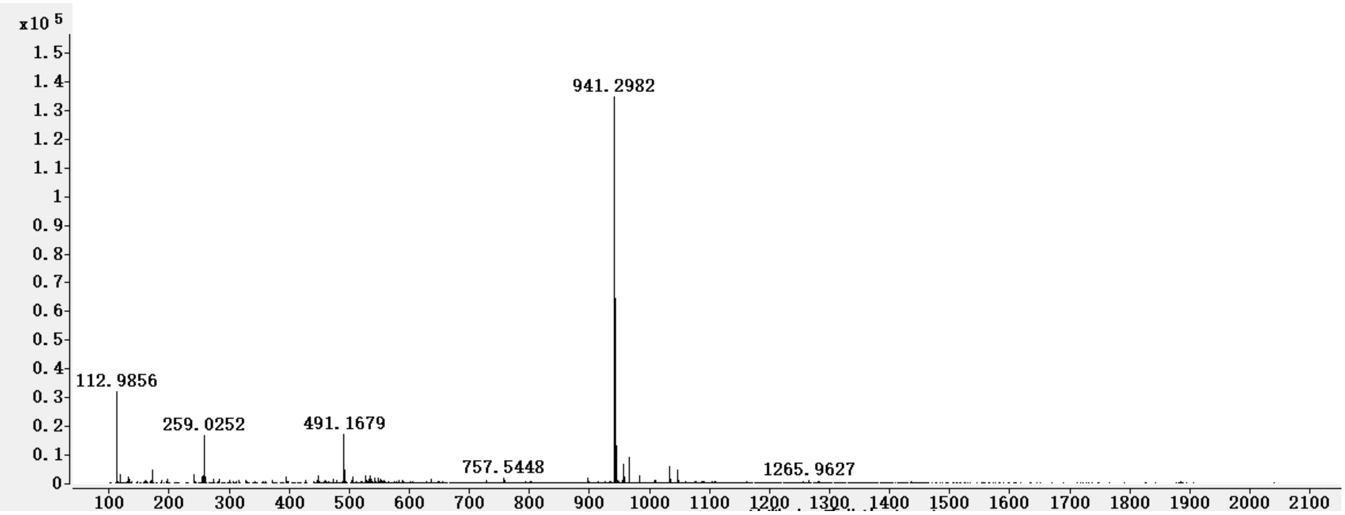

Supplementary Figure 78. HR-MS (ESI) spectrum of **S1**.

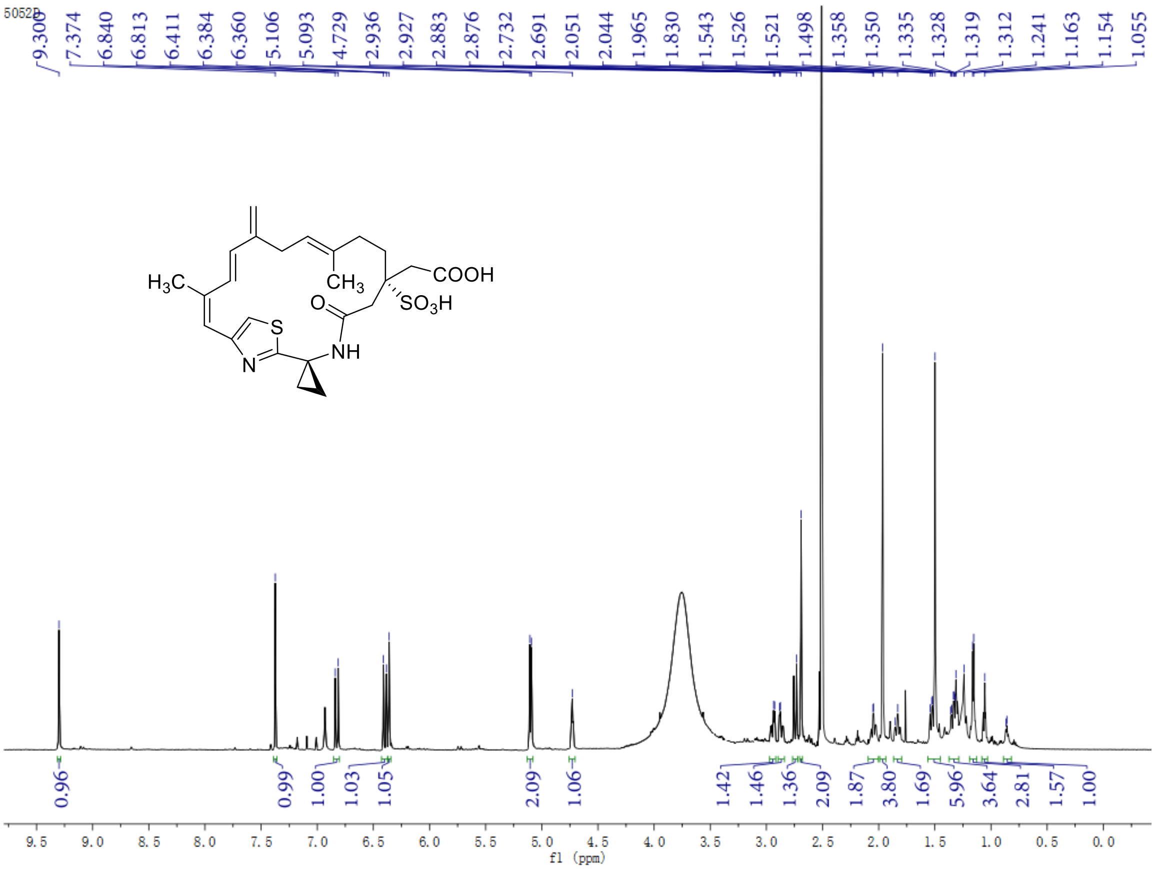

Supplementary Figure 79. ^{1}H NMR spectrum of **S2** (600 MHz, DMSO-d_6).

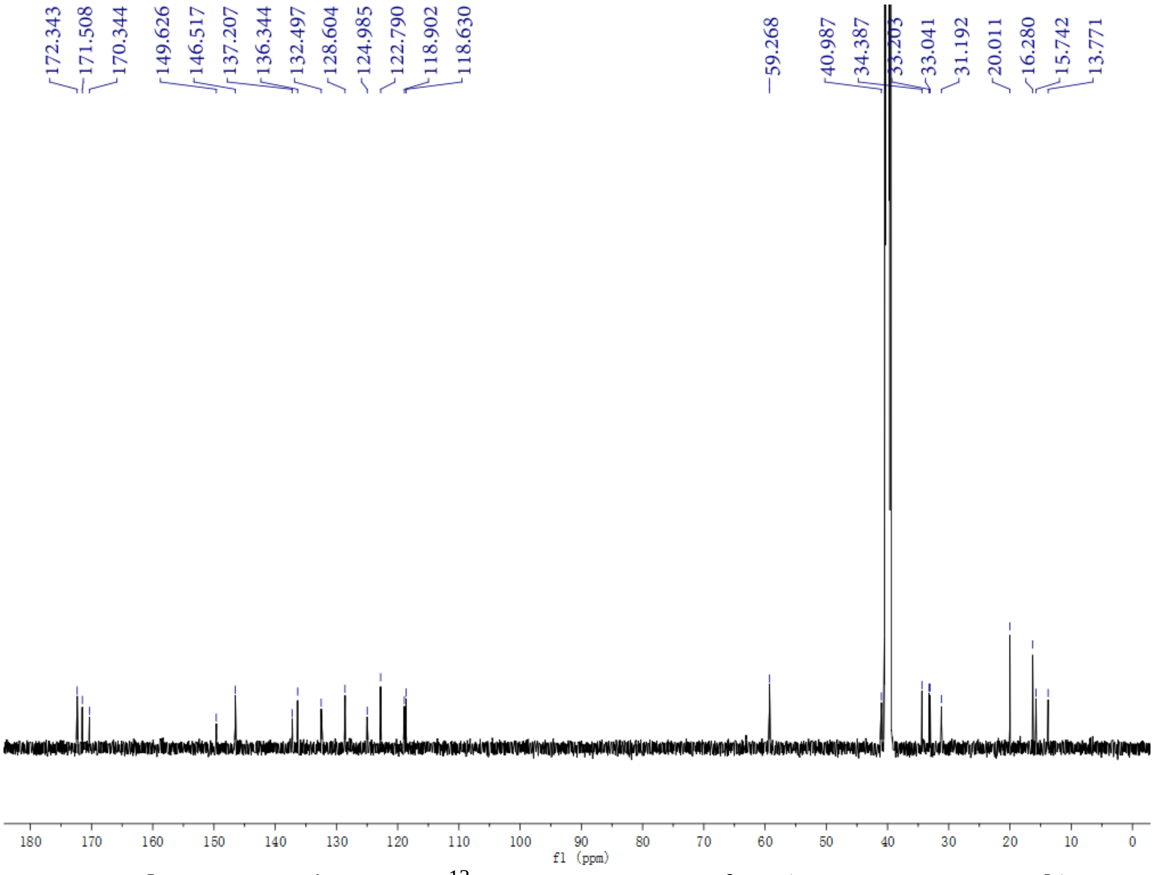

Supplementary Figure 80. ^{13}C NMR spectrum of **S2** (175 MHz, DMSO-d_6).

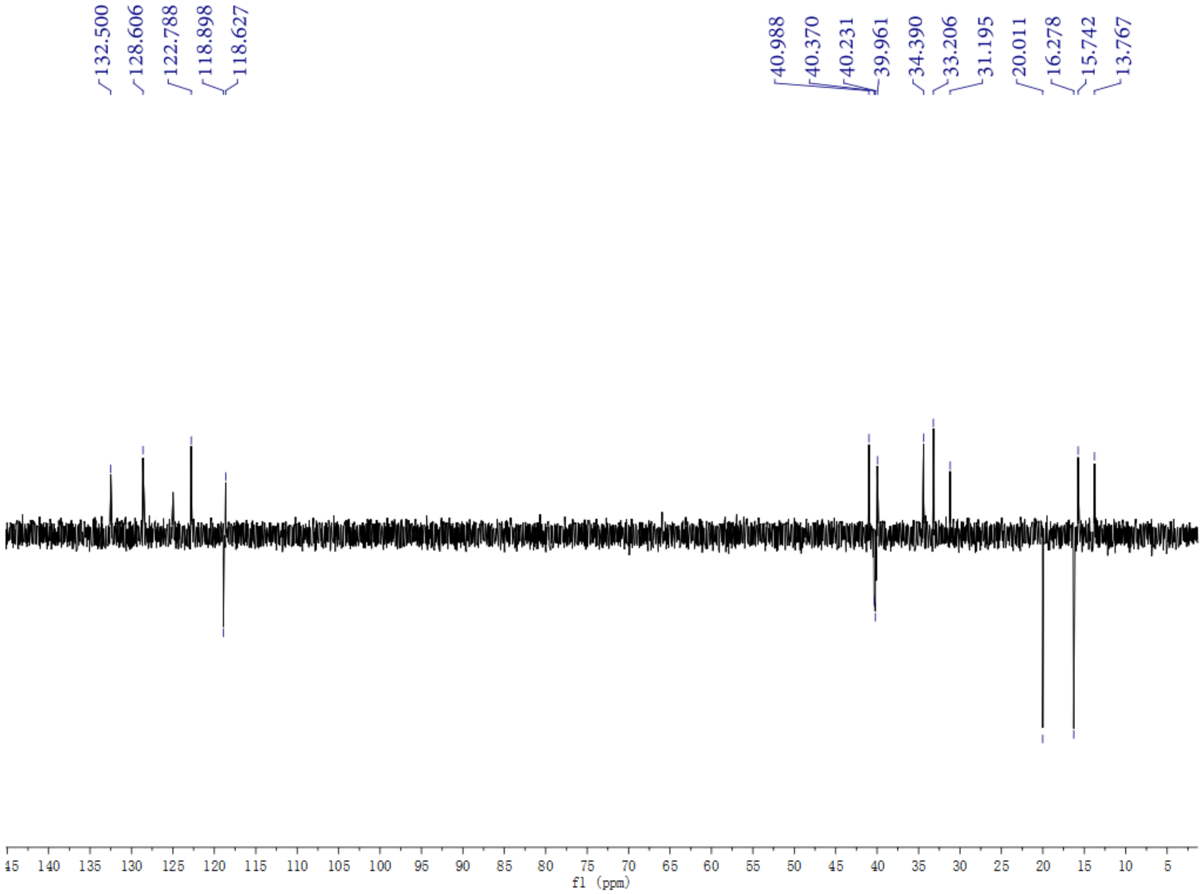

Supplementary Figure 81. DEPT-135 spectrum of **S2** (DMSO-d_6).

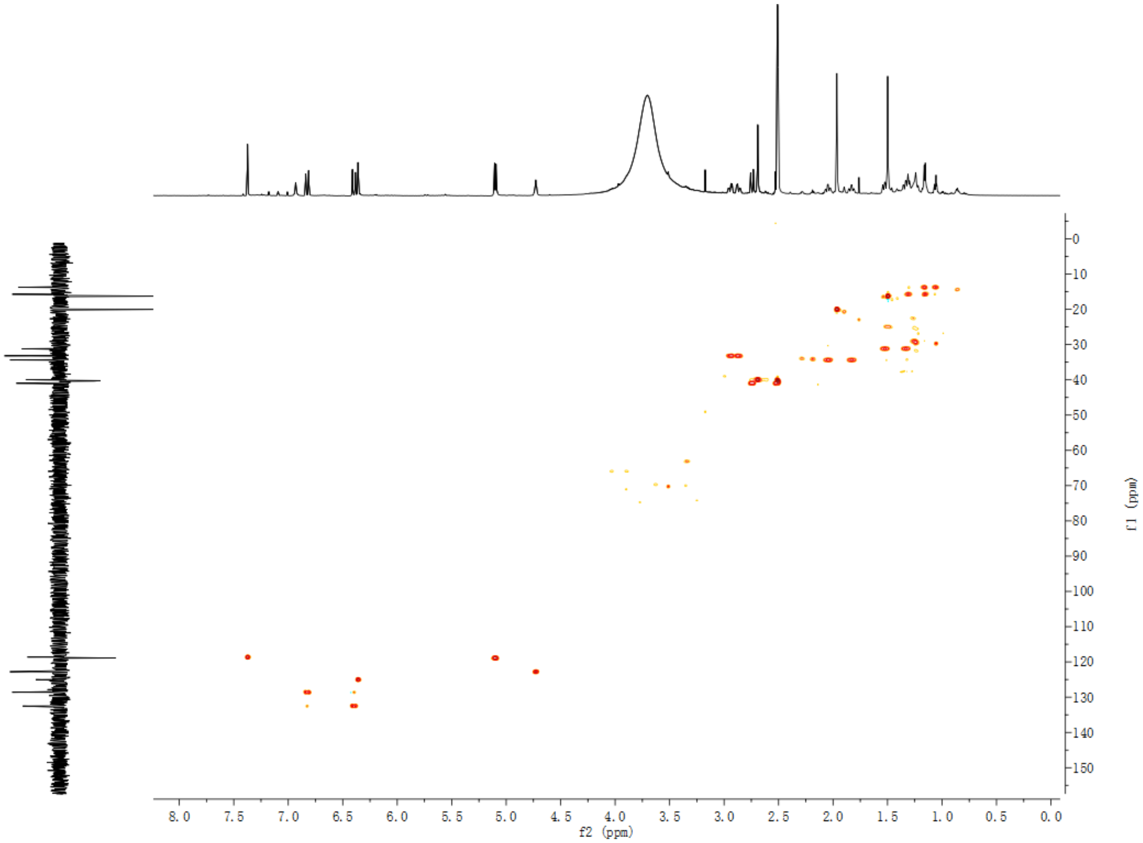

Supplementary Figure 82. HSQC spectrum of **S2** (DMSO-d_6).

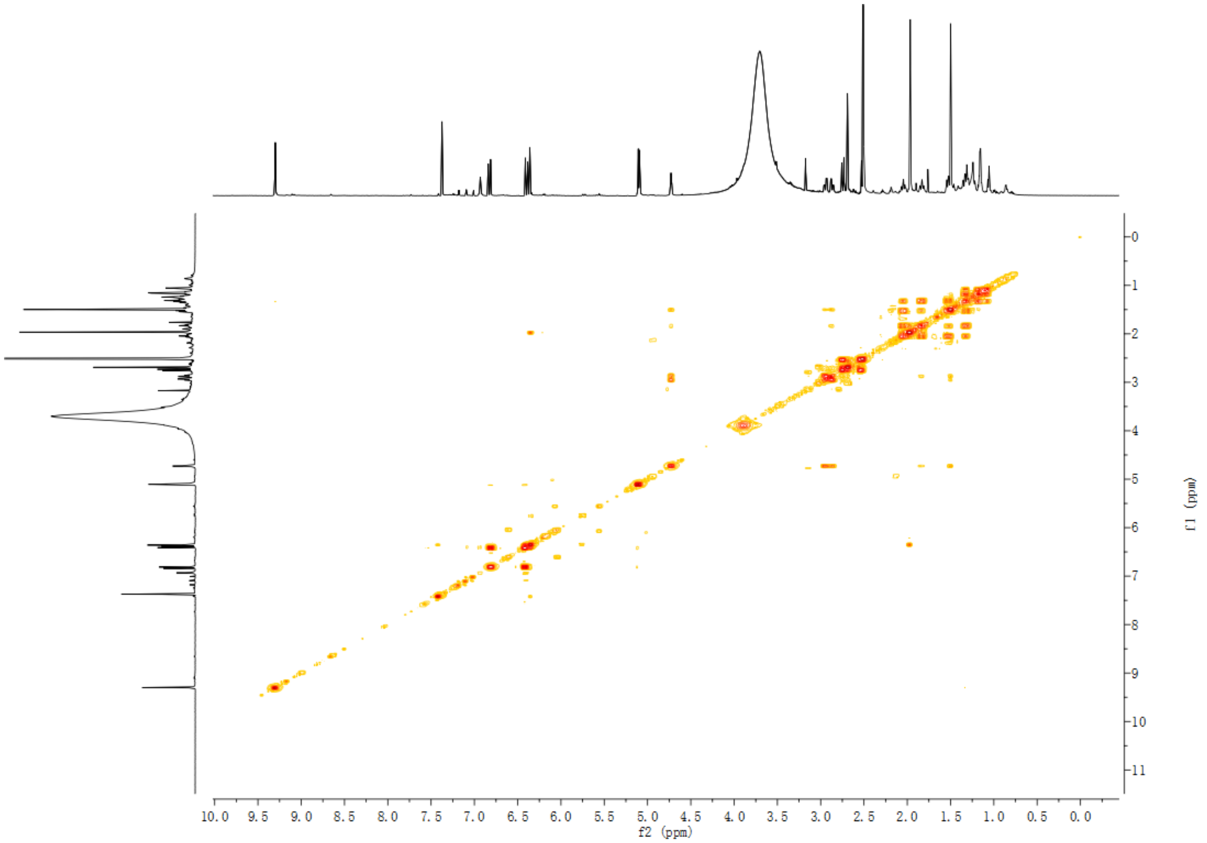

Supplementary Figure 83. ^{1}H-^{1}H COSY spectrum of **S2** (DMSO-d_6).

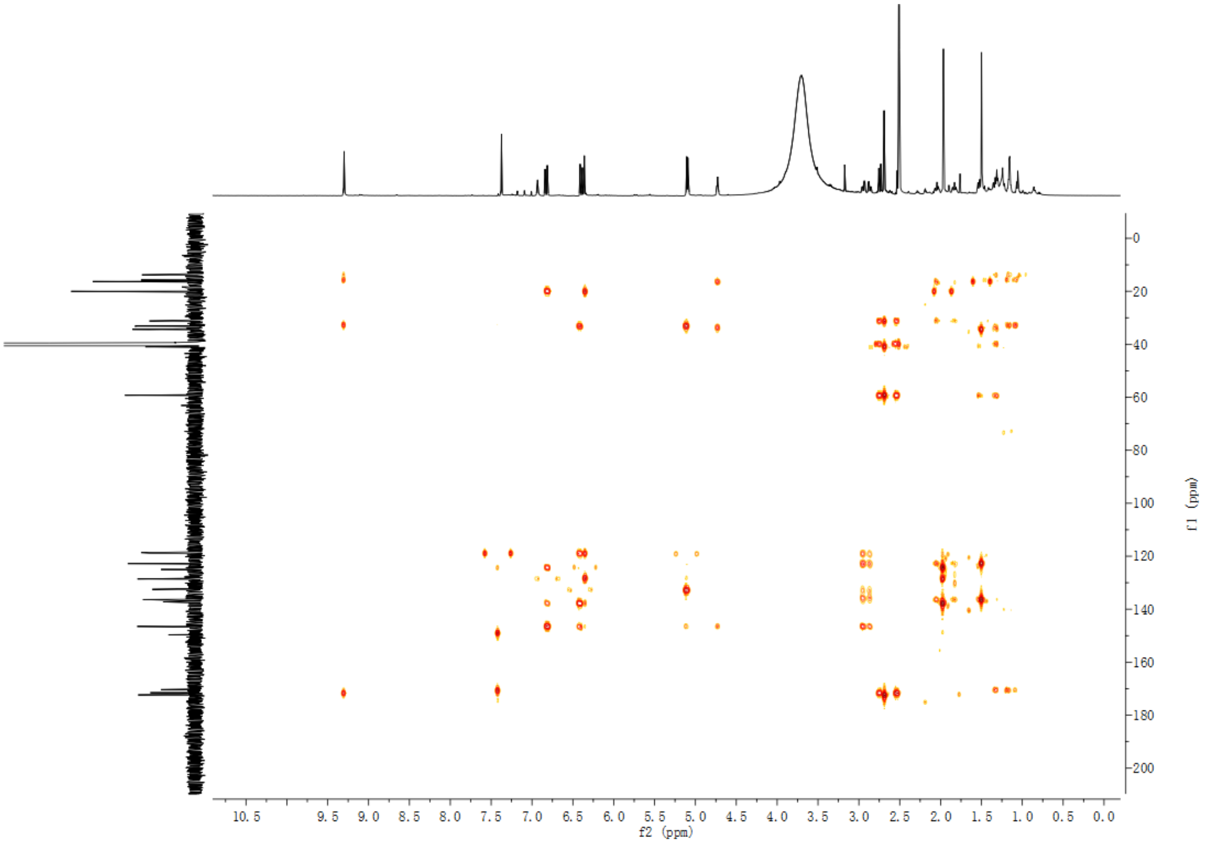

Supplementary Figure 84. HMBC spectrum of **S2** (DMSO-d_6).

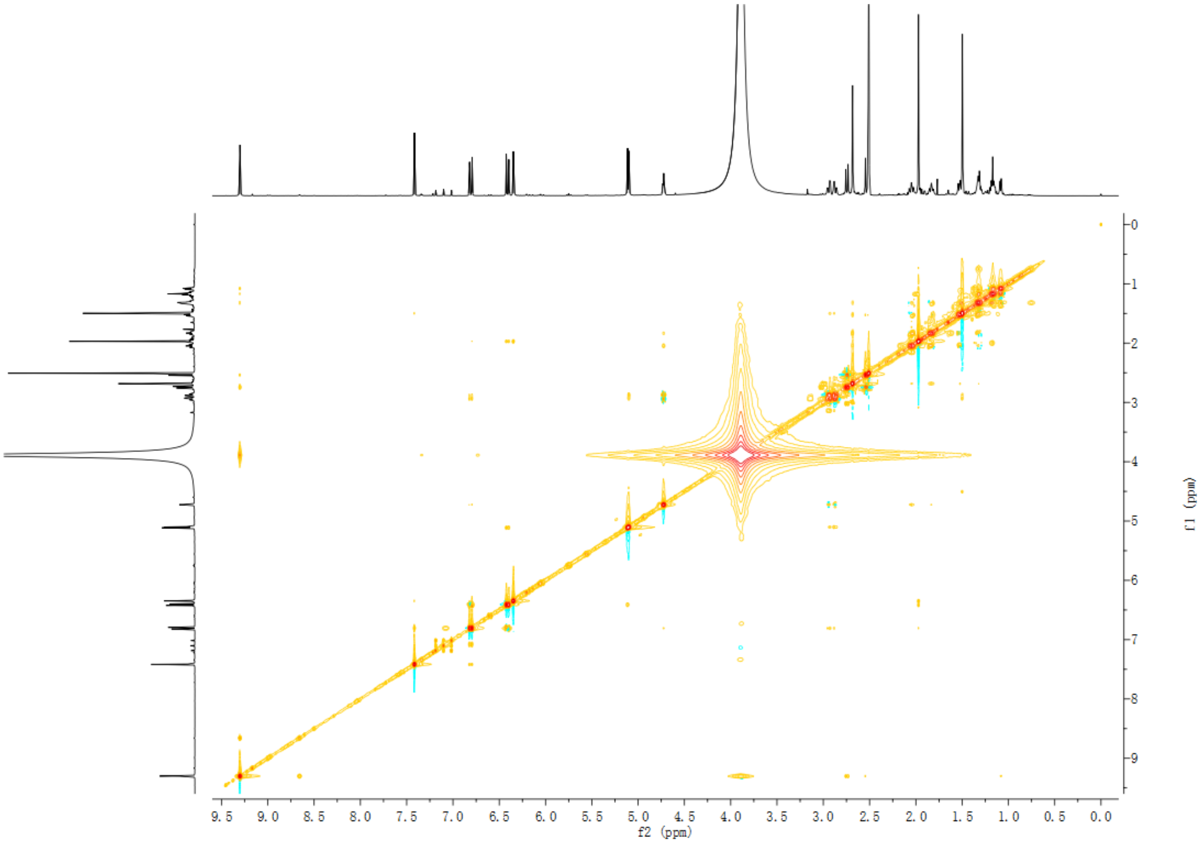

Supplementary Figure 85. ROESY spectrum of **S2** (DMSO-d_6).

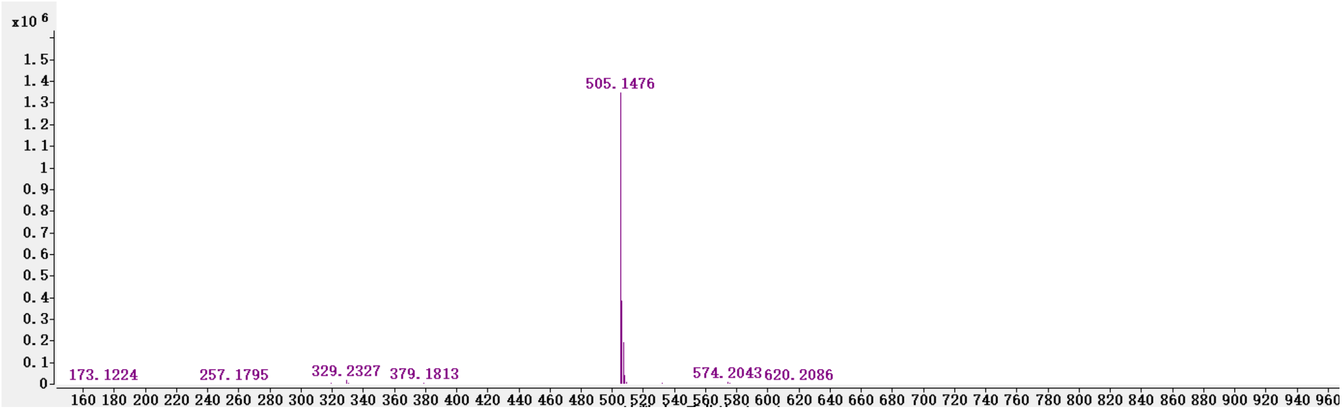

Supplementary Figure 86. HR-MS (ESI) spectrum of **S2**.

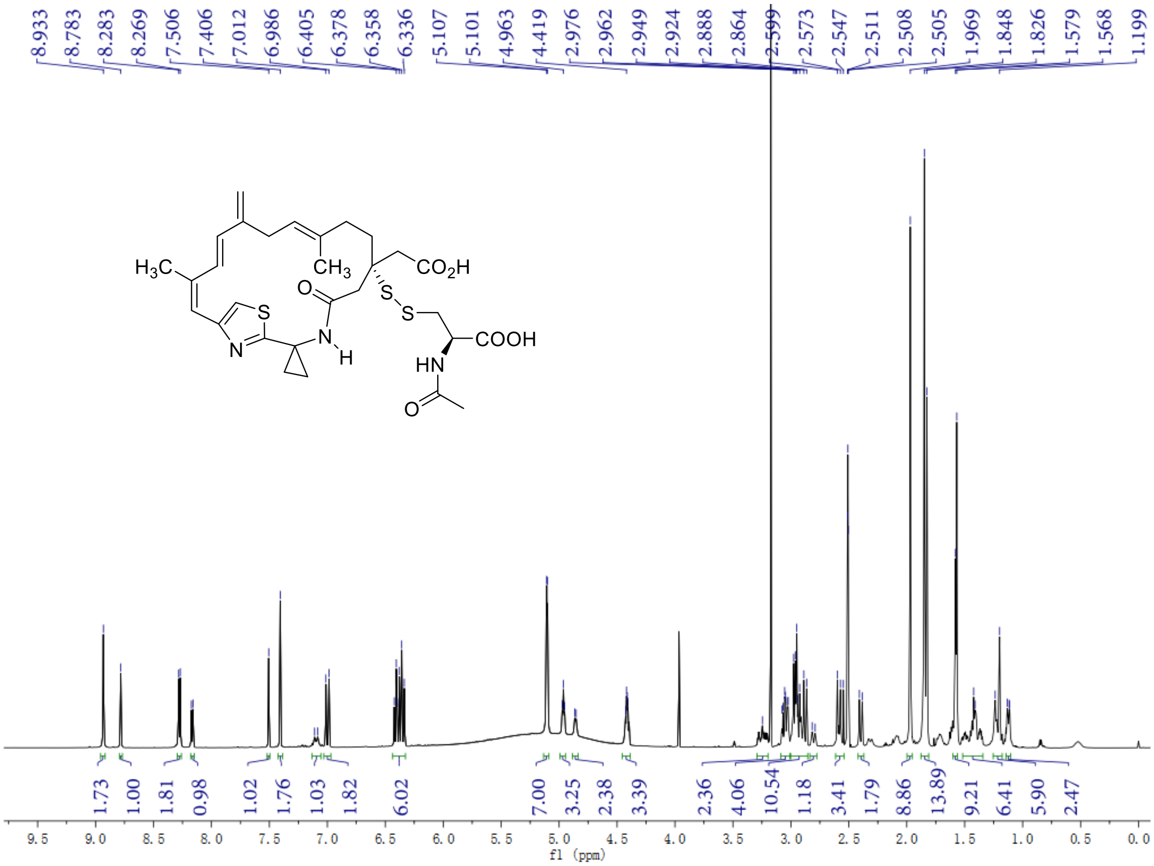

Supplementary Figure 87. ^{1}H NMR spectrum of **S3** (600 MHz, DMSO-d_6).

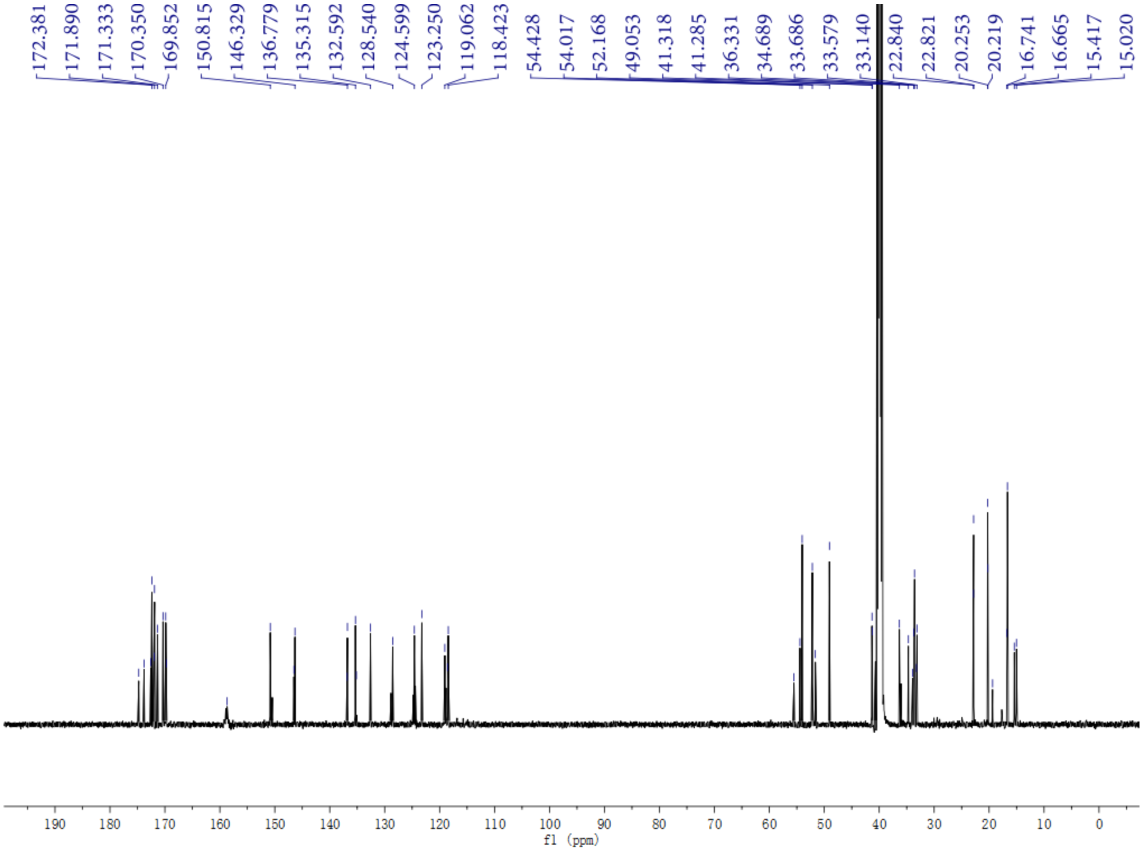

Supplementary Figure 88. ^{13}C NMR spectrum of **S3** (150 MHz, DMSO-d_6).

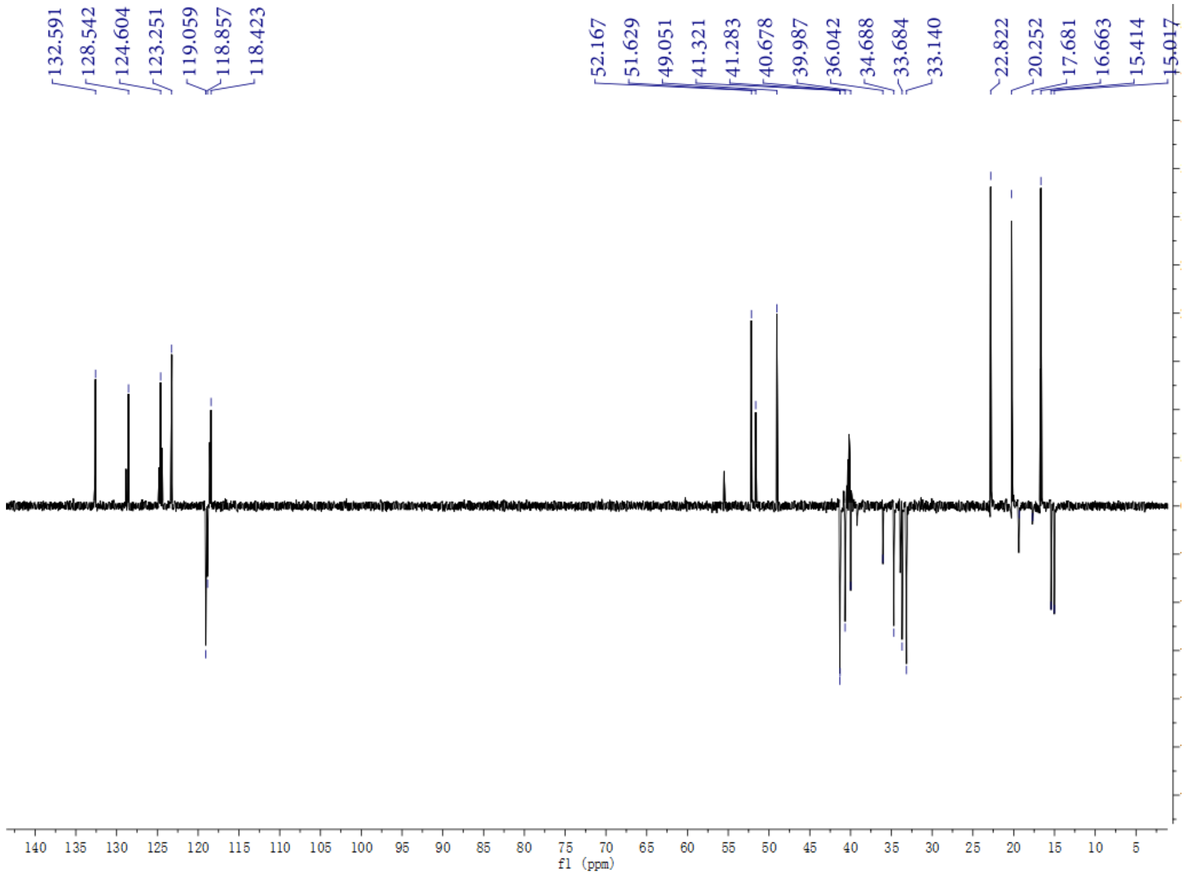

Supplementary Figure 89. DEPT-135 spectrum of **S3** (150 MHz, DMSO-d_6).

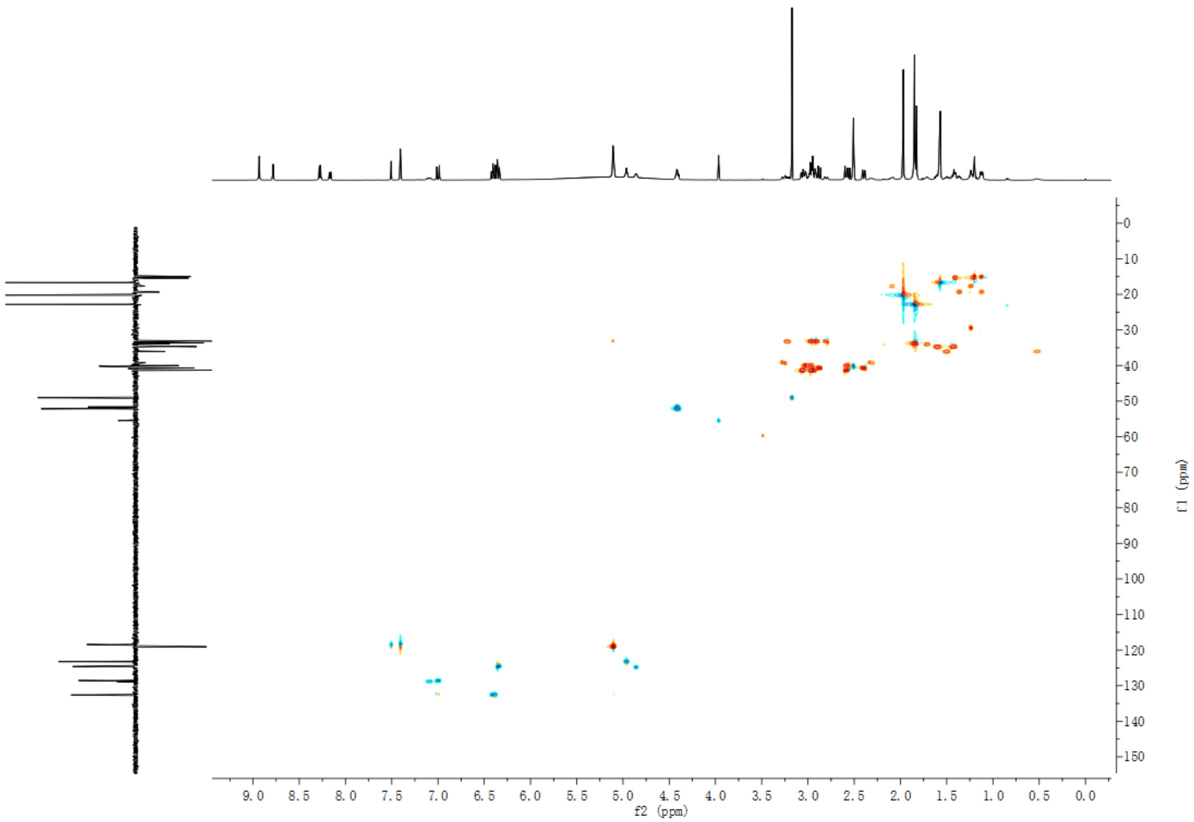

Supplementary Figure 90. HSQC spectrum of **S3** (DMSO-d_6).

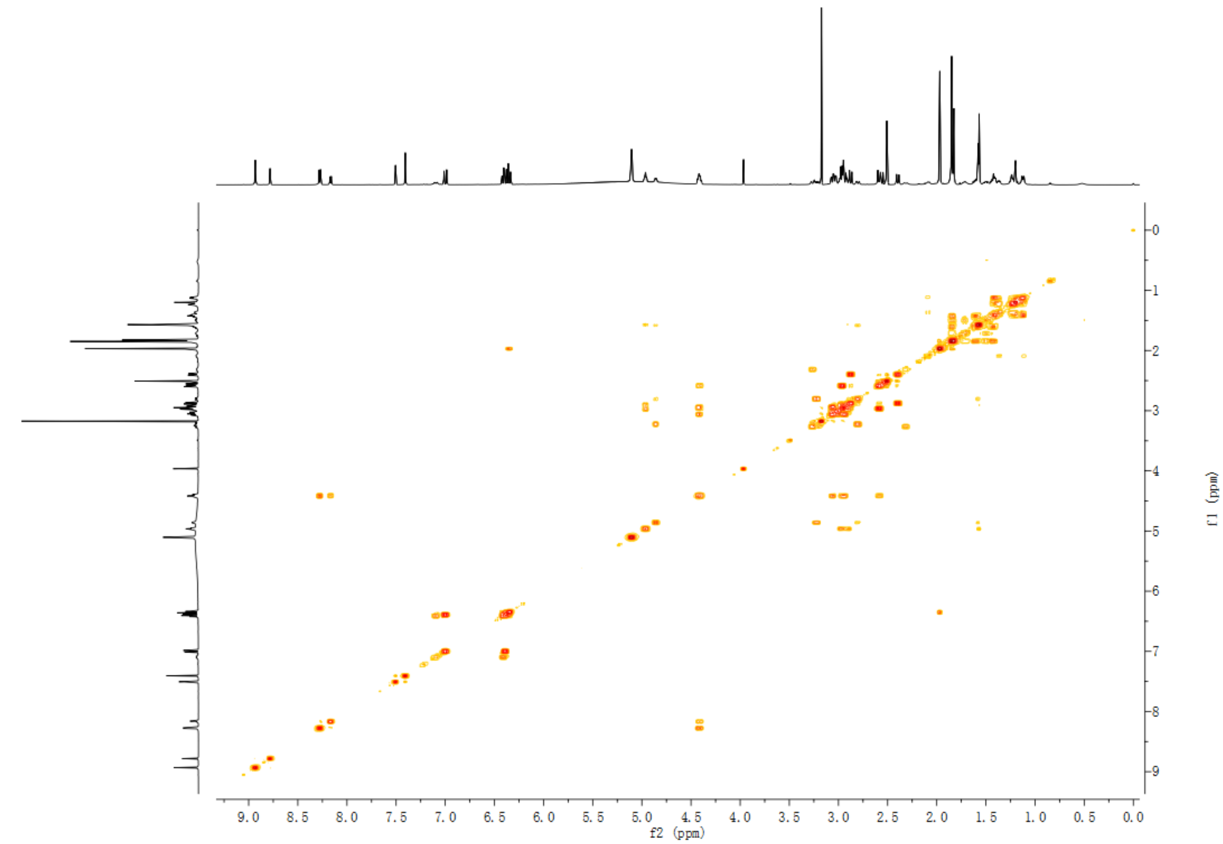

Supplementary Figure 91. ^{1}H-^{1}H COSY spectrum of **S3** (DMSO-d_6).

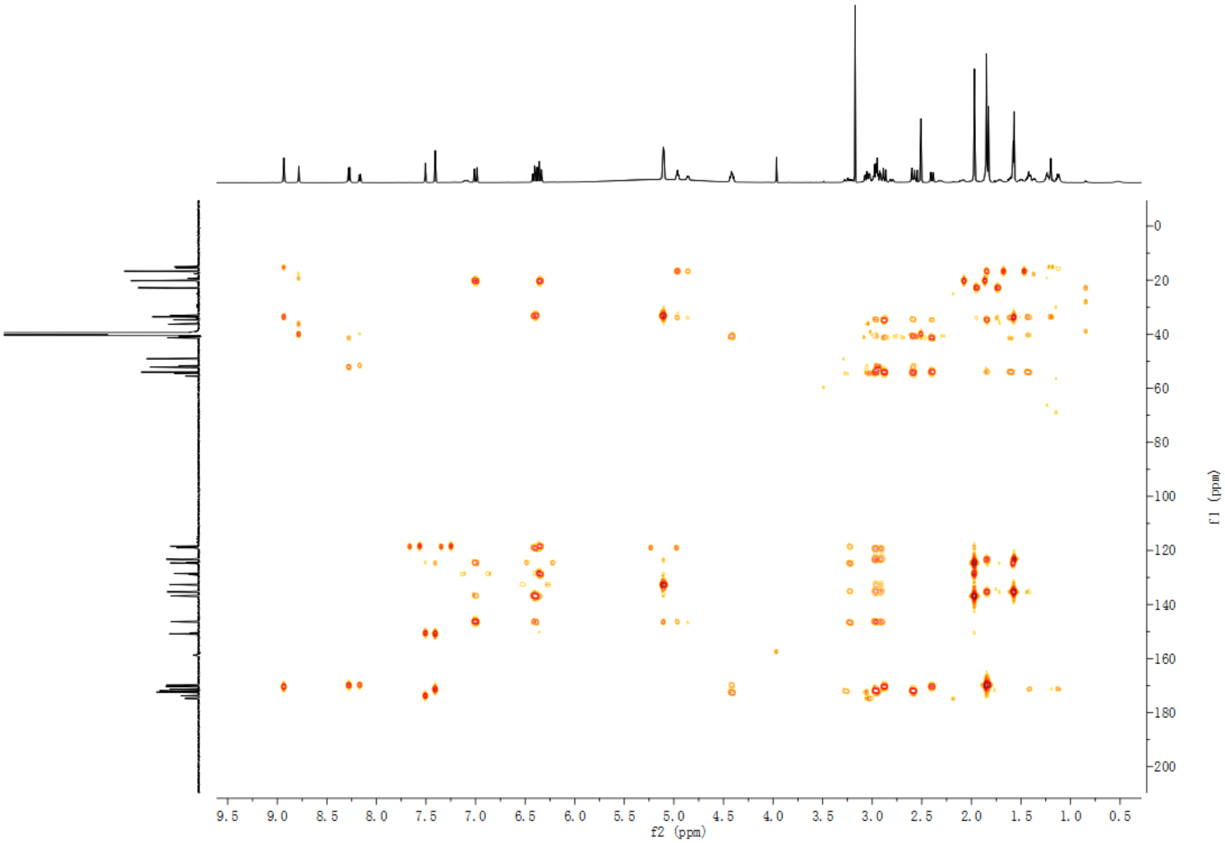

Supplementary Figure 92. HMBC spectrum of **S3** (DMSO-d_6).

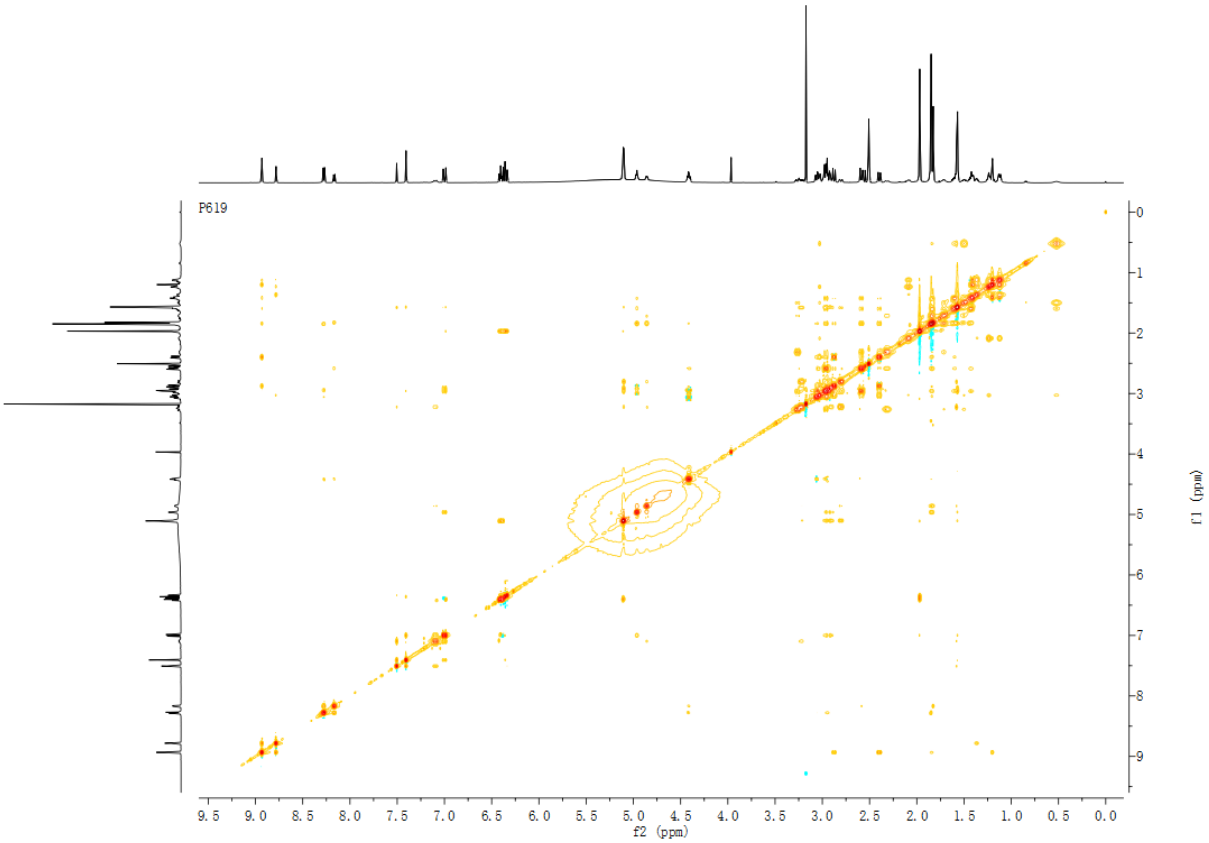

Supplementary Figure 93. ROESY spectrum of **S3** (DMSO-d_6).

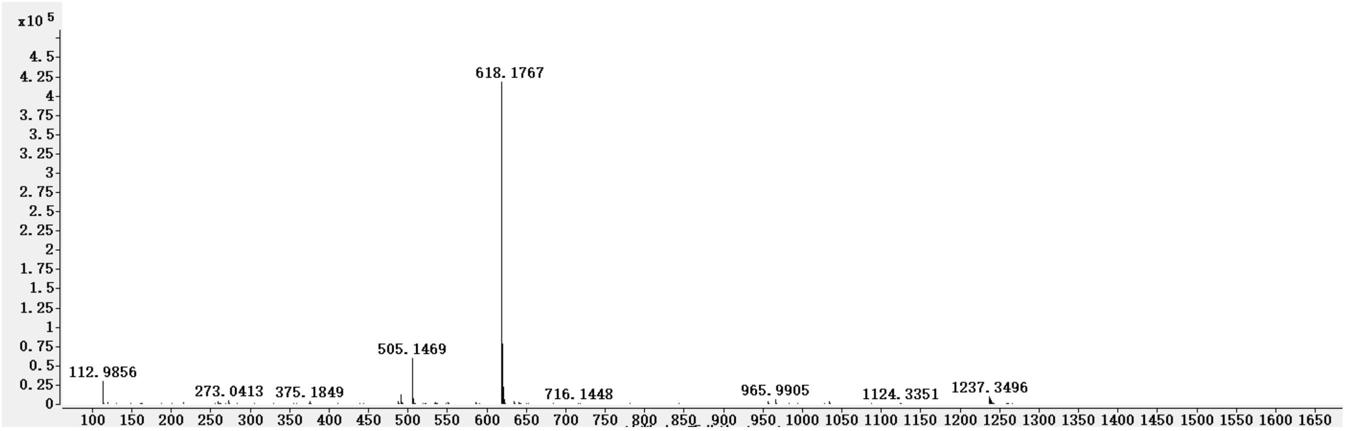

Supplementary Figure 94. HR-MS (ESI) spectrum of **S3**.

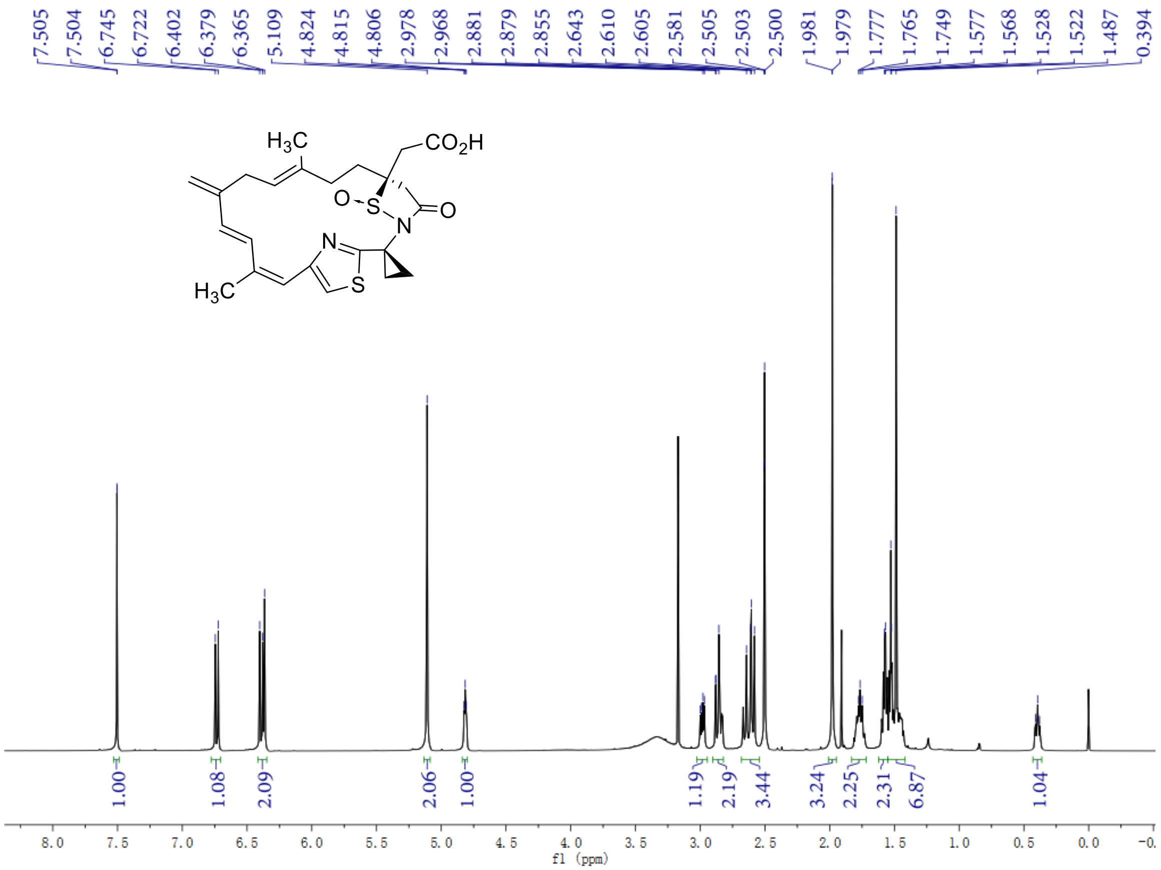

Supplementary Figure 95. ¹H NMR spectrum of **S4** (600 MHz, DMSO-d_6).

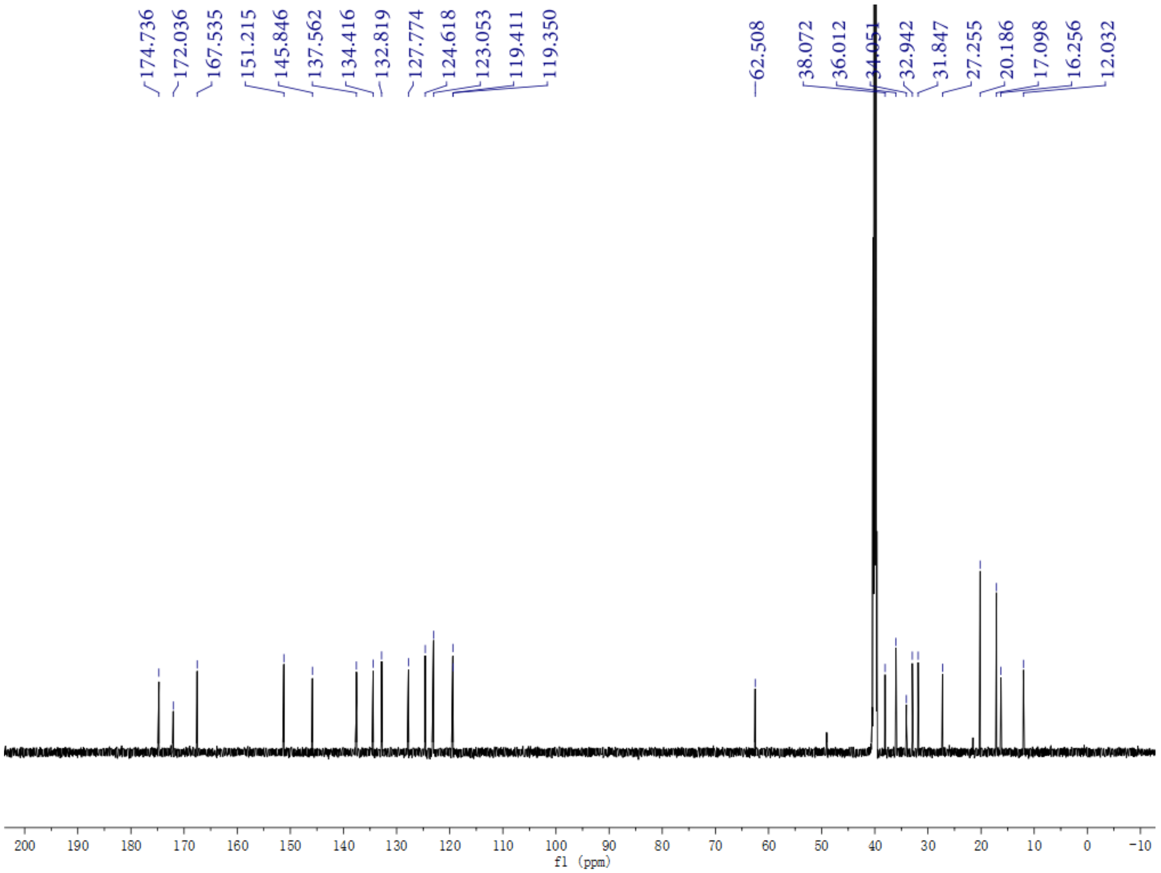

Supplementary Figure 96. ¹³C NMR spectrum of **S4** (150 MHz, DMSO-d_6).

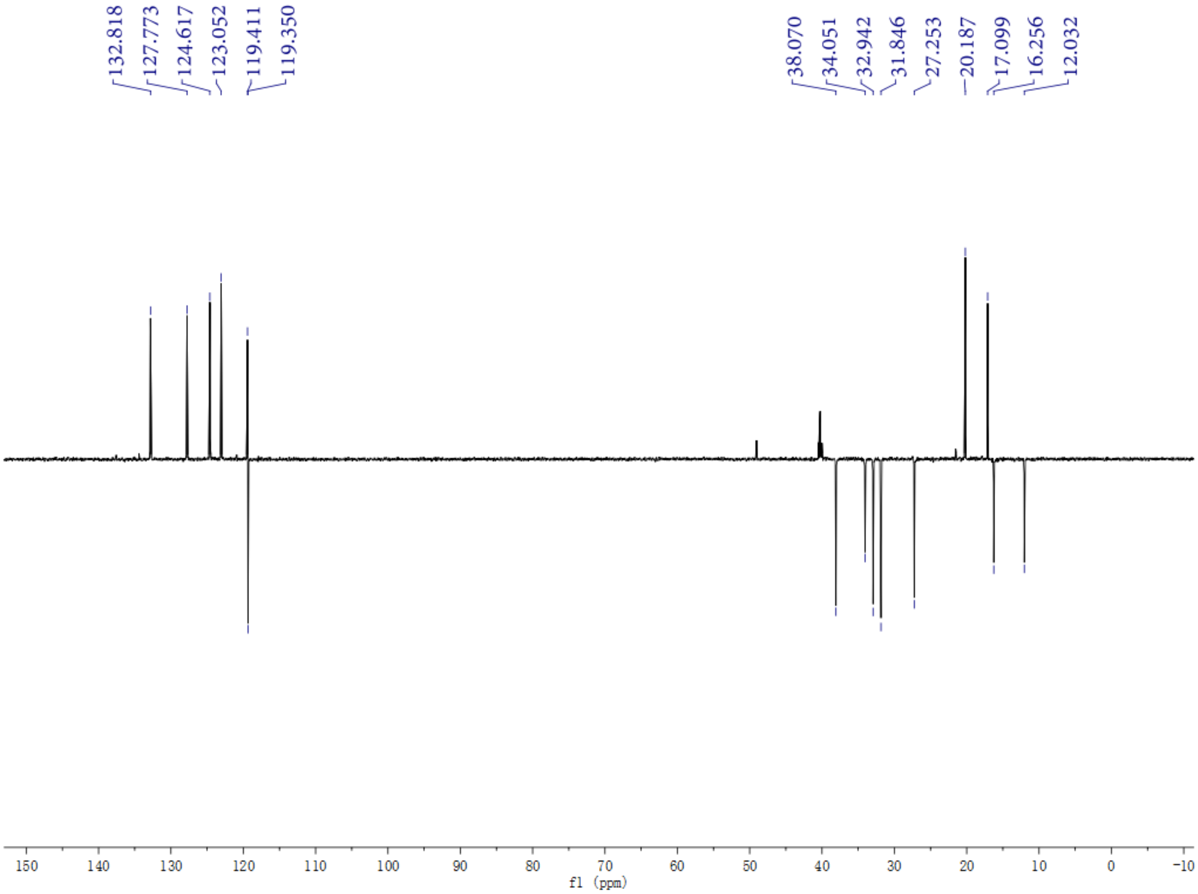

Supplementary Figure 97. DEPT-135 spectrum of **S4** (DMSO-d_6).

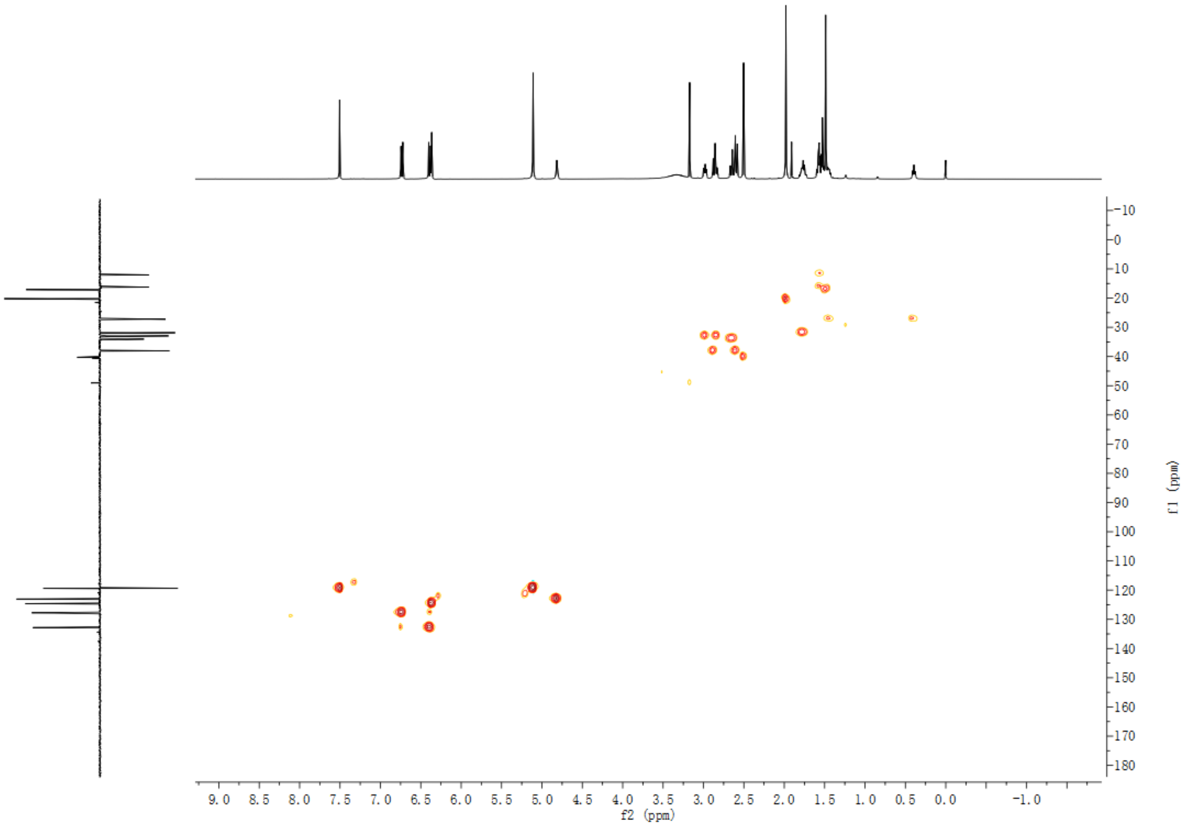

Supplementary Figure 98. HSQC spectrum of **S4** (DMSO-d_6).

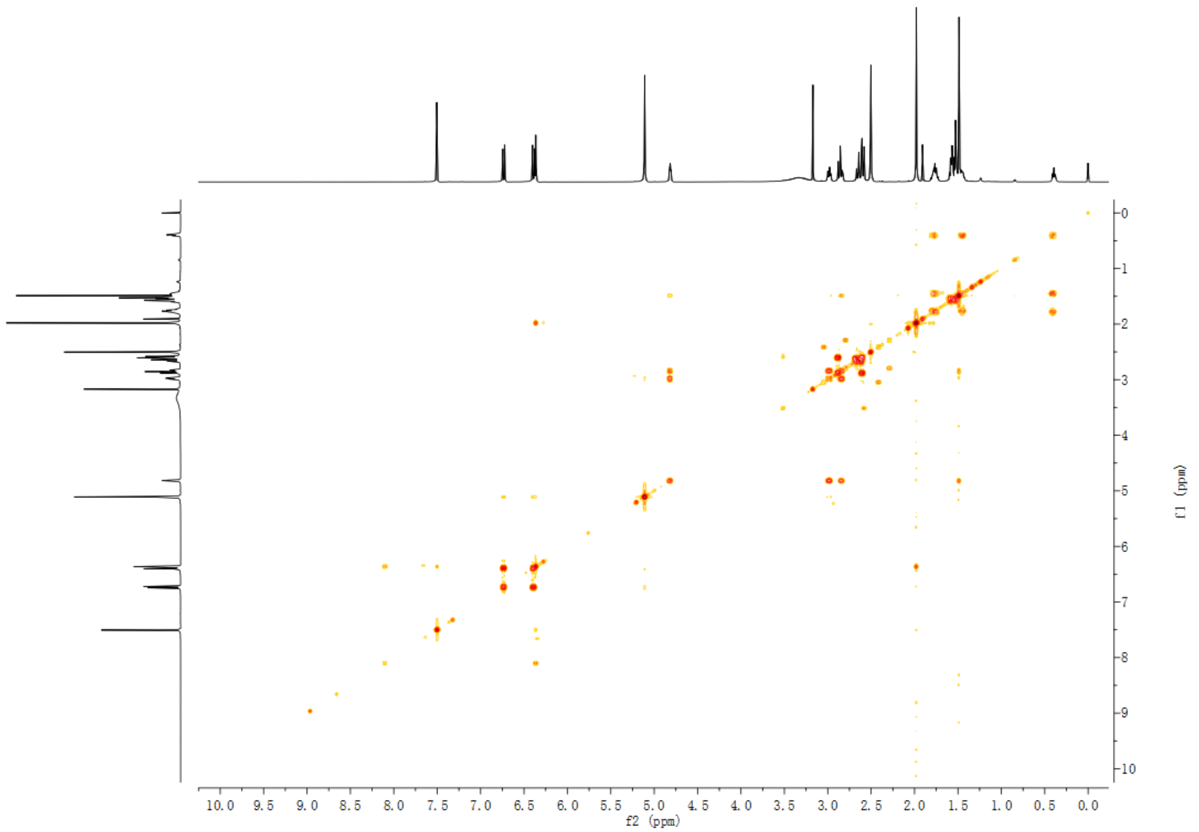

Supplementary Figure 99. ^{1}H-^{1}H COSY spectrum of **S4** (DMSO-d_6).

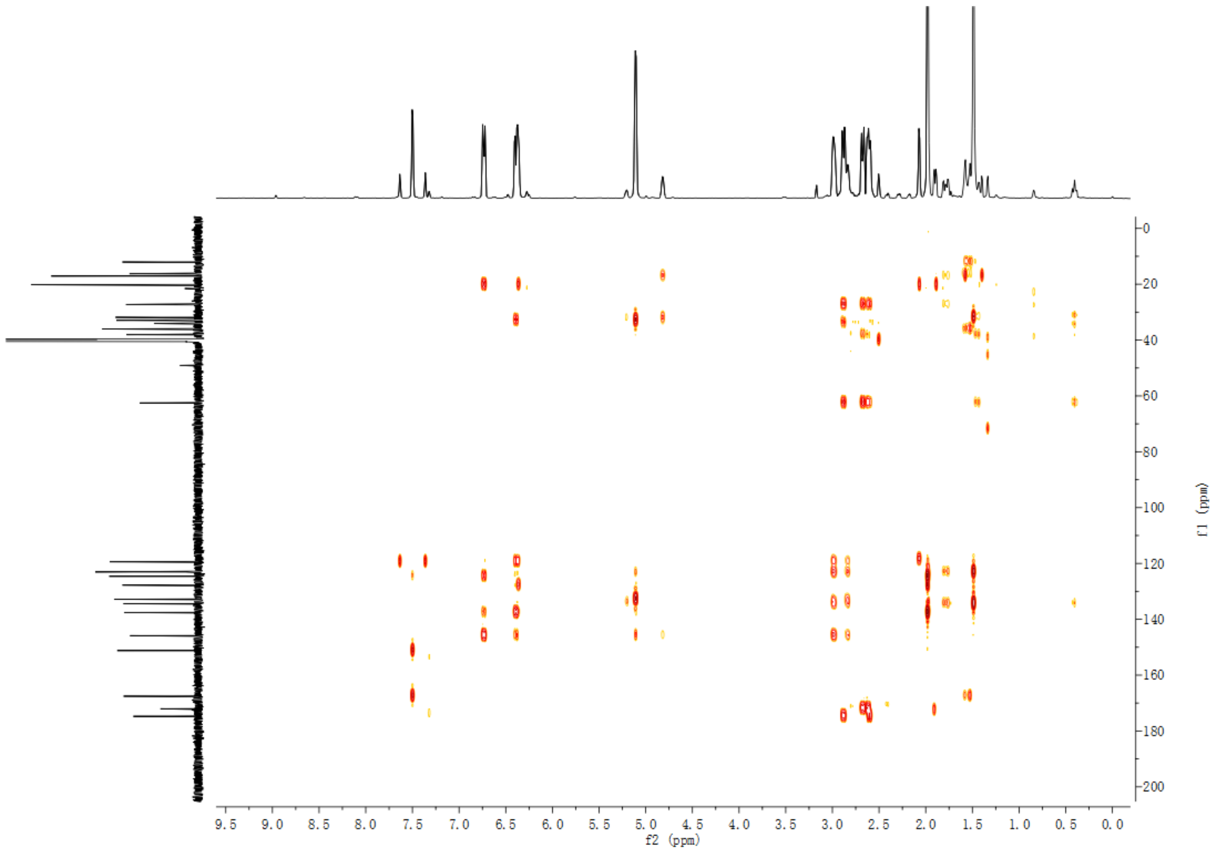

Supplementary Figure 100. HMBC spectrum of **S4** (DMSO-d_6).

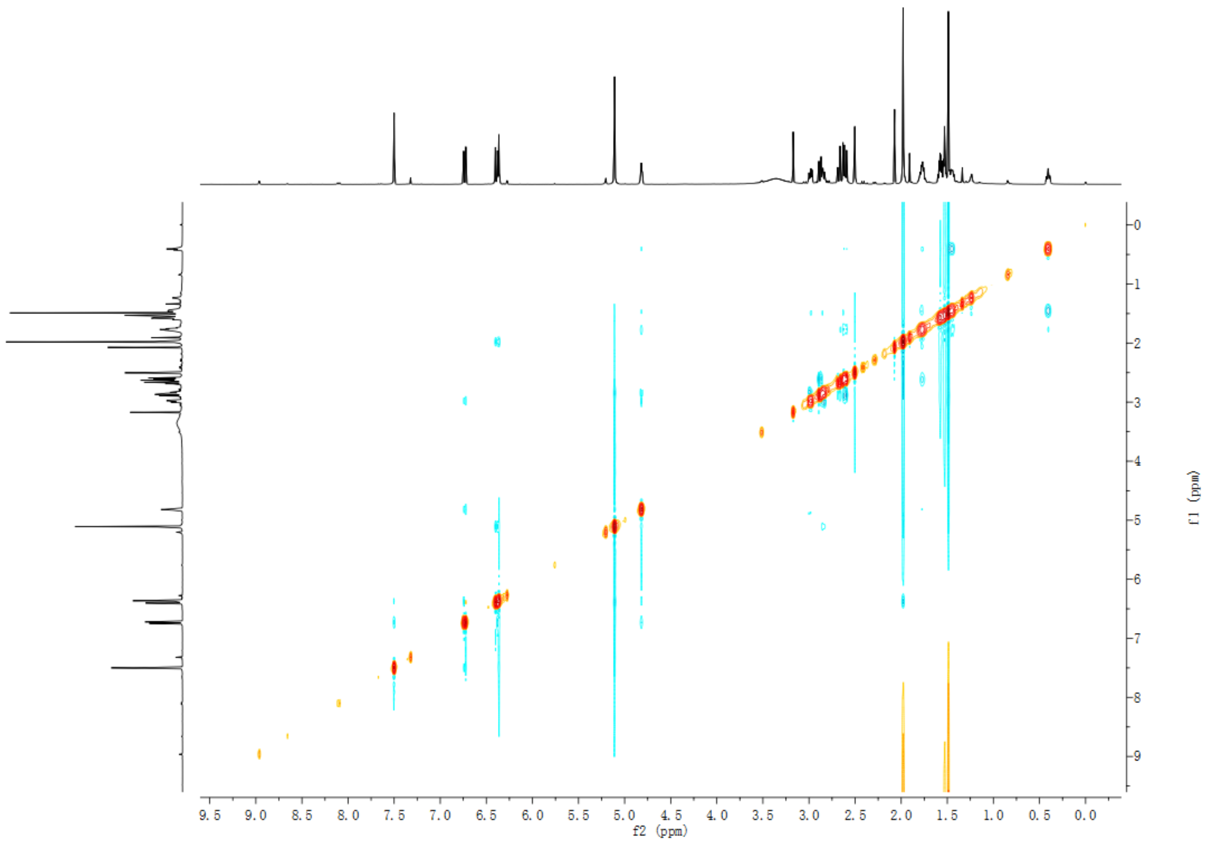

Supplementary Figure 101. ROESY spectrum of **S4** (DMSO-d_6).

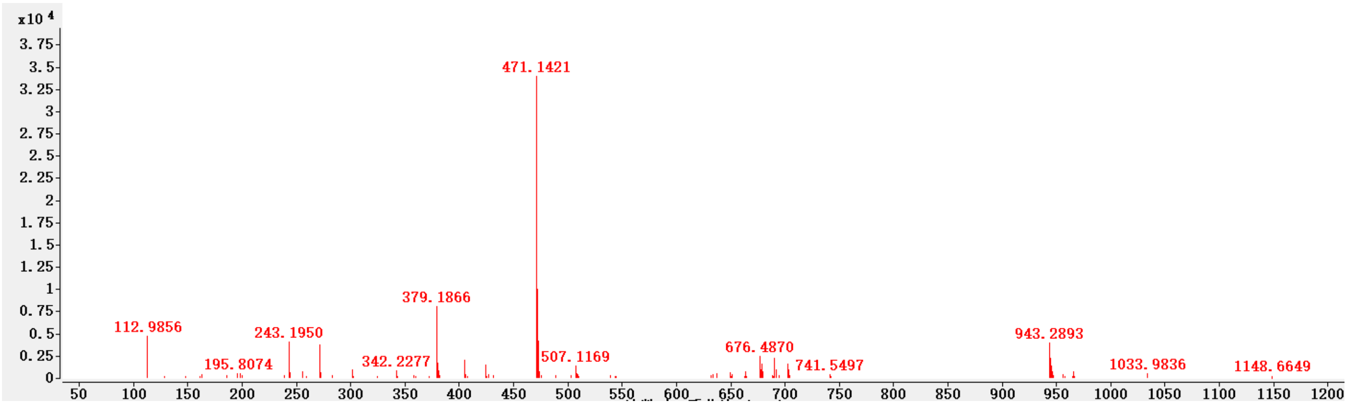

Supplementary Figure 102. HR-MS (ESI) spectrum of **S4**.

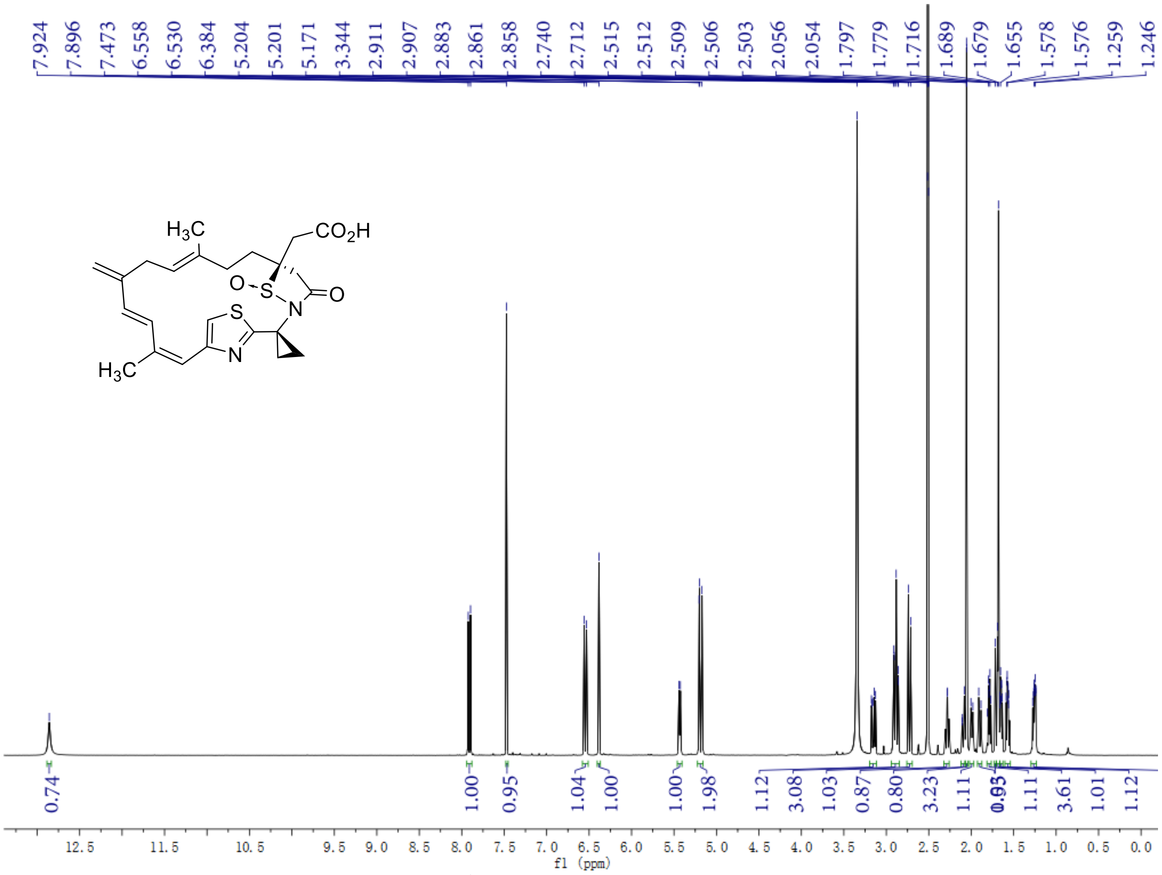

Supplementary Figure 103. ^{1}H NMR spectrum of **S5** (600 MHz, DMSO-d_6).

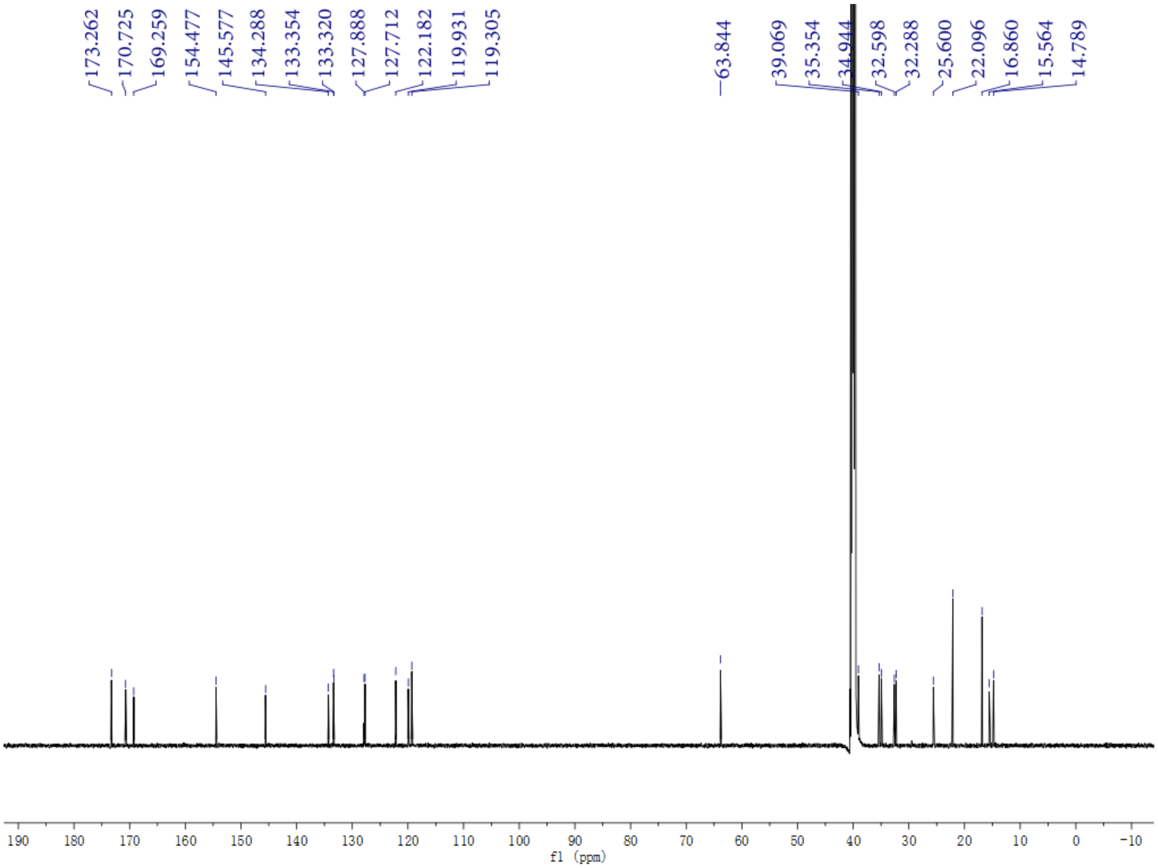

Supplementary Figure 104. ^{13}C NMR spectrum of **S5** (150 MHz, DMSO-d_6).

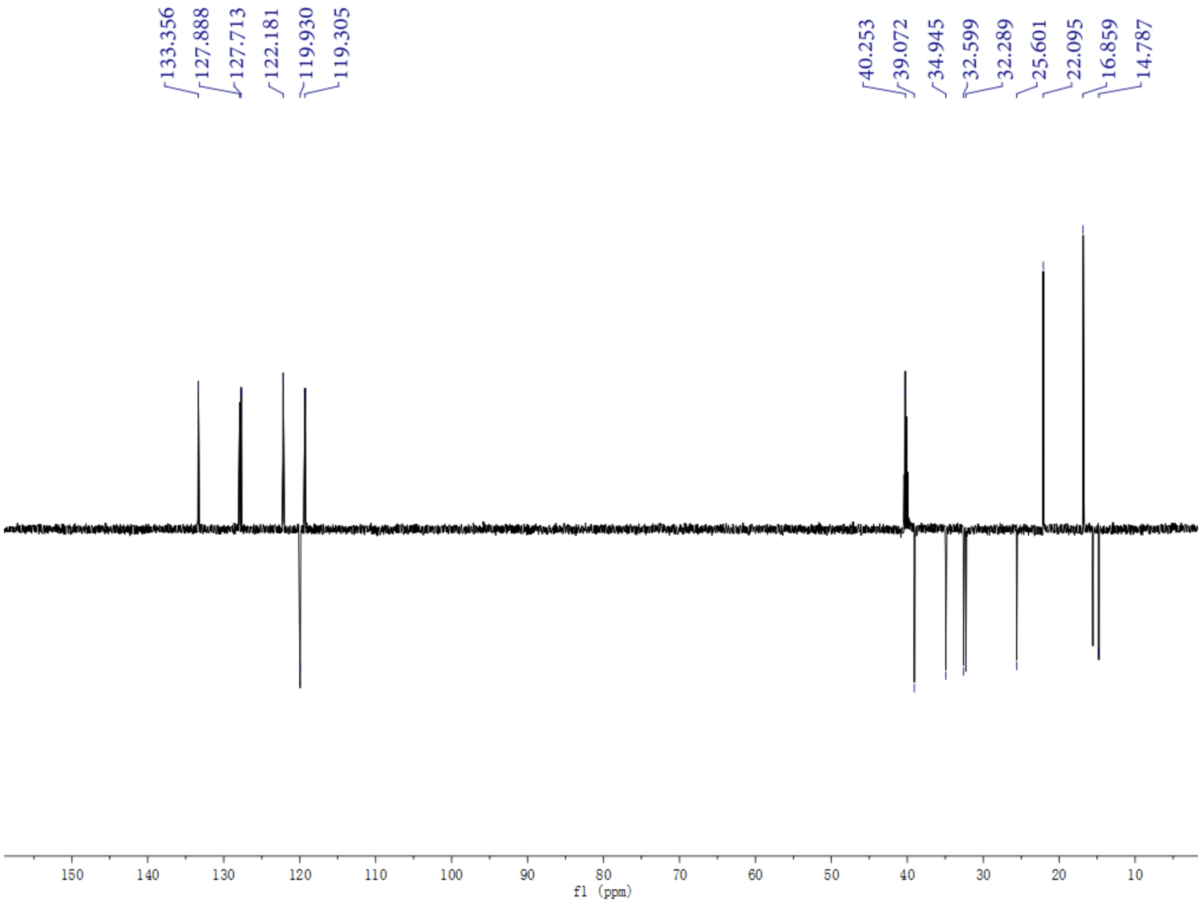

Supplementary Figure 105. DEPT-135 spectrum of **S5** (DMSO-d_6).

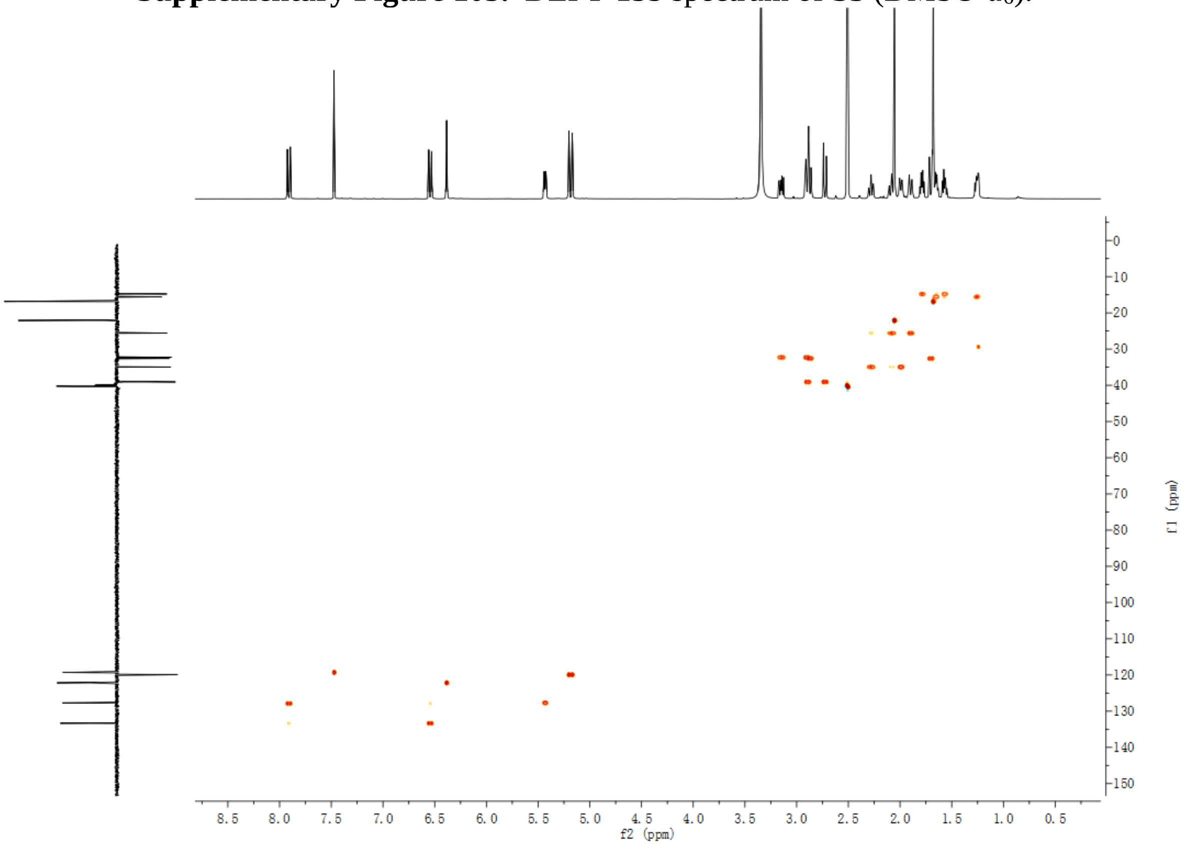

Supplementary Figure 106. HSQC spectrum of **S5** (DMSO-d_6).

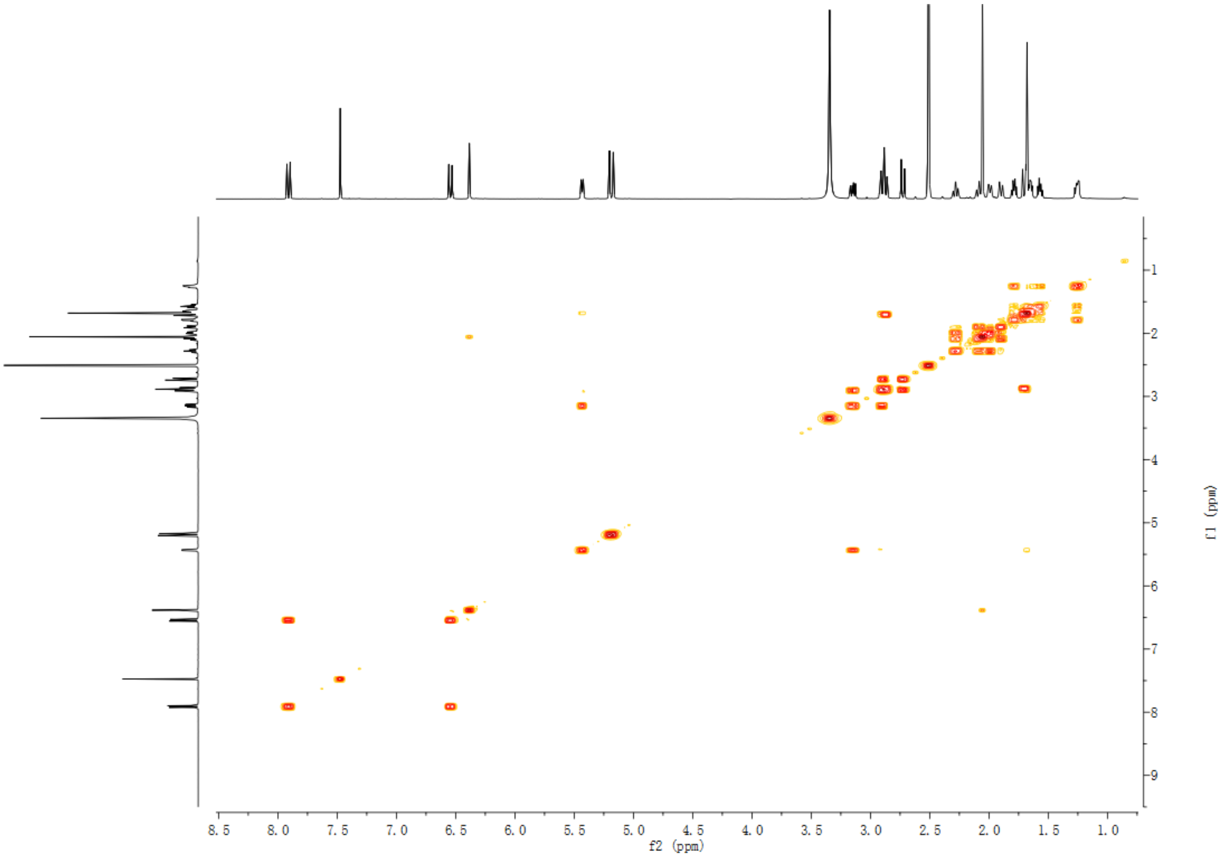

Supplementary Figure 107. ^{1}H-^{1}H COSY spectrum of **S5** (DMSO-d_6).

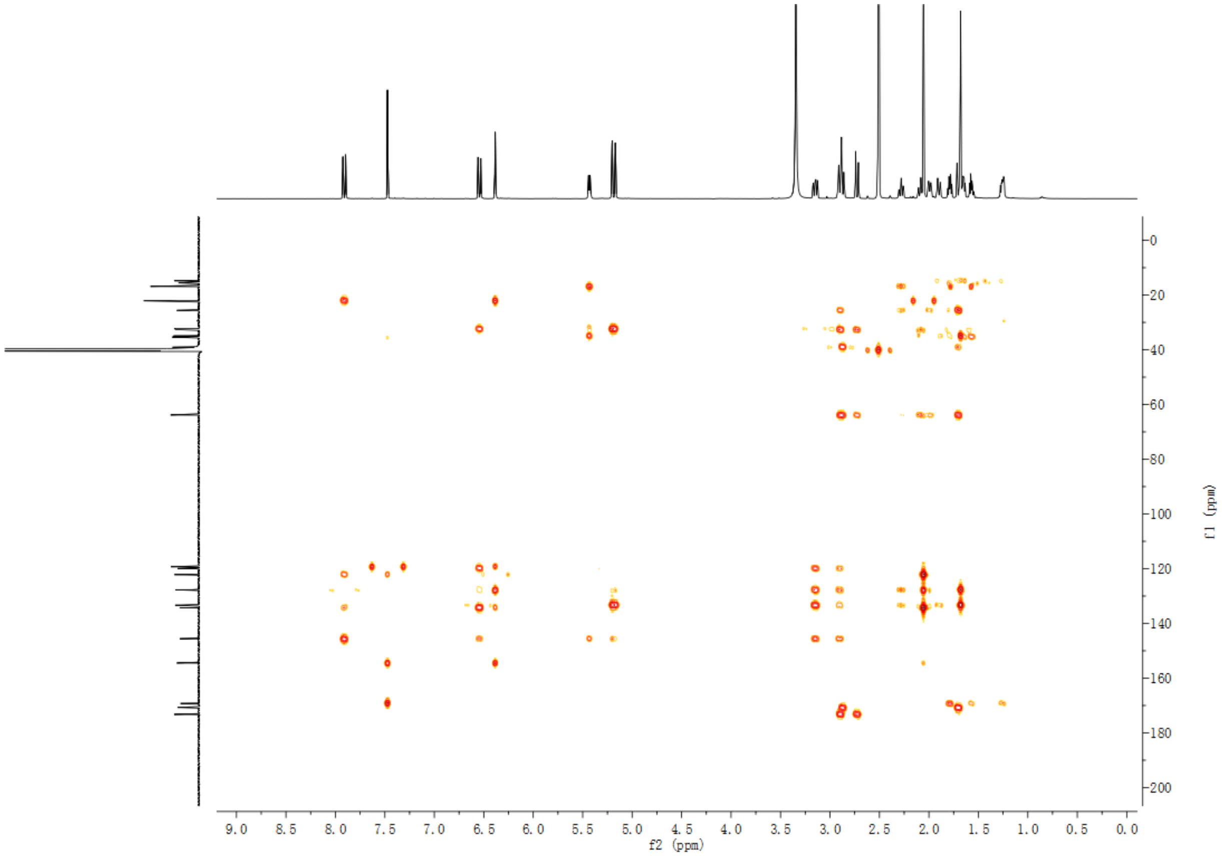

Supplementary Figure 108. HMBC spectrum of **S5** (DMSO-d_6).

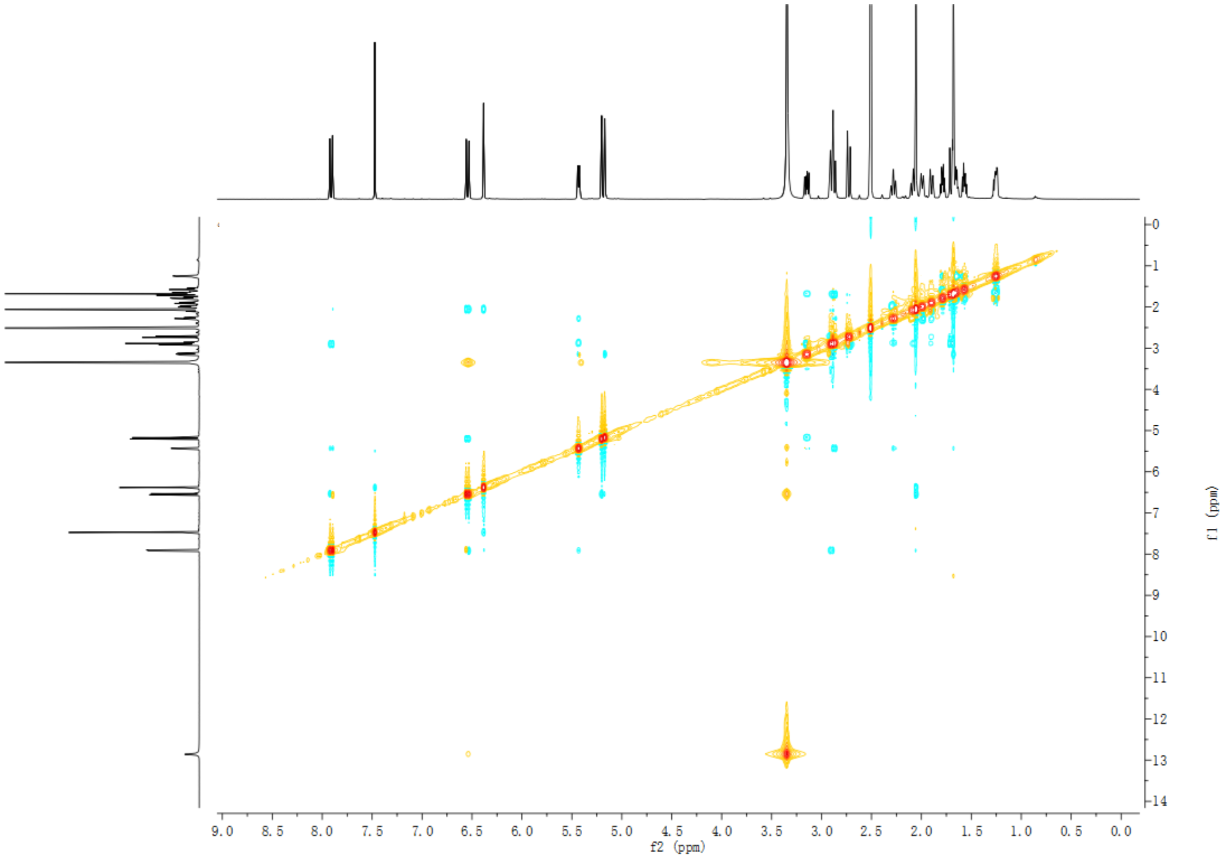

Supplementary Figure 109. ROESY spectrum of **S5** (DMSO-d_6).

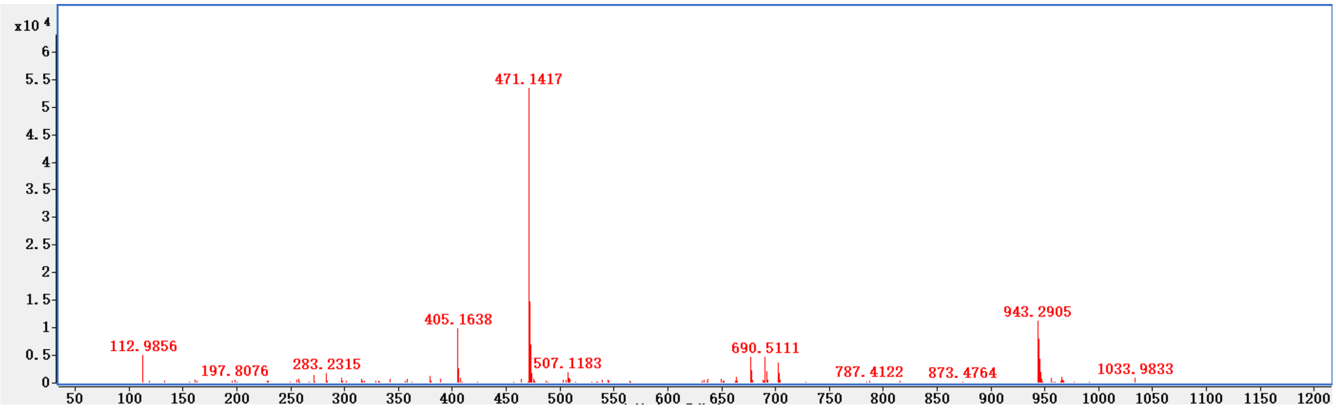

Supplementary Figure 110. HR-MS (ESI) spectrum of **S5**.

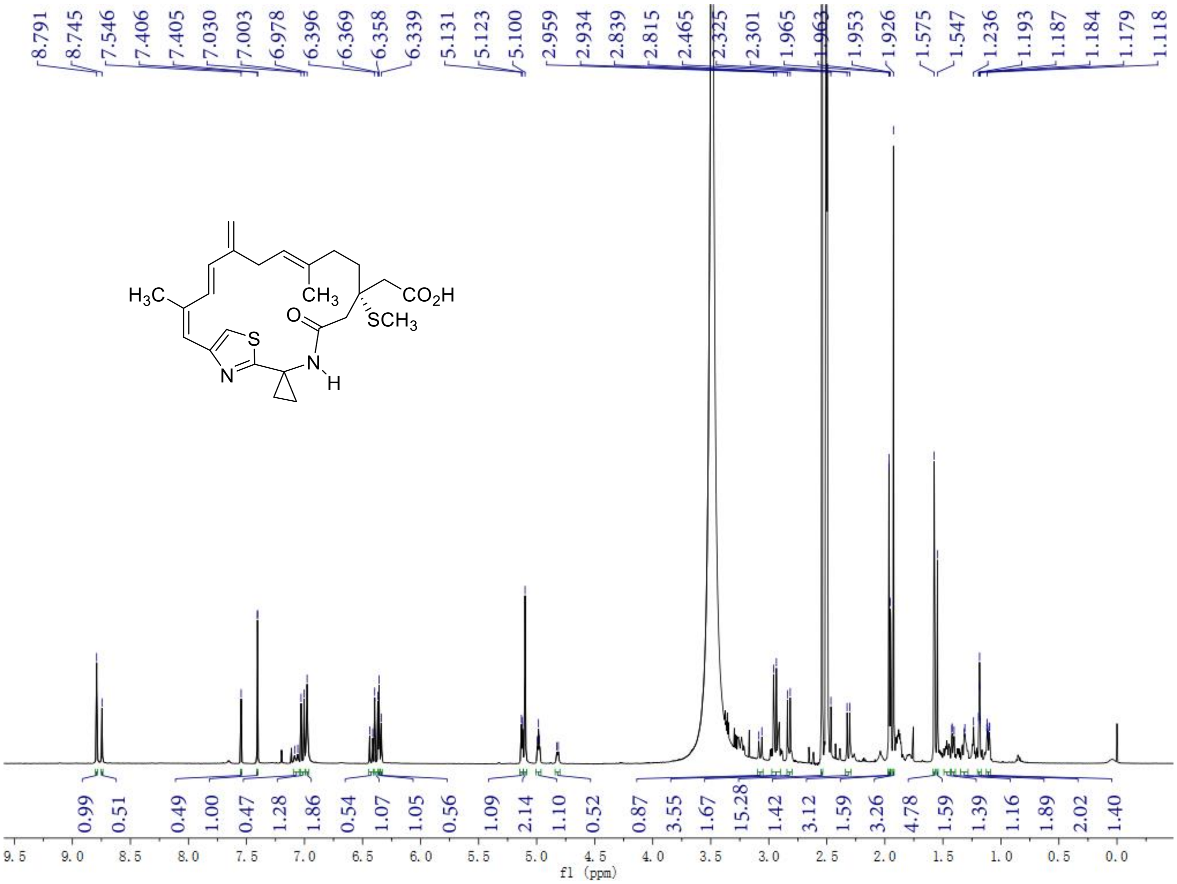

Supplementary Figure 111. ^{1}H NMR spectrum of **S8** (600 MHz, DMSO-d_6).

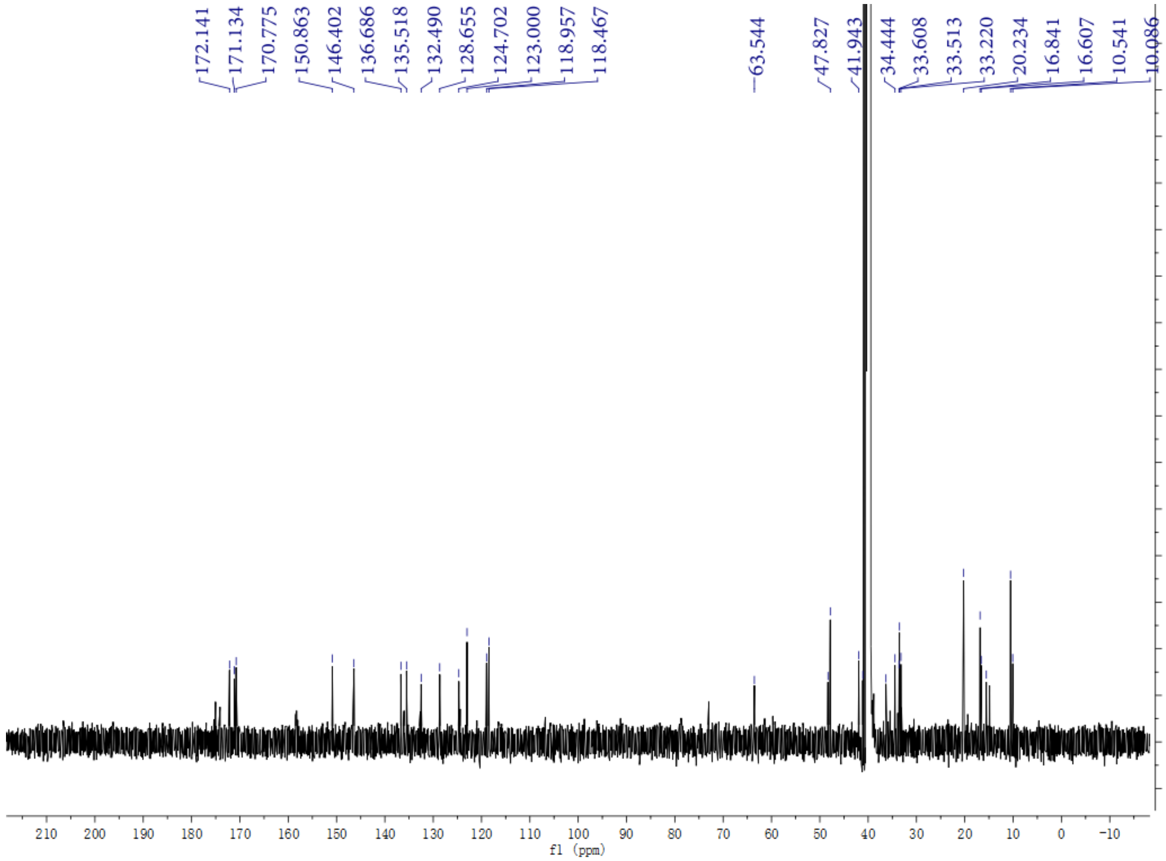

Supplementary Figure 112. ^{13}C NMR spectrum of **S8** (150 MHz, DMSO-d_6).

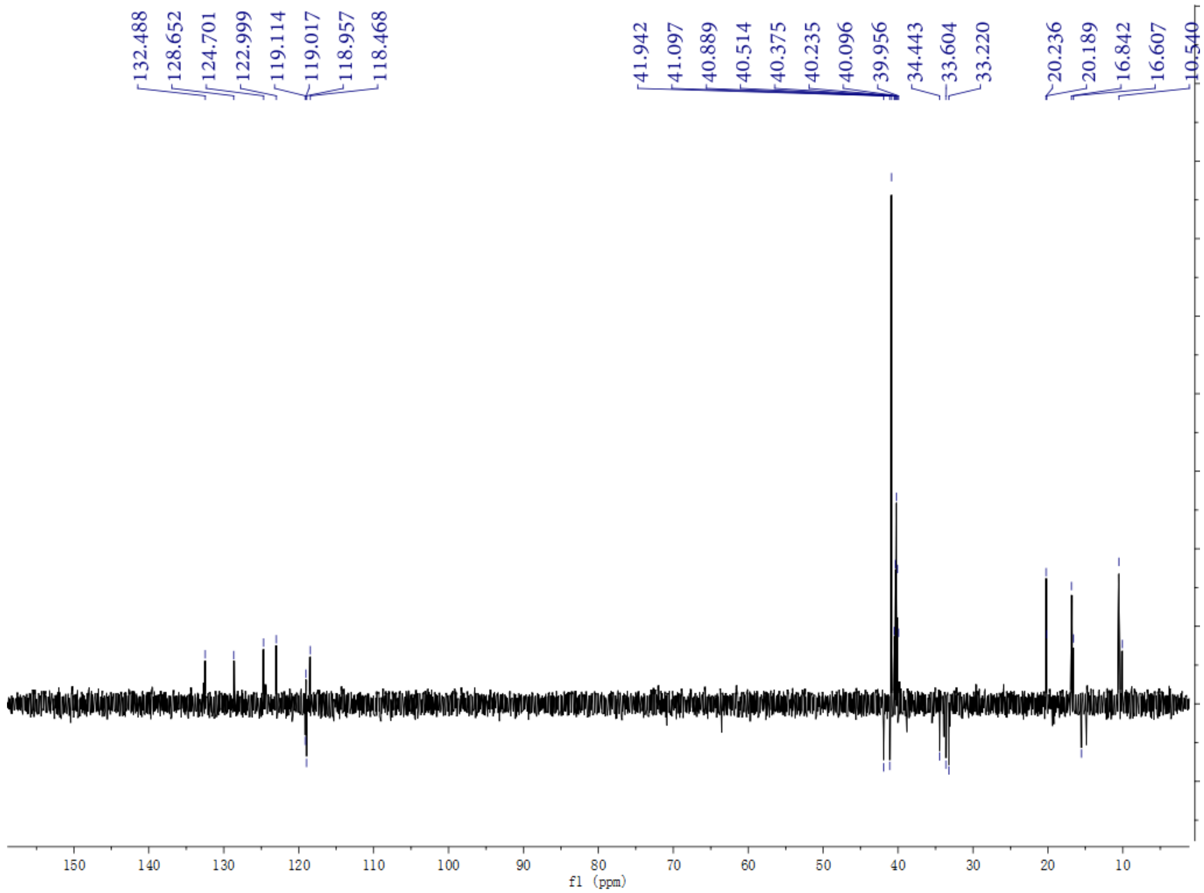

Supplementary Figure 113. DEPT-135 spectrum of **S8** (DMSO-d_6).

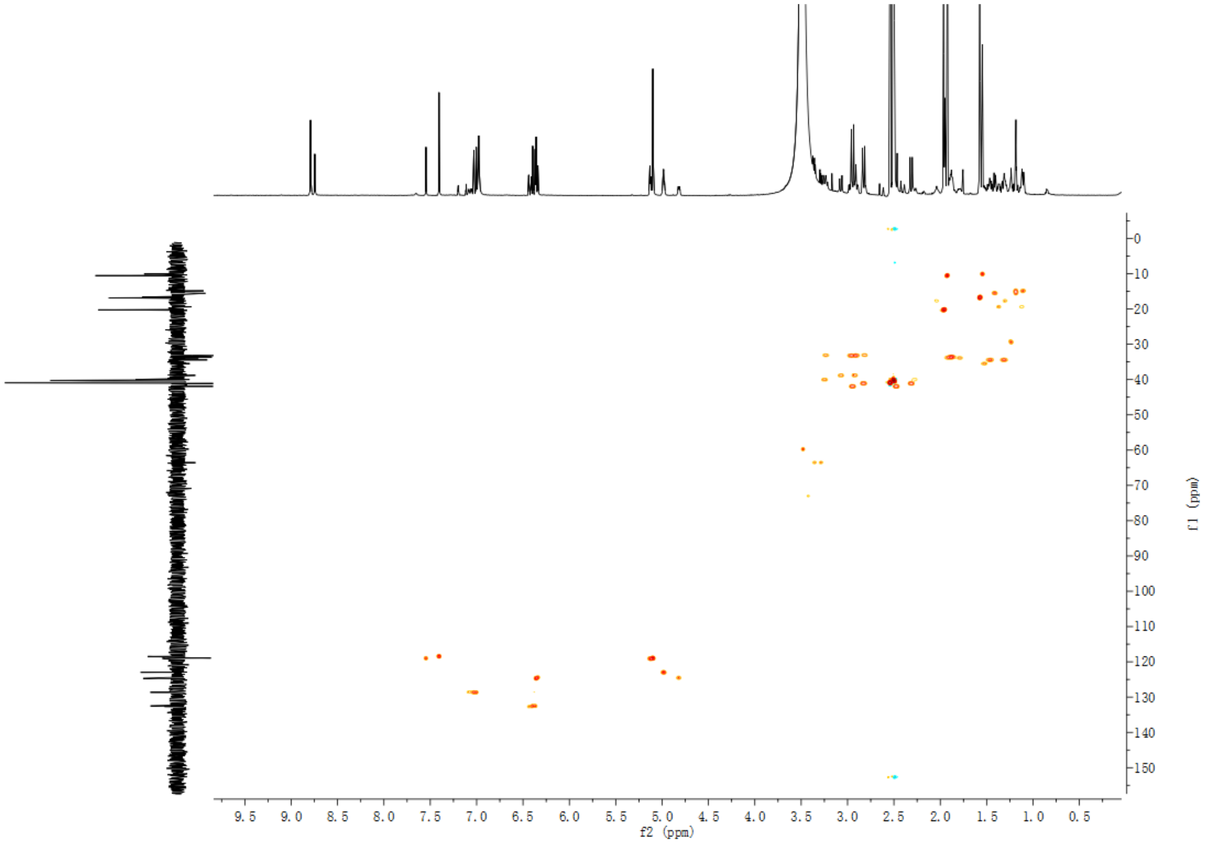

Supplementary Figure 114. HSQC spectrum of **S8** (DMSO-d_6).

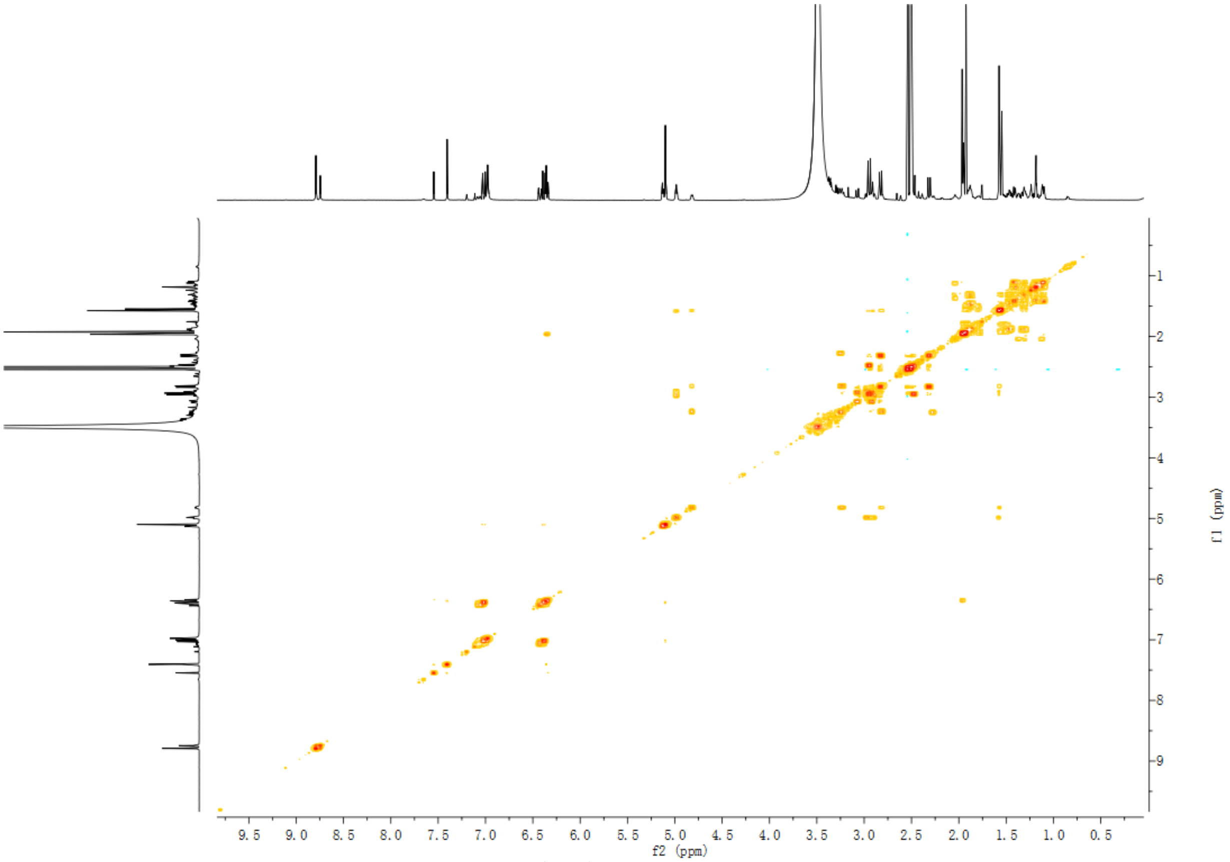

Supplementary Figure 115. ^{1}H-^{1}H COSY spectrum of **S8** (DMSO-d_6).

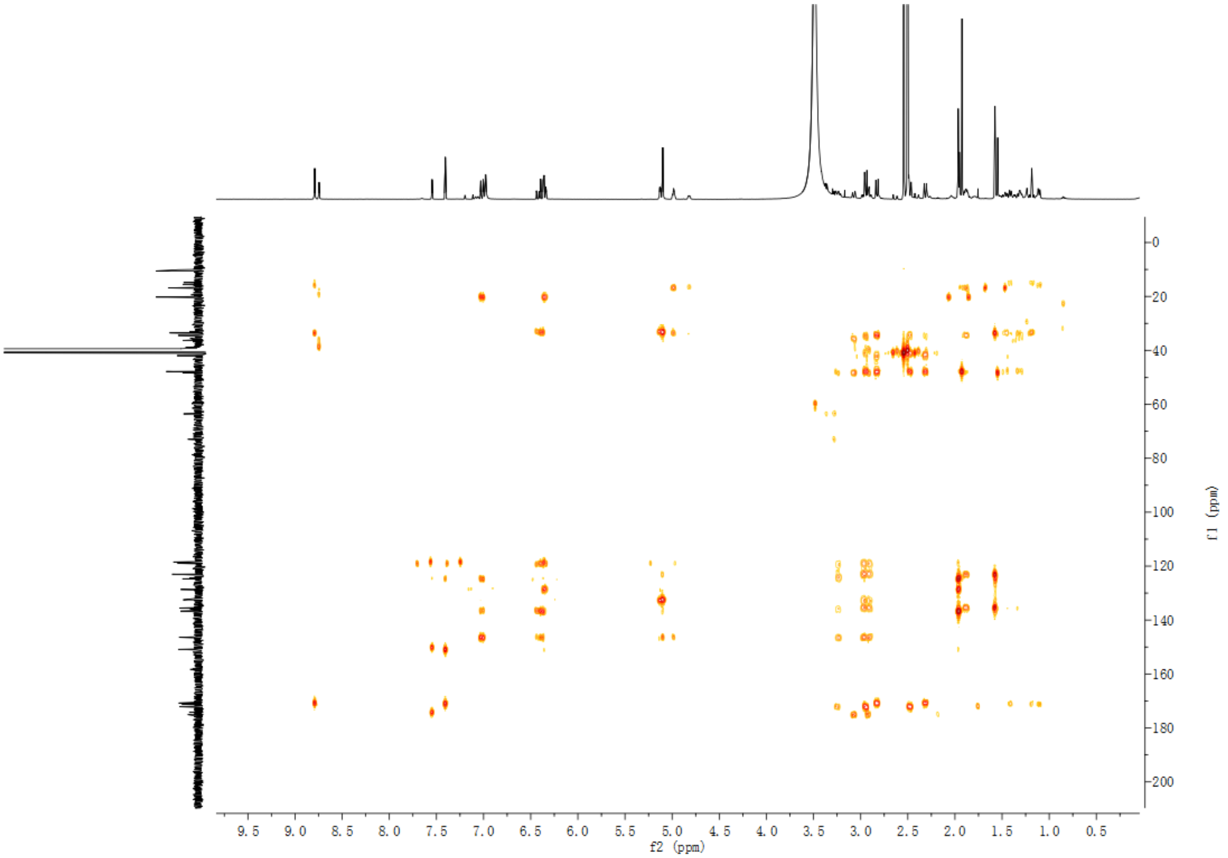

Supplementary Figure 116. HMBC spectrum of **S8** (DMSO-d_6).

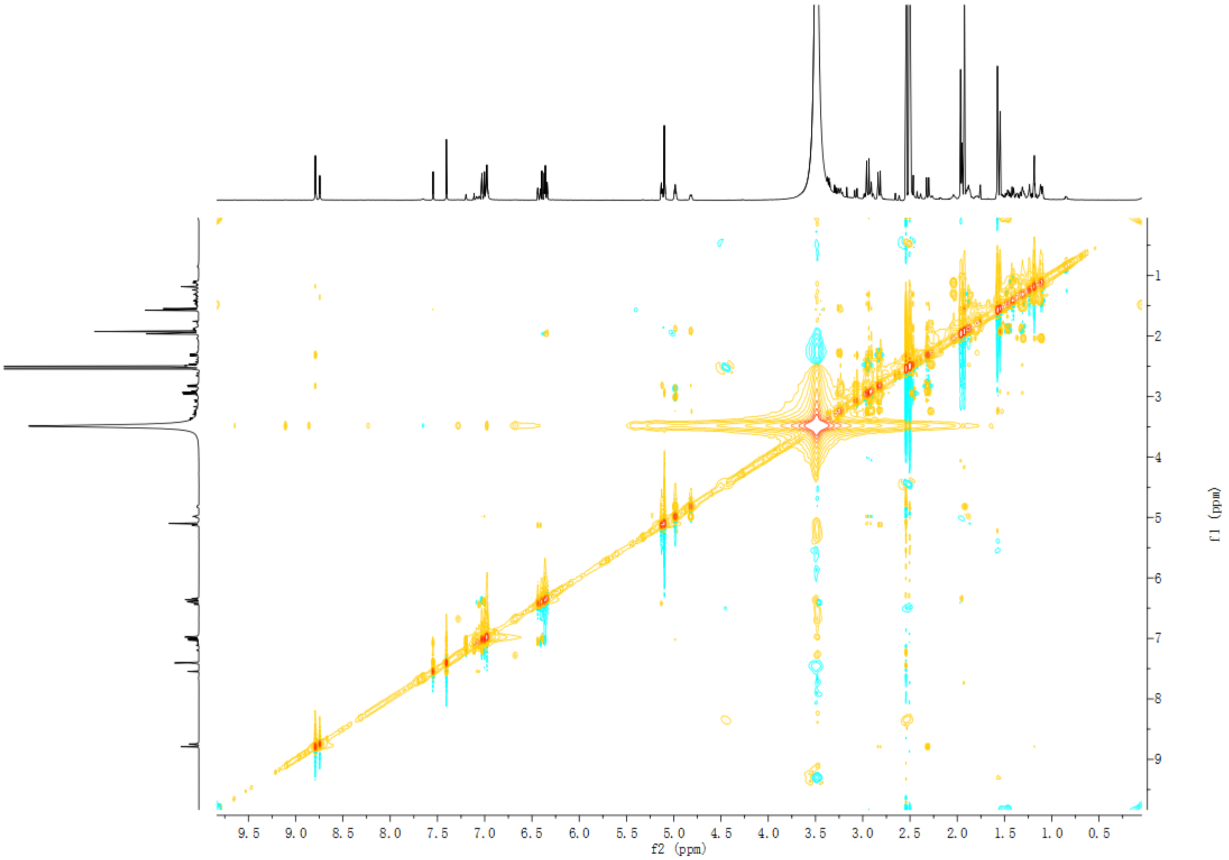

Supplementary Figure 117. ROESY spectrum of **S8** (DMSO-d_6).

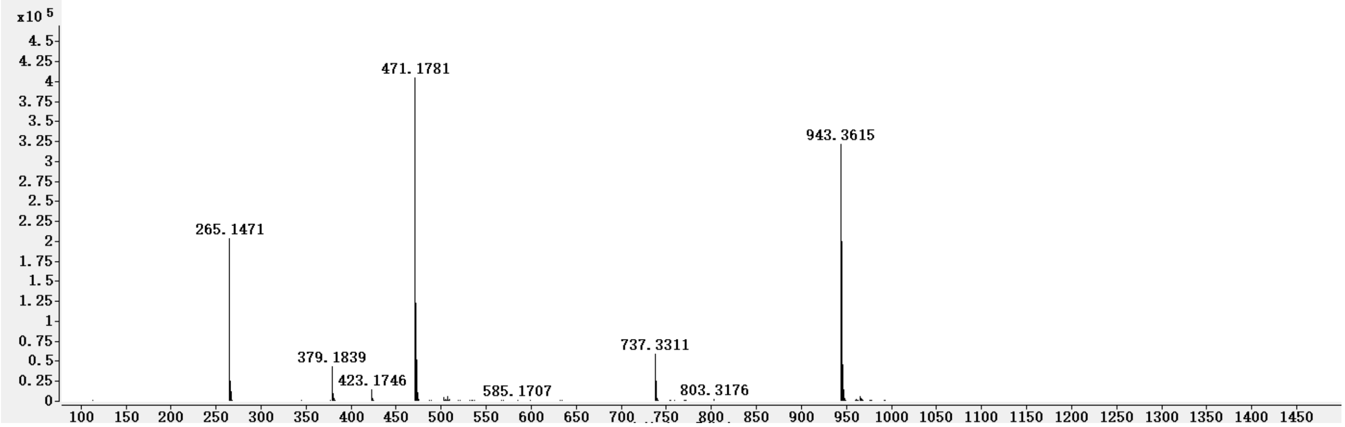

Supplementary Figure 118. HR-MS (ESI) spectrum of **S8**.

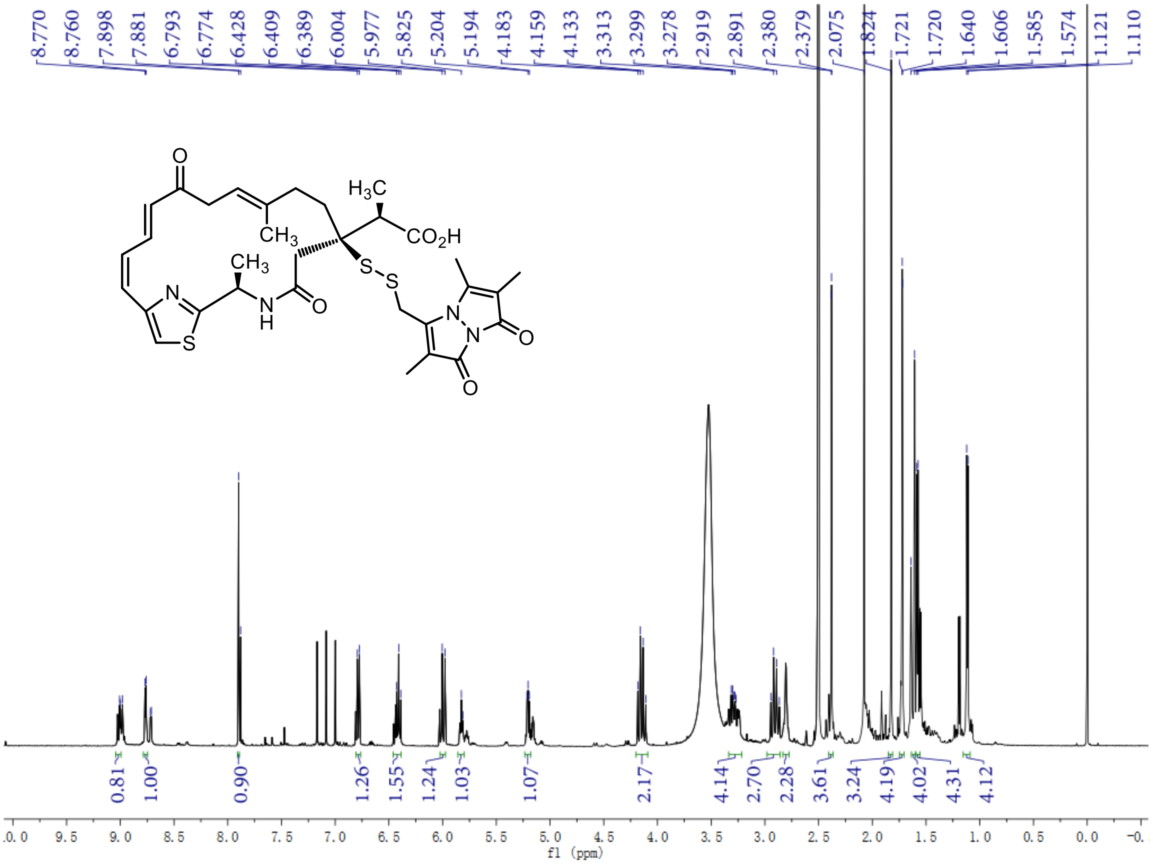

Supplementary Figure 119. ^{1}H NMR spectrum of **S9** (600 MHz, DMSO-d_6).

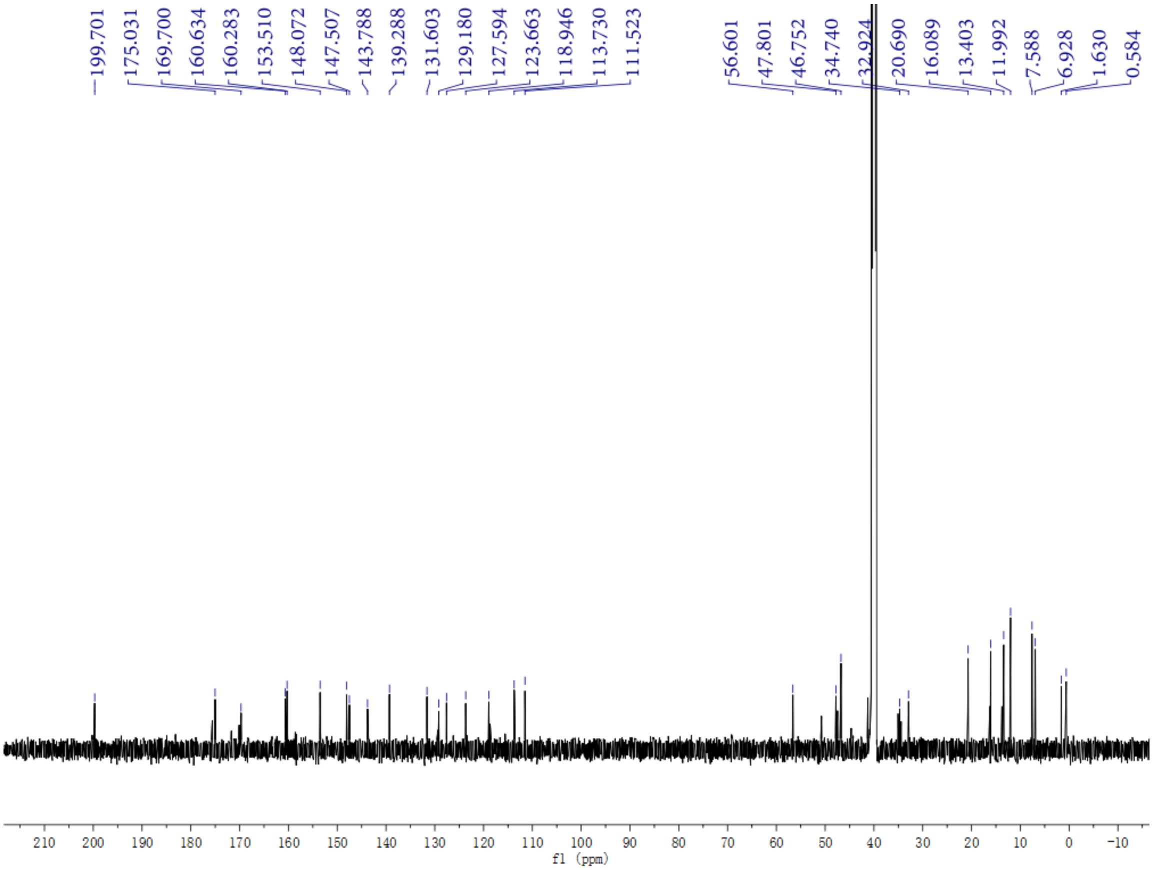

Supplementary Figure 120. ^{13}C NMR spectrum of **S9** (150 MHz, DMSO-d_6).

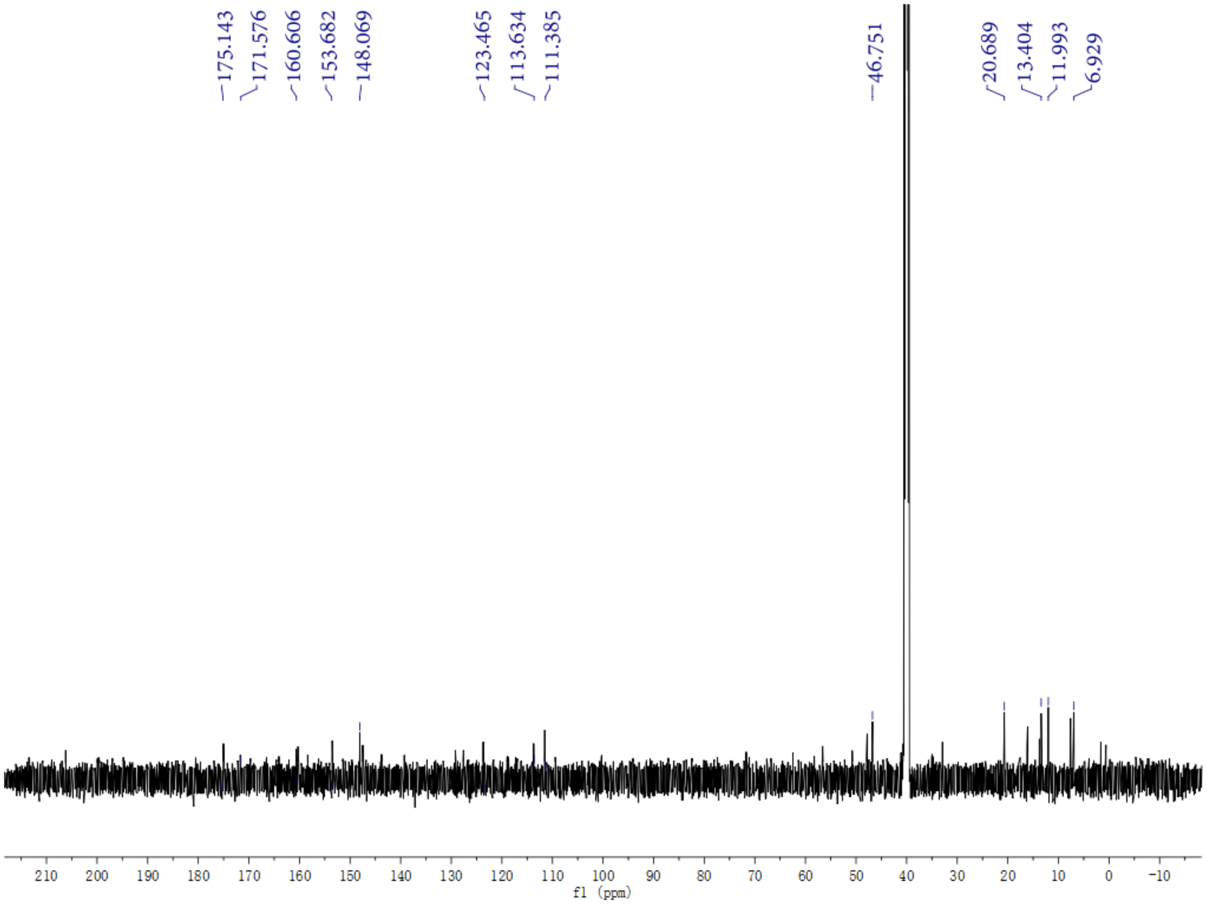

Supplementary Figure 121. DEPT-135 spectrum of **S9** (DMSO-d_6).

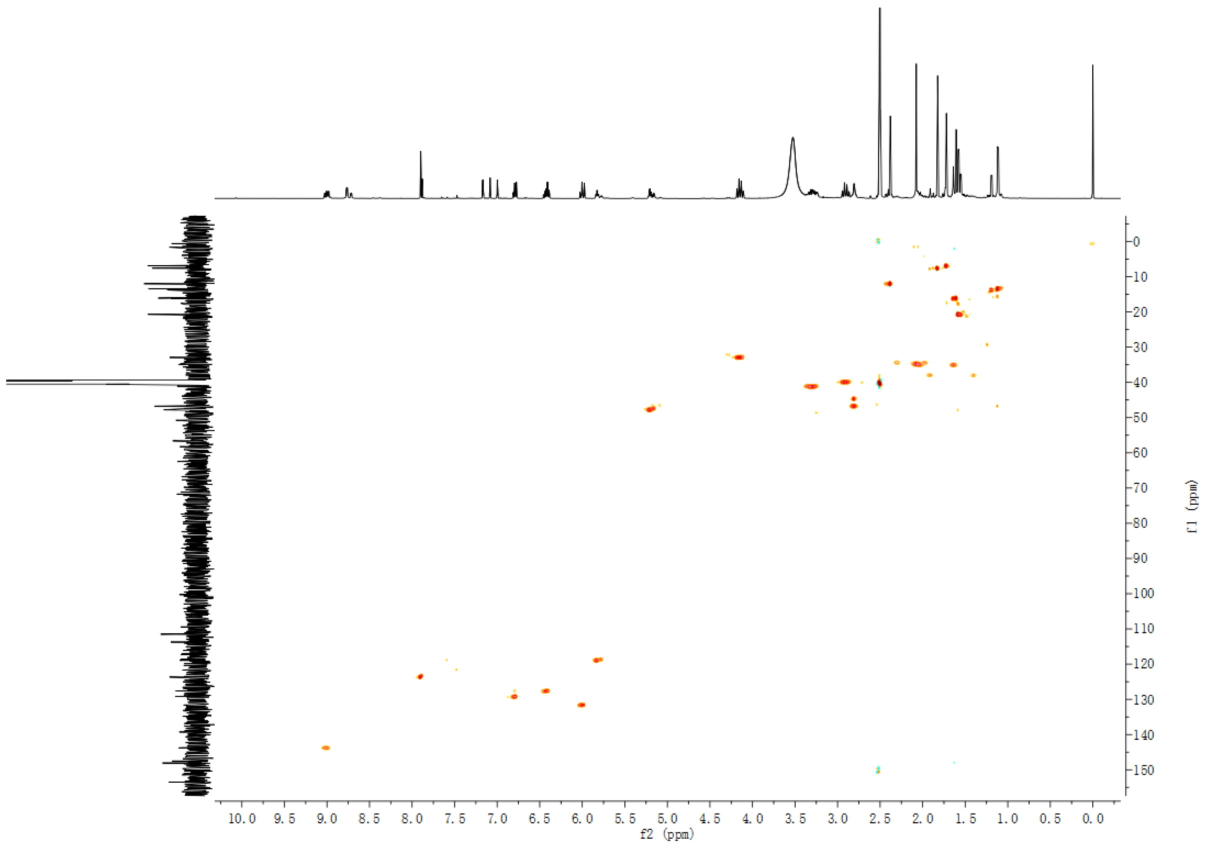

Supplementary Figure 122. HSQC spectrum of **S9** (DMSO-d_6).

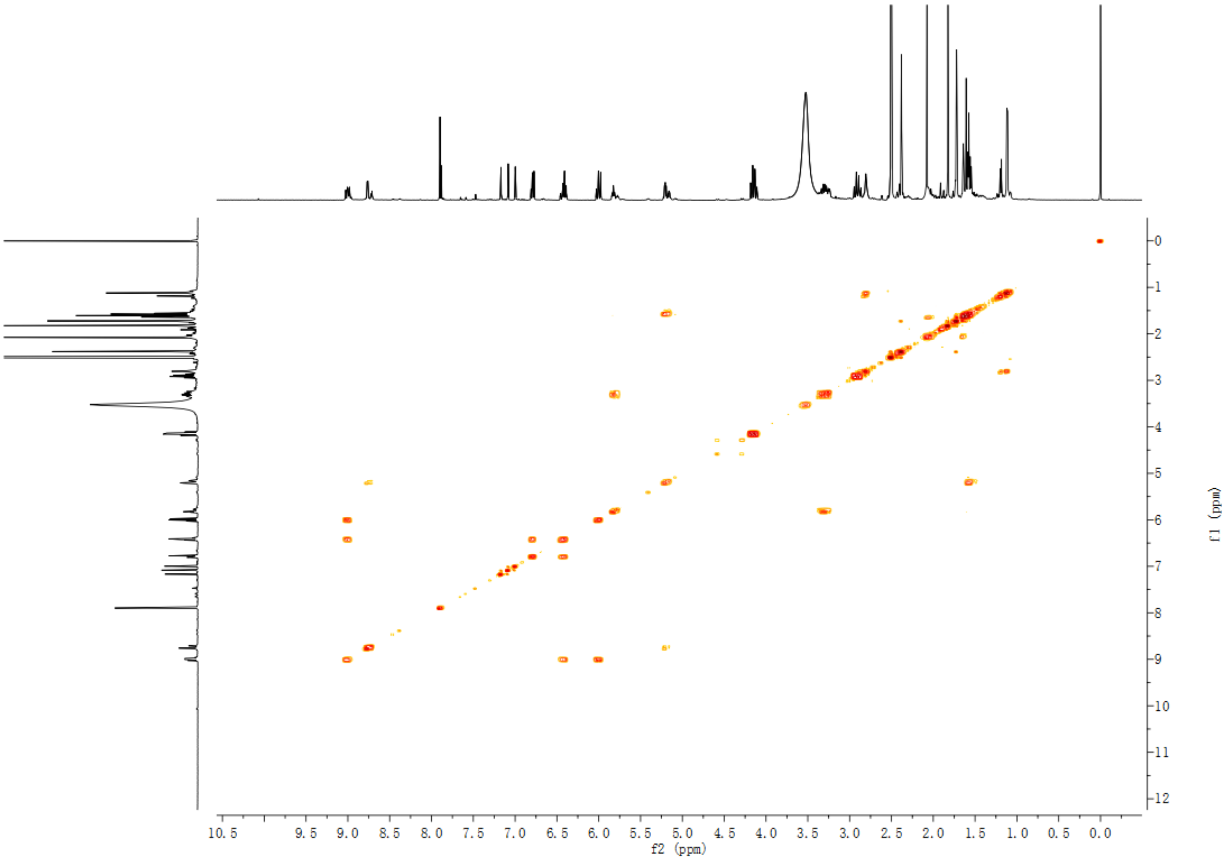

Supplementary Figure 123. ^{1}H-^{1}H COSY spectrum of **S9** (DMSO-d_6).

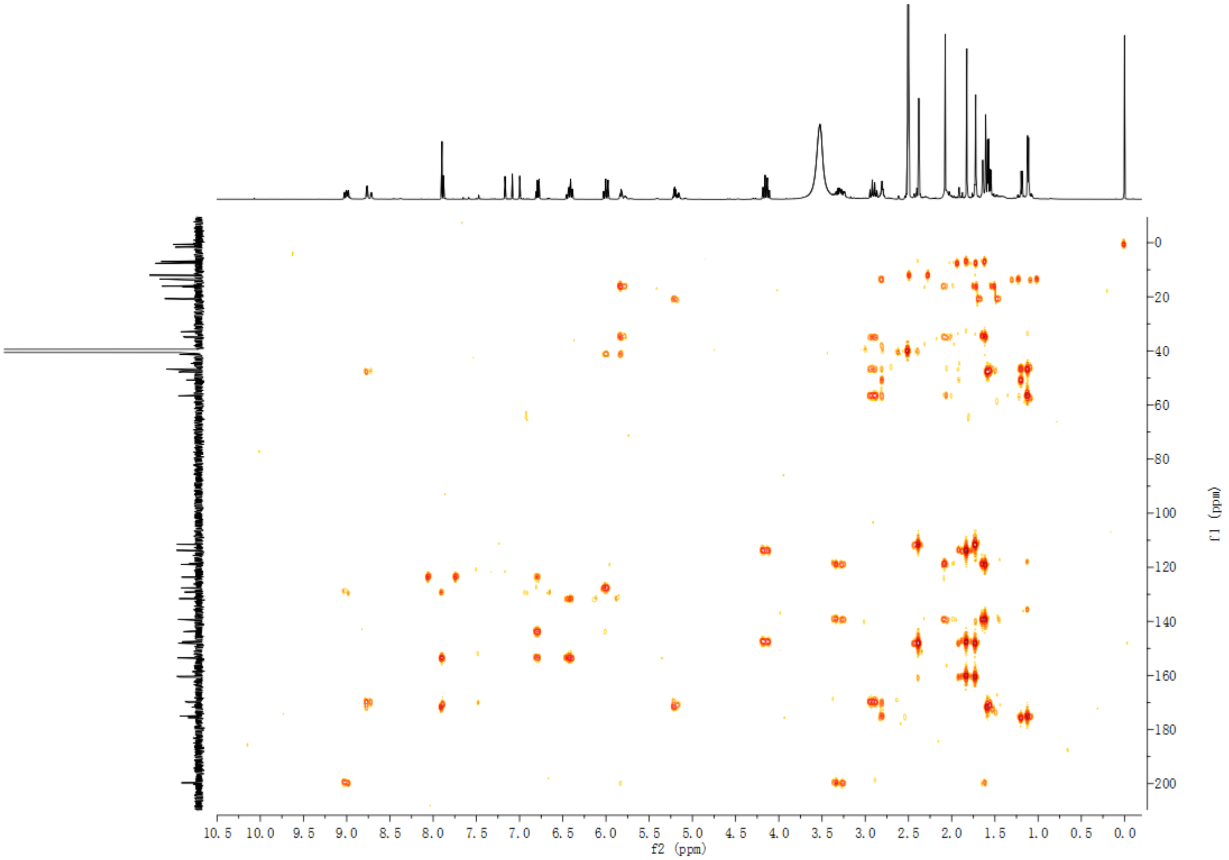

Supplementary Figure 124. HMBC spectrum of **S9** (DMSO-d_6).

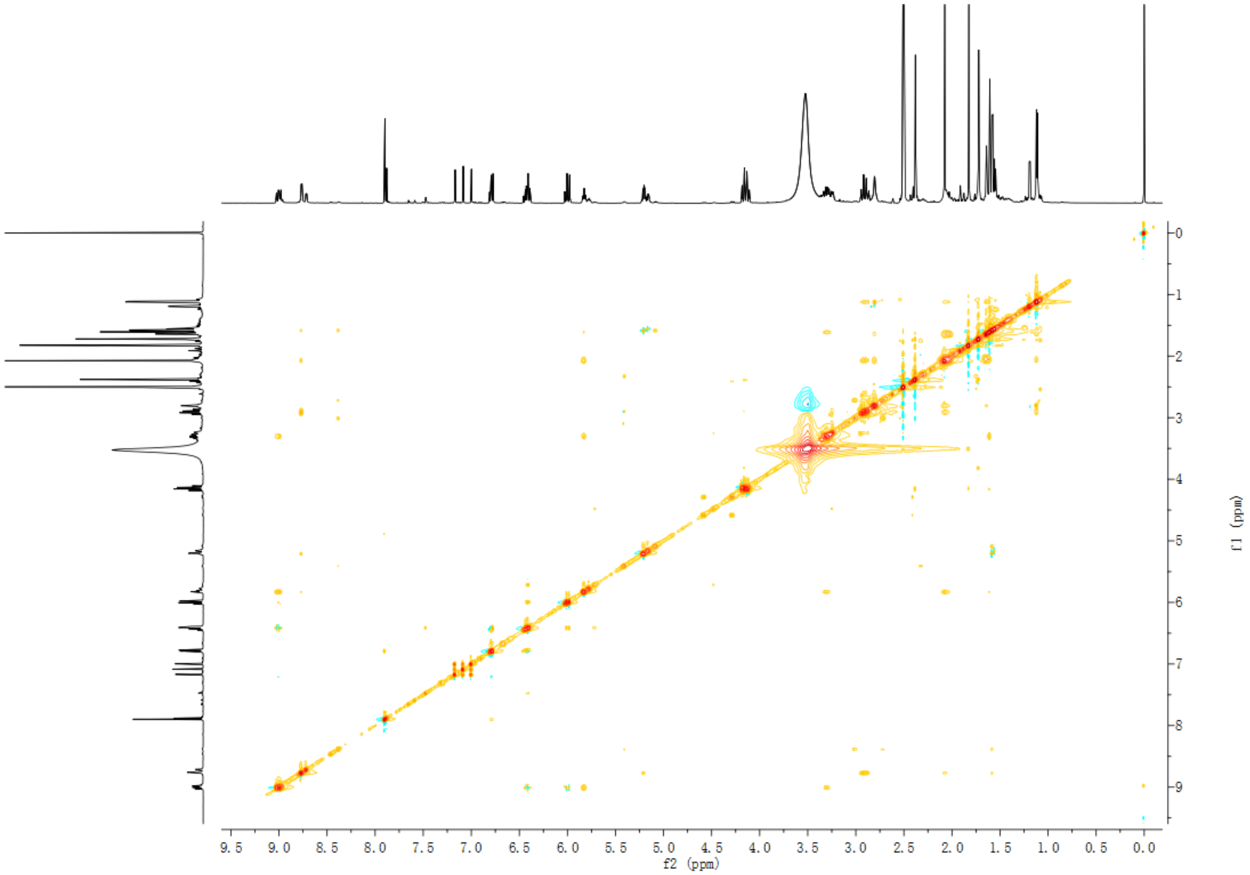

Supplementary Figure 125. ROESY spectrum of **S9** (DMSO-d_6).

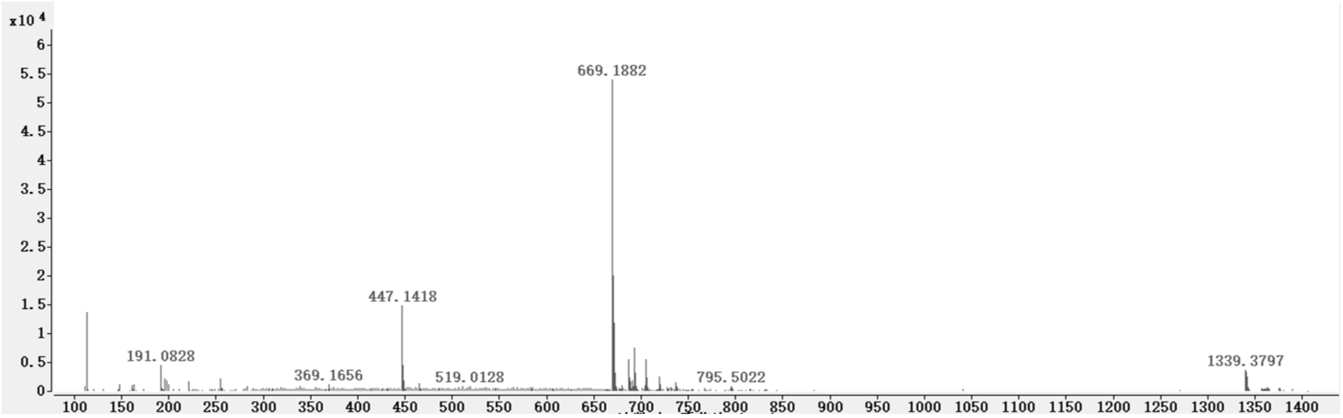

Supplementary Figure 126. HR-MS (ESI) spectrum of **S9**.

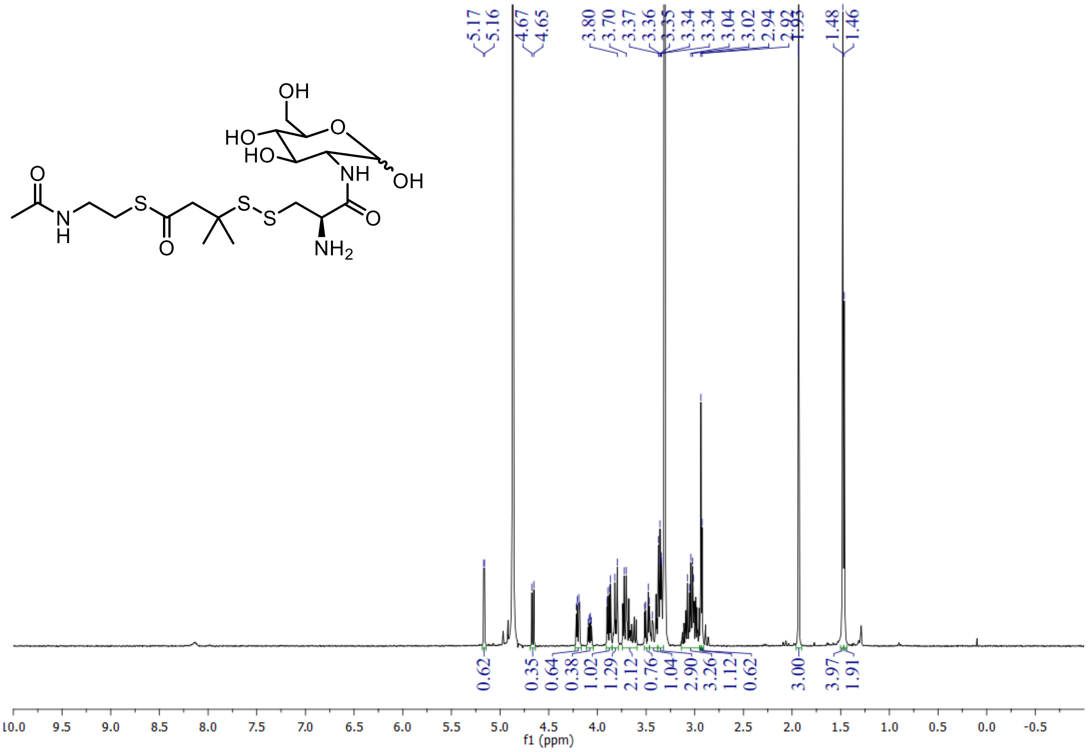

Supplementary Figure 127. ^{1}H NMR spectrum of **S15** (400 MHz, methanol-d_4).

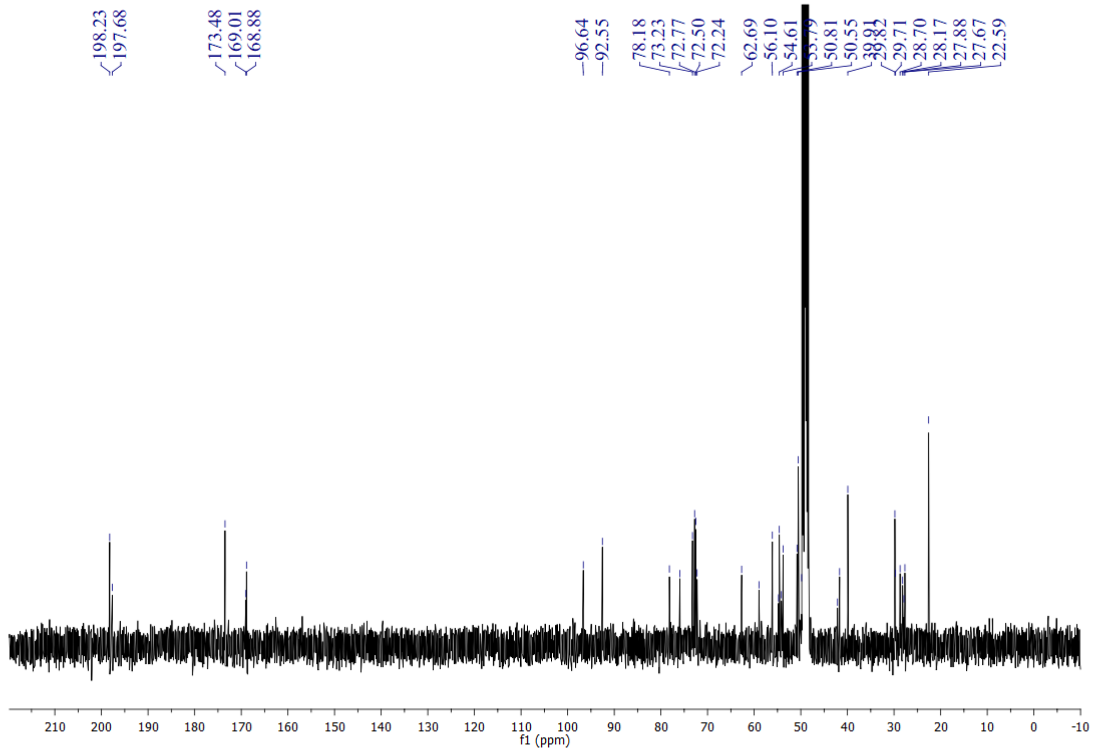

Supplementary Figure 128. ^{13}C NMR spectrum of **S15** (100 MHz, methanol-d_4).

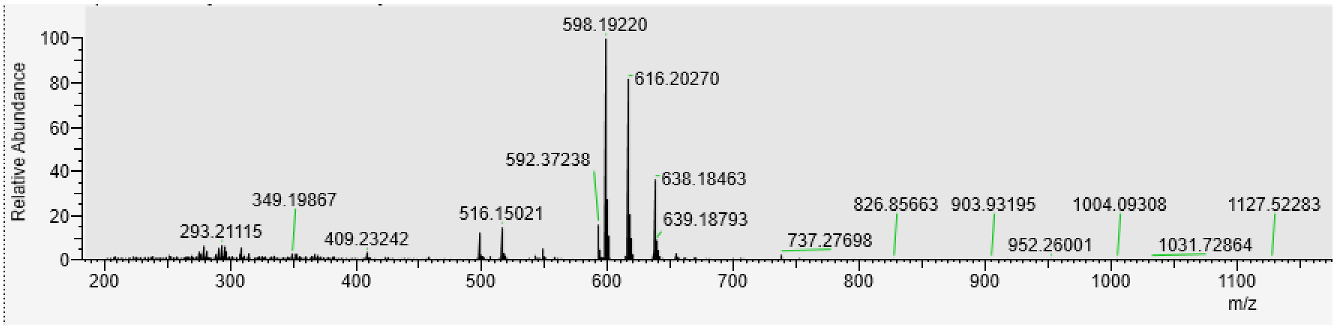

Supplementary Figure 129. HR-MS (ESI) spectrum of **S15**.

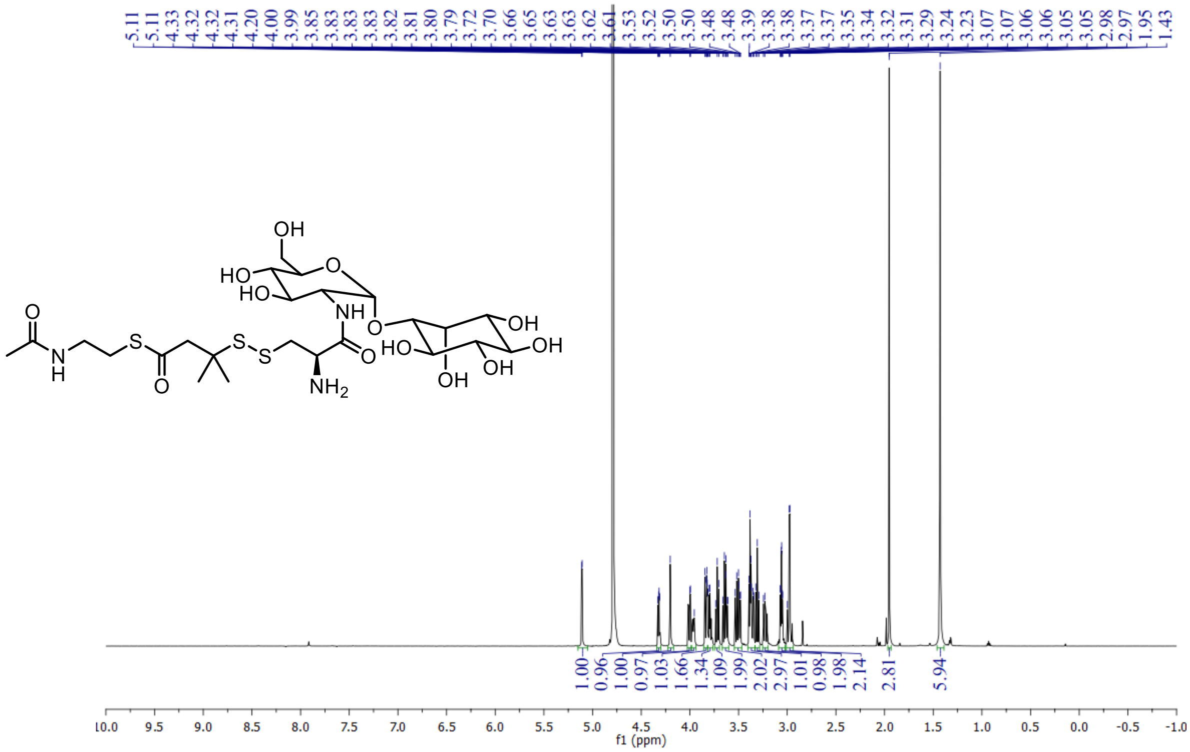

Supplementary Figure 130. ^{1}H NMR spectrum of **S16** (600 MHz, D_2O).

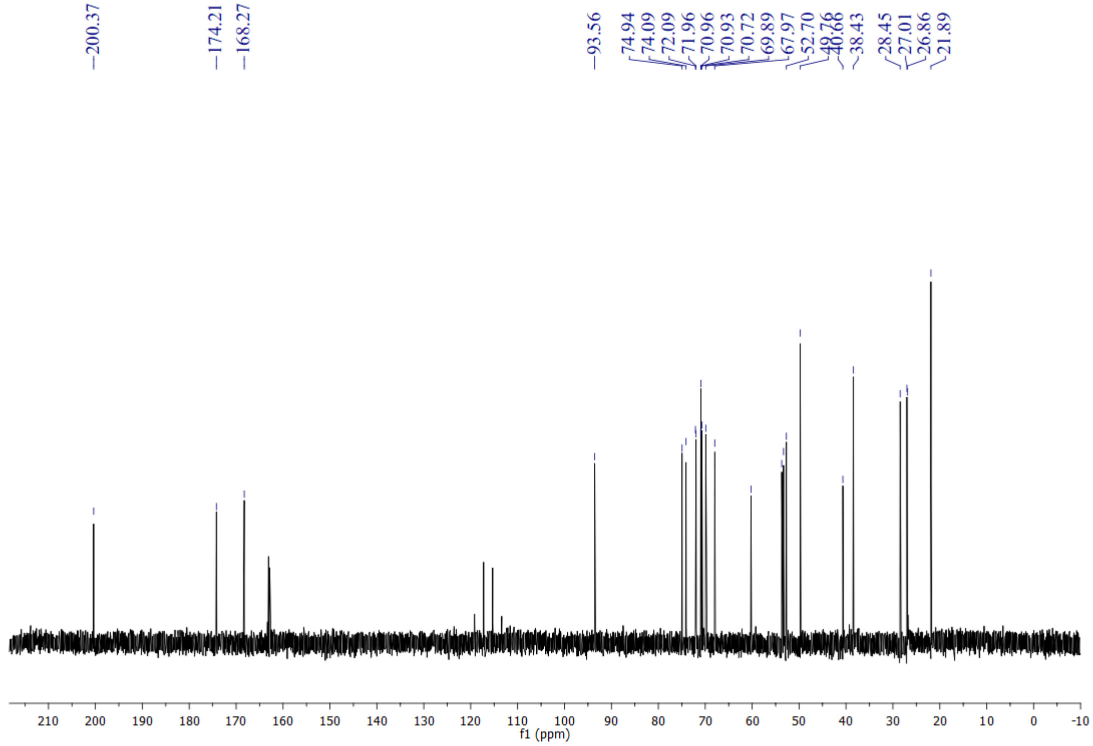

Supplementary Figure 131. ^{13}C NMR spectrum of **S16** (150 MHz, D_2O).

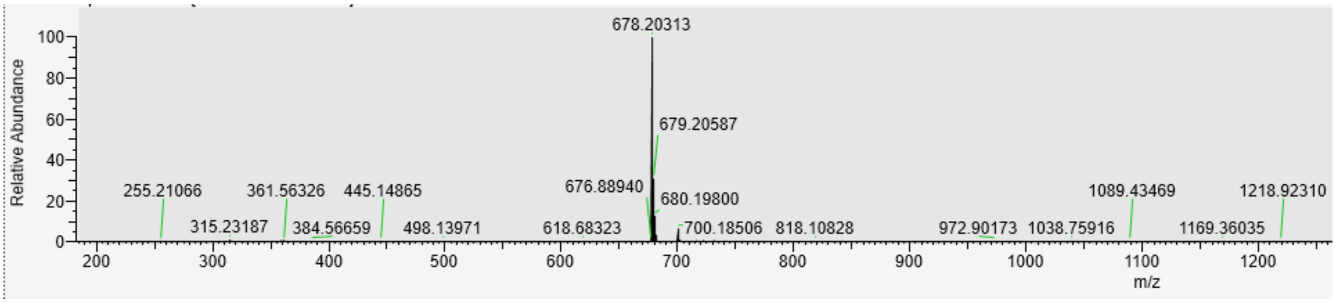

Supplementary Figure 132. HR-MS (ESI) spectrum of **S16**.

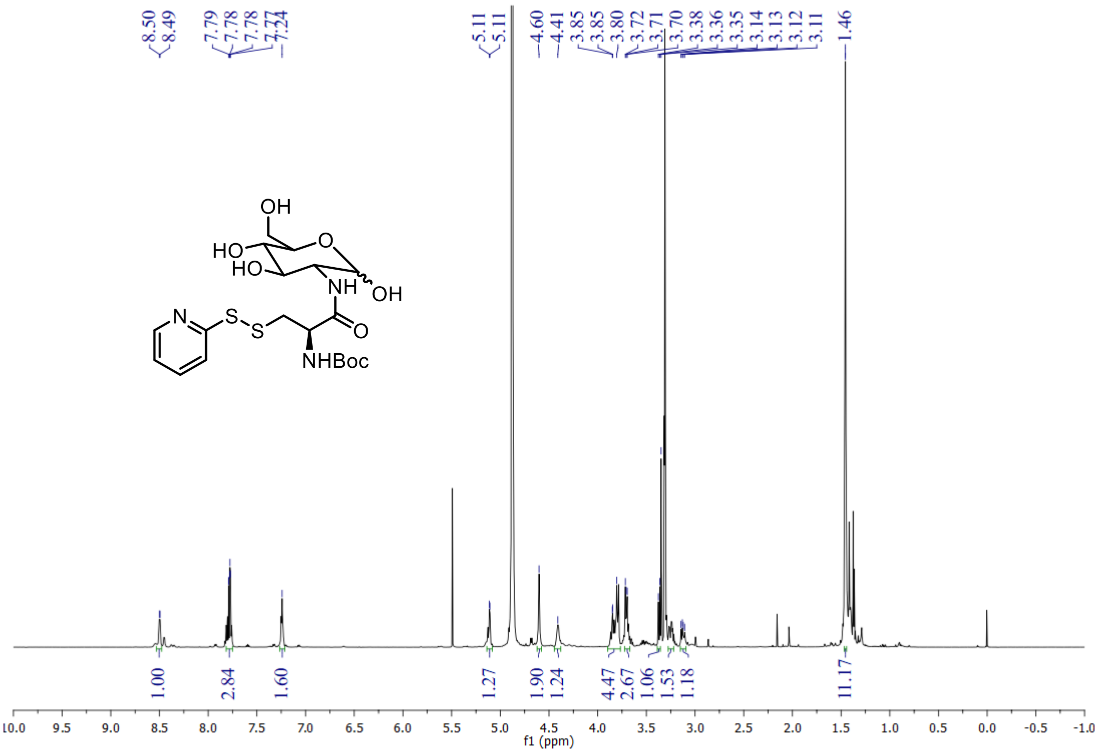

Supplementary Figure 133. ^{1}H NMR spectrum of **S17** (400 MHz, methanol-d_4).

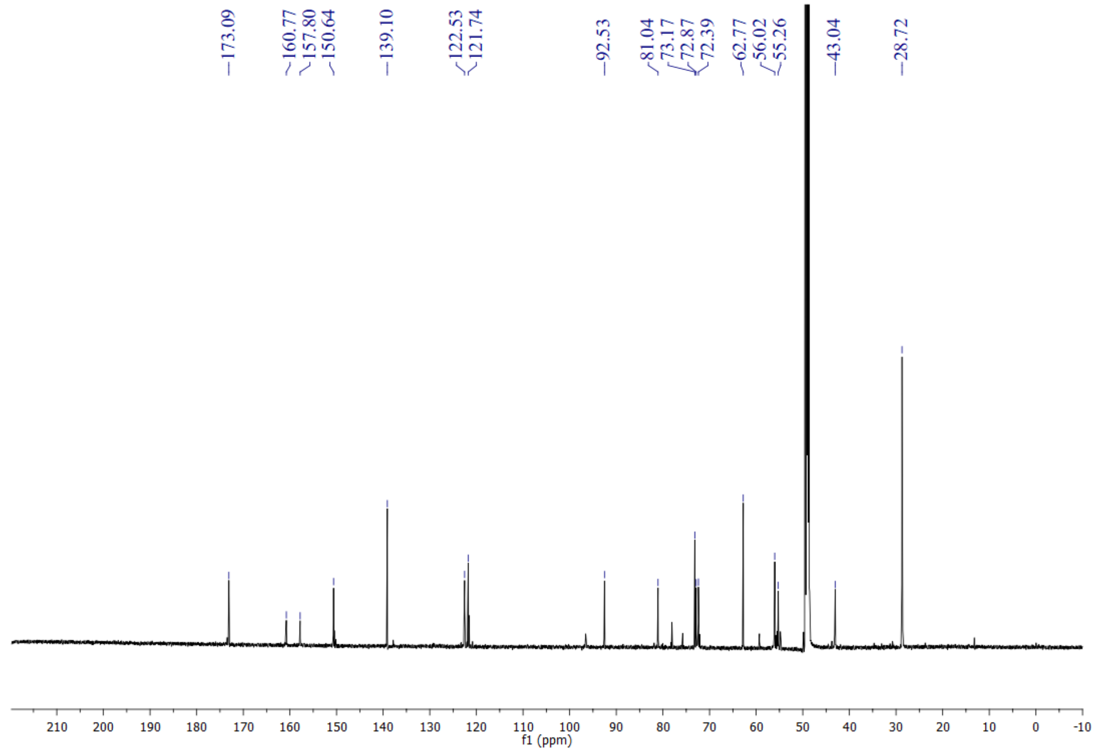

Supplementary Figure 134. ^{13}C NMR spectrum of **S17** (150 MHz, methanol-d_4).

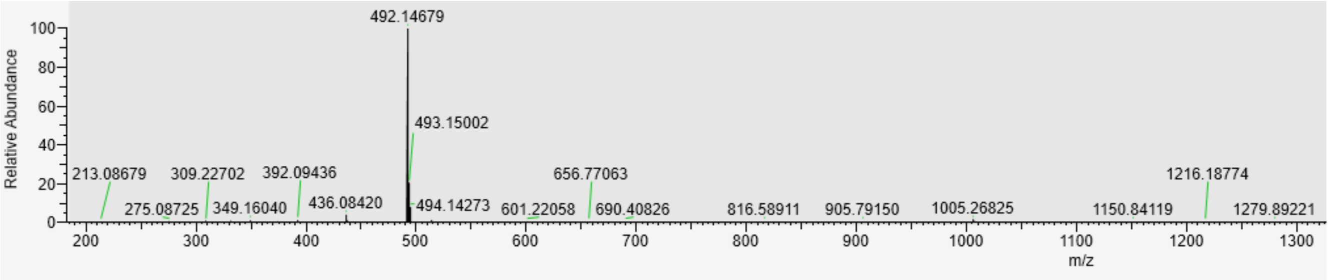

Supplementary Figure 135. HR-MS (ESI) spectrum of **S17**.

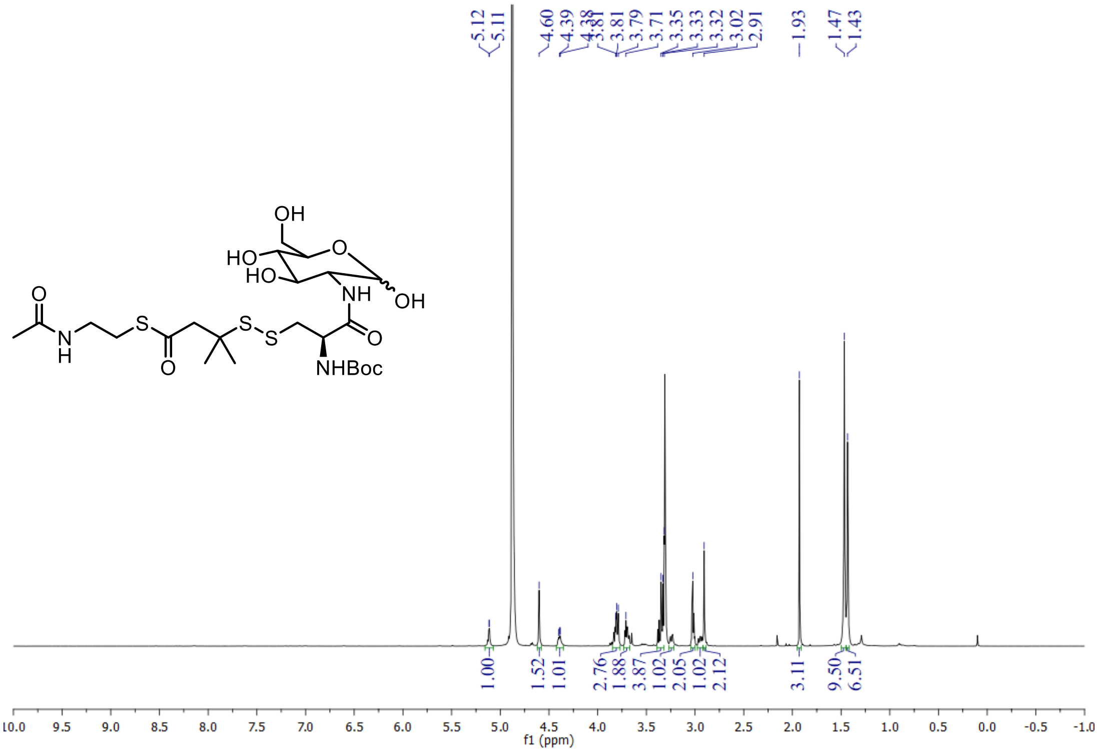

Supplementary Figure 136. ^{1}H NMR spectrum of **S18** (600 MHz, methanol-d_4).

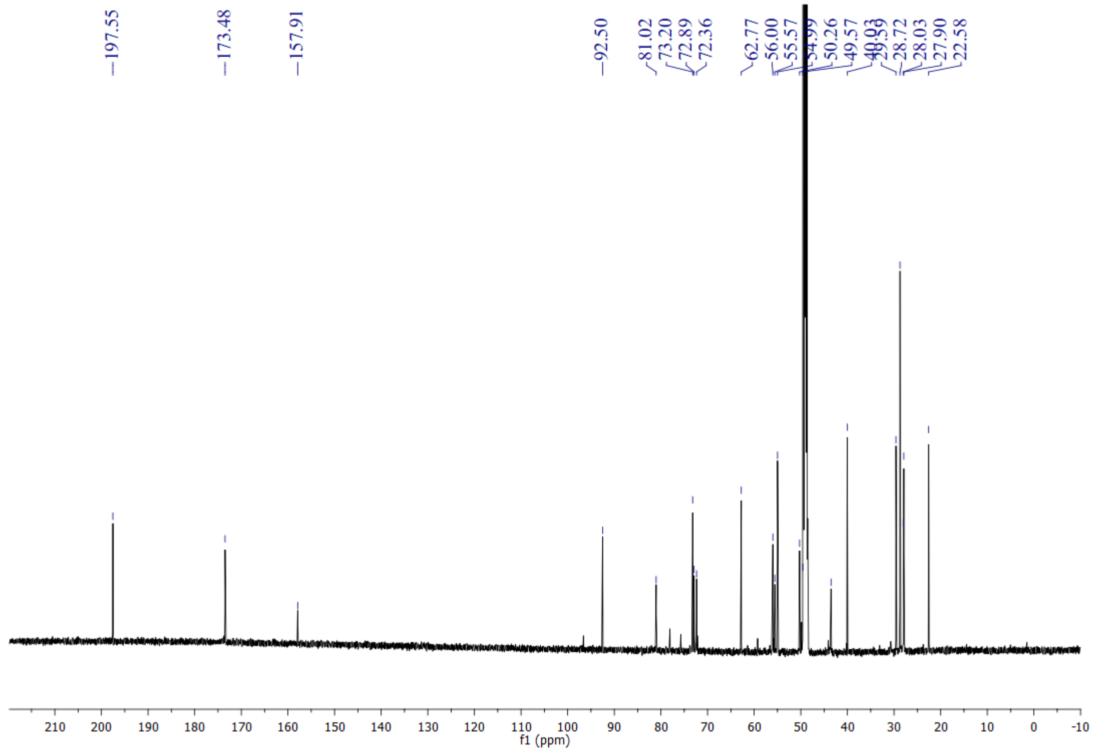

Supplementary Figure 137. ^{13}C NMR spectrum of **S18** (150 MHz, methanol-d_4).

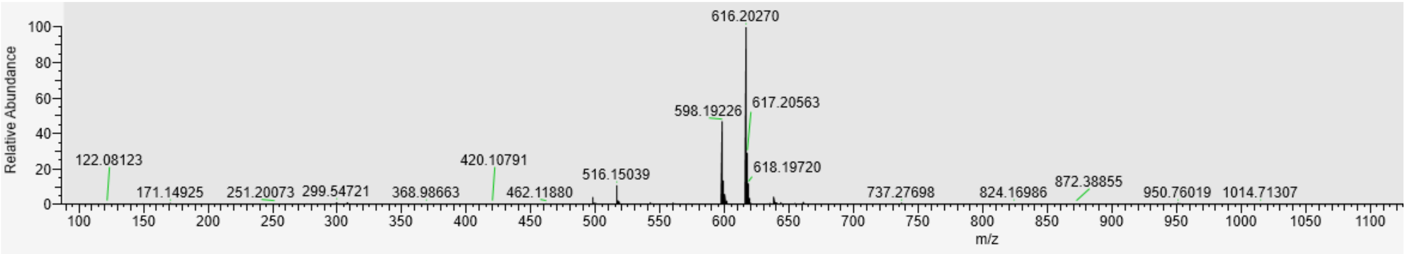

Supplementary Figure 138. HR-MS (ESI) spectrum of **S18**.

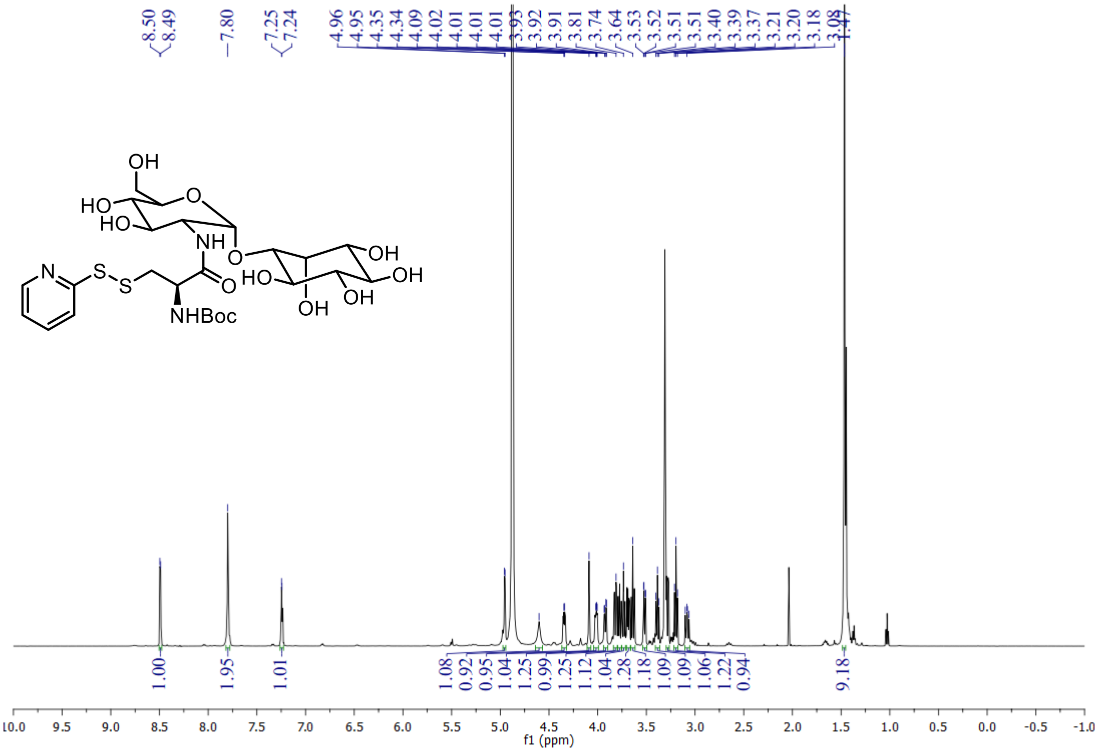

Supplementary Figure 139. ^{1}H NMR spectrum of **S19** (600 MHz, methanol-d_4).

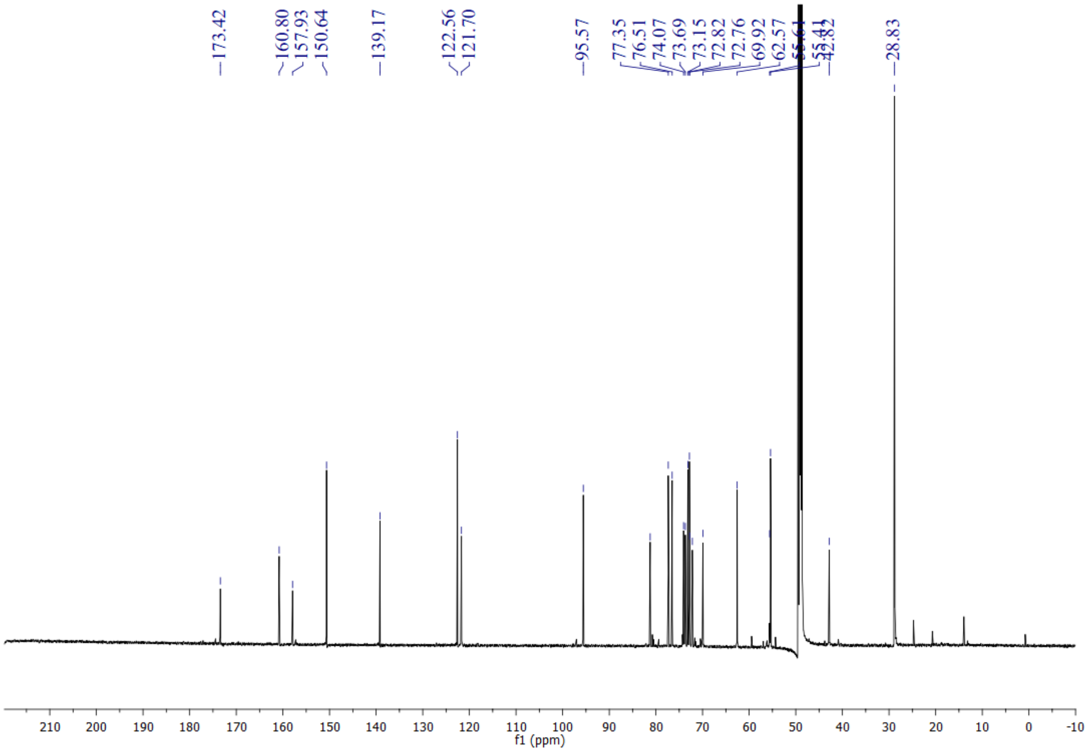

Supplementary Figure 140. ^{13}C NMR spectrum of **S19** (150 MHz, methanol-d_4).

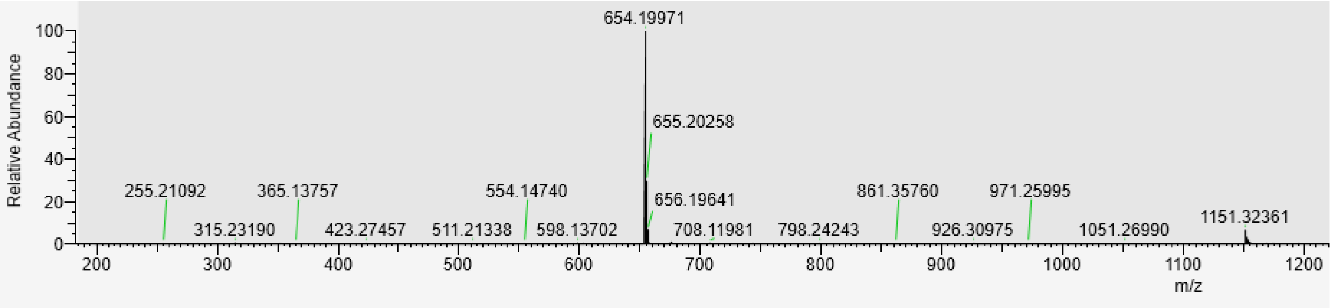

Supplementary Figure 141. HR-MS (ESI) spectrum of **S19**.

Supplementary Tables 1-9

Supplementary Table 1. Strains used in this study.

Strain	Genotype, Description	Source (Reference)
E. coli DH5α	*E. coli* host for general cloning	Life Technologies
E. coli BL21(DE3)	*E. coli* host for protein production	Life Technologies
E. coli ET12567/pUZ8002	Methylation-deficient *E. coli* host for intergeneric conjugation	(*10*)
Streptomyces sp. CB01883	Producing strain of guangnanmycins (GNM), wild-type	(*8*)
SB21007	A Δ*gnmP* in-frame deletion mutant strain of CB01883	This study
SB21008	The Δ*gnmP* mutant strain SB21007 harboring *gnmP* expressing plasmid pBS21020	This study
Streptomyces sp. CB02120-2	Producing strain of weishanmycins (WSM), wild-type	(*8*)
Streptomyces sp. CB01635	Alternative producer of leinamycin (LNM), wild-type	(*8*)

Supplementary Table 2. Plasmids and cosmids used in this study.

Plasmid	Description	Source (Reference)
pOJ260	*E. coli-Streptomyces* shuttle vector, AmR	(*11*)
pBS21001	Cosmid 1C4 containing partial *gnm* gene cluster	(*8*)
pBS21003	A pSETTurdR derived plasmid containing the promoter *KasO**, AmR	(*8*)
pBS21019	A pOJ260 derived plasmid for generating the in-frame deletion of *gnmP* in CB01883	This study
pBS21020	A pBS21003 derived plasmid for over-expression of *gnmP*	This study
pBS3080	A pRSFDuet-1 derived plasmid containing a *Bsm*FI site for ligation-independent cloning (LIC) and encodes a TEV protease site after the N-terminal His_6-tag	(*12*)
pBS21021	pBS3080 harboring *gnmP*; used for enzyme assays	This study
pBS21022	pBS3080 harboring *gnmT-SH*; used for enzyme assays	This study
pBS22010	pBS3080 harboring *wsmR-SH*; used for enzyme assays	This study
pBS21023	pBS3080 harboring *cb01883-CGL*; used for enzyme assays	This study
pBS22011	pBS3080 harboring *cb02120-2-CGL*; used for enzyme assays	This study
pBS3168	pBS3080 harboring *cb01635-CGL*; used for enzyme assays	This study

Supplementary Table 3. Primers used in this study.

Primer	Nucleotide Sequence (5'-3')	Function
pOJ-1883orf131L-del-F	TAAAACGACGGCCAGTGCCAACGGCGGATCAGGTGTGG	Inactivation of *gnmP*
pOJ-1883orf131L-del-R	TAGAGTCGACCTGCAGCCCACAGCATCGGCAGCGACAG	Inactivation of *gnmP*
pOJ-1883orf131R2-del-F	GCTTGGGCTGCAGGTCGACTCTAGAAGTTCGCGCCGGACTACG	Inactivation of *gnmP*
pOJ-1883orf131R2-del-R	ACAGCTATGACATGATTACGGCGTCAGGCCATGGTGGT	Inactivation of *gnmP*
1883orf131ifdel-2F	GGTGGCGGAACTGATCACGG	Screen for *ΔgnmP* mutant strain
1883orf131ifdel-2R	TATCAGCTCGAAGGCGATCGC	Screen for *ΔgnmP* mutant strain
1883orf131-Southern-F	GAAGCAGCGGGCCGCCTCG	Southern blot analysis of SB21007
1883orf131-Southern-R	GCTCGCCGTGTACGAGGCG	Southern blot analysis of SB21007
1883orf131-kasO-F	AGCTACTAGTGTGCTCGGGGTGCTGAGC	Complementation of SB21007
1883orf131-kasO-R	CCGGAATTCGATCGCCTTCGAGCTGATAC	Complementation of SB21007
1883orf131-F	AAAACCTCTATTTCCAGTCGGTGACCGAGGCCAAGGCCTTC	*gnmP* amplification for pBS21021
1883orf131-R	TACTTACTTAAATGTTATCACCGCTTGGCGTAGACGCTG	*gnmP* amplification for pBS21021
GnmT-SH-F	AAAACCTCTATTTCCAGTCGCGGCGTACGTCCGGGGACGGC	*gnmT-SH* amplification for pBS21022
GnmT-SH-R	TACTTACTTAAATGTTAGGGCTCCTCCTCCAGTGCCCGG	*gnmT-SH* amplification for pBS21022
WsmR-SH-F	AAAACCTCTATTTCCAGTCGGCCGGCACCCCCGCCG	*wsmR-SH* amplification for pBS22010
WsmR-SH-R	TACTTACTTAAATGTTAGTAGCCCTCGGCGAGCGCGGC	*wsmR-SH* amplification for pBS22010
CB01883-CGL-F	AAAACCTCTATTTCCAGTCGATGAGCGACGCGACCAGCAAC	*cb01883-CGL* amplification for pBS21023
CB01883-CGL-R	TACTTACTTAAATGTTATCACTCGGCGGACGCGTCCAG	*cb01883-CGL* amplification for pBS21023
CB01635-CGL-F	AAAACCTCTATTTCCAGTCGATGACTCAATCGACGCAGATC	*cb01635-CGL* amplification for pBS3169
CB01635-CGL-R	TACTTACTTAAATGTTACTAGACGGCCGCCTCGTCGAGC	*cb01635-CGL* amplification for pBS3169
CB02120-2-CGL-F	AAAACCTCTATTTCCAGTCGGTGACCGACCGCACCCCAGAC	*cb02120-2-CGL* amplification for pBS22011
CB02120-2-CGL-R	TACTTACTTAAATGTTATCAGCCGGCCGGGCCCGCCGCGTCCAG	*cb02120-2-CGL* amplification for pBS22011

Supplementary Table 4. ^{1}H NMR (600 MHz) and ^{13}C NMR (150 MHz) data of **9** in DMSO-d_6.[a] See Supplementary Fig. 13 for carbon numbering, and Supplementary Figs. 26 – 32 for spectra.

**No.**	**9 Major rotamer**		**9 Minor rotamer**	
	δ_C	δ_H	δ_C	δ_H
**1**	170.2		170.0	
**2**	41.1	2.86 (1H, m)<br>2.44 (1H, m)	41.5	2.95 (1H, m)<br>2.62 (1H, m)
**3**	54.4		54.4	
**4**	34.6	1.62 (1H, m)<br>1.47 (1H, m)	35.8	1.54 (2H, m)
**6**	135.2		135.3	
**7**	123.5	4.98 (1H, t, 6.0)	124.8	4.87 (1H, d, 7.7)
**8**	33.1	3.29 (2H, m)	33.2	3.24 (1H, m)<br>2.82 (1H, m)
**9**	146.3		146.5	
**10**	132.6	6.40 (1H, d, 16.5)	132.6	6.42 (1H, d, 16.0)
**11**	128.5	7.02 (1H, d, 16.5)	128.8	7.11 (1H, d, 16.0)
**12**	136.7		136.9	
**13**	124.6	6.36 (1H, s)	124.3	6.32 (1H, s)
**14**	150.9		150.3	
**15**	118.5	7.42 (1H, s)	118.8	7.55 (1H, s)
**16**	171.3		173.8	
**17**	33.6		36.0	
**18**	15.4	1.38 (2H, m)	19.2	1.38 (1H, m)<br>1.13 (1H, m)
**19**	15.0	1.20 (2H, m)	17.6	1.23 (2H, m)
**20**	20.3	1.97 (3H, s)	20.3	1.95 (3H, s)
**21**	119.0	5.11 (2H, m)	118.9	5.11 (2H, m)
**22**	16.8	1.58 (3H, s)	16.7	1.59 (3H, s)
**23**	41.5	2.95 (1H, m)<br>2.62 (1H, d, 15.5)	39.1	3.08 (1H, m)<br>2.96 (1H, m)
**24**	171.9		174.5	
**25**	40.0	3.18 (2H, m)	39.3	2.86 (2H, m)
**26**	52.0	4.16 (1H, m)	51.5	4.04 (1H, m)
**27**	169.8		170.0	
**NH**		8.96 (1H, s)		8.82 (1H, s)
**NH_2**		8.42 (2H, m)		8.38 (2H, m)

[a] Signals were assigned with aid of ^{1}H-^{1}H COSY, HMBC, and ROESY experiments.

Supplementary Table 5. ^{1}H NMR (600 MHz) and ^{13}C NMR (150 MHz) data of **11** and **S9** in DMSO-d_6.[a] See Supplementary Fig. 10 for carbon numbering, and Supplementary Figs. 34 – 38 and Supplementary Figs. 119 – 125 for spectra.

**No.**	**11**[b]	
	δ_C	δ_H
**1**	170.4	
**2**	41.4	2.89 (1H, d, 14.4)<br>2.51 (1H, m)
**3**	54.5	
**4**	34.9	1.63 (1H, m)<br>1.52 (1H, m)
**5**	33.8	1.85 (2H, m)
**6**	135.3	
**7**	123.5	4.99 (1H, t, 6.7)
**8**	32.8	2.95 (2H, m)
**9**	146.3	
**10**	132.6	6.40 (1H, d, 16.0)
**11**	128.5	7.07 (1H, d, 16.0)
**12**	136.5	
**13**	124.5	6.36 (1H, s)
**14**	151.2	
**15**	118.4	7.42 (1H, s)
**16**	171.3	
**17**	33.5	
**18**	15.6	1.36 (2H, m)
**20**	20.4	1.96 (3H, s)
**21**	119.1	5.11 (2H, m)
**23**	41.5	2.94 (1H, m)<br>2.63 (1H, d, 15.3)
**24**	171.9	
**NH**		8.93 (1H, s)
**1'**	160.1	
**2'**	114.2	
**3'**	147.0	
**4'**	148.1	
**5'**	111.6	
**6'**	160.6	
**7'**	7.6	1.80 (3H, s)
**8'**	32.9	4.13 (1H, d, 14.6)<br>4.09 (1H, d, 14.6)
**9'**	12.0	2.39 (3H, q, 0.7)
**10'**	6.9	1.72 (3H, q, 0.7)

[a] Signals were assigned with aid of ^{1}H-^{1}H COSY, HMBC, and ROESY experiments.
[b] Only the major rotamer is shown.

**No.**	**S9**[b]	
	δ_C[c]	δ_H
**1**	169.7	
**2**	39.9	2.88 (1H, d, 15.5)<br>2.93 (1H, d, 15.5)
**3**	56.6	
**4**	35.0	2.03 (1H, m)<br>1.64 (1H, m)
**5**	34.7	2.06 (2H, m)
**6**	139.2	
**7**	118.9	5.83 (1H, t, 8.1)
**8**	41.1	3.28 (2H, m)
**9**	199.7	
**10**	131.6	5.99 (1H, d, 16.4)
**11**	143.8	9.00 (1H, dd, 11.2, 16.4)
**12**	127.6	6.41 (1H, t, 11.1)
**13**	129.2	6.79 (1H, d, 11.2)
**13a**	153.5	
**14**	123.7	7.90 (1H, s)
**15**	171.7	
**16**	47.8	5.20 (1H, m)
**17**	20.7	1.58 (3H, d, 6.5)
**18**	16.1	1.61 (3H, s)
**3'**	175.0	
**4'**	46.9	2.80 (1H, m)
**5'**	13.4	1.11 (3H, d, 7.0)
**NH**		8.77 (1H, d, 5.4)
**1''**	160.3	
**2''**	113.6	
**3''**	147.4	
**4''**	148.1	
**5''**	111.4	
**6''**	160.6	
**7''**	7.6	1.82 (3H, s)
**8''**	32.9	4.17 (1H, d, 14.7)<br>4.12 (1H, d, 14.7)
**9''**	12.0	2.38 (3H, q, 0.7)
**10''**	6.9	1.72 (3H, q, 0.7)

[a] Signals were assigned with aid of ^{1}H-^{1}H COSY, HMBC, and ROESY experiments.
[b] Only the major rotamer is shown.
[c] ^{13}C chemical shifts determined from the indirect dimension of HSQC and HMBC spectra

Supplementary Table 6. ^{1}H NMR (600 MHz) and ^{13}C NMR (150 MHz) data of **13**, **14**, and **15** in DMSO-d_6.[a] See Supplementary Fig. 10 for carbon numbering, and Supplementary Figs. 40 – 46, 48 – 54, and 56 – 60 for spectra.

No.	13		14[b]		15	
	δ_C[c]	δ_H	δ_C	δ_H	δ_C	δ_H
**1**	170.2		169.9		170.2	
**2**	39.5	2.76 (1H, d, 15.1)<br>2.93 (1H, d, 15.1)	39.5	2.77 (1H, m)<br>2.90 (1H, m)	39.5	2.79 (1H, d, 14.8)<br>2.91 (1H, d, 14.8)
**3**	57.8		56.6		56.7	
**4**	35.6	2.13 (1H, m)<br>1.60 (1H, m)	35.1	2.05 (1H, m)<br>1.61 (1H, m)	35.6	2.08 (1H, m)<br>1.53 (1H, m)
**5**	34.7	2.27 (1H, m)<br>2.09 (1H, m)	34.7	2.17 (1H, m)<br>2.05 (1H, m)	34.7	2.27 (1H, m)<br>2.05 (1H, m)
**6**	139.3		139.1		139.3	
**7**	118.8	5.80 (1H, t, 8.1)	118.8	5.88 (1H, t, 8.0)	118.8	5.84 (1H, t, 8.1)
**8**	41.2	3.36 (1H, m)<br>3.25 (1H, m)	41.2	3.29 (2H, m)	41.2	3.38 (1H, m)<br>3.23 (1H, dd, 9.6, 13.0)
**9**	199.9		199.7		199.7	
**10**	131.5	6.01 (1H, d, 16.6)	131.6	5.99 (1H, d, 16.2)	131.5	6.01 (1H, d, 16.2)
**11**	143.7	8.97 (1H, dd, 11.6, 16.6)	143.8	9.01 (1H, dd, 11.4, 16.2)	143.9	9.06 (1H, dd, 11.4, 16.2)
**12**	127.7	6.43 (1H, t, 11.6)	127.6	6.42 (1H, t, 11.1)	127.5	6.43 (1H, t, 11.4)
**13**	129.5	6.80 (1H, d, 11.6)	129.3	6.79 (1H, d, 11.1)	129.2	6.80 (1H, d, 11.4)
**13a**	153.3		153.5		153.5	
**14**	123.3	7.88 (1H, s)	123.6	7.89 (1H, s)	123.5	7.88 (1H, s)
**15**	170.8		171.3		170.8	
**16**	47.6	5.19 (1H, m)	47.9	5.19 (1H, m)	47.6	5.20 (1H, m)
**17**	20.9	1.56 (3H, d, 6.6)	20.7	1.57 (3H, d, 6.5)	20.9	1.57 (3H, d, 6.6)
**18**	16.1	1.64 (3H, s)	16.1	1.63 (3H, s)	16.1	1.64 (3H, s)
**3’**	174.9		174.9		174.8	
**4’**	46.1	3.08 (1H, q, 6.4)	46.4	2.88 (1H, m)	46.4	2.98 (1H, q, 7.0)
**5’**	14.0	1.18 (3H, d, 6.4)	13.6	1.15 (3H, d, 7.0)	13.6	1.14 (3H, d, 7.0)
**6’**			39.5	3.17 (1H, m)<br>3.05 (1H, m)	24.8	2.31 (3H, s)
**7’**	22.6	2.51 (3H, s)	51.8	4.13 (1H, m)	22.6	
**8’**			169.9			
**NH**		8.77 (1H, d, 5.8)		8.77 (1H, d, 5.8)		8.77 (1H, d, 5.8)
**NH_2**				8.42 (2H, m)		

[a] Signals were assigned with aid of ^{1}H-^{1}H COSY, HMBC, and ROESY experiments.
[b] Only the major rotamer is shown.
[c] ^{13}C chemical shifts determined from the indirect dimension of HSQC and HMBC spectra

Supplementary Table 7. ^{1}H NMR (600 MHz) and ^{13}C NMR (150 MHz) data of GNM P1 (**S1**) in methanol-d_4 and GNM P3 (**S3**) in DMSO-d_6.[a] See Supplementary Fig. 9 for carbon numbering, and Supplementary Figs. 71 – 77 and 87 – 93 for spectra.

No.	S1		S3 Major rotamer		S3 Minor rotamer	
	δ_C	δ_H	δ_C	δ_H	δ_C	δ_H
**1**	171.6		170.3		172.0	
**2**	41.4	2.91 (1H, d, 15.3)<br>2.75 (1H, d, 15.3)	40.7	2.87 (1H, d, 14.1)<br>2.40 (1H, d, 14.1)	40.0	3.03 (2H, m)
**3**	53.3		54.0		54.4	
**4**	34.6	1.62 (1H, m)<br>1.50 (1H, m)	34.7	1.61 (1H, m)<br>1.43 (1H, m)	36.0	1.50 (2H, m)
**5**	33.5	1.95 (2H, m)	33.7	1.85 (2H, m)	33.9	1.71 (2H, m)
**6**	134.5		135.3		135.1	
**7**	123.6	4.96 (1H, t, 6.1)	123.2	4.96 (1H, t, 5.6)	124.8	4.86 (1H, d, 7.1)
**8**	32.7	3.00 (2H, m)	33.1	2.97 (1H, m)<br>2.91 (1H, m)	33.2	3.23 (1H, m)<br>2.81 (1H, m)
**9**	146.5		146.3		146.5	
**10**	132.7	6.49 (1H, d, 16.5)	132.6	6.39 (1H, d, 16.1)	132.6	6.41 (1H, d, 16.3)
**11**	127.4	6.90 (1H, d, 16.5)	128.5	7.00 (1H, d, 16.1)	128.9	7.09 (1H, d, 16.3)
**12**	138.7		136.8		136.8	
**13**	121.7	6.36 (1H, s)	124.6	6.36 (1H, s)	124.4	6.34 (1H, s)
**14**	150.4		150.8		150.5	
**15**	117.9	7.33 (1H, s)	118.4	7.41 (1H, s)	118.5	7.51 (1H, s)
**16**	171.4		171.3		173.8	
**17**	32.0		33.6		36.3	
**18**	14.2	1.38 (2H, m)	15.5	1.41 (2H, m)	19.4	1.36 (1H, m)<br>1.11 (1H, m)
**19**	14.3	1.45 (2H, m)	15.0	1.20 (1H, m)<br>1.13 (1H, m)	17.8	1.24 (2H, m)
**20**	18.8	2.06 (3H, s)	20.2	1.97 (3H, s)	20.2	1.97 (3H, s)
**21**	117.7	5.11 (1H, d, 2.1)<br>5.10 (1H, d, 2.1)	118.9	5.10 (2H, br s)	118.9	5.10 (2H, br s)
**22**	15.2	1.62 (3H, s)	16.6	1.57 (3H, s)	16.7	1.58 (3H, s)
**23**	41.4	2.91 (1H, d, 14.0)<br>2.57 (1H, d, 14.0)	40.0	2.96 (1H, m)<br>2.58 (1H, m)	41.3	3.06 (1H, m)<br>3.03 (1H, m)
**24**	172.4		171.9		174.8	
**25**	40.4	3.22 (1H, m)<br>3.05 (1H, dd, 9.3, 13.8)	41.3	3.06 (1H, dd, 4.6 13.5)<br>2.95 (1H, m)	41.3	2.95 (1H, m)<br>2.58 (1H, m)
**26**	53.8	4.64 (1H, dd, 4.2, 9.3)	52.2	4.41 (1H, m)	51.6	4.41 (1H, m)
**27**	171.7		172.4		169.7	
**28**	172.5		169.8		172.6	
**29**	21.4	2.04 (3H, s)	22.8	1.85 (3H, s)	22.8	1.83 (3H, s)
**1-NH**				8.93 (1H, s)<br>8.28 (1H, d, 7.9)		8.78 (1H, s)<br>8.16 (1H, d, 7.9)
**28-NH**	98.7<br>54.2	5.14 (1H, d, 3.9)<br>3.93 (1H, 3.7, 10.5)				
**1'**	98.7	5.14 (1H, d, 3.9)				
**2'**	54.2	3.93 (1H, 3.7, 10.5)				
**3'**	71.4	3.80 (1H, m)				

Supplementary Table 7 cont.

**4'**	70.9	3.34 (1H, m)
**5'**	72.9	3.86 (1H, m)
**6'**	61.4	3.88 (1H, m)
		3.68 (1H, m)
**1''**	79.0	3.51 (1H, dd, 2.7, 9.9)
**2''**	72.0	4.16 (1H, t, 2.7)
**3''**	71.8	3.38 (1H, dd, 2.7, 9.8)
**4''**	72.6	3.63 (1H, m)
**5''**	75.0	3.21 (1H, t, 9.1)
**6''**	72.6	3.83 (1H, m)

[a] Signals were assigned with aid of ^{1}H-^{1}H COSY, HMBC, and ROESY experiments.

Supplementary Table 8. ^{1}H NMR (600 MHz) and ^{13}C NMR (150 MHz) data of GNM P2 (**S2**), GNM P4 (**S4**), and GNM P5 (**S5**) in DMSO-d_6.[a] See Supplementary Fig. 9 for carbon numbering, and Supplementary Figs. 79 – 85, 95 – 101, and 103 – 109 for spectra.

**No.**	**S2**		**S4**		**S5**	
	δ_C	δ_H	δ_C	δ_H	δ_C	δ_H
**1**	172.3		174.3		170.7	
**2**	40.0	2.69 (2H, s)	37.6	2.59 (1H, d, 16.9)<br>2.87 (1H, m)	32.6	2.69 (2H, s)
**3**	59.3		62.0		63.8	
**4**	31.2	1.55 (1H, m)<br>1.33 (1H, m)	26.8	1.45 (1H, m)<br>0.39 (1H, m)	25.6	2.07 (1H, dd, 2.1, 15.2)<br>1.90 (1H, dd, 2.8, 15.2)
**5**	34.4	1.82 (1H, m)<br>2.05 (1H, m)	31.4	1.77 (2H, m)	34.9	1.99 (1H, m)<br>2.28 (1H, td, 2.8, 13.4)
**6**	136.3		133.9		133.3	
**7**	122.8	4.73 (1H, t, 5.2)	122.6	4.82 (1H, t, 6.2)	127.7	5.43 (1H, d, 9.8)
**8**	33.2	2.94 (1H, dd, 5.9, 15.9)<br>2.87 (1H, dd, 5.2, 15.9)	32.5	2.98 (1H, dd, 7.6, 15.2)<br>2.84 (1H, m)	32.3	3.15 (1H, dd, 9.4, 15.4)<br>2.90 (1H, m)
**9**	146.5		145.4		145.6	
**10**	132.5	6.40 (1H, d, 16.1)	132.4	6.39 (1H, d, 16.1)	133.4	6.54 (1H, d, 16.4)
**11**	128.6	6.83 (1H, d, 16.1)	127.3	6.73 (1H, d, 16.1)	127.9	7.91 (1H, d, 16.4)
**12**	137.2		137.1		134.3	
**13**	125.0	6.36 (1H, s)	124.2	6.37 (1H, s)	122.2	6.38 (1H, s)
**14**	149.6		150.8		154.5	
**15**	118.6	7.37 (1H, s)	118.9	7.50 (1H, d, 0.9)	119.3	7.45 (1H, s)
**16**	170.3		167.1		169.3	
**17**	33.0		35.6		35.4	
**18**	15.7	1.15 (1H, m)<br>1.31 (1H, m)	15.8	1.58 (2H, m)	15.6	1.25 (1H, m)<br>1.64 (1H, m)
**19**	13.7	1.06 (1H, m)<br>1.15 (1H, m)	11.6	1.58 (2H, m)	14.8	1.57 (1H, m)<br>1.78 (1H, m)
**20**	20.0	1.97 (3H, s)	19.7	1.98 (3H, d, 1.2)	22.1	2.05 (3H, d, 0.8)
**21**	118.9	5.11 (1H, br s)<br>5.09 (1H, br s)	118.9	5.11 (2H, s)	119.9	5.20 (1H, d, 2.0)<br>5.17 (1H, d, 2.0)
**22**	16.3	1.50 (3H, s)	16.6	1.49 (3H, s)	16.9	1.68 (3H, s)
**23**	41.0	2.74 (1H, d, 14.5)<br>2.52 (1H, m)	33.5	2.65 (1H, d, 17.2)<br>2.59 (1H, d, 17.2)	39.1	2.89 (1H, m)<br>2.72 (1H, d, 16.5)
**24**	171.5		171.6		173.3	
**NH**		9.30 (1H, s)				
**COOH**						12.9 (1H, br s)

[a] Signals were assigned with aid of ^{1}H-^{1}H COSY, HMBC, and ROESY experiments.

Supplementary Table 9. ^{1}H NMR (600 MHz) and ^{13}C NMR (150 MHz) data of **S8** in DMSO-d_6.[a] See Supplementary Fig. 17 for carbon numbering, and Supplementary Figs. 111 – 117 for spectra.

**No.**	**S8 Major rotamer**		**S8 Minor rotamer**	
	δ_C	δ_H	δ_C	δ_H
**1**	170.8		170.8	
**2**	41.1	2.83 (1H, m)<br>2.31 (1H, m)	41.1	2.83 (1H, d, 14.3)<br>2.31 (1H, d, 14.3)
**3**	47.8		48.3	
**4**	34.4	1.47 (1H, m)<br>1.31 (1H, m)	35.5	1.52 (2H, m)
**6**	135.5		136.9	
**7**	123.0	4.98 (1H, t, 6.1)	124.5	4.82 (1H, d, 7.7)
**8**	33.1	2.96 (1H, m)<br>2.91 (1H, m)	33.1	3.23 (1H, m)<br>2.81 (1H, m)
**9**	146.4		146.8	
**10**	132.5	6.38 (1H, d, 16.1)	132.7	6.43 (1H, d, 16.1)
**11**	128.7	7.02 (1H, d, 16.1)	128.6	7.07 (1H, d, 16.1)
**12**	136.7		136.7	
**13**	124.7	6.36 (1H, s)	124.4	6.34 (1H, s)
**14**	150.8		150.1	
**15**	118.5	7.41 (1H, s)	118.9	7.55 (1H, s)
**16**	171.2		174.1	
**17**	33.6		38.7	
**18**	15.6	1.41 (1H, m)<br>1.19 (1H, m)	19.0	1.37 (1H, m)<br>1.12 (1H, m)
**19**	14.9	1.18 (1H, m)<br>1.10 (1H, m)	17.3	1.30 (2H, m)
**20**	20.2	1.96 (3H, d, 1.2)	20.2	1.95 (3H, d, 1.2)
**21**	118.9	5.10 (2H, m)	118.9	5.13 (1H, d, 2.2)<br>5.12 (1H, d, 2.2)
**22**	16.8	1.57 (3H, s)	16.6	1.57 (3H, s)
**23**	41.9	2.94 (1H, d, 14.8)<br>2.47 (1H, m)	38.8	3.07 (1H, d, 15.6)<br>2.92 (1H, m)
**24**	172.2		175.0	
**25**	10.6	1.93 (3H, s)	10.1	1.55 (3H, s)
**NH**		8.79 (1H, s)		8.74 (1H, s)
**NH_2**				

[a] Signals were assigned with aid of ^{1}H-^{1}H COSY, HMBC, and ROESY experiments.

Supplementary Table 10. Characterized *C*-, *O*-, *N*-, and *S*-MTs from the literature used for the phylogenetic analysis in Supplementary Fig. 6.

Name	Accession Number	Function	Reference
GnmP	WP_073890562.1	Persulfide Methyltransferase	This study
ClmM1	CCC55912.1	*S*-Methyltransferase	(*13*)
CrSMT1	AAZ32409.1	*S*-Methyltransferase	(*14*)
Ecm18	4NEC_A	*S*-Methyltransferase	(*15*)
PtyS	ETW97601.1	*S*-Methyltransferase	(*16*)
TioN	CAJ34370.1	Adenylation(A) domain with *S*-methylation function	(*17*)
TmtA	5EGP_A	*S*-Methyltransferase	(*18*)
TPMT	O55060.1	*S*-Methyltransferase	(*19*)
BurB	WP_009894046.1	*S*-Methyltransferase	(*20*)
LmbG	CAA55753.1	*S*-Methyltransferase	(*21*)
DTCMT	XP_009127488.1	*S*-Methyltransferase	(*22*)
DhPI	ACZ13460.1	*O*-Methyltransferase	(*23*)
MmcR	AAD32742.2	*O*-Methyltransferase	(*24*)
NcsB1	Q84HC8.1	*O*-Methyltransferase	(*25*)
MdpB1	ABY66020.1	*C*-Methyltransferase	(*26*)
TcaB9	4E2Y_A	*C*-Methyltransferase	(*27*)
TleD	BAP27961.1	*C*-Methyltransferase	(*28*)
BamL	WP_012117040.1	*N*-Methyltransferase	(*29*)
CypM	E5KIC0.1	*N*-Methyltransferase	(*30*)
DesV1	AAC68678.1	*N*-Methyltransferase	(*31*)
EgtD	WP_158167473.1	*N*-Methyltransferase	(*32*)

Supplementary Table 11. Sequence comparison of representative cystathionine gamma lyase (CGL) proteins. The sequences were selected from strains harboring LNM-type BGCs and compared to CGL from *Streptomyces* sp. CB01883 (WP_073887233). The CGL from *Staphylococcus aureus*, MccB, was included as a reference.[33]

Strain (NCBI protein accession number)	% Identity (similarity) to CB01883_CGL
S. sp. CB01635 (WP_100597190)	84% (92%)
S. sp. CB02959 (PJN41589)	73% (79%)
S. sp. TSRI0384-2 (WP_100457469)	79% (86%)
S. sp. CB01373 (PJM93642)	88% (94%)
S. sp. CB01201 (WP_100575657)	78% (84%)
S. sp. CB02120-2 (PJN19200)	79% (85%)
Saccharothrix espanaensis DSM 44229 (CCH34415)	53% (63%)
S. sp. CB02613 (WP_100563214)	78% (86%)
S. aureus MccB (WP_001036647)	33% (50%)

Supplementary References

1. Navath, R. S., Menjoge, A. R., Wang, B., Romero, R., Kannan, S. & Kannan, R. M. Amino acid-functionalized dendrimers with heterobifunctional chemoselective peripheral groups for drug delivery applications. *Biomacromolecules.* **11**, 1544-1563 (2010).
2. Liu, Z., Wu, Y. & Liu, G. Efficient and convenient total synthesis of mycothiol on a large scale. *J. Chin. Pharm. Sci.* **24**, 347-355 (2015).
3. Zhao, Q., Wang, M., Xu, D., Zhang, Q. & Liu, W. Metabolic coupling of two small-molecule thiols programs the biosynthesis of lincomycin A. *Nature* **518**, 115-119 (2015).
4. Spies, H. S. C. & Steenkamp, D. J. Thiols of intracellular pathogens - Identification of ovothiol A in *Leishmania donovani* and structural analysis of a novel thiol from *Mycobacterium bovis*. *Eur. J. Biochem.* **224**, 203-213 (1994).
5. Tang, G.-L., Cheng, Y. Q. & Shen, B. Leinamycin biosynthesis revealing unprecedented architectural complexity for a hybrid polyketide synthase and nonribosomal peptide synthetase. *Chem. Biol.* **11**, 33-45 (2004).
6. Ma, M., Lohman, J. R., Liu, T. & Shen, B. C-S bond cleavage by a polyketide synthase domain. *Proc. Natl. Acad. Sci. U.S.A.* **112**, 10359-10364 (2015).
7. Huang, S.-X. et al. Leinamycin E1 acting as an anticancer prodrug activated by reactive oxygen species. *Proc. Natl. Acad. Sci. U. S. A.* **112**, 8278-8283 (2015).
8. Pan, G. et al. Discovery of the leinamycin family of natural products by mining actinobacterial genomes. *Proc. Natl. Acad. Sci. U.S.A.* **114**, E11131-E11140 (2017).
9. Nagasawa, T., Kanzaki, H. & Yamada, H. Cystathionine γ-lyase of *Streptomyces phaeochromogenes*. The occurrence of cystathionine γ-lyase in filamentous bacteria and its purification and characterization. *J. Biol. Chem.* **259**, 10393-10403 (1984).
10. Kieser, T., Bibb, M. J., Buttner, M. J., Chater, K. F. & Hopwood, D. A. *Practical Streptomyces Genetics*. The John Innes Foundation, Norwich (2000).
11. Bierman, M., Logan, R., O'Brien, K., Seno, E. T., Rao, R. N. & Schoner, B. E. Plasmid cloning vectors for the conjugal transfer of DNA from *Escherichia coli* to *Streptomyces* spp. *Gene* **116**, 43-49 (1992).
12. Lohman, J. R., Bingman, C. A., Phillips, G. N. & Shen, B. Structure of the bifunctional acyltransferase/decarboxylase LnmK from the leinamycin biosynthetic pathway revealing novel activity for a double-hot-dog fold. *Biochemistry* **52**, 902–911 (2013).
13. Garcia, I. et al. Elucidating the biosynthetic pathway for the polyketide-nonribosomal peptide collismycin A: Mechanism for formation of the 2,2-bipyridyl ring. *Chem. Biol.* **19**, 399-413 (2012).
14. Coiner, H. et al. Methylation of sulfhydryl groups: a new function for a family of small molecule plant *O*-methyltransferases. *Plant. J.* **46**, 193-205 (2006).
15. Hotta, K. et al. Conversion of a disulfide bond into a thioacetal group during echinomycin biosynthesis. *Angew. Chem. Int. Ed. Engl.* **53**, 824-828 (2014).
16. Helf, M. J., Jud, A. & Piel, J. Enzyme from an uncultivated sponge bacterium catalyzes S-methylation in a ribosomal peptide. *ChemBioChem*. **18**, 444-450 (2017).
17. Al-Mestarihi, A. H. et al. Adenylation and *S*-methylation of cysteine by the bifunctional enzyme TioN in thiocoraline biosynthesis. *J. Am. Chem. Soc.* **136**, 17350-17354 (2014).
18. Scharf, D. H., Habel, A., Heinekamp, T., Brakhage, A. A. & Hertweck, C. Opposed effects of enzymatic gliotoxin N- and S-methylations. *J. Am. Chem. Soc.* **136**, 11674-11679 (2014).
19. Peng, Y. et al. Structural basis of substrate recognition in thiopurine *S*-methyltransferase. *Biochemistry* **47**, 6216-6225 (2008).
20. Trottmann, F. et al. Sulfonium acids loaded onto an unusual thiotemplate assembly line construct the cyclopropanol warhead of a *Burkholderia* virulence factor. *Angew. Chem. Int. Ed. Engl.* **59**, 13511-13515 (2020).
21. Ushimaru, R., Lin, C.-I., Sasaki, E. & Liu, H.-W. Characterization of enzymes catalyzing transformations of cysteine *S*-conjugated intermediates in the lincosamide biosynthetic pathway. *ChemBioChem*, **17**, 1606-1611 (2016).

22. Klein, A. P. & Sattely, E. S. Biosynthesis of cabbage phytoalexins from indole glucosinolate. *Proc. Natl. Acad. Sci. U.S.A.* **114**, 1910-1915 (2017).
23. Lee, J.-H. et al. Characterization and structure of DhpI, a phosphonate *O*-methyltransferase involved in dehydrophos biosynthesis. *Proc. Natl. Acad. Sci. U.S.A.* **107**, 17557-17562 (2010).
24. Grüschow, S., Chang, L.-C., Mao, Y. & Sherman, D. H. Hydroxyquinone *O*-methylation in mitomycin biosynthesis. *J. Am. Chem. Soc.* **129**, 6470-6476 (2007).
25. Luo, Y. et al. Regiospecific *O*-methylation of naphthoic acids catalyzed by NcsB1, an *O*-methyltransferase involved in the biosynthesis of the enediyne antitumor antibiotic neocarzinostatin. *J. Biol. Chem.* **283**, 14694-14702 (2008).
26. Ling, J. et al. Enediyne antitumor antibiotic maduropeptin biosynthesis featuring a *C*-methyltransferase that acts on a CoA-tethered aromatic substrate. *J. Am. Chem. Soc.* **132**, 12534-12536 (2010).
27. Fang, J. et al. Cloning and characterization of the tetrocarcin A gene cluster from *Micromonospora chalcea* NRRL 11289 reveals a highly conserved strategy for tetronate biosynthesis in spirotetronate antibiotics. *J. Bacteriol.* **190**, 6014 (2008).
28. Awakawa, T. et al. A methyltransferase initiates terpene cyclization in teleocidin B biosynthesis. *J. Am. Chem. Soc.* **136**, 9910-9913 (2014).
29. Lee, J. et al. Structural and functional insight into an unexpectedly selective *N*-methyltransferase involved in plantazolicin biosynthesis. *Proc. Natl. Acad. Sci. U. S. A.* **110**, 12954 (2013).
30. Zhang, Q. & van der Donk, W. A. Catalytic promiscuity of a bacterial α-*N*-methyltransferase. *FEBS Lett.* **586**, 3391-3397 (2012).
31. Chen, H. et al. Expression, purification, and characterization of two *N*,*N*-dimethyltransferases, TylM1 and DesVI, involved in the biosynthesis of mycaminose and desosamine. *Biochemistry* **41**, 9165-9183 (2002).
32. Seebeck, F. P. In vitro reconstitution of mycobacterial ergothioneine biosynthesis. *J. Am. Chem. Soc.* **132**, 6632-6633 (2010).
33. Lee, D., Jeong, S., Ahn, J., Ha, N.-C. & Kwon, A.-R. Crystal structure of bacterial cystathionine Γ-lyase in the cysteine biosynthesis pathway of *Staphylococcus aureus*. *Crystals* **9**, 656 (2019).
