# Supplementary material for: Thiocysteine lyases as polyketide synthase domains installing hydropersulfide into natural products and a hydropersulfide methyltransferase
Source: Nat Commun. 2021 Sep 28;12:5672. doi: 10.1038/s41467-021-25798-8 (PMC8479088; doi:10.1038/s41467-021-25798-8)
Supplement: Supplementary file 3 — Reporting Summary [file 41467_2021_25798_MOESM3_ESM.pdf]

nature research

Corresponding author(s): Ben Shen

Last updated by author(s): Aug 23, 2021

Reporting Summary

Nature Research wishes to improve the reproducibility of the work that we publish. This form provides structure for consistency and transparency in reporting. For further information on Nature Research policies, see our Editorial Policies and the Editorial Policy Checklist.

Statistics

For all statistical analyses, confirm that the following items are present in the figure legend, table legend, main text, or Methods section.

n/a	Confirmed	
☐	☒	The exact sample size (*n*) for each experimental group/condition, given as a discrete number and unit of measurement
☐	☒	A statement on whether measurements were taken from distinct samples or whether the same sample was measured repeatedly
☒	☐	The statistical test(s) used AND whether they are one- or two-sided<br>*Only common tests should be described solely by name; describe more complex techniques in the Methods section.*
☒	☐	A description of all covariates tested
☒	☐	A description of any assumptions or corrections, such as tests of normality and adjustment for multiple comparisons
☐	☒	A full description of the statistical parameters including central tendency (e.g. means) or other basic estimates (e.g. regression coefficient) AND variation (e.g. standard deviation) or associated estimates of uncertainty (e.g. confidence intervals)
☒	☐	For null hypothesis testing, the test statistic (e.g. *F*, *t*, *r*) with confidence intervals, effect sizes, degrees of freedom and *P* value noted<br>*Give P values as exact values whenever suitable.*
☒	☐	For Bayesian analysis, information on the choice of priors and Markov chain Monte Carlo settings
☒	☐	For hierarchical and complex designs, identification of the appropriate level for tests and full reporting of outcomes
☒	☐	Estimates of effect sizes (e.g. Cohen's *d*, Pearson's *r*), indicating how they were calculated

Our web collection on statistics for biologists contains articles on many of the points above.

Software and code

Policy information about availability of computer code

Data collection	MS data were collected and analyzed by MassHunter B.06.01 (Agilent Technologies) or Freestyle 1.7.73.12 (Thermo Scientific)
Data analysis	Nonlinear regression analysis was performed for kinetic experiments with Prism 5.01 (GraphPad Software, Inc.), NMR data was analyzed with MestReNova 6.0.2-5475

For manuscripts utilizing custom algorithms or software that are central to the research but not yet described in published literature, software must be made available to editors and reviewers. We strongly encourage code deposition in a community repository (e.g. GitHub). See the Nature Research guidelines for submitting code & software for further information.

Data

Policy information about availability of data

All manuscripts must include a data availability statement. This statement should provide the following information, where applicable:

- Accession codes, unique identifiers, or web links for publicly available datasets
- A list of figures that have associated raw data
- A description of any restrictions on data availability

The authors declare that all of the data supporting the findings of this study are available within the manuscript, Extended Data, and Supplementary Information. All raw data is available by request. All sequence data used is publicly available: the leinamycin biosynthetic gene cluster sequence from Streptomyces atroolivaceous S-140 is accessible via the Genbank accession number AF484556, guangnanmycin from S. sp. CB01883 via MF925481, and weishanmycin from S. sp. CB02120-2 via MF925482.

Field-specific reporting

Please select the one below that is the best fit for your research. If you are not sure, read the appropriate sections before making your selection.

- [x] Life sciences
- [ ] Behavioural & social sciences
- [ ] Ecological, evolutionary & environmental sciences

For a reference copy of the document with all sections, see nature.com/documents/nr-reporting-summary-flat.pdf

Life sciences study design

All studies must disclose on these points even when the disclosure is negative.

Sample size	Quantitative experiments were performed with n=3 independent experiments, which is based on the standard sample size used for statistical analysis in biological experiments. Qualitative experiments not requiring statistical analysis were performed with n=2 independent experiments.
Data exclusions	No data was excluded.
Replication	Enzyme assays were confirmed with at least two separate preparations of the enzymes used. Reproducibility of microbial fermentation was verified with biological triplicates. All replication attempts were successful.
Randomization	No randomization was needed for the types of experiments reported in this study (microbial genetics, chemical synthesis and analysis, biochemical assays).
Blinding	Chemical and biochemical analysis experiments do not require blinded experiments, as the data is collected in an unbiased manner.

Reporting for specific materials, systems and methods

We require information from authors about some types of materials, experimental systems and methods used in many studies. Here, indicate whether each material, system or method listed is relevant to your study. If you are not sure if a list item applies to your research, read the appropriate section before selecting a response.

Materials & experimental systems

n/a	Involved in the study	
x		Antibodies
x		Eukaryotic cell lines
x		Palaeontology and archaeology
x		Animals and other organisms
x		Human research participants
x		Clinical data
x		Dual use research of concern

Methods

n/a	Involved in the study	
x		ChIP-seq
x		Flow cytometry
x		MRI-based neuroimaging
