# Supplementary material for: Thiocysteine lyases as polyketide synthase domains installing hydropersulfide into natural products and a hydropersulfide methyltransferase
Source: Nat Commun. 2021 Sep 28;12:5672. doi: 10.1038/s41467-021-25798-8 (PMC8479088; doi:10.1038/s41467-021-25798-8)

Thiocysteine Lyases as Polyketide Synthase Domains Installing Hydropersulfide into Natural Products and a Hydropersulfide Methyltransferase

Authors: Song Meng,^1,a^ Andrew D. Steele,^1,a^ Wei Yan,^1,a^ Guohui Pan,^1^ Edward Kalkreuter,^1^ Yu-Chen Liu,^1^ Zhengren Xu,^1^ Ben Shen^1,2,3,^*

Affiliation: ^1^Department of Chemistry, ^2^Department of Molecular Medicine, and ^3^Natural Products Discovery Center at Scripps Research, The Scripps Research Institute, 130 Scripps Way, Jupiter, FL 33458

^a^These authors contributed equally

*Correspondence to: [shenb@scripps.edu](mailto:shenb@scripps.edu)

**Source Data – Gel Images**

**Original Files for Supplementary Figure 7**


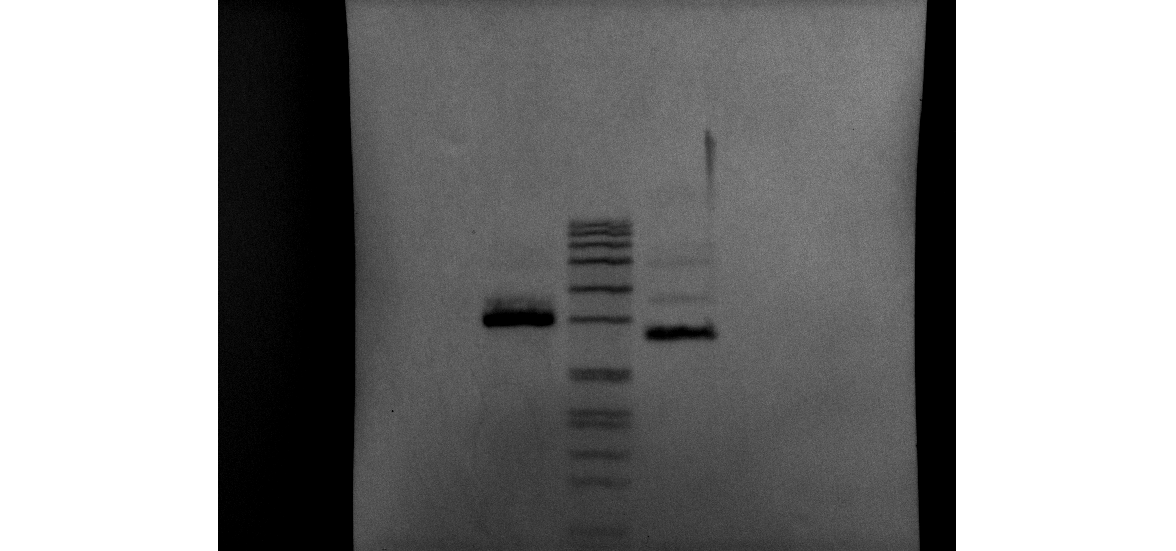


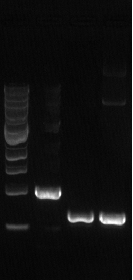


**Original File for Supplementary Figure 11**


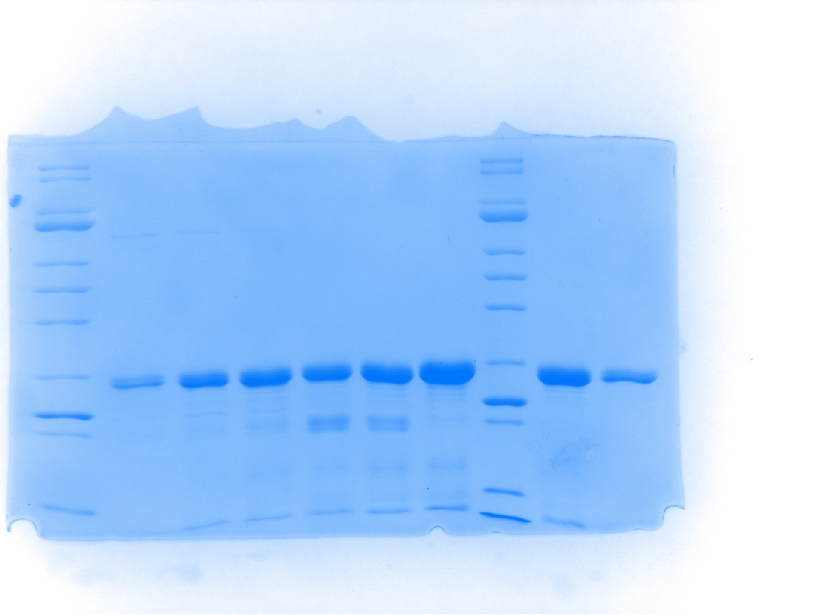


**Original File for Supplementary Figure 12**


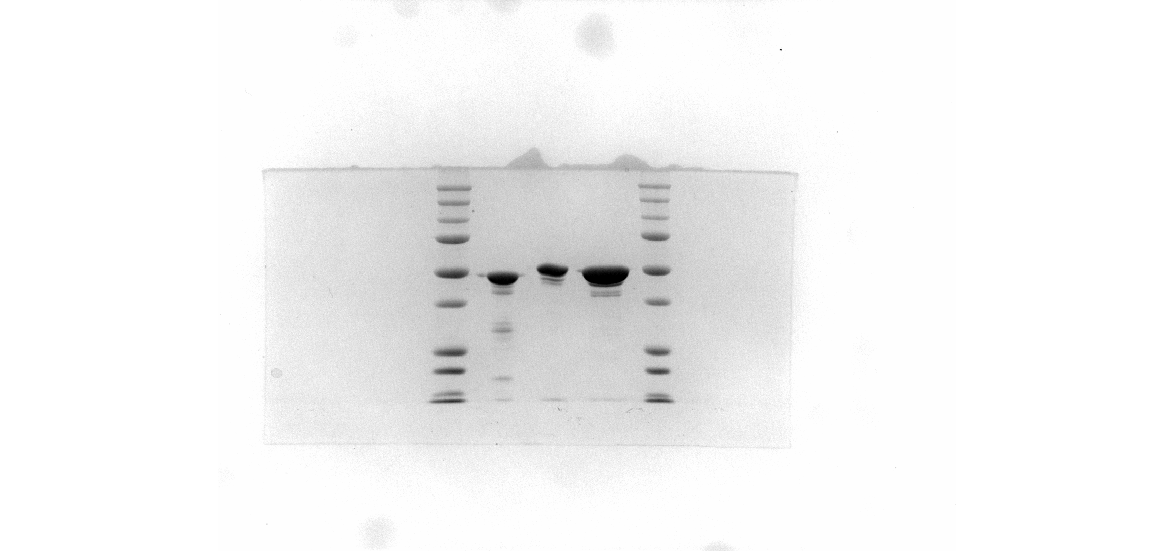


**Original File for Supplementary Figure 22**


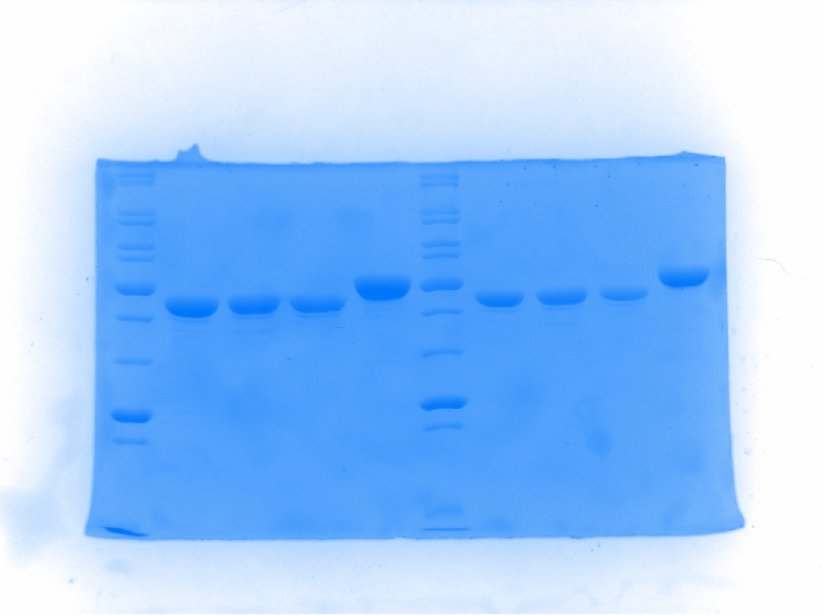

Supplement: Supplementary file 4 — Source data [file 41467_2021_25798_MOESM4_ESM.zip › Source data.docx]
